# Novel hepaci- and pegi-like viruses in native Australian wildlife and non-human primates

**DOI:** 10.1093/ve/veaa064

**Published:** 2020-08-20

**Authors:** Ashleigh F Porter, John H -O Pettersson, Wei-Shan Chang, Erin Harvey, Karrie Rose, Mang Shi, John-Sebastian Eden, Jan Buchmann, Craig Moritz, Edward C Holmes

**Affiliations:** School of Life and Environmental Sciences and School of Medical Sciences, Marie Bashir Institute for Infectious Diseases and Biosecurity, The University of Sydney, Sydney 2006, Australia; School of Life and Environmental Sciences and School of Medical Sciences, Marie Bashir Institute for Infectious Diseases and Biosecurity, The University of Sydney, Sydney 2006, Australia; Department of Medical Biochemistry and Microbiology, Zoonosis Science Center, Uppsala University, Uppsala 752 36, Sweden; School of Life and Environmental Sciences and School of Medical Sciences, Marie Bashir Institute for Infectious Diseases and Biosecurity, The University of Sydney, Sydney 2006, Australia; School of Life and Environmental Sciences and School of Medical Sciences, Marie Bashir Institute for Infectious Diseases and Biosecurity, The University of Sydney, Sydney 2006, Australia; Australian Registry of Wildlife Health, Taronga Conservation Society Australia, Mosman 2088, Australia; School of Medicine, Sun Yat-sen University, Guangzhou, China; School of Life and Environmental Sciences and School of Medical Sciences, Marie Bashir Institute for Infectious Diseases and Biosecurity, The University of Sydney, Sydney 2006, Australia; Westmead Institute for Medical Research, Centre for Virus Research, Westmead 2145, Australia; School of Life and Environmental Sciences and School of Medical Sciences, Marie Bashir Institute for Infectious Diseases and Biosecurity, The University of Sydney, Sydney 2006, Australia; Research School of Biology, Centre for Biodiversity Analysis, Australian National University, Acton, ACT, Australia; School of Life and Environmental Sciences and School of Medical Sciences, Marie Bashir Institute for Infectious Diseases and Biosecurity, The University of Sydney, Sydney 2006, Australia

**Keywords:** hepacivirus, pegivirus, marsupial, metagenomics

## Abstract

The *Flaviviridae* family of positive-sense RNA viruses contains important pathogens of humans and other animals, including Zika virus, dengue virus, and hepatitis C virus. The *Flaviviridae* are currently divided into four genera—*Hepacivirus*, *Pegivirus*, *Pestivirus*, and *Flavivirus*—each with a diverse host range. Members of the genus *Hepacivirus* are associated with an array of animal species, including humans, non-human primates, other mammalian species, as well as birds and fish, while the closely related pegiviruses have been identified in a variety of mammalian taxa, also including humans. Using a combination of total RNA and whole-genome sequencing we identified four novel hepaci-like viruses and one novel variant of a known hepacivirus in five species of Australian wildlife. The hosts infected comprised native Australian marsupials and birds, as well as a native gecko (*Gehyra lauta*). From these data we identified a distinct marsupial clade of hepaci-like viruses that also included an engorged *Ixodes holocyclus* tick collected while feeding on Australian long-nosed bandicoots (*Perameles nasuta*). Distinct lineages of hepaci-like viruses associated with geckos and birds were also identified. By mining the SRA database we similarly identified three new hepaci-like viruses from avian and primate hosts, as well as two novel pegi-like viruses associated with primates. The phylogenetic history of the hepaci- and pegi-like viruses as a whole, combined with co-phylogenetic analysis, provided support for virus-host co-divergence over the course of vertebrate evolution, although with frequent cross-species virus transmission. Overall, our work highlights the diversity of the *Hepacivirus* and *Pegivirus* genera as well as the uncertain phylogenetic distinction between.

## 1. Introduction

As the vast majority of emerging infectious disease in humans are caused by viral zoonoses (Jones et al. 2008), the characterisation of animal viruses is critical for identifying potential disease reservoirs and providing models for the study of human viruses (Pfaender et al. 2014, 2015). Two related groups of viruses that have repeated emerged in new hosts are the genera *Hepacivirus* and *Pegivirus* from the family *Flaviviridae* of single-strand positive-sense RNA viruses. Hepaciviruses and their close phylogenetic relatives (i.e. ‘hepaci-like’ viruses) infect a broad range of vertebrates, including humans, non-human primates (Lauck et al. 2013; Canuti et al. 2019), and a variety of other mammals including rodents (Drexler et al. 2013; Kapoor et al. 2013; Firth et al. 2014; Wu et al. 2018; de Souza et al. 2019), horses (Burbelo et al. 2012), bats (Quan et al. 2013), and cattle (Baechlein et al. 2015; Corman et al. 2015). Hepaci-like viruses have also been detected in birds (Chu et al. 2019; Goldberg et al. 2019), fish, and a variety of other vertebrates (Shi et al. 2016a, 2018). In addition, two hepaci-like virus sequences have been identified in arthropods (a mosquito and tick), although their true host is uncertain (Harvey et al. 2019a; Williams et al. 2020). Despite such host diversity, hepaciviruses remain synonymous with liver infection, with the most notable example being human hepatitis C virus (HCV). While some non-primate hepaci-like viruses have been well characterised, particularly equine hepacivirus (also known as hepacivirus AK, or non-primate hepacivirus) and canine hepacivirus (CHV), it seems likely that HCV arose from a currently unknown zoonotic source (Pybus and Gray 2013; Lyons et al. 2014; Thézé et al. 2015; Hartlage, Cullen, and Kapoor 2016).

The genus *Pegivirus* (and the related ‘pegi-like’ viruses) currently contain eleven defined virus species that infect humans and variety of other mammals (Stapleton et al. 2011), with a novel avian pegivirus recently identified in a Common myna bird (*Acridotheres tristis*) (Chang et al. in preparation). Human pegivirus (HPgV), previously known as GB virus C, infects humans with an unproven link with clinical illness (Tuddenham et al. 2020). Non-human primate pegiviruses have been identified in New World monkeys (Schaluder et al. 1995; Bukh and Apgar 1997), Old World monkeys (Sibley et al. 2014), and chimpanzees, the latter of which (SPgV_cpz_) is closely related to HPgV (Simons et al. 1995; Adams et al. 1998; Birkenmeyer et al. 1998). Other pegiviruses have been found in horses (Chandriani et al. 2013), bats (Epstein et al. 2010; Quan et al. 2013), and rodents (Drexler et al. 2013; Kapoor et al. 2013).

Recently, a novel hepaci-like virus, Brushtail possum hepacivirus, was discovered in brush-tailed possums (*Trichosurus vulpecula*), a native Australian marsupial (Chang et al. 2019). Similarly, Collins beach hepacivirus was identified in a tick (*Ixodes holocyclus*) feeding on another Australian marsupial (the long-nosed bandicoot, *Perameles nasuta*) (Harvey et al. 2019a). These data suggest that Australian native wildlife harbour a diversity of hepaci-like viruses and compatible with the idea that these viruses may perhaps be transmitted by biting arthropod vectors (Pybus and Thézé 2016), although this is yet to be formally demonstrated.

Despite their broad host range, all hepaci- and pegi-like viruses described to date possess a single-strand positive-sense RNA genome and a large open reading frame encoding a single polyprotein flanked by untranslated regions. Although this structure is common among the *Flaviviridae*, a more diverse set of genome structures, including segmented forms, have been identified in some invertebrate flaviviruses (Shi et al. 2018). The multifunctional polyprotein is cleaved by proteases to create ten proteins: three structural (core, E1, E2) and seven non-structural (NS1, NS2, NS3, NS4a, NS4b, NS5a, NS5b) (Hartlage, Cullen, and Kapoor 2016).

The recent expansion in the identification of animal hepaci- and pegi-like viruses reflects the advent of unbiased high-throughput sequencing. Herein, we used a total RNA-sequencing (meta-transcriptomic) approach to identify additional novel hepaci-like viruses in Australian wildlife. This approach has previously proven successful in identifying a variety of viruses in Australian wildlife, including native Australian and invasive species (Shi et al. 2017; Mahar et al. 2018; Wille et al. 2018; Harvey et al. 2019a,b,c). To supplement this analysis, we mined the sequence read archive (SRA) database for hepaci- and pegi-like virus sequences.

## 2. Materials and methods

### 2.1 Animal ethics

The magpie-lark and pelican were handled under a series of NSW Office of Environment and Heritage Licences to Rehabilitate Injured, Sick, or Orphaned Protected Wildlife (No. MWL000100542). Samples were collected under the Opportunistic Sample Collection Program of the Taronga Animal Ethics Committee, and scientific licence SL100104 issued by the NSW Office of Environment and Heritage. Ticks were removed from the long-nosed bandicoot under the approval of the NSW Office of Environment and Heritage Animal Ethics Committee (No. 000214/05) and scientific licence SL100104.

### 2.2 Sample collection

The samples analysed here were collected from a variety of sources (Table 1). Samples included in this study were either suspected to have an active infection (magpie-lark, koala, and pelican samples) or were involved in routine sample collection and sequencing. Gecko hepaci-like virus (GHV) (RNASeq library VERT7) was obtained from a liver sample (No. CCM0247) of a seemingly healthy gecko (*Gehyra lauta*) collected at West Leichardt Station, Queensland in 2013 (Oliver et al. 2020). Pelican hepaci-like virus (PeHV) (RNASeq library VERT5) was obtained from the liver (Australian Registry of Wildlife Health No. 9381.1) of an Australian pelican (*Pelecanus conspicillatus*) collected at Blackwall Bay, New South Wales (NSW) in 2013. The pelican presented with an ongoing syndrome of profound weakness, diarrhoea, dyspnoea, and death, consistently associated with myocardial degeneration to necrosis. Magpie-lark hepaci-like virus (MaHV) (Australian Registry of Wildlife Health No. 9585.8) was obtained from brain (RNASeq library VERT14) and heart samples (RNASeq library VERT 15) of an injured juvenile magpie-lark (*Grallina cyanoleuca*) collected at Warigee, NSW in 2013 (Chang et al. 2020). Collins Beach virus 1 (CBV1) was identified from three engorged female ticks (*I.holocyclus*) collected while feeding on a healthy long-nose bandicoot (*P.nasuta*) at North Head, NSW in 2016. These individual tick samples were part of a larger pool of ticks (RNASeq library TICK08, SRA projects SRS3932533 and SRS3932534) used to identify the partial genome sequence of Collins Beach virus (Harvey et al. 2019a). The Koala hepaci-like virus (KHV) was identified during our initial SRA mining of marsupial transcriptomes (Supplementary Table S1). In this case, the mRNA library (SRX501262) was prepared from the liver RNA of a deceased Australian koala (*Phascolarctos cinereus*) ‘Pacific Chocolate’ infected with chlamydia (Johnson et al. 2018). As viral coverage was incomplete due to the use of poly-A selection, the original tissue sample (No. M.45022.004) was kindly provided by the Australian Museum and subjected to total RNA sequencing (i.e. rRNA-depletion only) along with our other cases.

**Table 1.** Sample information for the novel hepaci-like viruses identified in Australian wildlife, including the host species, library, region isolated, host pathology, and assembly data.

Virus name	Acronym	Host (common)	Host (scientific)	RNA library (tissue)	Region	Pathology	Viral reads per library (abundance %)	Host marker reads per library (abundance %)^a^
Magpie-lark hepaci-like virus	MaHV	Magpie-lark	*G.cyanoleuca*	VERT14 (brain); VERT15 (heart)	Nowra, NSW	Hepatic rupture, urate nephrosis, ventricular haemorrhage, thin	VERT14 = 26 (0.00005%)	VERT14 = 3,095 (0.0063%)
							VERT15 = 172 (0.00045%)	VERT15 = 5,140 (0.013%)
Collins beach virus 1	BaHV	Long-nosed bandicoot	*P.nasuta*	TICK07 (tick); TICK08 (tick); TICK10 (tick); INVERT13 (tick)	Sydney, NSW	Healthy	TICK07 = 6 (0.00001%)	TICK07 = 19,752 (0.035%)
							TICK08 = 430 (0.00079%)	TICK08 = 33,706 (0.062%)
							TICK10 = 6 (0.00001%)	TICK10 = 6,274 (0.012%)
							INVERT13 = 43 (0.00014%)	INVERT13 = 12,207 (0.041%)
Koala hepaci-like virus	KHV	Koala	*P.cinereus*	VERT20 (liver)	Port Macquarie, NSW	Severe chlamydiosis	VERT20 = 29,391 (0.10%)	VERT20 = 3,205 (0.011%)
Pelican hepaci-like virus	PeHV	Australian pelican	*P.conspicillatus*	VERT5 (liver); VERT44 (liver)	Blackwall Bay, NSW	Profound weakness, diarrhoea, dyspnoea, and death, associated with myocardial degeneration to necrosis	VERT5 = 172 (0.00034%)	VERT5 = 8,642 (0.017%)
							VERT44 = 362 (0.0014%)	VERT44 = 2,978 (0.011%)
Gecko hepaci-like virus	GHV	House gecko	*G.lauta*	VERT7 (liver)	West Leichardt Station, QLD	Healthy	VERT7 = 762 (0.0018%)	VERT7 = 6,651 (0.015%)

^a^ Abundance of host determined using RPL13A.

### 2.3 RNA extraction, library preparation, and sequencing

Total RNA was extracted from individual animal tissues and whole ticks using the RNeasy Plus Mini Kit (Qiagen, Germany). For animal tissues, ∼50 mg pieces were prepared from livers and other organs by dissecting on a sterile and cool surface before homogenisation in 1 ml of lysis buffer (RLT Plus with 0.1% (v/v) β-mercaptoethanol and 0.5% (v/v) reagent DX) using a TissueRuptor (Qiagen, Germany) for 5 min at maximum speed. Whole ticks were similarly processed except were pre-washed in cold, sterile 1X PBS before homogenisation in lysis buffer. Lysates were then purified as per the manufacturers protocol before elution in nuclease-free water. RNA concentration and integrity were determined using a NanoDrop spectrophotometer (ThermoFisher) and TapeStation (Agilent). RNA samples were pooled in equal proportions based on animal tissue types and syndrome (maximum eight individuals per library). Illumina TruSeq stranded RNA libraries were prepared on the pooled samples following rRNA depletion using a RiboZero Gold kit (Epidemiology) at the Australian Genome Research Facility (AGRF), Melbourne. The rRNA depleted libraries were then sequenced on an Illumina HiSeq 2500 system (paired-end 100 or 125 bp reads) to depths of between 13 and 28 million paired reads.

### 2.4 Viral discovery pipeline

We employed an established meta-transcriptomic pipeline developed for RNA virus discovery (Shi et al. 2016b, 2018; Harvey et al. 2019a). RNA sequences were trimmed of low-quality bases and adapter sequences with Trimmomatic v0.36 (Bolger, Lohse, and Usadel 2014) before *de novo* assembly using Trinity 2.5.1 (Grabherr et al. 2011). The assembled contigs were then compared against the NCBI nucleotide (nt) and non-redundant (nr) protein databases with Blastn v2.7.1+ (Camacho et al. 2009) and Diamond v0.9.18 (Buchfink, Xie, and Huson 2015), respectively, with an e-value thresholds of 1E−5. Contig abundance was estimated by calculating the number of reads in each library that mapped to the hepacivirus genome divided by the number of total reads (Supplementary Table S2). Similarly, host abundance was compared by mapping reads to a reference gene—ribosomal protein L13a (RPL13A). Low abundance viruses (i.e. those that could not be assembled) were identified by aligning the trimmed reads against either NCBI viral RefSeq or a curated hepacivirus/pegivirus protein database using Diamond v.0.9.18, with an e-value threshold of 1E−4. Curated hepacivirus/pegivirus databases were regularly updated with novel viruses to increase read alignment for divergent viral species.

### 2.5 PCR confirmation and whole-genome sequencing

To confirm the presence of novel viruses in individual samples, RT-PCR was performed using primers designed to amplify a short region of the viral genome (<300 bp). Briefly, Superscript IV VILO master mix was used to generate cDNA from individual RNA samples before RT-PCR screening with Platinum SuperFi following the manufacturers recommended protocol. Following the confirmation of viruses to individual samples, long overlapping PCRs (2–4 kb) were designed from the mapped RNASeq data to amplify the available viral genome (i.e. complete or partial) for the hepaciviruses identified here using Platinum SuperFi. Viral amplicons were then pooled, purified, and sequenced using the Nextera XT library prep kit and an Illumina MiSeq (150 nt paired reads). Viral genomes were then assembled using MegaHit v1.1.3 (Li et al. 2016).

### 2.6 SRA mining

To identify hepaci- and pegi-like viruses present in the SRA, we screened primates (excluding *Homo sapiens*), birds (excluding *Gallus gallus*) and marsupials. We focused on these taxonomic groups because 1, although HCV is clearly a human virus it is uncertain whether it is present in non-human primates (which may be indicative of virus-host co-divergence), 2, a number of novel hepaci- and pegi-like viruses have recently been identified in avian hosts suggesting that these might be a rich source of novel viruses, and 3, to confirm the presence of a marsupial specific-lineage as suggested by our previous studies. Accordingly, three different sets of single- and paired-end RNA sequencing SRAs were analysed: one included 2,312 avian SRAs (Supplementary Table S3), a second that included 3,902 primate data sets (Supplementary Table S4) and finally a marsupial data set of 330 SRAs (Supplementary Table S1). SRAs were fetched with the NCBI SRA toolkit using fastq-dump and analysed using our virus discovery pipeline, with the exception that the initial viral screening was performed by aligning the downloaded reads to our curated hepacivirus/pegivirus protein database with Diamond v.0.9.18. SRAs that contained hepaci- and/or pegi-like virus reads were then assembled and annotated as above.

Three virus fragments were identified in three runs (SRR1325073, SRR1325072, SRR1325074) from SRA project SRX565268, isolated from Eurasian blue tit (*Cyanistes caeruleus*) from Germany. These three fragments were concatenated into a single genome, Blue tit hepaci-like virus (BtHV). The three fragments, and their respective coding regions, are shown in Supplementary Fig. S1.

### 2.7 Genome annotation

The hepaci- and pegi-like viruses identified in this study were subjected to an online sequence similarity search using NCBI blastx as well as a conserved domain (CDD) search. Genome annotation was performed using Geneious Prime (version 2019.2.1) (Kearse et al. 2012). Specifically, using the Live Annotate and Predict tool, each novel genome was screened for hepaci- and pegi-like virus specific gene annotations utilising those previously identified in viral genomes downloaded from NCBI (n = 145). To maximise the identification of hypothetical genes, even in divergent genomes, the gene similarity cut-off was set to 25 per cent, with predicted genes then manually annotated.

### 2.8 Inferring phylogenetic relationships

To investigate the evolutionary relationships among the hepaci- and pegi-like viruses we collated the amino acid sequences for the complete polyprotein of each genome from the ten novel viruses identified here, and from related viruses taken from the NCBI Refseq database, including both Brushtail possum hepacivirus and Collins Beach hepacivirus (accession numbers available in Supplementary Table S5). Sequences were aligned using the E-INS-i algorithm in MAFFT v7 (Katoh and Standley 2013), with ambiguous regions removed using GBlocks (Talavera and Castresana 2007). The final sequence alignment comprised 110 sequences of 1,345 amino acid residues in length. ProtTest v3.4 (Darriba et al. 2011) was used to determine the most appropriate model of amino acid substitution for these data. From these data, we estimated a phylogenetic tree using the maximum likelihood method in IQ-TREE, version 1.6.12 (Nguyen et al. 2015), employing the LG model of amino acid substitution with invariable sites and gamma model (four categories), along with a bootstrap resampling analysis using 1,000 replicates.

To determine if the evolutionary history of the hepaci- and pegi-like viruses was characterised by recombination, we screened for recombination signals using the methods (GENECONV, Bootscan, Maxchi, Chimaera, SiScan, PhylPro, LARD and 3Seq) available in the RDP4 package, assuming default parameters (Martin et al. 2015). Complete genome nucleotide sequence alignments were utilised as described above. Phylogenetic trees either side of the putative breakpoints identified for each hypothetical recombinant region (see Results section) were estimated using the same procedure as described above (but with one hundred bootstrap replicates). In addition, two additional phylogenetic trees were estimated for the NS2/3 (549 amino acid residues) and NS5 (407 amino acid residues) regions using the same procedure as described above (with one hundred bootstrap replicates).

To determine the relative frequencies of co-divergence and cross-species transmission in the evolutionary history of hepaci- and pegi-like viruses and their vertebrate hosts, we utilised the co-phylogenetic method available in the Jane package (version 4) (Conow et al. 2010). To this end a simplified virus phylogeny (i.e. removing viruses where an equivalent genome sequence was unavailable; total data set size = 64) was inferred using the methods as described above, while a tree of the vertebrate host species was inferred using TimeTree (Kumar et al. 2017). As in previous studies, we assigned the ‘event costs’ were set to 0 for co-divergence, 1 for duplications, 1 for host-switching, and 1 for extinction (Geoghegan et al. 2018; Shi et al. 2018). TreeMap3 (Charleston 2011) was used to visualise the data in tanglegrams, displaying the putative connections between each virus and each host species (employing the ‘untangle’ function).

## 3. Results

### 3.1 Novel hepaci-like viruses in Australian wildlife

**Figure 1.**
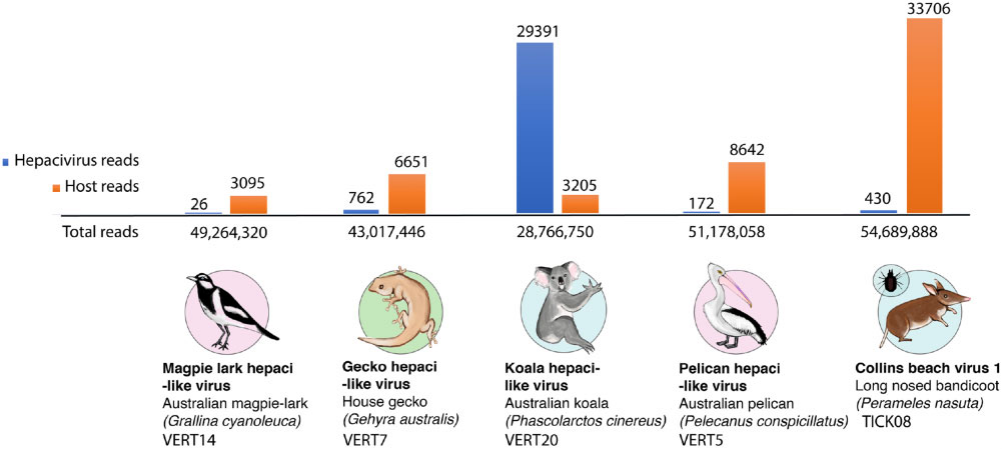
Abundance of hepacivirus-like contigs in each RNASeq library. The relative abundance of hepaci-like virus contigs (shown in blue) is presented as the number of hepaci-like virus reads compared to the number of host reads (based on the RPL13A gene, shown in orange) and the total number of reads each library (shown on the horizontal axis).

We identified four novel hepaci-like viruses and one new hepacivirus variant from five meta-transcriptomic libraries representing a variety of Australian wildlife samples (Table 1). The four novel hepaci-like viral genomes were: 1, KHV; 2, PeHV; 3, MaHV; and 4, GHV. We have assigned these as ‘hepaci-like’ viruses as they await formal classification by the International Committee on Taxonomy of Viruses. In addition, we identified a variant of the previously identified Collins beach virus isolated from ticks feeding on long-nosed bandicoots (Harvey et al. 2019a) and termed CBV1. The relative abundance of hepaci-like virus sequences in each library was low (Fig. 1 and Table 1), with the abundance of the host RPL13A gene shown for comparison. The KHV was the most abundant, comprising 0.1 per cent of the total reads and a complete genome could be assembled at a mean coverage depth of 386×. CBV1 reads were at an abundance <1 per cent in all tick libraries. The abundance levels for the remaining novel hepaci-like viruses ranged from 0.0018 to 0.00001 per cent of the reads in their respective libraries, and most of the genomes were incomplete. Therefore, for these remaining viruses, we used a long, overlapping amplicon-based sequencing approach to fill gaps in the RNASeq data and from this produce- or near-complete virus genomes.

### 3.2 Novel hepaci- and pegi-like viruses discovered through SRA data mining

Three hepaci-like virus sequences and two pegi-like virus sequences were identified through SRA data mining (Table 2). The hepaci-like virus genomes identified were isolated from 1, a Diademed sifaka (*Propithecus diadema*) sampled in Toamasina province, Madagascar, and termed Sifaka hepaci-like virus (SfHV-mad), 2, a Senegal bushbaby (*Galago senegalensis*) virus termed Bushbaby hepaci-like virus (BbHV), and 3, three hepaci-like virus fragments recovered from a Eurasian blue tit (*C.caeruleus*) in Montpellier, France, that were compiled into a viral single genome termed BtHV. Similarly, two pegi-like virus sequences were isolated from a 1, Common marmoset (*Callithrix jacchus*) termed Marmoset pegi-like virus (MHV), and 2, a South African Vervet monkey (*Chlorocebus pygerythrus*) that were assembled into a single viral genome termed Simian pegi-like virus (SPV-saf). Finally, we identified a partial fragment of KHV in the SRA screen (library SRX501262) that we re-sequenced to obtain a complete viral genome (VERT20). All individual sequence fragments are described in the Supplementary materials.

**Table 2.** The five novel hepaci- and pegi-like identified from SRA-mining, including host information, project runs (SRR) and SRA project.

Virus name	Acronym	Host (common)	Host (scientific)	Runs (SRR)	SRA project
Blue tit hepaci-like virus	BtHV	Eurasian blue tit	*C.caeruleus*	SRR1325073, SRR1325072, SRR1325074	SRX565268
Bushbaby hepaci-like virus	BbHV	Senegal bushbaby	*G.senegalensis*	SRR361358	SRX104357
Sifaka hepacivirus	SfHV-mad	Diademed sifaka	*P.diadema*	SRR3131110	SRX1550742
Marmoset pegi-like virus	MPV	Common marmoset	*C.jacchus*	SRR1758976	SRX843207
Simian pegi-like virus	SPV-saf	Vervet monkey	*C.pygerythrus*	SRR1046735, SRR1046733	SRX389659

### 3.3 Genome annotation of novel viruses

**Figure 2.**
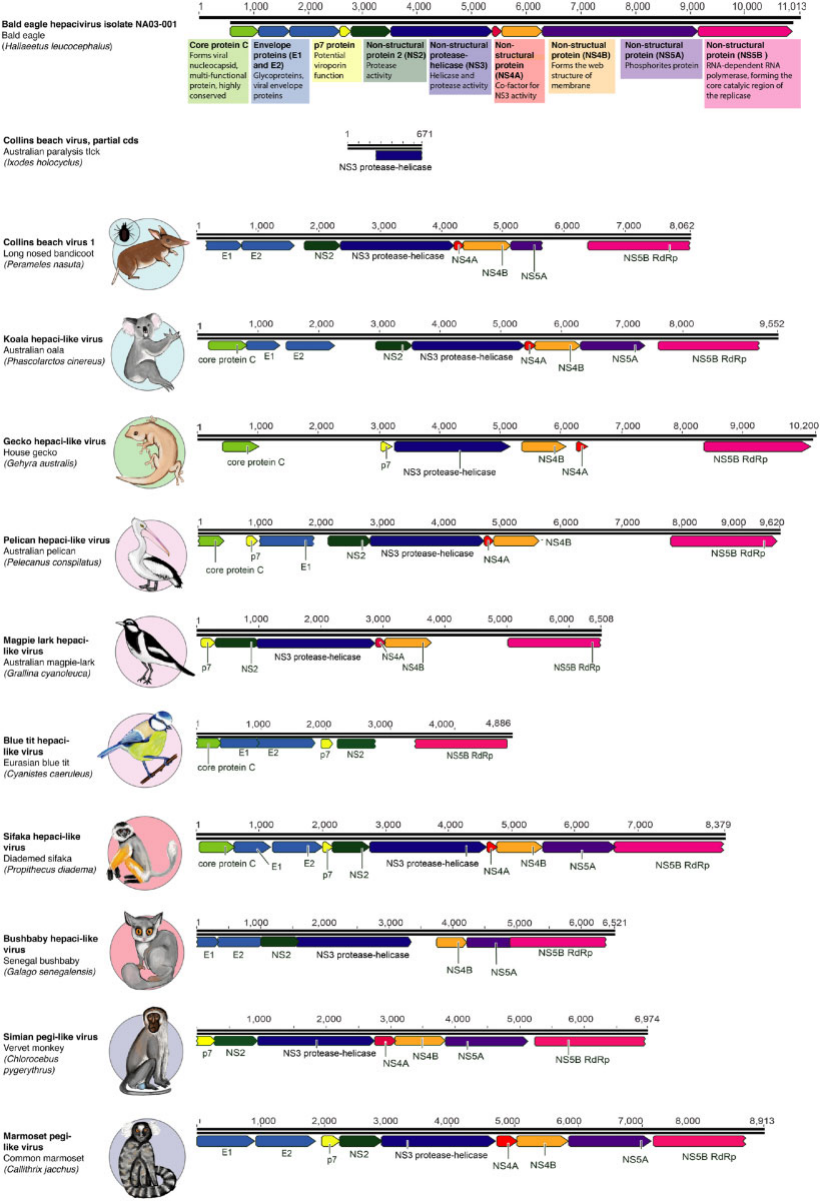
Genome annotation of the novel viruses identified in this study. The genome annotation of each virus is shown in comparison to Bald eagle hepacivirus (isolate NA03-001, accession MN062427) as a reference. The conserved flavivirus polyprotein is cleaved into several protein products, represented in the diagram as coloured arrows. A short description of the function of each protein (Krekulová, Rehák, and Riley 2006; Moradpour, Penin, and Rice 2007) is shown underneath the Bald eagle hepacivirus genome, highlighted in coloured boxes that refer to each protein.

Each of the novel hepaci-like viruses identified here, as well as CBV1, underwent genome annotation and were compared to the fully annotated Bald eagle hepacivirus as a reference (Fig. 2) (Krekulová, Rehák, and Riley 2006; Moradpour, Penin, and Rice 2007; Goldberg et al. 2019). All of the novel viruses encoded near-complete polyproteins. Two relatively well-conserved proteins, the NS5B protein that encodes the RNA-dependent RNA polymerase (RdRp) and the NS3 protease–helicase protein, were present in all cases. The remaining flavivirus proteins—core (C), protein p7, envelope protein E1, envelope protein E2, NS2, NS4A, NS4b, NS5A—were identified in most novel virus genomes, although we were unable to successfully identify NS5A in any of the novel bird or reptile hepaci-like viruses at our threshold levels (25% sequence similarity). Two of the SRA-derived viral sequences— BtHV and SPV-saf—comprised partial genomes and did not include all annotations. The more divergent GHV was similarly not fully annotated. The genome regions missing from the annotation were the envelope proteins (E1 and E2) and the non-structural proteins NS2 and NS5A (Fig. 2). That all of the novel viruses have near-complete polyprotein encoding regions suggests that successful annotation has simply been prevented by high levels of sequence divergence in some genes.

### 3.4 Phylogenetic relationships and evolutionary history of novel viruses

**Figure 3.**
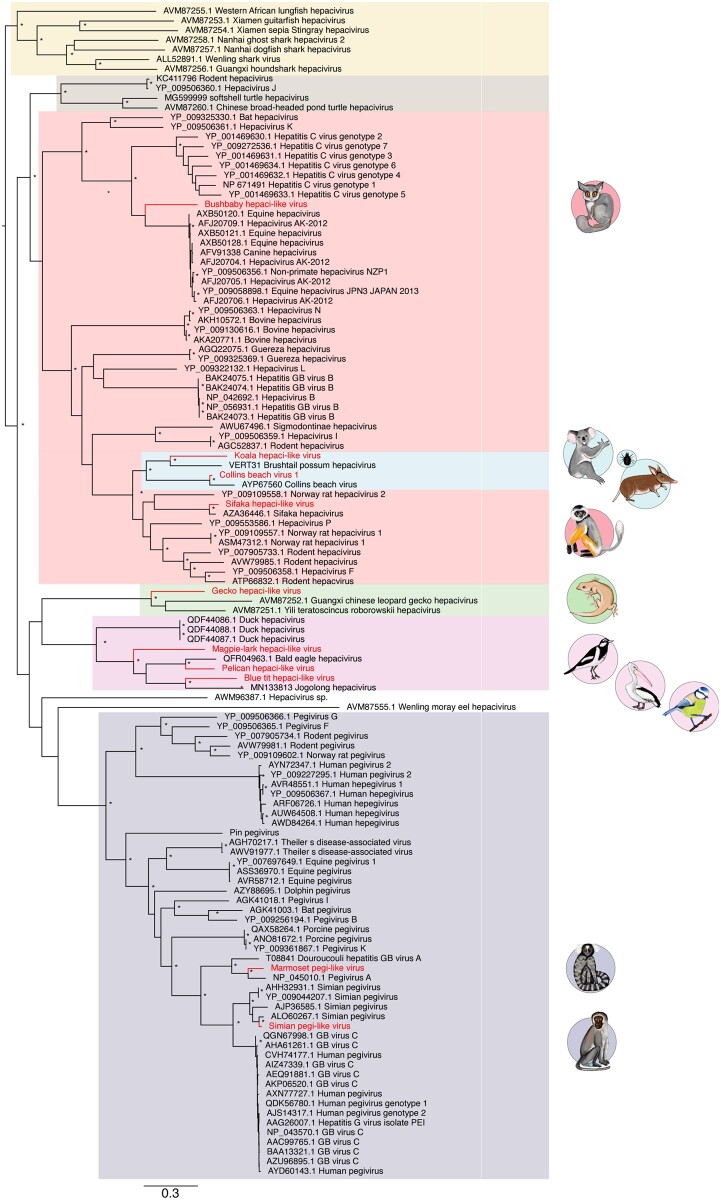
Phylogenetic analysis of the novel viruses identified in this study, identified by animal symbols, along with previously determined hepaci-and pegi-like viruses. Novel BtHV, MaHV, and PeHV, and other avian hepaciviruses are shown in the pink box, while the novel GHV is highlighted in the green box. Fish- and shark-associated hepaci-like viruses are shown in the yellow box, while turtle-associated hepaci-like viruses are shown in the brown box. Marsupial hepaci-like viruses, including those identified in bandicoots, their ticks, and koalas, are shown in the blue box. BbHV and Simian hepaci-like virus fall in the mammalian clade shown in the red box. Finally, two novel pegi-like viruses, MHV and SPV-saf, are shown in the purple box. Bootstrap values greater than 70 per cent are shown next to the relevant nodes, represented by an asterisk. All horizontal branch lengths are scaled according to the number of amino acid substitutions per site. The tree is rooted on the fish hepaci-like viruses under the assumption of virus-host co-divergence.

To determine the evolutionary relationships of the novel hepaci- and pegi-like viruses we utilised complete polyprotein amino acid sequences (Fig. 3). Maximum likelihood phylogenetic analysis revealed that the three avian hepaci-like viruses identified here—Australian MaHV, PeHV, and BtHV—fall with known bird hepaciviruses. This clade also includes the three variants of duck hepacivirus (DuHV) (Chu 2019), Bald eagle hepacivirus (Chu et al. 2019; Goldberg et al. 2019), and Jogolong hepacivirus, recently isolated from a mosquito feeding on a bird host in Western Australia (Williams et al. 2020) (see below). It is also noteworthy that the newly identified GHV clusters with other GHVs—Yili teratoscincus roborowskii hepacivirus and Guangxi Chinese leopard GHV (both sampled in China)—in a distinct gecko clade (Fig. 3).

The mammalian hepaci-like virus clade is the largest and best characterised and contains four of the novel viruses identified in this study, including the two primate hepaci-like viruses identified via SRA mining (Fig. 3). SfHV-mad groups with a SfHV identified previously (Canuti et al. 2019): both viruses were isolated from different samples of *P.diadema* and share 91 per cent amino acid identity. These two viruses cluster with a group of rodent hepaciviruses. In contrast, BbHV, sampled from a Senegal bushbaby, groups with the equine and CHVs, with HCV falling as a sister-group to this clade. Importantly, however, as this part of the phylogeny has poor bootstrap support, it is uncertain whether BbHV is more closely related to the equine/ CHVs or to HCV.

The remaining novel hepaciviruses identified in the mammalian clade were associated from marsupials: the Australian koala and a tick bloodmeal from a long-nosed bandicoot in the case of CBV1. The previously identified Brushtail possum hepacivirus and Collins beach hepacivirus fall into this clade. Notably, the partial sequence of Collins beach hepacivirus was found in the same pooled tick libraries as the CBV1 described here and they share 74 and 89 per cent identity at the nucleotide and amino acid levels, respectively. The ticks that made up these libraries were sampled from long-nosed bandicoots in New South Wales, Australia (Harvey et al. 2019a). This marsupial cluster is itself most closely related to the rodents and sifaka hepaciviruses.

Two novel pegi-like viruses were identified from the SRA—SPV-saf and MHV—and occupy phylogenetic positions suggestive of virus-host co-divergence (Fig. 3 and Table 2). Specifically, SPV-saf, identified in a Vervet monkey sample, groups with other Old World primate-associated pegiviruses, including those isolated from Yellow baboons (*Papio cynocephalus*) from Tanzania (Bailey et al. 2015), African green monkey (*Chlorocebus sabaeus*) from Gambia, and Red colobus monkey (*Piliocolobus tephrosceles*) from Uganda (Sibley et al. 2014). In contrast, MHV, isolated from a common marmoset, falls with New World primate-associated pegiviruses (Pegivirus A, Douroucouli hepatitis GB virus A), identified in tamarin monkeys (*Saguinus labiatus*), mystax monkeys (*Saguinus mystax*) and owl monkeys (*Aotus trivirgatus*) (Leary et al. 1996).

**Figure 4.**
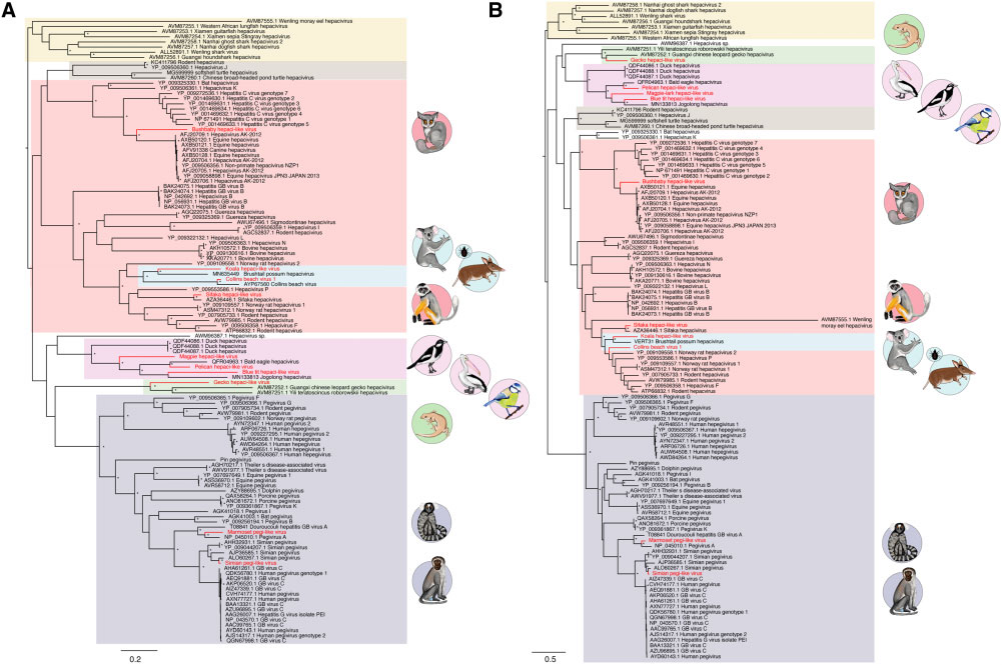
Phylogenetic analysis of the (A) NS2/NS3 and (B) NS5 amino acid sequences from the novel hepaci- and pegi-like viruses identified in this study, marked by animal symbols, combined with known hepaciviruses and pegiviruses. Animal groups are shaded as described in Fig. 3. Note that Collins beach virus is excluded from the NS5 phylogeny as it lacks this gene region. Bootstrap values greater than 70 per cent are shown next to the relevant nodes, represented by an asterisk. All horizontal branch lengths are scaled to the number of amino acid substitutions per site. The tree is rooted on the fish hepaci-like viruses under the assumption of virus-host co-divergence.

It is notable that two previously identified hepaci-like viruses, Hepacivirus sp., identified in a Red-eared slider turtle (*Trachemys scripta elegans*) and Wenling moray eel hepacivirus, identified from a moray eel (*Gymnothorax reticularis*), fall basal to all known pegi-like viruses, even though other fish and turtle viruses group with the hepaci-like viruses. To investigate this issue in more detail we performed an additional phylogenetic analysis of amino acid sequences of the NS2/3 and NS5 regions separately (Fig. 4). While the phylogenetic position of the Red-eared slider turtle virus remains unchanged, the moray eel virus fell in markedly different positions in these two phylogenies, falling closer to the other fish hepaci-like viruses in the NS2/3 phylogeny (as expected with virus-host co-divergence) but on an anomalously long branch on the NS5 phylogeny (and would constitute an outgroup sequence if the tree were mid-point rooted). Such a change in phylogenetic position means that the position of the moray eel virus as a sister-group to the pegiviruses in the polyprotein phylogeny is likely artifactual and could plausibly have resulted from recombination (see below) or extreme rate variation. While a number of other clades change position between the NS2/3 and NS5 phylogenies, the deeper nodes on both trees receive only weak bootstrap support such that these movements may simply reflect a lack of phylogenetic resolution.

We utilised the RDP program on complete genome alignments to investigate the potential occurrence of recombination in more detail. Accordingly, we identified two putative recombinant sequences— BbHV (minimum P-value = 0.04) and Hepacivirus F (minimum P-value = 5.30 E−03) —although the putative recombinant region in the latter was extremely short (Table 3). To confirm these events, we estimated phylogenetic trees either side of the putative recombination breakpoints. Critically, although there was some topological movement, none were consistently supported by significant (i.e. >70%) bootstrap support values. Hence, these data provide no conclusive evidence for the occurrence of recombination in the sequences analysed.

**Table 3.** RDP results from the hepacivirus and pegivirus recombination detection analysis.

Putative recombinant sequence	Start	End	Major parental sequence(s)	Minor parental sequence(s)	Programs with positive signal
BbHV	1039	5346	NC_009823.1 hepatitis C virus genotype 2	NC_001655.1 Hepatitis GB virus B	RDP, Bootscan, Maxchi, SiScan, LARD
NC_038427.1 Hepacivirus F	1	74	NC_021153.1 rodent hepacivirus	KC411796.1 rodent hepacivirus	RDP, GENECONV, Bootscan, 3Seq

### 3.5 Virus-host co-divergence

The existence of a number of taxon-specific clades (e.g. avian-specific hepaci-like viruses) is compatible with virus-host co-divergence, particularly if the hepaci- and pegi-like virus phylogeny is rooted (as here) using the fish-associated hepacivirus that represent the most phylogenetically divergent host taxa (Shi et al. 2018) (Fig. 3). For example, the lungfish hepacivirus falls as a sister-group to the tetrapod hepaciviruses as expected under virus-host co-divergence, and two turtle-infecting hepaciviruses fall as the sister-group to the mammalian hepaciviruses, although it is notable that two rodent hepaciviruses cluster anomalously with them.

**Figure 5.**
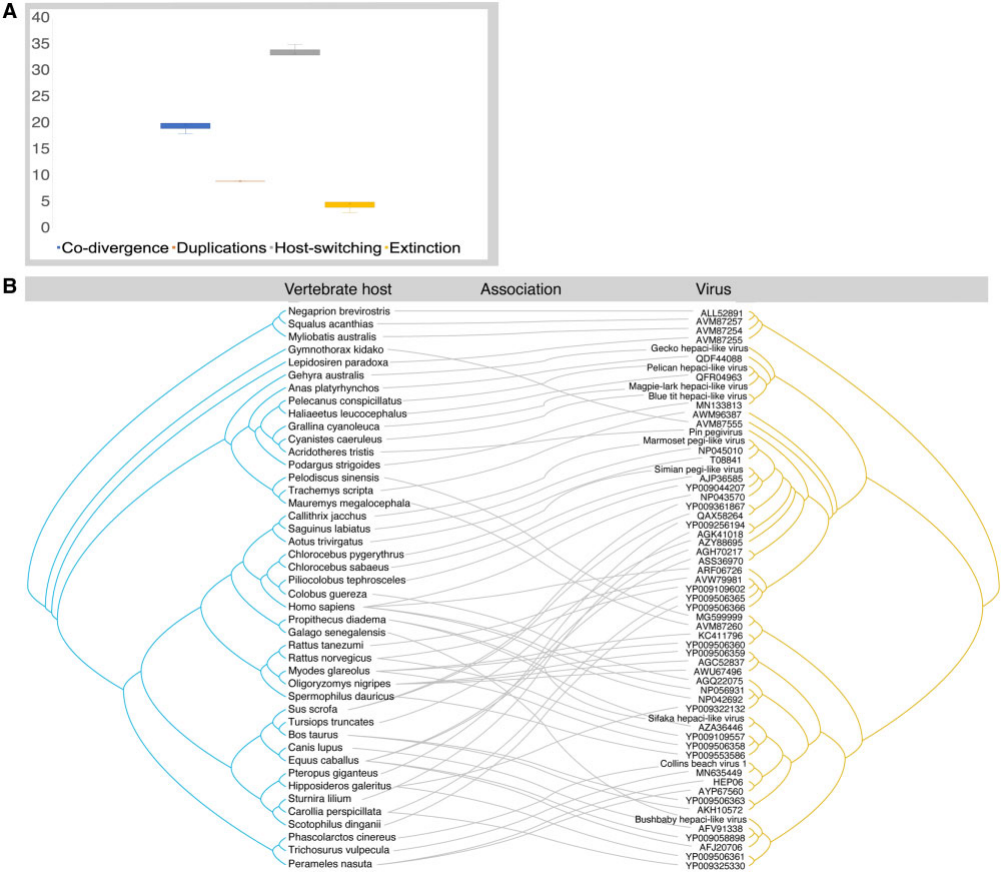
Co-phylogenetic analysis of hepaci- and pegi-like viruses. (A) Relative frequency of co-phylogenetic events shown as boxplots: co-divergence (blue), duplications (orange), host-switching (gray), and extinction (yellow). The median is represented as the cross, upper, and lower quartiles (box limits) and the upper and lower values (whiskers). The ‘event costs’ used in this analysis were 0 for co-divergence, 1 for duplication, 1 for host-switching, and 1 for extinction. (B) Tanglegram displaying the possible evolutionary connections between host species (left, tree in blue) and hepaci- and pegi-like virus (right, tree in yellow).

To explore possible co-divergence in the hepaci- and pegi-like viruses in more detail, we estimated the relative frequency of four evolutionary ‘events’: co-divergence, duplications, host-switching and extinction. Although this suggested that host-switching (i.e. cross-species virus transmission) was the most common co-phylogenetic process (33–35 estimated events), there was clearly also evidence for relatively commonplace virus-host co-divergence (18–20 events; Fig. 5A). A similar pattern is apparent from the tanglegram connecting the virus and host phylogenies (Fig. 5B).

## 4. Discussion

Using total RNA sequencing we identified four novel hepaci-like viruses and a single variant of a previously described hepacivirus in native Australian marsupials, birds, and reptiles. In addition, through SRA-mining, we were able to discover three novel hepaci-like viruses and two novel pegi-like viruses, the latter in primate hosts. Accordingly, this work broadens our knowledge of two viral genera within the family *Flaviviridae* and suggests that, with increased sampling, additional hepaci-like and pegi-like viruses will likely be identified in many other animal hosts.

Hepaciviruses encode a single polyprotein that is commonly observed among vertebrate members of the *Flaviviridae*. The hepaci-like viruses identified in this study were characterised by high conservation in the NS5B, RdRp, and NS3 genes. Other proteins were present in most of the viral genomes identified, although the NS5A gene was not readily identified in the avian and GHVs, while the two envelope genes could not be identified in the GHV. Given that the complete encoding region of the polyprotein is likely present, we anticipate that these genes are present in these viruses but are highly divergent in sequence and hence cannot be easily be identified using approaches that only assess primary sequence homology.

As hepaci- and pegi-like viruses are commonly thought to have co-diverged with their hosts over time-scales of many millions of years, we expected that these viruses would follow a phylogenetic pattern that is generally similar to that of their hosts (Shi et al. 2016a). Indeed, although the phylogenetic analysis performed here reveals frequent host-switching, which is of course associated with disease emergence, this seemingly occurs on an evolutionary backbone of long-term virus-host co-divergence (Geoghegan, Duchêne, and Holmes 2017; Shi et al. 2018). Indeed, despite abundant cross-species transmission, it is striking that the avian, reptile, mammal, and marsupial viruses generally fall into taxon-specific clades. Similarly, the primate pegi-like viruses, including those newly described here, can be placed into Old World and New World primate clades in a pattern that generally mirrors the host phylogeny.

There are, however, some notable exceptions to co-divergence, such as rodent hepacivirus and Hepacivirus J, identified in bank voles (*Myodes glareolus*) from Germany (Drexler et al. 2013), that cluster with turtle-associated hepaci-like viruses. Similarly, as noted above, it is striking that two previously identified hepaci-like viruses, Hepacivirus sp., identified in a Red-eared slider turtle and Wenling moray eel hepacivirus fall basal to all known pegi-like viruses, even though other fish and turtle sequences group with the hepaci-like viruses. Similarly, rather than falling as a sister-group to those hepaci-like viruses sampled from placental mammals, it is notable that the marsupial hepaci-like viruses fall within the placental mammal phylogenetic diversity. Such a phylogenetic pattern clearly highlights the complexity of the current division into *Hepacivirus* and *Pegivirus* genera and strongly suggests that a re-classification of these viruses is required.

The avian hepaci-like virus clade characterised here comprises BtHV, recovered from the SRA, as well the novel Australian pelican and MaHVs, three variants of DuHV (Chu et al. 2019), and the recently described Jogalong virus, identified from a *Culex annulirostris* mosquito in northern Western Australia. However, the mosquito from which Jogalong virus was identified is hypothesised to have recently fed upon a tawny frogmouth (*Podargus strigoides*) (Williams et al. 2020). Similarly, a distinct clade of GHVs was identified, containing the novel GHV from an Australian gecko (*G. lauta*), and the two previously identified GHVs from China. The gecko clade itself clusters with the avian hepaciviruses, which in turn groups with the pegi-like viruses. Importantly, however, there is relatively weak support for the nodes in question, and if this reptile–avian cluster grouped with the hepaci-like viruses then the overall phylogeny would offer better support to virus-host co-divergence.

The novel KHV and CBV1 (identified in ticks feeding on a long-nosed bandicoot) group closely together, along with the single marsupial-infecting hepaci-like virus identified previously, Brushtail possum hepacivirus (Chang et al. 2019). Together, these viruses establish a distinct marsupial clade of hepaci-like viruses. Both Collins beach virus and CBV1 also fall in this clade and we suggest that they represent variants of the same virus. Interestingly, two marsupial-associated gammaherpesviruses (double-strand DNA viruses) from Australian koalas (*P. cinereus*) and wombats (*Vombatus ursinus*), similarly formed a distinct clade (Vaz et al. 2018). Hence, marsupial-specific clades may be present in other vertebrate-infecting viruses. In addition, two primate hepaci-like viruses were identified from the SRA, including a novel variant of SfHV closely related to the previously identified SfHV, both of which were isolated from Diademed sifakas. In contrast, BbHV isolated from a Senegal bushbaby, falls as a sister-group to a cluster of equine and CHVs that are in turn closely related to HCV. Despite the obvious clinical importance of HCV, its ultimate animal reservoir is unknown (Pybus and Gray 2013; Lyons et al. 2014; Thézé et al. 2015; Hartlage, Cullen, and Kapoor 2016). The placement of another primate (i.e. bushbaby) virus tentatively suggests that there are additional primate viruses within this group that are yet to be discovered and that might shed light on the origin of HCV.

Arthropod vectors are known to transmit a wide variety of infectious disease agents, including members of the *Flaviviridae*, and are responsible for more than 17 per cent of total infectious diseases and cause upwards of 700,000 deaths per year (World Health Organisation 2017). All the vector-borne flaviviruses described to date are members of the genus *Flavivirus*, with no evidence of vector-borne transmission within the hepaci- or pegi-like viruses. Although the data presented here and previously (Harvey et al. 2019a) tentatively suggests that *I.holocyclus* ticks may act as a vector of Collins beach virus, the biology of ticks prevents us from clearly establishing this link. In particular, as the ticks sampled were engorged adult females it is impossible to determine if Collins beach virus infected the tick itself or was merely held within their considerable blood meal. As ticks take only one blood meal in each lifecycle stage (of which adulthood is the final stage) they do not act as ‘biological syringes’ as other arthropods such as mosquitos and biting flies do (Nuttall et al. 1994). As both Collins beach virus and CBV1 were detected in very low abundance it seems unlikely that this virus is actively infecting the tick itself. Future meta-transcriptomic studies on unfed questing ticks, as well as experimental transmission studies, are therefore a priority.

## Supplementary Material

veaa064_Supplementary_Data
