# Supplementary material for: Novel hepaci- and pegi-like viruses in native Australian wildlife and non-human primates
Source: Virus Evol. 2020 Aug 20;6(2):veaa064. doi: 10.1093/ve/veaa064 (PMC7673076; doi:10.1093/ve/veaa064)
Supplement: veaa064_Supplementary_Data [file veaa064_supplementary_data.zip › Porter.Supplementary Table 1.Revised.docx]

**Supplementary Table 1.** Results of the SRA mining of marsupial transcriptomes.

| **Accession** | **Organism** | **Tissue** |
| --- | --- | --- |
| DRX012229 | *Notamacropus eugenii* | Liver |
| DRX012230 | *Notamacropus eugenii* | Lung |
| DRX012231 | *Notamacropus eugenii* | Heart |
| DRX012232 | *Notamacropus eugenii* | Spleen |
| DRX012233 | *Notamacropus eugenii* | Adrenal glands |
| DRX012234 | *Notamacropus eugenii* | Mammary glands |
| DRX012235 | *Notamacropus eugenii* | Follicle ovary |
| DRX012236 | *Notamacropus eugenii* | Ovary |
| DRX012237 | *Notamacropus eugenii* | Retroperitoneal white adipose tissue |
| DRX012238 | *Notamacropus eugenii* | Gravid endometrium |
| DRX012239 | *Notamacropus eugenii* | Non-gravid endometrium |
| DRX012240 | *Notamacropus eugenii* | Gravid endometrium |
| DRX012241 | *Notamacropus eugenii* | Non-gravid endometrium |
| DRX012242 | *Notamacropus eugenii* | Mid-prostate |
| DRX012243 | *Notamacropus eugenii* | Testis |
| DRX012244 | *Notamacropus eugenii* | Caudal epididymus |
| DRX012245 | *Notamacropus eugenii* | Male phallus |
| DRX012246 | *Notamacropus eugenii* | Male phallus |
| DRX012247 | *Notamacropus eugenii* | Endo |
| DRX012248 | *Notamacropus eugenii* | Liver |
| DRX012249 | *Notamacropus eugenii* | Lung |
| DRX012250 | *Notamacropus eugenii* | Heart |
| DRX012251 | *Notamacropus eugenii* | Spleen |
| DRX012252 | *Notamacropus eugenii* | Adrenal glands |
| DRX012253 | *Notamacropus eugenii* | Mammary glands |
| DRX012254 | *Notamacropus eugenii* | Follicle ovary |
| DRX012255 | *Notamacropus eugenii* | Ovary |
| DRX012256 | *Notamacropus eugenii* | Retroperitoneal white adipose tissue |
| DRX012257 | *Notamacropus eugenii* | Gravid endometrium |
| DRX012258 | *Notamacropus eugenii* | Non-gravid endometrium |
| DRX012259 | *Notamacropus eugenii* | Gravid endometrium |
| DRX012260 | *Notamacropus eugenii* | Non-gravid endometrium |
| DRX012261 | *Notamacropus eugenii* | Mid-prostate |
| DRX012262 | *Notamacropus eugenii* | Testis |
| DRX012263 | *Notamacropus eugenii* | Caudal epididymus |
| DRX012264 | *Notamacropus eugenii* | Male phallus |
| DRX012265 | *Notamacropus eugenii* | Male phallus |
| DRX012266 | *Notamacropus eugenii* | Endo |
| ERX016329 | *Monodelphis domestica* | Liver |
| ERX016333 | *Monodelphis domestica* | Liver |
| ERX016352 | *Monodelphis domestica* | Liver |
| ERX144552 | *Monodelphis domestica* | Testis |
| ERX144554 | *Monodelphis domestica* | Testis |
| ERX640064 | *Sarcophilus harrisii* | Lymph node |
| ERX640065 | *Sarcophilus harrisii* | Spleen |
| SRX000121 | *Tachyglossus aculeatus* | Unknown |
| SRX000122 | *Tachyglossus aculeatus* | Unknown |
| SRX000186 | *Ornithorhynchus anatinus* | Unknown |
| SRX010966 | *Sarcophilus harrisii* | Facial tumor |
| SRX010967 | *Sarcophilus harrisii* | Facial tumor |
| SRX015780 | *Sarcophilus harrisii* | Facial tumor |
| SRX015781 | *Sarcophilus harrisii* | Kidney |
| SRX015782 | *Sarcophilus harrisii* | Brain |
| SRX015783 | *Sarcophilus harrisii* | Heart |
| SRX015784 | *Sarcophilus harrisii* | Liver |
| SRX015785 | *Sarcophilus harrisii* | Bone marrow |
| SRX015786 | *Sarcophilus harrisii* | Pancreas |
| SRX015787 | *Sarcophilus harrisii* | Skin |
| SRX015788 | *Sarcophilus harrisii* | Spleen |
| SRX015789 | *Sarcophilus harrisii* | Facial tumor |
| SRX015790 | *Sarcophilus harrisii* | Facial tumor |
| SRX015791 | *Sarcophilus harrisii* | Facial tumor |
| SRX015792 | *Sarcophilus harrisii* | Facial tumor |
| SRX015793 | *Sarcophilus harrisii* | Testis |
| SRX015794 | *Sarcophilus harrisii* | Lymph node |
| SRX019249 | *Notamacropus eugenii* | Unknown |
| SRX019250 | *Notamacropus eugenii* | Unknown |
| SRX026473 | *Ornithorhynchus anatinus* | Unknown |
| SRX028596 | *Ornithorhynchus anatinus* | Testis |
| SRX028597 | *Ornithorhynchus anatinus* | Testis |
| SRX028598 | *Tachyglossus aculeatus* | Testis |
| SRX081708 | *Notamacropus eugenii* | Pouch young cell |
| SRX081709 | *Notamacropus eugenii* | Pouch young testis |
| SRX081710 | *Notamacropus eugenii* | Pouch young brain |
| SRX081711 | *Notamacropus eugenii* | Pouch young fibroblasts |
| SRX081712 | *Notamacropus eugenii* | Pouch young liver |
| SRX081713 | *Notamacropus eugenii* | Pouch young ovary |
| SRX081714 | *Notamacropus eugenii* | Pouch young testis |
| SRX081715 | *Notamacropus eugenii* | Pouch young testis |
| SRX081881 | *Ornithorhynchus anatinus* | Brain |
| SRX081882 | *Ornithorhynchus anatinus* | Brain |
| SRX081883 | *Ornithorhynchus anatinus* | Cerebellum |
| SRX081884 | *Ornithorhynchus anatinus* | Cerebellum |
| SRX081885 | *Ornithorhynchus anatinus* | Heart |
| SRX081886 | *Ornithorhynchus anatinus* | Heart |
| SRX081887 | *Ornithorhynchus anatinus* | Kidney |
| SRX081888 | *Ornithorhynchus anatinus* | Kidney |
| SRX081889 | *Ornithorhynchus anatinus* | Liver |
| SRX081890 | *Ornithorhynchus anatinus* | Liver |
| SRX081891 | *Ornithorhynchus anatinus* | Testis |
| SRX081892 | *Ornithorhynchus anatinus* | Testis |
| SRX081893 | *Monodelphis domestica* | Brain |
| SRX081894 | *Monodelphis domestica* | Brain |
| SRX081895 | *Monodelphis domestica* | Cerebellum |
| SRX081896 | *Monodelphis domestica* | Cerebellum |
| SRX081897 | *Monodelphis domestica* | Heart |
| SRX081898 | *Monodelphis domestica* | Heart |
| SRX081899 | *Monodelphis domestica* | Kidney |
| SRX081900 | *Monodelphis domestica* | Kidney |
| SRX081901 | *Monodelphis domestica* | Liver |
| SRX081902 | *Monodelphis domestica* | Liver |
| SRX081903 | *Monodelphis domestica* | Testis |
| SRX081904 | *Monodelphis domestica* | Testis |
| SRX100226 | *Ornithorhynchus anatinus* | Unknown |
| SRX100227 | *Ornithorhynchus anatinus* | Unknown |
| SRX100327 | *Ornithorhynchus anatinus* | Unknown |
| SRX100328 | *Ornithorhynchus anatinus* | Unknown |
| SRX100329 | *Ornithorhynchus anatinus* | Unknown |
| SRX104355 | *Monodelphis domestica* | Unknown |
| SRX104358 | *Monodelphis domestica* | Unknown |
| SRX104783 | *Monodelphis domestica* | Unknown |
| SRX104787 | *Monodelphis domestica* | Unknown |
| SRX1121057 | *Monodelphis domestica* | Fore limb |
| SRX1121058 | *Monodelphis domestica* | Fore limb |
| SRX1121059 | *Monodelphis domestica* | Fore limb |
| SRX1121060 | *Monodelphis domestica* | Fore limb |
| SRX1121061 | *Monodelphis domestica* | Fore limb |
| SRX1121062 | *Monodelphis domestica* | Fore limb |
| SRX1121063 | *Monodelphis domestica* | Fore limb |
| SRX1121064 | *Monodelphis domestica* | Fore limb |
| SRX1121065 | *Monodelphis domestica* | Fore limb |
| SRX122683 | *Ornithorhynchus anatinus* | Ovary |
| SRX122684 | *Ornithorhynchus anatinus* | Fibroblast |
| SRX122685 | *Ornithorhynchus anatinus* | Fibroblast |
| SRX132007 | *Monodelphis domestica* | Brain |
| SRX132008 | *Monodelphis domestica* | Brain |
| SRX132009 | *Monodelphis domestica* | Liver |
| SRX132010 | *Monodelphis domestica* | Liver |
| SRX1438951 | *Monodelphis domestica* | Testis |
| SRX1439067 | *Monodelphis domestica* | Testis |
| SRX1458693 | *Monodelphis domestica* | Endometrium |
| SRX1458770 | *Monodelphis domestica* | Endometrium myometrium |
| SRX1459674 | *Monodelphis domestica* | Endometrium |
| SRX1462098 | *Monodelphis domestica* | Uterus |
| SRX1462099 | *Monodelphis domestica* | Uterus |
| SRX1462170 | *Monodelphis domestica* | Uterus |
| SRX1462218 | *Monodelphis domestica* | Uterus |
| SRX1462219 | *Monodelphis domestica* | Uterus |
| SRX1462220 | *Monodelphis domestica* | Uterus |
| SRX149626 | *Monodelphis domestica* | Unknown |
| SRX149627 | *Monodelphis domestica* | Unknown |
| SRX149628 | *Monodelphis domestica* | Unknown |
| SRX149629 | *Monodelphis domestica* | Unknown |
| SRX149630 | *Monodelphis domestica* | Unknown |
| SRX149631 | *Monodelphis domestica* | Unknown |
| SRX149632 | *Monodelphis domestica* | Unknown |
| SRX149633 | *Monodelphis domestica* | Unknown |
| SRX149634 | *Monodelphis domestica* | Unknown |
| SRX149635 | *Monodelphis domestica* | Unknown |
| SRX1629207 | *Monodelphis domestica* | Placenta |
| SRX182793 | *Monodelphis domestica* | Brain |
| SRX182794 | *Monodelphis domestica* | Cerebellum |
| SRX182795 | *Monodelphis domestica* | Heart |
| SRX182796 | *Monodelphis domestica* | Kidney |
| SRX182797 | *Monodelphis domestica* | Testis |
| SRX182798 | *Ornithorhynchus anatinus* | Brain |
| SRX182799 | *Ornithorhynchus anatinus* | Cerebellum |
| SRX182800 | *Ornithorhynchus anatinus* | Heart |
| SRX182801 | *Ornithorhynchus anatinus* | Kidney |
| SRX182802 | *Ornithorhynchus anatinus* | Testis |
| SRX182814 | *Didelphis virginiana* | Brain |
| SRX182815 | *Didelphis virginiana* | Kidney |
| SRX1881580 | *Phascolarctos cinereus* | Mammary |
| SRX1944674 | *Pseudantechinus macdonnellensis* | Liver |
| SRX1944675 | *Isoodon obesulus* | Liver |
| SRX1944676 | *Dactylopsila trivirgata* | Liver |
| SRX1944678 | *Pseudochirops cupreus* | Liver |
| SRX1944679 | *Petrogale xanthopus* | Liver |
| SRX1944680 | *Cercartetus concinnus* | Liver |
| SRX2034651 | *Monodelphis domestica* | Cervix |
| SRX2034652 | *Monodelphis domestica* | Cervix |
| SRX2034653 | *Monodelphis domestica* | Cervix |
| SRX2034654 | *Monodelphis domestica* | Cervix |
| SRX2034655 | *Monodelphis domestica* | Cervix |
| SRX2034656 | *Monodelphis domestica* | Cervix |
| SRX2034657 | *Monodelphis domestica* | Cervix |
| SRX2034658 | *Monodelphis domestica* | Cervix |
| SRX211661 | *Petaurus breviceps* | Kidney |
| SRX211662 | *Petaurus breviceps* | Kidney |
| SRX211706 | *Petaurus breviceps* | Brain |
| SRX211707 | *Petaurus breviceps* | Brain |
| SRX217694 | *Monodelphis domestica* | Brain |
| SRX217695 | *Monodelphis domestica* | Ovary |
| SRX217696 | *Monodelphis domestica* | Placenta |
| SRX217697 | *Monodelphis domestica* | Testis |
| SRX217698 | *Ornithorhynchus anatinus* | Brain |
| SRX217699 | *Ornithorhynchus anatinus* | Ovary |
| SRX217700 | *Ornithorhynchus anatinus* | Testis |
| SRX2310349 | *Sarcophilus harrisii* | Blood |
| SRX2310350 | *Sarcophilus harrisii* | Blood |
| SRX2310351 | *Sarcophilus harrisii* | Blood |
| SRX2310352 | *Sarcophilus harrisii* | Blood |
| SRX2310353 | *Sarcophilus harrisii* | Blood |
| SRX2310354 | *Sarcophilus harrisii* | Blood |
| SRX2310355 | *Sarcophilus harrisii* | Blood |
| SRX2310356 | *Sarcophilus harrisii* | Blood |
| SRX2310357 | *Sarcophilus harrisii* | Blood |
| SRX2310358 | *Sarcophilus harrisii* | Blood |
| SRX2310359 | *Sarcophilus harrisii* | Blood |
| SRX2310360 | *Sarcophilus harrisii* | Blood |
| SRX2310361 | *Sarcophilus harrisii* | Blood |
| SRX2310362 | *Sarcophilus harrisii* | Blood |
| SRX2310363 | *Sarcophilus harrisii* | Blood |
| SRX2310364 | *Sarcophilus harrisii* | Blood |
| SRX2310365 | *Sarcophilus harrisii* | Blood |
| SRX2310366 | *Sarcophilus harrisii* | Blood |
| SRX2310367 | *Sarcophilus harrisii* | Blood |
| SRX2310368 | *Sarcophilus harrisii* | Blood |
| SRX2310369 | *Sarcophilus harrisii* | Blood |
| SRX2310370 | *Sarcophilus harrisii* | Blood |
| SRX2310371 | *Sarcophilus harrisii* | Blood |
| SRX2310372 | *Sarcophilus harrisii* | Blood |
| SRX2310373 | *Sarcophilus harrisii* | Blood |
| SRX2310374 | *Sarcophilus harrisii* | Blood |
| SRX2310375 | *Sarcophilus harrisii* | Blood |
| SRX2310376 | *Sarcophilus harrisii* | Blood |
| SRX2310377 | *Sarcophilus harrisii* | Blood |
| SRX2310378 | *Sarcophilus harrisii* | Blood |
| SRX2310385 | *Sarcophilus harrisii* | Blood |
| SRX2310386 | *Sarcophilus harrisii* | Blood |
| SRX250119 | *Monodelphis domestica* | Extra-embryonic membranes |
| SRX250120 | *Monodelphis domestica* | Extra-embryonic membranes |
| SRX250121 | *Monodelphis domestica* | Extra-embryonic membranes |
| SRX250122 | *Monodelphis domestica* | Extra-embryonic membranes |
| SRX250123 | *Monodelphis domestica* | Fetal brain |
| SRX250124 | *Monodelphis domestica* | Fetal brain |
| SRX250125 | *Monodelphis domestica* | Fetal brain |
| SRX250126 | *Monodelphis domestica* | Fetal brain |
| SRX250127 | *Monodelphis domestica* | Extra-embryonic membranes |
| SRX250128 | *Monodelphis domestica* | Extra-embryonic membranes |
| SRX250129 | *Monodelphis domestica* | Extra-embryonic membranes |
| SRX250130 | *Monodelphis domestica* | Extra-embryonic membranes |
| SRX250131 | *Monodelphis domestica* | Fetal brain |
| SRX250132 | *Monodelphis domestica* | Fetal brain |
| SRX250133 | *Monodelphis domestica* | Fetal brain |
| SRX250134 | *Monodelphis domestica* | Fetal brain |
| SRX2532330 | *Monodelphis domestica* | Meckel's cartilage and anterior malleus |
| SRX2532331 | *Monodelphis domestica* | Meckel's cartilage and anterior malleus |
| SRX2532332 | *Monodelphis domestica* | Meckel's cartilage and anterior malleus |
| SRX2532333 | *Monodelphis domestica* | Meckel's cartilage and anterior malleus |
| SRX2532334 | *Monodelphis domestica* | Meckel's cartilage and anterior malleus |
| SRX2532335 | *Monodelphis domestica* | Meckel's cartilage and anterior malleus |
| SRX2532336 | *Monodelphis domestica* | Meckel's cartilage and anterior malleus |
| SRX2532337 | *Monodelphis domestica* | Meckel's cartilage and anterior malleus |
| SRX2532338 | *Monodelphis domestica* | Meckel's cartilage and anterior malleus |
| SRX2711970 | *Sarcophilus harrisii* | PBMC |
| SRX2711971 | *Sarcophilus harrisii* | PBMC |
| SRX286481 | *Monodelphis domestica* | Unknown |
| SRX290643 | *Monodelphis domestica* | Unknown |
| SRX290644 | *Monodelphis domestica* | Unknown |
| SRX290645 | *Monodelphis domestica* | Unknown |
| SRX290646 | *Monodelphis domestica* | Unknown |
| SRX290647 | *Monodelphis domestica* | Unknown |
| SRX290648 | *Monodelphis domestica* | Unknown |
| SRX2999980 | *Monodelphis domestica* | Uterus |
| SRX2999981 | *Monodelphis domestica* | Uterus |
| SRX2999982 | *Monodelphis domestica* | Uterus |
| SRX2999983 | *Monodelphis domestica* | Uterus |
| SRX2999984 | *Monodelphis domestica* | Uterus |
| SRX2999985 | *Monodelphis domestica* | Uterus |
| SRX2999986 | *Monodelphis domestica* | Uterus |
| SRX2999987 | *Monodelphis domestica* | Uterus |
| SRX309998 | *Monodelphis domestica* | Unknown |
| SRX309999 | *Monodelphis domestica* | Unknown |
| SRX310000 | *Monodelphis domestica* | Unknown |
| SRX310001 | *Monodelphis domestica* | Unknown |
| SRX310002 | *Monodelphis domestica* | Unknown |
| SRX310003 | *Monodelphis domestica* | Unknown |
| SRX310004 | *Monodelphis domestica* | Unknown |
| SRX310005 | *Monodelphis domestica* | Unknown |
| SRX310006 | *Monodelphis domestica* | Unknown |
| SRX310007 | *Monodelphis domestica* | Unknown |
| SRX310008 | *Monodelphis domestica* | Unknown |
| SRX310009 | *Monodelphis domestica* | Unknown |
| SRX310011 | *Monodelphis domestica* | Unknown |
| SRX312217 | *Monodelphis domestica* | Unknown |
| SRX312219 | *Monodelphis domestica* | Unknown |
| SRX312220 | *Monodelphis domestica* | Unknown |
| SRX312221 | *Monodelphis domestica* | Unknown |
| SRX312222 | *Monodelphis domestica* | Unknown |
| SRX312223 | *Monodelphis domestica* | Unknown |
| SRX312226 | *Monodelphis domestica* | Unknown |
| SRX319665 | *Tachyglossus aculeatus* | Unknown |
| SRX328081 | *Monodelphis domestica* | Ovary |
| SRX328082 | *Monodelphis domestica* | Placenta |
| SRX328083 | *Monodelphis domestica* | Placenta |
| SRX328084 | *Ornithorhynchus anatinus* | Ovary |
| SRX328085 | *Ornithorhynchus anatinus* | Ovary |
| SRX386112 | *Notamacropus eugenii* | Liver |
| SRX386113 | *Notamacropus eugenii* | Testis |
| SRX389450 | *Phascolarctos cinereus* | Unknown |
| SRX467707 | *Monodelphis domestica* | Spinal cord |
| SRX467708 | *Monodelphis domestica* | Spinal cord |
| SRX467709 | *Monodelphis domestica* | Spinal cord |
| SRX467710 | *Monodelphis domestica* | Spinal cord |
| SRX467711 | *Monodelphis domestica* | Spinal cord |
| SRX467712 | *Monodelphis domestica* | Spinal cord |
| SRX467713 | *Monodelphis domestica* | Spinal cord |
| SRX500280 | *Phascolarctos cinereus* | Spleen |
| SRX501262 | *Phascolarctos cinereus* | Liver |
| SRX501302 | *Phascolarctos cinereus* | Uterus |
| SRX501314 | *Phascolarctos cinereus* | Kidney |
| SRX501320 | *Phascolarctos cinereus* | Lung |
| SRX501342 | *Phascolarctos cinereus* | Heart |
| SRX501343 | *Phascolarctos cinereus* | Brain |
| SRX501344 | *Phascolarctos cinereus* | Adrenal |
| SRX544838 | *Ornithorhynchus anatinus* | Pregnant endometrium |
| SRX641589 | *Notamacropus eugenii* | Milk |
| SRX641590 | *Notamacropus eugenii* | Milk |
| SRX641591 | *Notamacropus eugenii* | Milk |
| SRX641592 | *Notamacropus eugenii* | Milk |
| SRX641593 | *Notamacropus eugenii* | Milk |
| SRX641594 | *Notamacropus eugenii* | Serum |
| SRX641595 | *Notamacropus eugenii* | Serum |
| SRX681637 | *Notamacropus eugenii* | Blood |
| SRX681735 | *Notamacropus eugenii* | Blood |
| SRX681743 | *Notamacropus eugenii* | Liver |
| SRX862745 | *Sarcophilus harrisii* | Milk |
| SRX873004 | *Phascolarctos cinereus adustus* | Spleen |
| SRX873837 | *Phascolarctos cinereus adustus* | Buffy coat |
| SRX873905 | *Phascolarctos cinereus adustus* | Buffy coat |
| SRX876875 | *Phascolarctos cinereus adustus* | Spleen |
| SRX877075 | *Phascolarctos cinereus adustus* | Buffy coat |
| SRX877109 | *Phascolarctos cinereus adustus* | Buffy coat |
| SRX877987 | *Monodelphis domestica* | Endometrium |
| SRX913284 | *Potorous tridactylus* | Kidney |
| SRX982566 | *Monodelphis domestica* | Endometrial stromal fibroblasts |
| SRX982567 | *Monodelphis domestica* | Endometrial stromal fibroblasts |
| SRX997075 | *Monodelphis domestica* | Testis |
| SRX997079 | *Monodelphis domestica* | Testis |
