# Supplementary material for: Novel hepaci- and pegi-like viruses in native Australian wildlife and non-human primates
Source: Virus Evol. 2020 Aug 20;6(2):veaa064. doi: 10.1093/ve/veaa064 (PMC7673076; doi:10.1093/ve/veaa064)
Supplement: veaa064_Supplementary_Data [file veaa064_supplementary_data.zip › Porter.Supplementary Table 2.Revised.docx]

**Supplementary Table 2.** Abundance of hepaci-like virus reads in the total read count of the sequencing libraries used in this analysis.

| **Library** | **Raw reads** | | **Hepaci-like virus** | | **Host (rpl13a)** | |
| --- | --- | --- | --- | --- | --- | --- |
|  | *Pairs* | *Total* | *Reads* | *Abundance (%)* | *Reads* | *Abundance (%)* |
| Vert5 | 25,589,029 | 51,178,058 | 172 | 0.00034 | 8,642 | 0.01689 |
| Vert7 | 21,508,723 | 43,017,446 | 762 | 0.00177 | 6,651 | 0.01546 |
| Vert14 | 24,632,160 | 49,264,320 | 26 | 0.00005 | 3,095 | 0.00628 |
| Vert15 | 19,091,826 | 38,183,652 | 172 | 0.00045 | 5,140 | 0.01346 |
| Vert20 | 14,383,375 | 28,766,750 | 29,391 | 0.10217 | 3,205 | 0.01114 |
| Vert31 | 22,148,613 | 44,297,226 | 54,619 | 0.12330 | 794 | 0.00179 |
| Vert44 | 13,086,370 | 26,172,740 | 362 | 0.00138 | 2,978 | 0.01138 |
| Tick07 | 28,027,815 | 56,055,630 | 6 | 0.00001 | 19,752 | 0.03524 |
| Tick08 | 27,344,944 | 54,689,888 | 430 | 0.00079 | 33,706 | 0.06163 |
| Tick10 | 26,474,448 | 52,948,896 | 6 | 0.00001 | 6,274 | 0.01185 |
| Invert13 | 15,050,377 | 30,100,754 | 43 | 0.00014 | 12,207 | 0.04055 |
