# Supplementary material for: Novel hepaci- and pegi-like viruses in native Australian wildlife and non-human primates
Source: Virus Evol. 2020 Aug 20;6(2):veaa064. doi: 10.1093/ve/veaa064 (PMC7673076; doi:10.1093/ve/veaa064)
Supplement: veaa064_Supplementary_Data [file veaa064_supplementary_data.zip › Porter.Supplementary Table 3.Revised.docx]

**Supplementary Table 3.** Results of the SRA mining of avian transcriptomes (excluding *Gallus gallus*).

| **Organism** | **Accession** |
| --- | --- |
| *Acanthis flammea* | SRR1531383 |
| *Acanthis flammea* | SRR1531385 |
| *Acanthis flammea* | SRR1531381 |
| *Acanthis flammea* | SRR1531384 |
| *Acanthis flammea* | SRR1531382 |
| *Acanthis flammea* | SRR1531390 |
| *Acanthis flammea* | SRR1531388 |
| *Acanthis hornemanni* | SRR1531386 |
| *Acanthis hornemanni* | SRR1531387 |
| *Acanthis hornemanni* | SRR1531389 |
| *Accipiter virgatus* | SRR3203234 |
| *Aegypius monachus* | SRR1265955 |
| *Aegypius monachus* | SRR3203236 |
| *Aerodramus fuciphagus* | SRR4116906 |
| *Aerodramus fuciphagus* | SRR4116899 |
| *Aerodramus fuciphagus* | SRR4116905 |
| *Aerodramus fuciphagus* | SRR4116901 |
| *Aerodramus fuciphagus* | SRR4116902 |
| *Aerodramus fuciphagus* | SRR4428213 |
| *Aerodramus fuciphagus* | SRR4107069 |
| *Aerodramus fuciphagus* | SRR4107068 |
| *Aerodramus fuciphagus* | SRR4107074 |
| *Aerodramus fuciphagus* | SRR4428214 |
| *Aerodramus fuciphagus* | SRR4107073 |
| *Aerodramus fuciphagus* | SRR5196680 |
| *Aerodramus fuciphagus* | SRR4428212 |
| *Aerodramus fuciphagus* | SRR5196679 |
| *Aerodramus fuciphagus* | SRR4086939 |
| *Aerodramus fuciphagus* | SRR4084719 |
| *Aerodramus maximus* | SRR4116890 |
| *Aerodramus maximus* | SRR4116892 |
| *Aerodramus maximus* | SRR4116889 |
| *Aerodramus maximus* | SRR4116895 |
| *Aerodramus maximus* | SRR4107066 |
| *Aerodramus maximus* | SRR4107067 |
| *Alectoris rufa* | SRR1664672 |
| *Alectoris rufa* | SRR1664674 |
| *Alectoris rufa* | SRR1664677 |
| *Alectoris rufa* | SRR1664666 |
| *Alectoris rufa* | SRR1664678 |
| *Alectoris rufa* | SRR1664667 |
| *Alectoris rufa* | SRR1664675 |
| *Alectoris rufa* | SRR1664679 |
| *Alectoris rufa* | SRR2089995 |
| *Anas platyrhynchos* | ERR597454 |
| *Anas platyrhynchos* | ERR597442 |
| *Anas platyrhynchos* | ERR597430 |
| *Anas platyrhynchos* | ERR597439 |
| *Anas platyrhynchos* | ERR597438 |
| *Anas platyrhynchos* | ERR597463 |
| *Anas platyrhynchos* | ERR597450 |
| *Anas platyrhynchos* | ERR597453 |
| *Anas platyrhynchos* | ERR597436 |
| *Anas platyrhynchos* | ERR597443 |
| *Anas platyrhynchos* | ERR597456 |
| *Anas platyrhynchos* | ERR597434 |
| *Anas platyrhynchos* | ERR597452 |
| *Anas platyrhynchos* | ERR597433 |
| *Anas platyrhynchos* | ERR597457 |
| *Anas platyrhynchos* | ERR707019 |
| *Anas platyrhynchos* | ERR597429 |
| *Anas platyrhynchos* | ERR597446 |
| *Anas platyrhynchos* | SRR345227 |
| *Anas platyrhynchos* | ERR597448 |
| *Anas platyrhynchos* | ERR597449 |
| *Anas platyrhynchos* | ERR597461 |
| *Anas platyrhynchos* | ERR597445 |
| *Anas platyrhynchos* | ERR597455 |
| *Anas platyrhynchos* | ERR597441 |
| *Anas platyrhynchos* | ERR597444 |
| *Anas platyrhynchos* | ERR597447 |
| *Anas platyrhynchos* | ERR597458 |
| *Anas platyrhynchos* | ERR597459 |
| *Anas platyrhynchos* | ERR597435 |
| *Anas platyrhynchos* | ERR597464 |
| *Anas platyrhynchos* | ERR597431 |
| *Anas platyrhynchos* | ERR707020 |
| *Anas platyrhynchos* | ERR707022 |
| *Anas platyrhynchos* | ERR597462 |
| *Anas platyrhynchos* | ERR707021 |
| *Anas platyrhynchos* | ERR707024 |
| *Anas platyrhynchos* | ERR597437 |
| *Anas platyrhynchos* | SRR2042815 |
| *Anas platyrhynchos* | ERR597460 |
| *Anas platyrhynchos* | SRR2042817 |
| *Anas platyrhynchos* | ERR597451 |
| *Anas platyrhynchos* | ERR597432 |
| *Anas platyrhynchos* | SRR345226 |
| *Anas platyrhynchos* | SRR954946 |
| *Anas platyrhynchos* | SRR2042818 |
| *Anas platyrhynchos* | SRR2042816 |
| *Anas platyrhynchos* | SRR954945 |
| *Anas platyrhynchos* | ERR597440 |
| *Anas platyrhynchos* | SRR345228 |
| *Anas platyrhynchos* | SRR954947 |
| *Anas platyrhynchos* | SRR2042814 |
| *Anas platyrhynchos* | SRR345229 |
| *Anas platyrhynchos* | SRR1786969 |
| *Anas platyrhynchos* | SRR1786971 |
| *Anas platyrhynchos* | SRR1786970 |
| *Anas platyrhynchos* | SRR064714 |
| *Anas platyrhynchos* | SRR1910464 |
| *Anas platyrhynchos* | SRR1786968 |
| *Anas platyrhynchos* | SRR3097717 |
| *Anas platyrhynchos* | SRR064718 |
| *Anas platyrhynchos* | SRR2321391 |
| *Anas platyrhynchos* | SRR1910465 |
| *Anas platyrhynchos* | SRR3097718 |
| *Anas platyrhynchos* | SRR3097716 |
| *Anas platyrhynchos* | SRR3097715 |
| *Anas platyrhynchos* | SRR3097711 |
| *Anas platyrhynchos* | SRR3097714 |
| *Anas platyrhynchos* | SRR3097713 |
| *Anas platyrhynchos* | SRR2321393 |
| *Anas platyrhynchos* | SRR2321395 |
| *Anas platyrhynchos* | SRR3097712 |
| *Anas platyrhynchos* | SRR2321394 |
| *Anas platyrhynchos* | SRR1910463 |
| *Anas platyrhynchos* | SRR2321390 |
| *Anas platyrhynchos* | SRR064716 |
| *Anas platyrhynchos* | SRR1051527 |
| *Anas platyrhynchos* | SRR064720 |
| *Anas platyrhynchos* | SRR2321392 |
| *Anas platyrhynchos* | SRR1055250 |
| *Anas platyrhynchos* | SRR3018355 |
| *Anas platyrhynchos* | SRR1295556 |
| *Anas platyrhynchos* | SRR5204377 |
| *Anas platyrhynchos* | SRR5204380 |
| *Anas platyrhynchos* | SRR1796037 |
| *Anas platyrhynchos* | SRR1055274 |
| *Anas platyrhynchos* | SRR5204378 |
| *Anas platyrhynchos* | SRR1055276 |
| *Anas platyrhynchos* | SRR5204376 |
| *Anas platyrhynchos* | SRR5204379 |
| *Anas platyrhynchos* | SRR1796026 |
| *Anas platyrhynchos* | SRR1796032 |
| *Anas platyrhynchos* | SRR1796039 |
| *Anas platyrhynchos* | SRR1295593 |
| *Anas platyrhynchos* | SRR5204381 |
| *Anas platyrhynchos* | SRR1055272 |
| *Anas platyrhynchos* | SRR5131566 |
| *Anas platyrhynchos* | SRR1796029 |
| *Anas platyrhynchos* | SRR5131565 |
| *Anas platyrhynchos* | SRR5131568 |
| *Anas platyrhynchos* | SRR1796040 |
| *Anas platyrhynchos* | SRR1796024 |
| *Anas platyrhynchos* | SRR1796041 |
| *Anas platyrhynchos* | SRR1295594 |
| *Anas platyrhynchos* | SRR1295588 |
| *Anas platyrhynchos* | SRR1796033 |
| *Anas platyrhynchos* | SRR1796036 |
| *Anas platyrhynchos* | SRR1796028 |
| *Anas platyrhynchos* | SRR1796023 |
| *Anas platyrhynchos* | SRR5131569 |
| *Anas platyrhynchos* | SRR1796025 |
| *Anas platyrhynchos* | SRR4434791 |
| *Anas platyrhynchos* | SRR1796038 |
| *Anas platyrhynchos* | SRR5131563 |
| *Anas platyrhynchos* | SRR4434781 |
| *Anas platyrhynchos* | SRR5131562 |
| *Anas platyrhynchos* | SRR1796035 |
| *Anas platyrhynchos* | SRR1796027 |
| *Anas platyrhynchos* | SRR4434794 |
| *Anas platyrhynchos* | SRR5131567 |
| *Anas platyrhynchos* | SRR4434780 |
| *Anas platyrhynchos* | SRR1796030 |
| *Anas platyrhynchos* | SRR5131564 |
| *Anas platyrhynchos* | SRR1796022 |
| *Anas platyrhynchos* | SRR4434784 |
| *Anas platyrhynchos* | SRR4434796 |
| *Anas platyrhynchos* | SRR4434795 |
| *Anas platyrhynchos* | SRR4434782 |
| *Anas platyrhynchos* | SRR4434788 |
| *Anas platyrhynchos* | SRR1796034 |
| *Anas platyrhynchos* | SRR4434786 |
| *Anas platyrhynchos* | SRR4434793 |
| *Anas platyrhynchos* | SRR4434783 |
| *Anas platyrhynchos* | SRR4434797 |
| *Anas platyrhynchos* | SRR4434790 |
| *Anas platyrhynchos* | SRR4434787 |
| *Anas platyrhynchos* | SRR1295549 |
| *Anas platyrhynchos* | SRR4434792 |
| *Anas platyrhynchos* | SRR5204383 |
| *Anas platyrhynchos* | SRR5204382 |
| *Anas platyrhynchos* | SRR5204387 |
| *Anas platyrhynchos* | SRR5204388 |
| *Anas platyrhynchos* | SRR1610925 |
| *Anas platyrhynchos* | SRR1610929 |
| *Anas platyrhynchos* | SRR4434785 |
| *Anas platyrhynchos* | SRR667172 |
| *Anas platyrhynchos* | SRR5204386 |
| *Anas platyrhynchos* | SRR1796031 |
| *Anas platyrhynchos* | SRR4434789 |
| *Anas platyrhynchos* | SRR1610928 |
| *Anas platyrhynchos* | SRR1610924 |
| *Anas platyrhynchos* | SRR1610923 |
| *Anas platyrhynchos* | SRR1610927 |
| *Anas platyrhynchos* | SRR1610926 |
| *Anas platyrhynchos* | SRR1610922 |
| *Anas platyrhynchos* | SRR667173 |
| *Anas platyrhynchos* | SRR3097698 |
| *Anas platyrhynchos* | SRR5204384 |
| *Anas platyrhynchos* | SRR797841 |
| *Anas platyrhynchos* | SRR797836 |
| *Anas platyrhynchos* | SRR3097699 |
| *Anas platyrhynchos* | SRR3097702 |
| *Anas platyrhynchos* | SRR1295590 |
| *Anas platyrhynchos* | SRR3097710 |
| *Anas platyrhynchos* | SRR797840 |
| *Anas platyrhynchos* | SRR3097695 |
| *Anas platyrhynchos* | SRR3097696 |
| *Anas platyrhynchos* | SRR3097697 |
| *Anas platyrhynchos* | SRR797837 |
| *Anas platyrhynchos* | SRR3097707 |
| *Anas platyrhynchos* | SRR797838 |
| *Anas platyrhynchos* | SRR3097705 |
| *Anas platyrhynchos* | SRR797839 |
| *Anas platyrhynchos* | SRR3097709 |
| *Anas platyrhynchos* | SRR3097708 |
| *Anas platyrhynchos* | SRR3097701 |
| *Anas platyrhynchos* | SRR3097706 |
| *Anas platyrhynchos* | SRR5098026 |
| *Anas platyrhynchos* | SRR3097703 |
| *Anas platyrhynchos* | SRR3097700 |
| *Anas platyrhynchos* | SRR797835 |
| *Anas platyrhynchos* | SRR5098023 |
| *Anas platyrhynchos* | SRR5098022 |
| *Anas platyrhynchos* | SRR5098028 |
| *Anas platyrhynchos* | SRR3097704 |
| *Anas platyrhynchos* | SRR5098029 |
| *Anas platyrhynchos* | SRR906437 |
| *Anas platyrhynchos* | SRR5098025 |
| *Anas platyrhynchos* | ERR515179 |
| *Anas platyrhynchos* | SRR3747332 |
| *Anser* | SRR3030923 |
| *Anser* | SRR3030953 |
| *Anser* | SRR3030915 |
| *Anser* | SRR3030920 |
| *Anser* | SRR3030922 |
| *Anser* | SRR3030919 |
| *Anser* | SRR3030925 |
| *Anser* | SRR3030918 |
| *Anser* | SRR3030917 |
| *Anser* | SRR3030916 |
| *Anser* | SRR2124850 |
| *Anser* | SRR3030979 |
| *Anser* | SRR3030950 |
| *Anser* | SRR3030952 |
| *Anser* | SRR3030949 |
| *Anser* | SRR2124861 |
| *Anser* | SRR3030954 |
| *Anser* | SRR2124855 |
| *Anser* | SRR2912172 |
| *Anser* | SRR2912171 |
| *Anser* | SRR2912230 |
| *Anser* | SRR2912231 |
| *Anser* | SRR2912170 |
| *Anser* | SRR2912200 |
| *Anser* | SRR2912229 |
| *Anser* | SRR2912228 |
| *Anser* | SRR2912234 |
| *Anser* | SRR2912227 |
| *Anser* | SRR2912208 |
| *Anser* | SRR2912226 |
| *Anser* | SRR2912205 |
| *Anser* | SRR2912225 |
| *Anser* | SRR2912236 |
| *Anser anser* | SRR3030913 |
| *Anser anser* | SRR3030912 |
| *Anser anser* | SRR3023402 |
| *Anser anser* | SRR3023401 |
| *Anser anser* | SRR3023400 |
| *Anser anser* | SRR3030914 |
| *Anser anser* | SRR3225584 |
| *Anser anser* | SRR3303824 |
| *Anser anser* | SRR3303825 |
| *Anser anser* | SRR3303828 |
| *Anser anser* | SRR2912184 |
| *Anser anser* | SRR2912187 |
| *Anser anser* | SRR3303826 |
| *Anser anser* | SRR3303827 |
| *Anser anser* | SRR2912186 |
| *Anser anser* | SRR2912167 |
| *Anser anser* | SRR2912169 |
| *Anser anser* | SRR2912168 |
| *Anser cygnoides* | SRR1051740 |
| *Anser cygnoides* | SRR1051741 |
| *Anser cygnoides* | SRR1119186 |
| *Anser cygnoides* | SRR1796009 |
| *Anser cygnoides* | SRR1796019 |
| *Anser cygnoides* | SRR1796016 |
| *Anser cygnoides* | SRR1796008 |
| *Anser cygnoides* | SRR1796014 |
| *Anser cygnoides* | SRR1796015 |
| *Anser cygnoides* | SRR1796011 |
| *Anser cygnoides* | SRR1796000 |
| *Anser cygnoides* | SRR1796013 |
| *Anser cygnoides* | SRR1796017 |
| *Anser cygnoides* | SRR1796005 |
| *Anser cygnoides* | SRR1796020 |
| *Anser cygnoides* | SRR1796010 |
| *Anser cygnoides* | SRR1796021 |
| *Anser cygnoides* | SRR1796002 |
| *Anser cygnoides* | SRR1796004 |
| *Anser cygnoides* | SRR1796006 |
| *Anser cygnoides* | SRR1796012 |
| *Anser cygnoides* | SRR1796003 |
| *Anser cygnoides* | SRR1795999 |
| *Anser cygnoides* | SRR1502193 |
| *Anser cygnoides* | SRR2125737 |
| *Anser cygnoides* | SRR1502195 |
| *Anser cygnoides* | SRR1502197 |
| *Anser cygnoides* | SRR1502198 |
| *Anser cygnoides* | SRR5006762 |
| *Anser cygnoides* | SRR5006761 |
| *Anser cygnoides* | SRR5006763 |
| *Anser cygnoides* | SRR5019747 |
| *Anser sp.* | SRR1060398 |
| *Aptenodytes forsteri* | SRR1693186 |
| *Aptenodytes patagonicus* | SRR1324794 |
| *Aptenodytes patagonicus* | SRR1324791 |
| *Aptenodytes patagonicus* | SRR1324789 |
| *Aptenodytes patagonicus* | SRR1324792 |
| *Aptenodytes patagonicus* | SRR1324788 |
| *Aptenodytes patagonicus* | SRR1324795 |
| *Aptenodytes patagonicus* | SRR1324790 |
| *Aptenodytes patagonicus* | SRR1324793 |
| *Aptenodytes patagonicus* | SRR1324796 |
| *Aptenodytes patagonicus* | SRR1324797 |
| *Apteryx australis mantelli* | SRR067426 |
| *Apteryx australis mantelli* | ERR522067 |
| *Apteryx australis mantelli* | ERR522068 |
| *Apteryx owenii* | SRR3480137 |
| *Apteryx owenii* | SRR3496354 |
| *Apteryx owenii* | SRR3480321 |
| *Apteryx owenii* | SRR3496353 |
| *Apteryx owenii* | SRR3496343 |
| *Apteryx owenii* | SRR3486430 |
| *Apteryx owenii* | SRR3496351 |
| *Apteryx owenii* | SRR3480211 |
| *Apteryx owenii* | SRR3496355 |
| *Apteryx owenii* | SRR3486429 |
| *Apteryx owenii* | SRR3496356 |
| *Apteryx owenii* | SRR3486428 |
| *Apteryx owenii* | SRR3496352 |
| *Apteryx owenii* | SRR3480320 |
| *Apteryx owenii* | SRR3496344 |
| *Apteryx rowi* | SRR3486432 |
| *Apteryx rowi* | SRR3496358 |
| *Apteryx rowi* | SRR3486427 |
| *Apteryx rowi* | SRR3496347 |
| *Apteryx rowi* | SRR3486436 |
| *Apteryx rowi* | SRR3496357 |
| *Apteryx rowi* | SRR3486433 |
| *Apteryx rowi* | SRR3496345 |
| *Apteryx rowi* | SRR3486431 |
| *Apteryx rowi* | SRR3496346 |
| *Apteryx rowi* | SRR3486435 |
| *Apteryx rowi* | SRR3496350 |
| *Apteryx rowi* | SRR3486437 |
| *Apteryx rowi* | SRR3496349 |
| *Apteryx rowi* | SRR3486434 |
| *Apteryx rowi* | SRR3496348 |
| *Apus affinis* | SRR4107077 |
| *Apus affinis* | SRR4107118 |
| *Aquila chrysaetos canadensis* | SRR1818011 |
| *Aquila chrysaetos canadensis* | SRR1817947 |
| *Aquila chrysaetos canadensis* | SRR1818080 |
| *Archilochus colubris* | SRR029421 |
| *Archilochus colubris* | SRR5237173 |
| *Asio otus* | SRR3203220 |
| *Athene noctua* | SRR3203242 |
| *Bubo bubo* | SRR3203225 |
| *Butastur indicus* | SRR3203233 |
| *Buteo buteo* | ERR441003 |
| *Buteo buteo* | ERR441001 |
| *Buteo buteo* | ERR441000 |
| *Buteo buteo* | ERR441002 |
| *Calidris pugnax* | SRR400307 |
| *Calidris pugnax* | SRR400311 |
| *Calidris pugnax* | SRR400304 |
| *Calidris pugnax* | SRR400305 |
| *Calidris pugnax* | SRR400309 |
| *Calidris pugnax* | SRR400315 |
| *Calidris pugnax* | SRR400310 |
| *Calidris pugnax* | SRR400308 |
| *Calidris pugnax* | SRR400313 |
| *Calidris pugnax* | SRR400314 |
| *Calidris pugnax* | SRR400316 |
| *Calidris pugnax* | ERR1018142 |
| *Calidris pugnax* | ERR1018143 |
| *Calidris pugnax* | ERR1018134 |
| *Calidris pugnax* | ERR1018151 |
| *Calidris pugnax* | ERR1018138 |
| *Calidris pugnax* | ERR1018140 |
| *Calidris pugnax* | ERR1018147 |
| *Calidris pugnax* | ERR1018145 |
| *Calidris pugnax* | ERR1018135 |
| *Calidris pugnax* | ERR1018136 |
| *Calidris pugnax* | ERR1018141 |
| *Calidris pugnax* | ERR1018149 |
| *Calidris pugnax* | ERR1018150 |
| *Calidris pugnax* | ERR1018144 |
| *Calidris pugnax* | ERR1018139 |
| *Calidris pugnax* | ERR1018146 |
| *Calidris pugnax* | ERR1018137 |
| *Calidris pugnax* | ERR1018148 |
| *Calypte anna* | SRR029422 |
| *Catharus ustulatus* | SRR4340326 |
| *Catharus ustulatus* | SRR4340333 |
| *Catharus ustulatus* | SRR4340329 |
| *Catharus ustulatus* | SRR4340338 |
| *Catharus ustulatus* | SRR4340331 |
| *Catharus ustulatus* | SRR4340342 |
| *Catharus ustulatus* | SRR4340328 |
| *Catharus ustulatus* | SRR4340341 |
| *Catharus ustulatus* | SRR4340324 |
| *Catharus ustulatus* | SRR4340325 |
| *Catharus ustulatus* | SRR4340334 |
| *Catharus ustulatus* | SRR4340335 |
| *Catharus ustulatus* | SRR4340336 |
| *Catharus ustulatus* | SRR4340337 |
| *Catharus ustulatus* | SRR4340327 |
| *Catharus ustulatus* | SRR4340340 |
| *Catharus ustulatus* | SRR4340332 |
| *Catharus ustulatus* | SRR4340339 |
| *Catharus ustulatus* | SRR4340330 |
| *Chloris chloris* | SRR1210636 |
| *Chloris chloris* | SRR1210588 |
| *Chloris chloris* | SRR1210634 |
| *Chloris chloris* | SRR1210642 |
| *Chloris chloris* | SRR1210637 |
| *Chloris chloris* | SRR1210635 |
| *Chloris chloris* | SRR1210631 |
| *Chloris chloris* | SRR1210604 |
| *Circus melanoleucos* | SRR3203217 |
| *Colinus virginianus* | SRR036708 |
| *Columba livia* | ERR1691876 |
| *Columba livia* | ERR1691882 |
| *Columba livia* | ERR1691867 |
| *Columba livia* | ERR1691883 |
| *Columba livia* | ERR1691864 |
| *Columba livia* | ERR1691865 |
| *Columba livia* | ERR1691880 |
| *Columba livia* | ERR1691866 |
| *Columba livia* | ERR1691871 |
| *Columba livia* | ERR1691909 |
| *Columba livia* | ERR1691920 |
| *Columba livia* | ERR1691870 |
| *Columba livia* | ERR1691863 |
| *Columba livia* | ERR1691907 |
| *Columba livia* | ERR1691899 |
| *Columba livia* | SRR4929905 |
| *Columba livia* | SRR4929903 |
| *Columba livia* | SRR4929902 |
| *Columba livia* | SRR4929904 |
| *Columba livia* | SRR4929897 |
| *Columba livia* | SRR4929901 |
| *Columba livia* | ERR1691921 |
| *Columba livia* | SRR4929898 |
| *Columba livia* | SRR4929894 |
| *Columba livia* | SRR4929900 |
| *Columba livia* | SRR4929896 |
| *Columba livia* | SRR4929885 |
| *Columba livia* | SRR4929899 |
| *Columba livia* | SRR4929895 |
| *Columba livia* | SRR4929877 |
| *Columba livia* | SRR4929883 |
| *Columba livia* | SRR4929875 |
| *Columba livia* | SRR4929882 |
| *Columba livia* | SRR4929884 |
| *Columba livia* | SRR4929893 |
| *Columba livia* | SRR4929876 |
| *Columba livia* | SRR4929874 |
| *Columba livia* | SRR4929881 |
| *Columba livia* | SRR4929873 |
| *Columba livia* | SRR4929890 |
| *Columba livia* | SRR4929878 |
| *Columba livia* | SRR4929892 |
| *Columba livia* | SRR4929880 |
| *Columba livia* | SRR4929891 |
| *Columba livia* | SRR4929879 |
| *Columba livia* | ERR1691905 |
| *Columba livia* | SRR4929870 |
| *Columba livia* | SRR4929871 |
| *Columba livia* | SRR4929872 |
| *Columba livia* | ERR1691891 |
| *Columba livia* | ERR1691892 |
| *Columba livia* | ERR1691896 |
| *Columba livia* | ERR1691862 |
| *Columba livia* | ERR1691887 |
| *Columba livia* | ERR1691878 |
| *Columba livia* | ERR1691889 |
| *Columba livia* | ERR1691872 |
| *Columba livia* | SRR4929889 |
| *Columba livia* | SRR4929886 |
| *Columba livia* | SRR4929888 |
| *Columba livia* | SRR4929887 |
| *Columba livia* | ERR1691895 |
| *Columba livia* | ERR1691894 |
| *Columba livia* | ERR1691903 |
| *Columba livia* | ERR1691886 |
| *Columba livia* | ERR1691890 |
| *Columba livia* | ERR1691877 |
| *Columba livia* | ERR1691893 |
| *Columba livia* | ERR1691915 |
| *Columba livia* | ERR1691918 |
| *Columba livia* | ERR1691898 |
| *Columba livia* | ERR1691885 |
| *Columba livia* | ERR1691913 |
| *Columba livia* | ERR1691869 |
| *Columba livia* | SRR521360 |
| *Columba livia* | ERR1691884 |
| *Columba livia* | ERR1691922 |
| *Columba livia* | ERR1691916 |
| *Columba livia* | ERR1691908 |
| *Columba livia* | ERR1691919 |
| *Columba livia* | ERR1691917 |
| *Columba livia* | ERR1691900 |
| *Columba livia* | SRR521359 |
| *Columba livia* | ERR1691914 |
| *Columba livia* | SRR2094777 |
| *Columba livia* | SRR521361 |
| *Columba livia* | ERR1691910 |
| *Columba livia* | ERR1691881 |
| *Columba livia* | SRR2148831 |
| *Columba livia* | ERR1691897 |
| *Columba livia* | SRR2148833 |
| *Columba livia* | SRR521362 |
| *Columba livia* | SRR2148830 |
| *Columba livia* | SRR521358 |
| *Columba livia* | ERR1691906 |
| *Columba livia* | ERR1691874 |
| *Columba livia* | ERR1691912 |
| *Columba livia* | ERR1691911 |
| *Columba livia* | ERR1691868 |
| *Columba livia* | ERR1691888 |
| *Columba livia* | ERR1691902 |
| *Columba livia* | SRR2094734 |
| *Columba livia* | SRR2094879 |
| *Columba livia* | SRR521357 |
| *Columba livia* | SRR2094799 |
| *Columba livia* | SRR2094789 |
| *Columba livia* | ERR1691904 |
| *Columba livia* | SRR2094764 |
| *Columba livia* | ERR1691901 |
| *Columba livia* | ERR1691873 |
| *Columba livia* | SRR2148832 |
| *Columba livia* | ERR1691879 |
| *Columba livia* | SRR2148834 |
| *Columba livia* | SRR2148835 |
| *Columba livia* | SRR2094746 |
| *Columba livia* | ERR1691875 |
| *Corvus brachyrhynchos* | SRR029463 |
| *Corvus brachyrhynchos* | SRR029464 |
| *Corvus cornix* | SRR019144 |
| *Corvus cornix cornix* | SRR1947444 |
| *Corvus cornix cornix* | SRR946432 |
| *Corvus cornix cornix* | SRR1947452 |
| *Corvus cornix cornix* | SRR1947387 |
| *Corvus cornix cornix* | SRR1947441 |
| *Corvus cornix cornix* | SRR1947405 |
| *Corvus cornix cornix* | SRR1947456 |
| *Corvus cornix cornix* | SRR1947431 |
| *Corvus cornix cornix* | SRR947851 |
| *Corvus cornix cornix* | SRR1947474 |
| *Corvus cornix cornix* | SRR2106828 |
| *Corvus cornix cornix* | SRR1947437 |
| *Corvus cornix cornix* | SRR1928171 |
| *Corvus cornix cornix* | SRR1947408 |
| *Corvus cornix cornix* | SRR1947414 |
| *Corvus cornix cornix* | SRR947859 |
| *Corvus cornix cornix* | SRR1947406 |
| *Corvus cornix cornix* | SRR947870 |
| *Corvus cornix cornix* | SRR1947417 |
| *Corvus cornix cornix* | SRR1947399 |
| *Corvus cornix cornix* | SRR1947420 |
| *Corvus cornix cornix* | SRR1947433 |
| *Corvus cornix cornix* | SRR947835 |
| *Corvus cornix cornix* | SRR947857 |
| *Corvus cornix cornix* | SRR1947384 |
| *Corvus cornix cornix* | SRR1947421 |
| *Corvus cornix cornix* | SRR1947478 |
| *Corvus cornix cornix* | SRR946450 |
| *Corvus cornix cornix* | SRR1947472 |
| *Corvus cornix cornix* | SRR1947422 |
| *Corvus cornix cornix* | SRR2107332 |
| *Corvus cornix cornix* | SRR1947438 |
| *Corvus cornix cornix* | SRR947854 |
| *Corvus cornix cornix* | SRR1947449 |
| *Corvus cornix cornix* | SRR947833 |
| *Corvus cornix cornix* | SRR2107367 |
| *Corvus cornix cornix* | SRR1947413 |
| *Corvus cornix cornix* | SRR1947435 |
| *Corvus cornix cornix* | SRR2106747 |
| *Corvus cornix cornix* | SRR2106742 |
| *Corvus cornix cornix* | SRR1947415 |
| *Corvus cornix cornix* | SRR1947467 |
| *Corvus cornix cornix* | SRR2106792 |
| *Corvus cornix cornix* | SRR1947429 |
| *Corvus cornix cornix* | SRR1947385 |
| *Corvus cornix cornix* | SRR2106802 |
| *Corvus cornix cornix* | SRR2106918 |
| *Corvus cornix cornix* | SRR1947461 |
| *Corvus cornix cornix* | SRR2107333 |
| *Corvus cornix cornix* | SRR1947455 |
| *Corvus cornix cornix* | SRR2107334 |
| *Corvus cornix cornix* | SRR2107331 |
| *Corvus cornix cornix* | SRR2106917 |
| *Corvus cornix cornix* | SRR1947475 |
| *Corvus cornix cornix* | SRR1947447 |
| *Corvus cornix cornix* | SRR2107327 |
| *Corvus cornix cornix* | SRR2106888 |
| *Corvus cornix cornix* | SRR2107220 |
| *Corvus cornix cornix* | SRR1947477 |
| *Corvus cornix cornix* | SRR2107326 |
| *Corvus cornix cornix* | SRR1947416 |
| *Corvus cornix cornix* | SRR947860 |
| *Corvus cornix cornix* | SRR1947386 |
| *Corvus cornix cornix* | SRR1947473 |
| *Corvus cornix cornix* | SRR947855 |
| *Corvus cornix cornix* | SRR1947393 |
| *Corvus cornix cornix* | SRR1947476 |
| *Corvus cornix cornix* | SRR1947440 |
| *Corvus cornix cornix* | SRR1947446 |
| *Corvus cornix cornix* | SRR1947471 |
| *Corvus cornix cornix* | SRR2106916 |
| *Corvus cornix cornix* | SRR2107373 |
| *Corvus cornix cornix* | SRR1947479 |
| *Corvus cornix cornix* | SRR1947423 |
| *Corvus cornix cornix* | SRR1947445 |
| *Corvus cornix cornix* | SRR2106863 |
| *Corvus cornix cornix* | SRR2107362 |
| *Corvus cornix cornix* | SRR1947463 |
| *Corvus cornix cornix* | SRR1947400 |
| *Corvus cornix cornix* | SRR947853 |
| *Corvus cornix cornix* | SRR1947460 |
| *Corvus cornix cornix* | SRR2106849 |
| *Corvus cornix cornix* | SRR1947395 |
| *Corvus cornix cornix* | SRR1947425 |
| *Corvus cornix cornix* | SRR1947480 |
| *Corvus cornix cornix* | SRR1947470 |
| *Corvus cornix cornix* | SRR1947407 |
| *Corvus cornix cornix* | SRR1947383 |
| *Corvus cornix cornix* | SRR947852 |
| *Corvus cornix cornix* | SRR1947402 |
| *Corvus cornix cornix* | SRR1947443 |
| *Corvus cornix cornix* | SRR1947430 |
| *Corvus cornix cornix* | SRR1947453 |
| *Corvus cornix cornix* | SRR1947403 |
| *Corvus cornix cornix* | SRR1947388 |
| *Corvus cornix cornix* | SRR947858 |
| *Corvus cornix cornix* | SRR1947394 |
| *Corvus cornix cornix* | SRR1947411 |
| *Corvus cornix cornix* | SRR1947451 |
| *Corvus cornix cornix* | SRR947856 |
| *Corvus corone corone* | SRR019143 |
| *Corvus corone corone* | SRR1947458 |
| *Corvus corone corone* | SRR1947428 |
| *Corvus corone corone* | SRR1947389 |
| *Corvus corone corone* | SRR947871 |
| *Corvus corone corone* | SRR1947466 |
| *Corvus corone corone* | SRR947869 |
| *Corvus corone corone* | SRR947867 |
| *Corvus corone corone* | SRR1947465 |
| *Corvus corone corone* | SRR1947391 |
| *Corvus corone corone* | SRR947862 |
| *Corvus corone corone* | SRR1947442 |
| *Corvus corone corone* | SRR1947464 |
| *Corvus corone corone* | SRR2107343 |
| *Corvus corone corone* | SRR1947412 |
| *Corvus corone corone* | SRR1947404 |
| *Corvus corone corone* | SRR947865 |
| *Corvus corone corone* | SRR1947397 |
| *Corvus corone corone* | SRR1947448 |
| *Corvus corone corone* | SRR2106782 |
| *Corvus corone corone* | SRR2106836 |
| *Corvus corone corone* | SRR1947392 |
| *Corvus corone corone* | SRR2107130 |
| *Corvus corone corone* | SRR947863 |
| *Corvus corone corone* | SRR947795 |
| *Corvus corone corone* | SRR2106913 |
| *Corvus corone corone* | SRR947866 |
| *Corvus corone corone* | SRR2107364 |
| *Corvus corone corone* | SRR1947401 |
| *Corvus corone corone* | SRR947861 |
| *Corvus corone corone* | SRR947864 |
| *Corvus corone corone* | SRR1947409 |
| *Corvus corone corone* | SRR2106753 |
| *Corvus corone corone* | SRR1947436 |
| *Corvus corone corone* | SRR1947410 |
| *Corvus corone corone* | SRR1947427 |
| *Corvus corone corone* | SRR1947419 |
| *Corvus corone corone* | SRR2106734 |
| *Corvus corone corone* | SRR2106809 |
| *Corvus corone corone* | SRR2107328 |
| *Corvus corone corone* | SRR1947450 |
| *Corvus corone corone* | SRR947793 |
| *Corvus corone corone* | SRR1947426 |
| *Corvus corone corone* | SRR1947462 |
| *Corvus corone corone* | SRR1947398 |
| *Corvus corone corone* | SRR947868 |
| *Corvus corone corone* | SRR1947390 |
| *Corvus corone corone* | SRR1947457 |
| *Corvus corone corone* | SRR1947424 |
| *Corvus corone corone* | SRR1947418 |
| *Corvus corone corone* | SRR1947468 |
| *Corvus corone corone* | SRR1947434 |
| *Corvus corone corone* | SRR1947469 |
| *Corvus corone corone* | SRR1947396 |
| *Corvus corone corone* | SRR1947454 |
| *Corvus corone corone* | SRR1947439 |
| *Corvus corone corone* | SRR1947432 |
| *Corvus corone corone* | SRR1947459 |
| *Corvus macrorhynchos* | SRR1023633 |
| *Corvus macrorhynchos* | SRR1023635 |
| *Coturnix japonica* | SRR039139 |
| *Coturnix japonica* | SRR039145 |
| *Coturnix japonica* | SRR039079 |
| *Coturnix japonica* | SRR039140 |
| *Coturnix japonica* | SRR039141 |
| *Coturnix japonica* | SRR039143 |
| *Coturnix japonica* | SRR1352724 |
| *Coturnix japonica* | ERR756313 |
| *Coturnix japonica* | ERR756297 |
| *Coturnix japonica* | ERR756306 |
| *Coturnix japonica* | ERR756311 |
| *Coturnix japonica* | ERR756332 |
| *Coturnix japonica* | ERR756320 |
| *Coturnix japonica* | ERR756330 |
| *Coturnix japonica* | ERR756305 |
| *Coturnix japonica* | ERR756322 |
| *Coturnix japonica* | ERR756324 |
| *Coturnix japonica* | ERR756321 |
| *Coturnix japonica* | ERR756319 |
| *Coturnix japonica* | ERR756333 |
| *Coturnix japonica* | ERR756309 |
| *Coturnix japonica* | ERR756302 |
| *Coturnix japonica* | ERR756328 |
| *Coturnix japonica* | ERR756314 |
| *Coturnix japonica* | ERR756308 |
| *Coturnix japonica* | ERR756315 |
| *Coturnix japonica* | ERR756296 |
| *Coturnix japonica* | ERR756312 |
| *Coturnix japonica* | ERR756318 |
| *Coturnix japonica* | ERR756327 |
| *Coturnix japonica* | ERR756304 |
| *Coturnix japonica* | ERR756331 |
| *Coturnix japonica* | ERR756329 |
| *Coturnix japonica* | ERR756300 |
| *Coturnix japonica* | ERR756323 |
| *Coturnix japonica* | ERR756303 |
| *Coturnix japonica* | ERR756326 |
| *Coturnix japonica* | ERR756310 |
| *Coturnix japonica* | SRR345225 |
| *Coturnix japonica* | ERR756301 |
| *Coturnix japonica* | ERR756317 |
| *Coturnix japonica* | ERR756316 |
| *Coturnix japonica* | ERR756325 |
| *Coturnix japonica* | ERR756298 |
| *Coturnix japonica* | SRR345224 |
| *Coturnix japonica* | ERR756307 |
| *Coturnix japonica* | ERR756299 |
| *Coturnix japonica* | SRR3218003 |
| *Coturnix japonica* | SRR3222240 |
| *Coturnix japonica* | SRR3222233 |
| *Coturnix japonica* | SRR3218008 |
| *Coturnix japonica* | SRR3218011 |
| *Coturnix japonica* | SRR3217994 |
| *Coturnix japonica* | SRR3222236 |
| *Coturnix japonica* | SRR3222224 |
| *Coturnix japonica* | SRR3222220 |
| *Coturnix japonica* | SRR3218002 |
| *Coturnix japonica* | SRR3222225 |
| *Coturnix japonica* | SRR3222211 |
| *Coturnix japonica* | SRR3218001 |
| *Coturnix japonica* | SRR3222215 |
| *Coturnix japonica* | SRR3222227 |
| *Coturnix japonica* | SRR3222219 |
| *Coturnix japonica* | SRR3222291 |
| *Coturnix japonica* | DRR045018 |
| *Coturnix japonica* | SRR3222243 |
| *Coturnix japonica* | SRR3222222 |
| *Coturnix japonica* | SRR3218006 |
| *Coturnix japonica* | SRR3222221 |
| *Coturnix japonica* | SRR3217985 |
| *Coturnix japonica* | SRR3217990 |
| *Coturnix japonica* | SRR3222217 |
| *Coturnix japonica* | SRR3222234 |
| *Coturnix japonica* | SRR3222229 |
| *Coturnix japonica* | SRR3218000 |
| *Coturnix japonica* | SRR3217988 |
| *Coturnix japonica* | SRR3222218 |
| *Coturnix japonica* | SRR3217986 |
| *Coturnix japonica* | SRR3222210 |
| *Coturnix japonica* | ERR1346243 |
| *Coturnix japonica* | SRR3217991 |
| *Coturnix japonica* | SRR3217980 |
| *Coturnix japonica* | SRR3217997 |
| *Coturnix japonica* | DRR045017 |
| *Coturnix japonica* | SRR3222244 |
| *Coturnix japonica* | SRR3217989 |
| *Coturnix japonica* | SRR3217987 |
| *Coturnix japonica* | SRR3222242 |
| *Coturnix japonica* | ERR1346221 |
| *Coturnix japonica* | SRR3217981 |
| *Coturnix japonica* | SRR3222292 |
| *Coturnix japonica* | SRR3222214 |
| *Coturnix japonica* | ERR1346238 |
| *Coturnix japonica* | SRR3222230 |
| *Coturnix japonica* | SRR3218010 |
| *Coturnix japonica* | SRR3217993 |
| *Coturnix japonica* | SRR3218013 |
| *Coturnix japonica* | ERR1346237 |
| *Coturnix japonica* | SRR3217982 |
| *Coturnix japonica* | SRR3217996 |
| *Coturnix japonica* | SRR3217999 |
| *Coturnix japonica* | SRR3218014 |
| *Coturnix japonica* | ERR1346240 |
| *Coturnix japonica* | ERR1346235 |
| *Coturnix japonica* | SRR3222293 |
| *Coturnix japonica* | SRR3218009 |
| *Coturnix japonica* | SRR3217998 |
| *Coturnix japonica* | ERR1346220 |
| *Coturnix japonica* | SRR3217984 |
| *Coturnix japonica* | ERR1346222 |
| *Coturnix japonica* | SRR3222216 |
| *Coturnix japonica* | SRR3222212 |
| *Coturnix japonica* | SRR3218012 |
| *Coturnix japonica* | SRR3217983 |
| *Coturnix japonica* | SRR3217992 |
| *Coturnix japonica* | SRR3217995 |
| *Coturnix japonica* | ERR1346239 |
| *Coturnix japonica* | ERR1346229 |
| *Coturnix japonica* | ERR1346227 |
| *Coturnix japonica* | ERR1346230 |
| *Coturnix japonica* | ERR1346234 |
| *Coturnix japonica* | ERR1346224 |
| *Coturnix japonica* | ERR1346226 |
| *Coturnix japonica* | SRR3218004 |
| *Coturnix japonica* | ERR1346223 |
| *Coturnix japonica* | SRR3218005 |
| *Coturnix japonica* | SRR3217979 |
| *Coturnix japonica* | ERR1346242 |
| *Coturnix japonica* | SRR3222237 |
| *Coturnix japonica* | ERR1346228 |
| *Coturnix japonica* | ERR1346225 |
| *Coturnix japonica* | SRR3222223 |
| *Coturnix japonica* | SRR3218007 |
| *Coturnix japonica* | SRR2968870 |
| *Coturnix japonica* | ERR1346236 |
| *Coturnix japonica* | SRR3222241 |
| *Coturnix japonica* | ERR1346231 |
| *Coturnix japonica* | SRR3222228 |
| *Coturnix japonica* | SRR3222235 |
| *Coturnix japonica* | SRR3222226 |
| *Coturnix japonica* | ERR1346232 |
| *Coturnix japonica* | ERR1346233 |
| *Coturnix japonica* | SRR2968896 |
| *Coturnix japonica* | SRR2968895 |
| *Coturnix japonica* | ERR1346241 |
| *Coturnix japonica* | SRR2968880 |
| *Coturnix japonica* | SRR2968908 |
| *Coturnix japonica* | SRR2968899 |
| *Coturnix japonica* | SRR2968898 |
| *Coturnix japonica* | SRR2968897 |
| *Coturnix japonica* | ERR1041372 |
| *Coturnix japonica* | SRR2968909 |
| *Coturnix japonica* | SRR2968910 |
| *Coturnix japonica* | SRR2968881 |
| *Coturnix japonica* | SRR2968883 |
| *Coturnix japonica* | SRR3222239 |
| *Coturnix japonica* | ERR1041377 |
| *Coturnix japonica* | ERR1041369 |
| *Coturnix japonica* | SRR2968873 |
| *Coturnix japonica* | ERR1041374 |
| *Coturnix japonica* | SRR2968872 |
| *Coturnix japonica* | ERR1041380 |
| *Coturnix japonica* | ERR1041368 |
| *Coturnix japonica* | SRR2968886 |
| *Coturnix japonica* | SRR1758122 |
| *Coturnix japonica* | ERR1041378 |
| *Coturnix japonica* | SRR2968911 |
| *Coturnix japonica* | SRR2968885 |
| *Coturnix japonica* | ERR1041376 |
| *Coturnix japonica* | SRR2968884 |
| *Coturnix japonica* | SRR2968874 |
| *Coturnix japonica* | ERR1041375 |
| *Coturnix japonica* | SRR2968887 |
| *Coturnix japonica* | ERR1041370 |
| *Coturnix japonica* | ERR1041371 |
| *Coturnix japonica* | ERR1041366 |
| *Coturnix japonica* | ERR1041367 |
| *Coturnix japonica* | SRR1758112 |
| *Coturnix japonica* | ERR1041381 |
| *Coturnix japonica* | SRR3222232 |
| *Coturnix japonica* | ERR1041373 |
| *Coturnix japonica* | SRR1758116 |
| *Coturnix japonica* | SRR2968871 |
| *Coturnix japonica* | SRR2968882 |
| *Coturnix japonica* | SRR1758123 |
| *Coturnix japonica* | ERR1041379 |
| *Coturnix japonica* | SRR1758119 |
| *Coturnix japonica* | SRR3222231 |
| *Coturnix japonica* | SRR2968907 |
| *Coturnix japonica* | SRR2968904 |
| *Coturnix japonica* | SRR2968893 |
| *Coturnix japonica* | SRR2968894 |
| *Coturnix japonica* | SRR2968875 |
| *Coturnix japonica* | SRR2968876 |
| *Coturnix japonica* | SRR2968902 |
| *Coturnix japonica* | SRR2968901 |
| *Coturnix japonica* | SRR2968888 |
| *Coturnix japonica* | SRR2968889 |
| *Coturnix japonica* | SRR1758121 |
| *Coturnix japonica* | SRR1758118 |
| *Coturnix japonica* | SRR1758115 |
| *Coturnix japonica* | SRR2968879 |
| *Coturnix japonica* | SRR2968906 |
| *Coturnix japonica* | SRR1758114 |
| *Coturnix japonica* | SRR2968878 |
| *Coturnix japonica* | SRR2968905 |
| *Coturnix japonica* | SRR2968877 |
| *Coturnix japonica* | SRR2968903 |
| *Coturnix japonica* | SRR2968892 |
| *Coturnix japonica* | SRR1758117 |
| *Coturnix japonica* | SRR2968891 |
| *Coturnix japonica* | SRR1758113 |
| *Coturnix japonica* | SRR2968890 |
| *Coturnix japonica* | SRR1758120 |
| *Coturnix japonica* | SRR2968900 |
| *Coturnix japonica* | SRR2984772 |
| *Coturnix japonica* | ERR986063 |
| *Coturnix japonica* | SRR2984779 |
| *Coturnix japonica* | ERR986067 |
| *Coturnix japonica* | SRR2984775 |
| *Coturnix japonica* | ERR986066 |
| *Coturnix japonica* | SRR2984759 |
| *Coturnix japonica* | ERR986069 |
| *Coturnix japonica* | SRR2984777 |
| *Coturnix japonica* | ERR986068 |
| *Coturnix japonica* | SRR2984774 |
| *Coturnix japonica* | ERR986065 |
| *Coturnix japonica* | SRR2984776 |
| *Coturnix japonica* | ERR986070 |
| *Coturnix japonica* | SRR2984778 |
| *Coturnix japonica* | ERR986064 |
| *Coturnix japonica* | SRR1346108 |
| *Cyanistes caeruleus* | SRR029162 |
| *Cyanistes caeruleus* | SRR1325069 |
| *Cyanistes caeruleus* | SRR1325072 |
| *Cyanistes caeruleus* | SRR1325076 |
| *Cyanistes caeruleus* | SRR1325073 |
| *Cyanistes caeruleus* | SRR1325070 |
| *Cyanistes caeruleus* | SRR1325075 |
| *Cyanistes caeruleus* | SRR1325068 |
| *Cyanistes caeruleus* | SRR1325067 |
| *Cyanistes caeruleus* | SRR1325071 |
| *Cyanistes caeruleus* | SRR1325074 |
| *Cyanistes caeruleus* | SRR2040626 |
| *Cyanistes caeruleus* | SRR2040636 |
| *Cyanistes caeruleus* | SRR2040623 |
| *Cyanistes caeruleus* | SRR2040630 |
| *Cyanistes caeruleus* | SRR2040836 |
| *Cyanistes caeruleus* | SRR2040624 |
| *Cyanistes caeruleus* | SRR2040629 |
| *Cyanistes caeruleus* | SRR2040633 |
| *Cyanistes caeruleus* | SRR2040634 |
| *Cyanistes caeruleus* | SRR2040635 |
| *Cyanistes caeruleus* | SRR2040846 |
| *Cyanistes caeruleus* | SRR2040631 |
| *Cyanistes caeruleus* | SRR2040637 |
| *Cyanistes caeruleus* | SRR2040622 |
| *Cyanistes caeruleus* | SRR2040839 |
| *Cyanistes caeruleus* | SRR2040638 |
| *Cyanistes caeruleus* | SRR2040837 |
| *Cyanistes caeruleus* | SRR2040641 |
| *Cyanistes caeruleus* | SRR2040838 |
| *Cyanistes caeruleus* | SRR2040835 |
| *Cyanistes caeruleus* | SRR2040628 |
| *Cyanistes caeruleus* | SRR2040640 |
| *Cyanistes caeruleus* | SRR2040632 |
| *Cyanistes caeruleus* | SRR2040627 |
| *Cyanistes caeruleus* | SRR2040645 |
| *Cyanistes caeruleus* | SRR2040625 |
| *Cyanistes caeruleus* | SRR2040639 |
| *Cyanistes caeruleus* | SRR2040854 |
| *Cyanistes caeruleus* | SRR2040853 |
| *Cyanistes caeruleus* | SRR2040849 |
| *Cyanopica cyanus* | SRR1916171 |
| *Cyanopica cyanus* | SRR1917029 |
| *Dromaius novaehollandiae* | SRR029466 |
| *Dromaius novaehollandiae* | SRR029467 |
| *Dromaius novaehollandiae* | SRR787604 |
| *Dromaius novaehollandiae* | SRR787605 |
| *Dromaius novaehollandiae* | SRR787599 |
| *Dromaius novaehollandiae* | SRR787603 |
| *Dromaius novaehollandiae* | SRR787600 |
| *Dromaius novaehollandiae* | SRR787602 |
| *Dromaius novaehollandiae* | SRR787601 |
| *Elanus caeruleus* | SRR3203227 |
| *Eudyptes chrysocome moseleyi* | SRR1324932 |
| *Eudyptes chrysocome moseleyi* | SRR1324933 |
| *Eudyptes chrysocome moseleyi* | SRR1324934 |
| *Eudyptes chrysocome moseleyi* | SRR1324935 |
| *Eudyptes chrysolophus* | SRR5253661 |
| *Eudyptes filholi* | SRR1324929 |
| *Eudyptes filholi* | SRR1324930 |
| *Eudyptes filholi* | SRR1324931 |
| *Eudyptes filholi* | SRR1324928 |
| *Falco* | SRR522907 |
| *Falco* | SRR522906 |
| *Falco cherrug cherrug* | SRR671935 |
| *Falco peregrinus peregrinus* | SRR671934 |
| *Falco sparverius* | SRR3217264 |
| *Falco sparverius* | SRR3217259 |
| *Falco sparverius* | SRR3217263 |
| *Falco sparverius* | SRR3217260 |
| *Falco sparverius* | SRR3217261 |
| *Falco sparverius* | SRR3217262 |
| *Falco sparverius* | SRR3217265 |
| *Falco sparverius* | SRR3217258 |
| *Falco sparverius* | SRR5270413 |
| *Falco sparverius* | SRR5070564 |
| *Falco sparverius* | SRR5270429 |
| *Falco sparverius* | SRR5270417 |
| *Falco sparverius* | SRR5270424 |
| *Falco sparverius* | SRR5270426 |
| *Falco sparverius* | SRR5270422 |
| *Falco sparverius* | SRR5270425 |
| *Falco sparverius* | SRR5270414 |
| *Falco sparverius* | SRR5270419 |
| *Falco sparverius* | SRR5270423 |
| *Falco sparverius* | SRR5270421 |
| *Falco sparverius* | SRR5270415 |
| *Falco sparverius* | SRR5270428 |
| *Falco sparverius* | SRR5270416 |
| *Falco sparverius* | SRR5270418 |
| *Falco sparverius* | SRR5270420 |
| *Falco sparverius* | SRR5270427 |
| *Falco sparverius* | SRR3217266 |
| *Falco subbuteo* | SRR3203238 |
| *Falco tinnunculus* | SRR3203231 |
| *Ficedula albicollis* | ERR168730 |
| *Ficedula albicollis* | ERR168714 |
| *Ficedula albicollis* | ERR168715 |
| *Ficedula albicollis* | ERR168707 |
| *Ficedula albicollis* | ERR168709 |
| *Ficedula albicollis* | ERR168755 |
| *Ficedula albicollis* | ERR168734 |
| *Ficedula albicollis* | ERR168711 |
| *Ficedula albicollis* | ERR168731 |
| *Ficedula albicollis* | ERR168710 |
| *Ficedula albicollis* | ERR168712 |
| *Ficedula albicollis* | ERR168735 |
| *Ficedula albicollis* | ERR168708 |
| *Ficedula albicollis* | ERR168713 |
| *Ficedula albicollis* | ERR168732 |
| *Ficedula albicollis* | ERR168733 |
| *Ficedula albicollis* | ERR168838 |
| *Ficedula albicollis* | ERR168728 |
| *Ficedula albicollis* | ERR168802 |
| *Ficedula albicollis* | ERR168727 |
| *Ficedula albicollis* | ERR168812 |
| *Ficedula albicollis* | ERR168702 |
| *Ficedula albicollis* | ERR168729 |
| *Ficedula albicollis* | ERR168813 |
| *Ficedula albicollis* | ERR168701 |
| *Ficedula albicollis* | ERR168698 |
| *Ficedula albicollis* | ERR168804 |
| *Ficedula albicollis* | ERR168726 |
| *Ficedula albicollis* | ERR168810 |
| *Ficedula albicollis* | ERR168699 |
| *Ficedula albicollis* | ERR168786 |
| *Ficedula albicollis* | ERR168806 |
| *Ficedula albicollis* | ERR168686 |
| *Ficedula albicollis* | ERR168683 |
| *Ficedula albicollis* | ERR168685 |
| *Ficedula albicollis* | ERR168693 |
| *Ficedula albicollis* | ERR168809 |
| *Ficedula albicollis* | ERR168682 |
| *Ficedula albicollis* | ERR168807 |
| *Ficedula albicollis* | ERR168791 |
| *Ficedula albicollis* | ERR168689 |
| *Ficedula albicollis* | ERR168694 |
| *Ficedula albicollis* | ERR168811 |
| *Ficedula albicollis* | ERR168808 |
| *Ficedula albicollis* | ERR168700 |
| *Ficedula albicollis* | ERR168765 |
| *Ficedula albicollis* | ERR168706 |
| *Ficedula albicollis* | ERR168789 |
| *Ficedula albicollis* | ERR168803 |
| *Ficedula albicollis* | ERR168790 |
| *Ficedula albicollis* | ERR168687 |
| *Ficedula albicollis* | ERR168690 |
| *Ficedula albicollis* | ERR168688 |
| *Ficedula albicollis* | ERR168766 |
| *Ficedula albicollis* | ERR168761 |
| *Ficedula albicollis* | ERR168760 |
| *Ficedula albicollis* | ERR168754 |
| *Ficedula albicollis* | ERR168767 |
| *Ficedula albicollis* | ERR168805 |
| *Ficedula albicollis* | ERR168788 |
| *Ficedula albicollis* | ERR168684 |
| *Ficedula albicollis* | ERR168691 |
| *Ficedula albicollis* | ERR168787 |
| *Ficedula albicollis* | ERR168783 |
| *Ficedula albicollis* | ERR168764 |
| *Ficedula albicollis* | ERR168692 |
| *Ficedula albicollis* | ERR168785 |
| *Ficedula albicollis* | ERR168758 |
| *Ficedula albicollis* | ERR168759 |
| *Ficedula albicollis* | ERR168779 |
| *Ficedula albicollis* | ERR168778 |
| *Ficedula albicollis* | ERR168780 |
| *Ficedula albicollis* | ERR168784 |
| *Ficedula albicollis* | ERR168762 |
| *Ficedula albicollis* | ERR168763 |
| *Ficedula albicollis* | ERR168842 |
| *Ficedula albicollis* | ERR168782 |
| *Ficedula albicollis* | ERR168781 |
| *Ficedula albicollis* | ERR168846 |
| *Ficedula albicollis* | ERR168757 |
| *Ficedula albicollis* | ERR168756 |
| *Ficedula albicollis* | ERR168848 |
| *Ficedula hypoleuca* | SRR029161 |
| *Ficedula hypoleuca* | ERR168801 |
| *Ficedula hypoleuca* | ERR168798 |
| *Ficedula hypoleuca* | ERR168799 |
| *Ficedula hypoleuca* | ERR168800 |
| *Ficedula hypoleuca* | SRR029160 |
| *Ficedula hypoleuca* | SRR029159 |
| *Ficedula hypoleuca* | ERR168797 |
| *Ficedula hypoleuca* | ERR168720 |
| *Ficedula hypoleuca* | ERR168725 |
| *Ficedula hypoleuca* | ERR168719 |
| *Ficedula hypoleuca* | ERR168717 |
| *Ficedula hypoleuca* | ERR168716 |
| *Ficedula hypoleuca* | ERR168724 |
| *Ficedula hypoleuca* | ERR168748 |
| *Ficedula hypoleuca* | ERR168738 |
| *Ficedula hypoleuca* | ERR168723 |
| *Ficedula hypoleuca* | ERR168737 |
| *Ficedula hypoleuca* | ERR168739 |
| *Ficedula hypoleuca* | ERR168740 |
| *Ficedula hypoleuca* | ERR168743 |
| *Ficedula hypoleuca* | ERR168821 |
| *Ficedula hypoleuca* | ERR168722 |
| *Ficedula hypoleuca* | ERR168721 |
| *Ficedula hypoleuca* | ERR168744 |
| *Ficedula hypoleuca* | ERR168741 |
| *Ficedula hypoleuca* | ERR168718 |
| *Ficedula hypoleuca* | ERR168736 |
| *Ficedula hypoleuca* | ERR168742 |
| *Ficedula hypoleuca* | ERR168746 |
| *Ficedula hypoleuca* | ERR168833 |
| *Ficedula hypoleuca* | ERR168703 |
| *Ficedula hypoleuca* | ERR168835 |
| *Ficedula hypoleuca* | ERR168704 |
| *Ficedula hypoleuca* | ERR168745 |
| *Ficedula hypoleuca* | ERR168705 |
| *Ficedula hypoleuca* | ERR168747 |
| *Ficedula hypoleuca* | ERR168827 |
| *Ficedula hypoleuca* | ERR168814 |
| *Ficedula hypoleuca* | ERR168817 |
| *Ficedula hypoleuca* | ERR168820 |
| *Ficedula hypoleuca* | ERR168794 |
| *Ficedula hypoleuca* | ERR168749 |
| *Ficedula hypoleuca* | ERR168777 |
| *Ficedula hypoleuca* | ERR168753 |
| *Ficedula hypoleuca* | ERR168752 |
| *Ficedula hypoleuca* | ERR168796 |
| *Ficedula hypoleuca* | ERR168751 |
| *Ficedula hypoleuca* | ERR168695 |
| *Ficedula hypoleuca* | ERR168775 |
| *Ficedula hypoleuca* | ERR168697 |
| *Ficedula hypoleuca* | ERR168822 |
| *Ficedula hypoleuca* | ERR168834 |
| *Ficedula hypoleuca* | ERR168823 |
| *Ficedula hypoleuca* | ERR168696 |
| *Ficedula hypoleuca* | ERR168815 |
| *Ficedula hypoleuca* | ERR168750 |
| *Ficedula hypoleuca* | ERR168816 |
| *Ficedula hypoleuca* | ERR168830 |
| *Ficedula hypoleuca* | ERR168772 |
| *Ficedula hypoleuca* | ERR168771 |
| *Ficedula hypoleuca* | ERR168825 |
| *Ficedula hypoleuca* | ERR168829 |
| *Ficedula hypoleuca* | ERR168770 |
| *Ficedula hypoleuca* | ERR168837 |
| *Ficedula hypoleuca* | ERR168843 |
| *Ficedula hypoleuca* | ERR168774 |
| *Ficedula hypoleuca* | ERR168819 |
| *Ficedula hypoleuca* | ERR168824 |
| *Ficedula hypoleuca* | ERR168795 |
| *Ficedula hypoleuca* | ERR168769 |
| *Ficedula hypoleuca* | ERR168826 |
| *Ficedula hypoleuca* | ERR168793 |
| *Ficedula hypoleuca* | ERR168792 |
| *Ficedula hypoleuca* | ERR168832 |
| *Ficedula hypoleuca* | ERR168831 |
| *Ficedula hypoleuca* | ERR168773 |
| *Ficedula hypoleuca* | ERR168768 |
| *Ficedula hypoleuca* | ERR168776 |
| *Ficedula hypoleuca* | ERR168818 |
| *Ficedula hypoleuca* | ERR168828 |
| *Ficedula hypoleuca* | ERR168840 |
| *Ficedula hypoleuca* | ERR168847 |
| *Ficedula hypoleuca* | ERR168849 |
| *Ficedula hypoleuca* | ERR168845 |
| *Ficedula hypoleuca* | ERR168836 |
| *Ficedula hypoleuca* | ERR168839 |
| *Ficedula hypoleuca* | ERR168844 |
| *Ficedula hypoleuca* | ERR168841 |
| *Gallinago media* | SRR609721 |
| *Gallinago media* | SRR609949 |
| *Gallinago media* | SRR609946 |
| *Gallinago media* | SRR609950 |
| *Gallinago media* | SRR609717 |
| *Gallinago media* | SRR609720 |
| *Gallinago media* | SRR609718 |
| *Gallinago media* | SRR609948 |
| *Gallinago media* | SRR609719 |
| *Gallinago media* | SRR609944 |
| *Gallinago media* | SRR609716 |
| *Gallinago media* | SRR609715 |
| *Gallinago media* | SRR609945 |
| *Gallinago media* | SRR609947 |
| *Haemorhous mexicanus* | SRR768496 |
| *Haemorhous mexicanus* | SRR768509 |
| *Junco hyemalis* | SRR490869 |
| *Junco hyemalis* | SRR490738 |
| *Junco hyemalis* | SRR1763891 |
| *Junco hyemalis* | SRR1763889 |
| *Junco hyemalis* | SRR1759141 |
| *Junco hyemalis* | SRR1759134 |
| *Junco hyemalis* | SRR1759167 |
| *Junco hyemalis* | SRR1759276 |
| *Junco hyemalis* | SRR1763898 |
| *Junco hyemalis* | SRR1759133 |
| *Junco hyemalis* | SRR1763897 |
| *Junco hyemalis* | SRR1759135 |
| *Junco hyemalis* | SRR1759180 |
| *Junco hyemalis* | SRR1759137 |
| *Junco hyemalis* | SRR1763892 |
| *Junco hyemalis* | SRR1763877 |
| *Junco hyemalis* | SRR1759292 |
| *Junco hyemalis* | SRR1763893 |
| *Junco hyemalis* | SRR1759138 |
| *Junco hyemalis* | SRR1759142 |
| *Junco hyemalis* | SRR1759277 |
| *Junco hyemalis* | SRR1763890 |
| *Junco hyemalis* | SRR1763895 |
| *Junco hyemalis* | SRR1759294 |
| *Junco hyemalis* | SRR1759136 |
| *Junco hyemalis* | SRR1759131 |
| *Junco hyemalis* | SRR1763899 |
| *Junco hyemalis* | SRR1759143 |
| *Junco hyemalis* | SRR1763854 |
| *Junco hyemalis* | SRR1763902 |
| *Junco hyemalis* | SRR1759293 |
| *Junco hyemalis* | SRR1763881 |
| *Junco hyemalis* | SRR1759211 |
| *Junco hyemalis* | SRR1763784 |
| *Junco hyemalis* | SRR1759132 |
| *Junco hyemalis* | SRR1763887 |
| *Junco hyemalis* | SRR1763894 |
| *Junco hyemalis* | SRR1763872 |
| *Junco hyemalis* | SRR1763896 |
| *Junco hyemalis* | SRR1763885 |
| *Junco hyemalis* | SRR1759140 |
| *Lamprotornis superbus* | SRR1567380 |
| *Lamprotornis superbus* | SRR1566236 |
| *Lamprotornis superbus* | SRR1566294 |
| *Lamprotornis superbus* | SRR1567346 |
| *Lamprotornis superbus* | SRR1567393 |
| *Lamprotornis superbus* | SRR1565490 |
| *Lamprotornis superbus* | SRR1565487 |
| *Lamprotornis superbus* | SRR1565489 |
| *Lamprotornis superbus* | SRR1567314 |
| *Lamprotornis superbus* | SRR1565488 |
| *Larus atricilla* | SRR3218029 |
| *Larus atricilla* | SRR3218031 |
| *Larus atricilla* | SRR3218028 |
| *Larus atricilla* | SRR3218026 |
| *Larus atricilla* | SRR3218032 |
| *Larus atricilla* | SRR3218021 |
| *Larus atricilla* | SRR3218025 |
| *Larus atricilla* | SRR3218033 |
| *Larus atricilla* | SRR3218022 |
| *Larus atricilla* | SRR3218030 |
| *Larus atricilla* | SRR3218027 |
| *Larus atricilla* | SRR3218019 |
| *Larus atricilla* | SRR3218024 |
| *Larus atricilla* | SRR3218023 |
| *Larus atricilla* | SRR3218020 |
| *Lepidothrix coronata* | SRR3493972 |
| *Lonchura striata domestica* | SRR5223633 |
| *Lonchura striata domestica* | SRR5223632 |
| *Lonchura striata domestica* | SRR5223634 |
| *Lonchura striata domestica* | SRR5223627 |
| *Lonchura striata domestica* | SRR5223630 |
| *Lonchura striata domestica* | SRR5223628 |
| *Lonchura striata domestica* | SRR5223629 |
| *Lonchura striata domestica* | SRR5223631 |
| *Loxia curvirostra* | SRR834578 |
| *Lyrurus tetrix* | SRR208078 |
| *Malurus lamberti* | SRR3901709 |
| *Manacus vitellinus* | SRR029478 |
| *Manacus vitellinus* | SRR029477 |
| *Manacus vitellinus* | SRR2545932 |
| *Manacus vitellinus* | SRR2545934 |
| *Manacus vitellinus* | SRR2545933 |
| *Manacus vitellinus* | SRR2545929 |
| *Manacus vitellinus* | SRR2545939 |
| *Manacus vitellinus* | SRR2545935 |
| *Manacus vitellinus* | SRR2545940 |
| *Manacus vitellinus* | SRR2545936 |
| *Manacus vitellinus* | SRR2545931 |
| *Manacus vitellinus* | SRR2545930 |
| *Manacus vitellinus* | SRR2545938 |
| *Manacus vitellinus* | SRR2545937 |
| *Manacus vitellinus* | SRR3476292 |
| *Meleagris gallopavo* | SRR1570192 |
| *Meleagris gallopavo* | ERR267986 |
| *Meleagris gallopavo* | SRR1570635 |
| *Meleagris gallopavo* | SRR1570699 |
| *Meleagris gallopavo* | SRR1570359 |
| *Meleagris gallopavo* | SRR1570579 |
| *Meleagris gallopavo* | SRR1570395 |
| *Meleagris gallopavo* | SRR1570311 |
| *Meleagris gallopavo* | SRR1732102 |
| *Meleagris gallopavo* | SRR1570523 |
| *Meleagris gallopavo* | SRR1570647 |
| *Meleagris gallopavo* | SRR1732287 |
| *Meleagris gallopavo* | SRR1570614 |
| *Meleagris gallopavo* | SRR1570703 |
| *Meleagris gallopavo* | SRR1570292 |
| *Meleagris gallopavo* | SRR1570587 |
| *Meleagris gallopavo* | SRR1732273 |
| *Meleagris gallopavo* | SRR1732262 |
| *Meleagris gallopavo* | SRR1570590 |
| *Meleagris gallopavo* | SRR1570607 |
| *Meleagris gallopavo* | SRR1732261 |
| *Meleagris gallopavo* | SRR1570581 |
| *Meleagris gallopavo* | SRR1570320 |
| *Meleagris gallopavo* | SRR1570230 |
| *Meleagris gallopavo* | SRR1570528 |
| *Meleagris gallopavo* | SRR1570597 |
| *Meleagris gallopavo* | SRR1570382 |
| *Meleagris gallopavo* | SRR1570610 |
| *Meleagris gallopavo* | SRR1570331 |
| *Meleagris gallopavo* | SRR1732240 |
| *Meleagris gallopavo* | SRR1570328 |
| *Meleagris gallopavo* | SRR1570375 |
| *Meleagris gallopavo* | SRR1570293 |
| *Meleagris gallopavo* | SRR1570573 |
| *Meleagris gallopavo* | SRR1732288 |
| *Meleagris gallopavo* | SRR1570341 |
| *Meleagris gallopavo* | SRR1661445 |
| *Meleagris gallopavo* | SRR1570251 |
| *Meleagris gallopavo* | SRR1570594 |
| *Meleagris gallopavo* | SRR1732256 |
| *Meleagris gallopavo* | SRR1661443 |
| *Meleagris gallopavo* | SRR1570294 |
| *Meleagris gallopavo* | SRR1661431 |
| *Meleagris gallopavo* | SRR1732277 |
| *Meleagris gallopavo* | SRR1570708 |
| *Meleagris gallopavo* | SRR1661437 |
| *Meleagris gallopavo* | SRR1570233 |
| *Meleagris gallopavo* | SRR1570658 |
| *Meleagris gallopavo* | SRR1570350 |
| *Meleagris gallopavo* | SRR1570589 |
| *Meleagris gallopavo* | SRR1732269 |
| *Meleagris gallopavo* | SRR1570302 |
| *Meleagris gallopavo* | SRR1570599 |
| *Meleagris gallopavo* | SRR1570532 |
| *Meleagris gallopavo* | SRR1570312 |
| *Meleagris gallopavo* | SRR1570429 |
| *Meleagris gallopavo* | SRR1570561 |
| *Meleagris gallopavo* | SRR1570584 |
| *Meleagris gallopavo* | SRR1570296 |
| *Meleagris gallopavo* | SRR1570316 |
| *Meleagris gallopavo* | SRR1570432 |
| *Meleagris gallopavo* | SRR1570725 |
| *Meleagris gallopavo* | SRR1570389 |
| *Meleagris gallopavo* | SRR1570304 |
| *Meleagris gallopavo* | SRR1570351 |
| *Meleagris gallopavo* | SRR1732264 |
| *Meleagris gallopavo* | SRR1570606 |
| *Meleagris gallopavo* | SRR1570193 |
| *Meleagris gallopavo* | SRR1570476 |
| *Meleagris gallopavo* | SRR1661442 |
| *Meleagris gallopavo* | SRR1570518 |
| *Meleagris gallopavo* | SRR1570611 |
| *Meleagris gallopavo* | SRR1570343 |
| *Meleagris gallopavo* | SRR1570533 |
| *Meleagris gallopavo* | SRR1570301 |
| *Meleagris gallopavo* | SRR1570355 |
| *Meleagris gallopavo* | SRR1570660 |
| *Meleagris gallopavo* | SRR1661449 |
| *Meleagris gallopavo* | SRR1570529 |
| *Meleagris gallopavo* | SRR1570336 |
| *Meleagris gallopavo* | SRR1570280 |
| *Meleagris gallopavo* | SRR1570344 |
| *Meleagris gallopavo* | SRR1732272 |
| *Meleagris gallopavo* | SRR1570334 |
| *Meleagris gallopavo* | SRR1570374 |
| *Meleagris gallopavo* | SRR1570596 |
| *Meleagris gallopavo* | SRR1570609 |
| *Meleagris gallopavo* | SRR1570577 |
| *Meleagris gallopavo* | SRR1570276 |
| *Meleagris gallopavo* | SRR1570268 |
| *Meleagris gallopavo* | SRR1570353 |
| *Meleagris gallopavo* | SRR1570602 |
| *Meleagris gallopavo* | SRR1570585 |
| *Meleagris gallopavo* | SRR1570722 |
| *Meleagris gallopavo* | SRR1570284 |
| *Meleagris gallopavo* | SRR1570524 |
| *Meleagris gallopavo* | SRR1570574 |
| *Meleagris gallopavo* | SRR1570714 |
| *Meleagris gallopavo* | SRR1570305 |
| *Meleagris gallopavo* | SRR1570310 |
| *Meleagris gallopavo* | SRR1570271 |
| *Meleagris gallopavo* | SRR1570717 |
| *Meleagris gallopavo* | SRR1570698 |
| *Meleagris gallopavo* | SRR1570243 |
| *Meleagris gallopavo* | SRR1570399 |
| *Meleagris gallopavo* | SRR1570339 |
| *Meleagris gallopavo* | SRR1570592 |
| *Meleagris gallopavo* | SRR1570604 |
| *Meleagris gallopavo* | SRR1570313 |
| *Meleagris gallopavo* | SRR1661453 |
| *Meleagris gallopavo* | SRR1570459 |
| *Meleagris gallopavo* | SRR1570213 |
| *Meleagris gallopavo* | SRR1661438 |
| *Meleagris gallopavo* | SRR1570434 |
| *Meleagris gallopavo* | SRR1570367 |
| *Meleagris gallopavo* | SRR1570540 |
| *Meleagris gallopavo* | SRR1570469 |
| *Meleagris gallopavo* | SRR1570723 |
| *Meleagris gallopavo* | SRR1570290 |
| *Meleagris gallopavo* | SRR1570322 |
| *Meleagris gallopavo* | SRR1570661 |
| *Meleagris gallopavo* | SRR1570485 |
| *Meleagris gallopavo* | SRR1570696 |
| *Meleagris gallopavo* | SRR1570659 |
| *Meleagris gallopavo* | SRR1570383 |
| *Meleagris gallopavo* | SRR1570291 |
| *Meleagris gallopavo* | SRR1570436 |
| *Meleagris gallopavo* | SRR1570366 |
| *Meleagris gallopavo* | SRR1570538 |
| *Meleagris gallopavo* | SRR1570541 |
| *Meleagris gallopavo* | SRR1570224 |
| *Meleagris gallopavo* | SRR1570392 |
| *Meleagris gallopavo* | SRR1570521 |
| *Meleagris gallopavo* | SRR1570263 |
| *Meleagris gallopavo* | SRR1570398 |
| *Meleagris gallopavo* | SRR1570662 |
| *Meleagris gallopavo* | SRR1570319 |
| *Meleagris gallopavo* | SRR1570279 |
| *Meleagris gallopavo* | SRR1570576 |
| *Meleagris gallopavo* | SRR1570539 |
| *Meleagris gallopavo* | SRR1661421 |
| *Meleagris gallopavo* | SRR1570527 |
| *Meleagris gallopavo* | SRR1570333 |
| *Meleagris gallopavo* | SRR1570330 |
| *Meleagris gallopavo* | SRR1570255 |
| *Meleagris gallopavo* | SRR1570706 |
| *Meleagris gallopavo* | SRR1570715 |
| *Meleagris gallopavo* | SRR1570300 |
| *Meleagris gallopavo* | SRR1570612 |
| *Meleagris gallopavo* | SRR1570347 |
| *Meleagris gallopavo* | SRR1570307 |
| *Meleagris gallopavo* | SRR1570558 |
| *Meleagris gallopavo* | SRR1570260 |
| *Meleagris gallopavo* | SRR1570731 |
| *Meleagris gallopavo* | SRR1570308 |
| *Meleagris gallopavo* | SRR1570462 |
| *Meleagris gallopavo* | SRR1570346 |
| *Meleagris gallopavo* | SRR1570444 |
| *Meleagris gallopavo* | SRR1570603 |
| *Meleagris gallopavo* | SRR1570716 |
| *Meleagris gallopavo* | SRR1570583 |
| *Meleagris gallopavo* | SRR1570727 |
| *Meleagris gallopavo* | SRR1570277 |
| *Meleagris gallopavo* | SRR1570543 |
| *Meleagris gallopavo* | SRR1570445 |
| *Meleagris gallopavo* | SRR1570530 |
| *Meleagris gallopavo* | SRR1570601 |
| *Meleagris gallopavo* | SRR1570560 |
| *Meleagris gallopavo* | SRR1661423 |
| *Meleagris gallopavo* | SRR1570734 |
| *Meleagris gallopavo* | SRR1570421 |
| *Meleagris gallopavo* | SRR1570477 |
| *Meleagris gallopavo* | SRR1570591 |
| *Meleagris gallopavo* | SRR1570710 |
| *Meleagris gallopavo* | SRR1570582 |
| *Meleagris gallopavo* | SRR1570712 |
| *Meleagris gallopavo* | SRR1570535 |
| *Meleagris gallopavo* | SRR1661440 |
| *Meleagris gallopavo* | SRR1570422 |
| *Meleagris gallopavo* | SRR1570683 |
| *Meleagris gallopavo* | SRR1570733 |
| *Meleagris gallopavo* | SRR1570568 |
| *Meleagris gallopavo* | SRR1570678 |
| *Meleagris gallopavo* | SRR1570720 |
| *Meleagris gallopavo* | SRR1570728 |
| *Meleagris gallopavo* | SRR1570272 |
| *Meleagris gallopavo* | SRR1570643 |
| *Meleagris gallopavo* | SRR1570352 |
| *Meleagris gallopavo* | SRR1570474 |
| *Meleagris gallopavo* | SRR1570407 |
| *Meleagris gallopavo* | SRR1570627 |
| *Meleagris gallopavo* | SRR1661429 |
| *Meleagris gallopavo* | SRR1661425 |
| *Meleagris gallopavo* | SRR1570486 |
| *Meleagris gallopavo* | SRR1661448 |
| *Meleagris gallopavo* | SRR1570674 |
| *Meleagris gallopavo* | SRR1570726 |
| *Meleagris gallopavo* | SRR1570299 |
| *Meleagris gallopavo* | SRR1570700 |
| *Meleagris gallopavo* | SRR1570735 |
| *Meleagris gallopavo* | SRR1570652 |
| *Meleagris gallopavo* | SRR1570289 |
| *Meleagris gallopavo* | SRR1570354 |
| *Meleagris gallopavo* | SRR1570494 |
| *Meleagris gallopavo* | SRR1570664 |
| *Meleagris gallopavo* | SRR1570736 |
| *Meleagris gallopavo* | SRR1570729 |
| *Meleagris gallopavo* | SRR1570564 |
| *Meleagris gallopavo* | SRR1570718 |
| *Meleagris gallopavo* | SRR1661446 |
| *Meleagris gallopavo* | SRR1570273 |
| *Meleagris gallopavo* | SRR1570455 |
| *Meleagris gallopavo* | SRR1570470 |
| *Meleagris gallopavo* | SRR1570454 |
| *Meleagris gallopavo* | SRR1570478 |
| *Meleagris gallopavo* | SRR1570531 |
| *Meleagris gallopavo* | SRR1570680 |
| *Meleagris gallopavo* | SRR1661461 |
| *Meleagris gallopavo* | SRR1570198 |
| *Meleagris gallopavo* | SRR1570206 |
| *Meleagris gallopavo* | SRR1570321 |
| *Meleagris gallopavo* | SRR1570450 |
| *Meleagris gallopavo* | SRR1570622 |
| *Meleagris gallopavo* | SRR1570282 |
| *Meleagris gallopavo* | SRR1570461 |
| *Meleagris gallopavo* | SRR1570466 |
| *Meleagris gallopavo* | SRR1570519 |
| *Meleagris gallopavo* | SRR1570236 |
| *Meleagris gallopavo* | SRR1570252 |
| *Meleagris gallopavo* | SRR1570575 |
| *Meleagris gallopavo* | SRR1570414 |
| *Meleagris gallopavo* | SRR1570408 |
| *Meleagris gallopavo* | SRR1570537 |
| *Meleagris gallopavo* | SRR1570437 |
| *Meleagris gallopavo* | SRR1661426 |
| *Meleagris gallopavo* | SRR1570210 |
| *Meleagris gallopavo* | SRR1570467 |
| *Meleagris gallopavo* | SRR1570721 |
| *Meleagris gallopavo* | SRR1570387 |
| *Meleagris gallopavo* | SRR1661428 |
| *Meleagris gallopavo* | SRR1570730 |
| *Meleagris gallopavo* | SRR1570325 |
| *Meleagris gallopavo* | SRR1570247 |
| *Meleagris gallopavo* | SRR1570471 |
| *Meleagris gallopavo* | SRR1570222 |
| *Meleagris gallopavo* | SRR1661441 |
| *Meleagris gallopavo* | SRR1570226 |
| *Meleagris gallopavo* | SRR1570687 |
| *Meleagris gallopavo* | SRR1570237 |
| *Meleagris gallopavo* | SRR1570415 |
| *Meleagris gallopavo* | SRR1570595 |
| *Meleagris gallopavo* | SRR1570642 |
| *Meleagris gallopavo* | SRR1570709 |
| *Meleagris gallopavo* | SRR1661427 |
| *Meleagris gallopavo* | SRR1661422 |
| *Meleagris gallopavo* | SRR1570431 |
| *Meleagris gallopavo* | SRR1570536 |
| *Meleagris gallopavo* | SRR1570267 |
| *Meleagris gallopavo* | SRR1570667 |
| *Meleagris gallopavo* | SRR1570463 |
| *Meleagris gallopavo* | SRR1570262 |
| *Meleagris gallopavo* | SRR1570446 |
| *Meleagris gallopavo* | SRR1570547 |
| *Meleagris gallopavo* | SRR1570552 |
| *Meleagris gallopavo* | SRR1570324 |
| *Meleagris gallopavo* | SRR1570335 |
| *Meleagris gallopavo* | SRR1570534 |
| *Meleagris gallopavo* | SRR1570205 |
| *Meleagris gallopavo* | SRR1570342 |
| *Meleagris gallopavo* | SRR1661462 |
| *Meleagris gallopavo* | SRR1570338 |
| *Meleagris gallopavo* | SRR1570244 |
| *Meleagris gallopavo* | SRR1570298 |
| *Meleagris gallopavo* | SRR1570278 |
| *Meleagris gallopavo* | SRR1570326 |
| *Meleagris gallopavo* | SRR1570323 |
| *Meleagris gallopavo* | SRR1570256 |
| *Meleagris gallopavo* | SRR1570668 |
| *Meleagris gallopavo* | SRR1570238 |
| *Meleagris gallopavo* | SRR1570309 |
| *Meleagris gallopavo* | SRR1570245 |
| *Meleagris gallopavo* | SRR1570348 |
| *Meleagris gallopavo* | SRR1570317 |
| *Meleagris gallopavo* | SRR1570578 |
| *Meleagris gallopavo* | SRR1570732 |
| *Meleagris gallopavo* | SRR1661444 |
| *Meleagris gallopavo* | SRR1570358 |
| *Meleagris gallopavo* | SRR1570265 |
| *Meleagris gallopavo* | SRR1570553 |
| *Meleagris gallopavo* | SRR1570681 |
| *Meleagris gallopavo* | SRR1570438 |
| *Meleagris gallopavo* | SRR1570482 |
| *Meleagris gallopavo* | SRR1570545 |
| *Meleagris gallopavo* | SRR1570380 |
| *Meleagris gallopavo* | SRR1570261 |
| *Meleagris gallopavo* | SRR1570257 |
| *Meleagris gallopavo* | SRR1570626 |
| *Meleagris gallopavo* | SRR1570410 |
| *Meleagris gallopavo* | SRR1570517 |
| *Meleagris gallopavo* | SRR1661424 |
| *Meleagris gallopavo* | SRR1570423 |
| *Meleagris gallopavo* | SRR1661432 |
| *Meleagris gallopavo* | SRR1570713 |
| *Meleagris gallopavo* | SRR1570250 |
| *Meleagris gallopavo* | SRR1570416 |
| *Meleagris gallopavo* | SRR1570229 |
| *Meleagris gallopavo* | SRR1570253 |
| *Meleagris gallopavo* | SRR1570555 |
| *Meleagris gallopavo* | SRR1570618 |
| *Meleagris gallopavo* | SRR1570588 |
| *Meleagris gallopavo* | SRR1570600 |
| *Meleagris gallopavo* | SRR1661454 |
| *Meleagris gallopavo* | SRR1570506 |
| *Meleagris gallopavo* | SRR1570625 |
| *Meleagris gallopavo* | SRR1661451 |
| *Meleagris gallopavo* | SRR1570418 |
| *Meleagris gallopavo* | SRR1570570 |
| *Meleagris gallopavo* | SRR1570406 |
| *Meleagris gallopavo* | SRR1570447 |
| *Meleagris gallopavo* | SRR1570557 |
| *Meleagris gallopavo* | SRR1661434 |
| *Meleagris gallopavo* | SRR1661460 |
| *Meleagris gallopavo* | SRR1570563 |
| *Meleagris gallopavo* | SRR1570370 |
| *Meleagris gallopavo* | SRR1570542 |
| *Meleagris gallopavo* | SRR1570270 |
| *Meleagris gallopavo* | SRR1570315 |
| *Meleagris gallopavo* | SRR1570522 |
| *Meleagris gallopavo* | SRR1570400 |
| *Meleagris gallopavo* | SRR1570645 |
| *Meleagris gallopavo* | SRR1570456 |
| *Meleagris gallopavo* | SRR1570498 |
| *Meleagris gallopavo* | SRR1570287 |
| *Meleagris gallopavo* | SRR1570593 |
| *Meleagris gallopavo* | SRR1570239 |
| *Meleagris gallopavo* | SRR1570448 |
| *Meleagris gallopavo* | SRR1570525 |
| *Meleagris gallopavo* | SRR1570691 |
| *Meleagris gallopavo* | SRR1570487 |
| *Meleagris gallopavo* | SRR1570475 |
| *Meleagris gallopavo* | SRR1570580 |
| *Meleagris gallopavo* | SRR1570695 |
| *Meleagris gallopavo* | SRR1570361 |
| *Meleagris gallopavo* | SRR1570314 |
| *Meleagris gallopavo* | SRR1570605 |
| *Meleagris gallopavo* | SRR1570235 |
| *Meleagris gallopavo* | SRR1570349 |
| *Meleagris gallopavo* | SRR1570719 |
| *Meleagris gallopavo* | SRR1570356 |
| *Meleagris gallopavo* | SRR1570484 |
| *Meleagris gallopavo* | SRR1570705 |
| *Meleagris gallopavo* | SRR1570264 |
| *Meleagris gallopavo* | SRR1570234 |
| *Meleagris gallopavo* | SRR1570460 |
| *Meleagris gallopavo* | SRR1570428 |
| *Meleagris gallopavo* | SRR1570402 |
| *Meleagris gallopavo* | SRR1570673 |
| *Meleagris gallopavo* | SRR1570697 |
| *Meleagris gallopavo* | SRR1570549 |
| *Meleagris gallopavo* | SRR1570394 |
| *Meleagris gallopavo* | SRR1570369 |
| *Meleagris gallopavo* | SRR1570363 |
| *Meleagris gallopavo* | SRR1570571 |
| *Meleagris gallopavo* | SRR1570526 |
| *Meleagris gallopavo* | SRR1570546 |
| *Meleagris gallopavo* | SRR1570464 |
| *Meleagris gallopavo* | SRR1570608 |
| *Meleagris gallopavo* | SRR1570372 |
| *Meleagris gallopavo* | SRR1570409 |
| *Meleagris gallopavo* | SRR1570671 |
| *Meleagris gallopavo* | SRR1570457 |
| *Meleagris gallopavo* | SRR1570565 |
| *Meleagris gallopavo* | SRR1570468 |
| *Meleagris gallopavo* | SRR1570544 |
| *Meleagris gallopavo* | SRR1570327 |
| *Meleagris gallopavo* | SRR1570701 |
| *Meleagris gallopavo* | SRR1570686 |
| *Meleagris gallopavo* | SRR1661439 |
| *Meleagris gallopavo* | SRR1661459 |
| *Meleagris gallopavo* | SRR1570586 |
| *Meleagris gallopavo* | SRR1570491 |
| *Meleagris gallopavo* | SRR1570246 |
| *Meleagris gallopavo* | SRR1570345 |
| *Meleagris gallopavo* | SRR1570281 |
| *Meleagris gallopavo* | SRR1570654 |
| *Meleagris gallopavo* | SRR1570655 |
| *Meleagris gallopavo* | SRR1570413 |
| *Meleagris gallopavo* | SRR1570195 |
| *Meleagris gallopavo* | SRR1570231 |
| *Meleagris gallopavo* | SRR1570648 |
| *Meleagris gallopavo* | SRR1570425 |
| *Meleagris gallopavo* | SRR1570442 |
| *Meleagris gallopavo* | SRR1570285 |
| *Meleagris gallopavo* | SRR1570702 |
| *Meleagris gallopavo* | SRR1570692 |
| *Meleagris gallopavo* | SRR1570682 |
| *Meleagris gallopavo* | SRR1570228 |
| *Meleagris gallopavo* | SRR1661456 |
| *Meleagris gallopavo* | SRR1570269 |
| *Meleagris gallopavo* | SRR1661457 |
| *Meleagris gallopavo* | SRR1570631 |
| *Meleagris gallopavo* | SRR1570675 |
| *Meleagris gallopavo* | SRR1661436 |
| *Meleagris gallopavo* | SRR1570340 |
| *Meleagris gallopavo* | SRR1570390 |
| *Meleagris gallopavo* | SRR1570440 |
| *Meleagris gallopavo* | SRR1570283 |
| *Meleagris gallopavo* | SRR1570391 |
| *Meleagris gallopavo* | SRR1570223 |
| *Meleagris gallopavo* | SRR1570689 |
| *Meleagris gallopavo* | SRR1570203 |
| *Meleagris gallopavo* | SRR1570481 |
| *Meleagris gallopavo* | SRR1570385 |
| *Meleagris gallopavo* | SRR1570332 |
| *Meleagris gallopavo* | SRR1570259 |
| *Meleagris gallopavo* | SRR1570384 |
| *Meleagris gallopavo* | SRR1570488 |
| *Meleagris gallopavo* | SRR1570551 |
| *Meleagris gallopavo* | SRR1570451 |
| *Meleagris gallopavo* | SRR1570496 |
| *Meleagris gallopavo* | SRR1570401 |
| *Meleagris gallopavo* | SRR1661433 |
| *Meleagris gallopavo* | SRR1570288 |
| *Meleagris gallopavo* | SRR1570404 |
| *Meleagris gallopavo* | SRR1570275 |
| *Meleagris gallopavo* | SRR1570458 |
| *Meleagris gallopavo* | SRR1570566 |
| *Meleagris gallopavo* | SRR1570376 |
| *Meleagris gallopavo* | SRR1570693 |
| *Meleagris gallopavo* | SRR1570657 |
| *Meleagris gallopavo* | SRR1570232 |
| *Meleagris gallopavo* | SRR1570632 |
| *Meleagris gallopavo* | SRR1570249 |
| *Meleagris gallopavo* | SRR1570656 |
| *Meleagris gallopavo* | SRR1570412 |
| *Meleagris gallopavo* | SRR1570405 |
| *Meleagris gallopavo* | SRR1570510 |
| *Meleagris gallopavo* | SRR1661458 |
| *Meleagris gallopavo* | SRR1570360 |
| *Meleagris gallopavo* | SRR1570378 |
| *Meleagris gallopavo* | SRR1570665 |
| *Meleagris gallopavo* | SRR1570556 |
| *Meleagris gallopavo* | SRR1570554 |
| *Meleagris gallopavo* | SRR1570214 |
| *Meleagris gallopavo* | SRR1570227 |
| *Meleagris gallopavo* | SRR1570212 |
| *Meleagris gallopavo* | SRR1570242 |
| *Meleagris gallopavo* | SRR1570489 |
| *Meleagris gallopavo* | SRR1570221 |
| *Meleagris gallopavo* | SRR1570562 |
| *Meleagris gallopavo* | SRR1570433 |
| *Meleagris gallopavo* | SRR1570240 |
| *Meleagris gallopavo* | SRR1570636 |
| *Meleagris gallopavo* | SRR1570688 |
| *Meleagris gallopavo* | SRR1570472 |
| *Meleagris gallopavo* | SRR1570194 |
| *Meleagris gallopavo* | SRR1570479 |
| *Meleagris gallopavo* | SRR1570694 |
| *Meleagris gallopavo* | SRR1570303 |
| *Meleagris gallopavo* | SRR1570362 |
| *Meleagris gallopavo* | SRR1570209 |
| *Meleagris gallopavo* | SRR1570639 |
| *Meleagris gallopavo* | SRR1570550 |
| *Meleagris gallopavo* | SRR1570676 |
| *Meleagris gallopavo* | SRR1570514 |
| *Meleagris gallopavo* | SRR1570629 |
| *Meleagris gallopavo* | SRR1570286 |
| *Meleagris gallopavo* | SRR1570559 |
| *Meleagris gallopavo* | SRR1570507 |
| *Meleagris gallopavo* | SRR1570393 |
| *Meleagris gallopavo* | SRR1570357 |
| *Meleagris gallopavo* | SRR1570630 |
| *Meleagris gallopavo* | SRR1570651 |
| *Meleagris gallopavo* | SRR1570441 |
| *Meleagris gallopavo* | SRR1570569 |
| *Meleagris gallopavo* | SRR1570548 |
| *Meleagris gallopavo* | SRR1661435 |
| *Meleagris gallopavo* | SRR1570452 |
| *Meleagris gallopavo* | SRR1570598 |
| *Meleagris gallopavo* | SRR1570225 |
| *Meleagris gallopavo* | SRR1570388 |
| *Meleagris gallopavo* | SRR1570637 |
| *Meleagris gallopavo* | SRR1570254 |
| *Meleagris gallopavo* | SRR1570248 |
| *Meleagris gallopavo* | SRR3084618 |
| *Meleagris gallopavo* | SRR1570200 |
| *Meleagris gallopavo* | SRR1570424 |
| *Meleagris gallopavo* | SRR1570640 |
| *Meleagris gallopavo* | SRR1570628 |
| *Meleagris gallopavo* | SRR1570207 |
| *Meleagris gallopavo* | SRR1570690 |
| *Meleagris gallopavo* | SRR1570217 |
| *Meleagris gallopavo* | SRR1570515 |
| *Meleagris gallopavo* | SRR1570426 |
| *Meleagris gallopavo* | SRR1570453 |
| *Meleagris gallopavo* | SRR1570420 |
| *Meleagris gallopavo* | SRR1570337 |
| *Meleagris gallopavo* | SRR3084614 |
| *Meleagris gallopavo* | SRR1570449 |
| *Meleagris gallopavo* | SRR1570711 |
| *Meleagris gallopavo* | SRR1570670 |
| *Meleagris gallopavo* | SRR1570649 |
| *Meleagris gallopavo* | SRR1570297 |
| *Meleagris gallopavo* | SRR1570196 |
| *Meleagris gallopavo* | SRR1570624 |
| *Meleagris gallopavo* | SRR1570443 |
| *Meleagris gallopavo* | SRR1570646 |
| *Meleagris gallopavo* | SRR478422 |
| *Meleagris gallopavo* | SRR1570617 |
| *Meleagris gallopavo* | SRR1570641 |
| *Meleagris gallopavo* | SRR478420 |
| *Meleagris gallopavo* | SRR1570685 |
| *Meleagris gallopavo* | SRR1570403 |
| *Meleagris gallopavo* | SRR1570377 |
| *Meleagris gallopavo* | SRR1570439 |
| *Meleagris gallopavo* | SRR1570623 |
| *Meleagris gallopavo* | SRR1570411 |
| *Meleagris gallopavo* | SRR1661450 |
| *Meleagris gallopavo* | SRR478421 |
| *Meleagris gallopavo* | SRR3084609 |
| *Meleagris gallopavo* | SRR1570500 |
| *Meleagris gallopavo* | SRR1661447 |
| *Meleagris gallopavo* | SRR1570368 |
| *Meleagris gallopavo* | SRR1570417 |
| *Meleagris gallopavo* | SRR1570495 |
| *Meleagris gallopavo* | SRR1570633 |
| *Meleagris gallopavo* | SRR1570508 |
| *Meleagris gallopavo* | SRR1570381 |
| *Meleagris gallopavo* | SRR1570201 |
| *Meleagris gallopavo* | SRR1570684 |
| *Meleagris gallopavo* | SRR1570501 |
| *Meleagris gallopavo* | SRR1570490 |
| *Meleagris gallopavo* | SRR478419 |
| *Meleagris gallopavo* | SRR1570499 |
| *Meleagris gallopavo* | SRR3084625 |
| *Meleagris gallopavo* | SRR1570419 |
| *Meleagris gallopavo* | SRR3084649 |
| *Meleagris gallopavo* | SRR1570567 |
| *Meleagris gallopavo* | SRR1570513 |
| *Meleagris gallopavo* | SRR3084613 |
| *Meleagris gallopavo* | SRR1570306 |
| *Meleagris gallopavo* | SRR1570572 |
| *Meleagris gallopavo* | SRR3084630 |
| *Meleagris gallopavo* | SRR3084632 |
| *Meleagris gallopavo* | SRR1570465 |
| *Meleagris gallopavo* | SRR3084611 |
| *Meleagris gallopavo* | SRR3084647 |
| *Meleagris gallopavo* | SRR1570318 |
| *Meleagris gallopavo* | SRR478415 |
| *Meleagris gallopavo* | SRR1570371 |
| *Meleagris gallopavo* | SRR1570616 |
| *Meleagris gallopavo* | SRR1570511 |
| *Meleagris gallopavo* | SRR1570241 |
| *Meleagris gallopavo* | SRR478418 |
| *Meleagris gallopavo* | SRR1570435 |
| *Meleagris gallopavo* | SRR1570208 |
| *Meleagris gallopavo* | SRR1570216 |
| *Meleagris gallopavo* | SRR3084652 |
| *Meleagris gallopavo* | SRR1570473 |
| *Meleagris gallopavo* | SRR1570379 |
| *Meleagris gallopavo* | SRR1661430 |
| *Meleagris gallopavo* | SRR1570480 |
| *Meleagris gallopavo* | SRR1570396 |
| *Meleagris gallopavo* | SRR1570430 |
| *Meleagris gallopavo* | SRR1570620 |
| *Meleagris gallopavo* | SRR3084605 |
| *Meleagris gallopavo* | SRR1570211 |
| *Meleagris gallopavo* | SRR1570365 |
| *Meleagris gallopavo* | SRR3084635 |
| *Meleagris gallopavo* | SRR1570215 |
| *Meleagris gallopavo* | SRR1570220 |
| *Meleagris gallopavo* | SRR1570197 |
| *Meleagris gallopavo* | SRR3084637 |
| *Meleagris gallopavo* | SRR1570516 |
| *Meleagris gallopavo* | SRR1570364 |
| *Meleagris gallopavo* | SRR1570663 |
| *Meleagris gallopavo* | SRR1570613 |
| *Meleagris gallopavo* | SRR1570653 |
| *Meleagris gallopavo* | SRR1570504 |
| *Meleagris gallopavo* | SRR3084634 |
| *Meleagris gallopavo* | SRR1570644 |
| *Meleagris gallopavo* | SRR1570669 |
| *Meleagris gallopavo* | SRR478417 |
| *Meleagris gallopavo* | SRR1570679 |
| *Meleagris gallopavo* | SRR3084244 |
| *Meleagris gallopavo* | SRR1570724 |
| *Meleagris gallopavo* | SRR1570509 |
| *Meleagris gallopavo* | SRR1570204 |
| *Meleagris gallopavo* | SRR1570329 |
| *Meleagris gallopavo* | SRR1570621 |
| *Meleagris gallopavo* | SRR3084247 |
| *Meleagris gallopavo* | SRR1570520 |
| *Meleagris gallopavo* | SRR3084620 |
| *Meleagris gallopavo* | SRR1570386 |
| *Meleagris gallopavo* | SRR1570493 |
| *Meleagris gallopavo* | SRR1570615 |
| *Meleagris gallopavo* | SRR3084645 |
| *Meleagris gallopavo* | SRR1570492 |
| *Meleagris gallopavo* | SRR1570427 |
| *Meleagris gallopavo* | SRR3084617 |
| *Meleagris gallopavo* | SRR3084643 |
| *Meleagris gallopavo* | SRR1570505 |
| *Meleagris gallopavo* | SRR1570503 |
| *Meleagris gallopavo* | SRR3084243 |
| *Meleagris gallopavo* | SRR3084642 |
| *Meleagris gallopavo* | SRR3084610 |
| *Meleagris gallopavo* | SRR1570497 |
| *Meleagris gallopavo* | SRR1570512 |
| *Meleagris gallopavo* | SRR1570638 |
| *Meleagris gallopavo* | SRR1570295 |
| *Meleagris gallopavo* | SRR1570502 |
| *Meleagris gallopavo* | SRR1570707 |
| *Meleagris gallopavo* | SRR1570634 |
| *Meleagris gallopavo* | SRR1570218 |
| *Meleagris gallopavo* | SRR1570619 |
| *Meleagris gallopavo* | SRR478416 |
| *Meleagris gallopavo* | SRR1570483 |
| *Meleagris gallopavo* | SRR1570266 |
| *Meleagris gallopavo* | SRR1570397 |
| *Meleagris gallopavo* | SRR1570258 |
| *Meleagris gallopavo* | SRR1570373 |
| *Meleagris gallopavo* | SRR1570677 |
| *Meleagris gallopavo* | SRR1335088 |
| *Meleagris gallopavo* | SRR1570202 |
| *Meleagris gallopavo* | SRR1570650 |
| *Meleagris gallopavo* | SRR1334841 |
| *Meleagris gallopavo* | SRR1570666 |
| *Meleagris gallopavo* | SRR1570672 |
| *Meleagris gallopavo* | SRR1796119 |
| *Meleagris gallopavo* | SRR1570199 |
| *Meleagris gallopavo* | SRR1796079 |
| *Meleagris gallopavo* | SRR1796082 |
| *Meleagris gallopavo* | SRR1796088 |
| *Meleagris gallopavo* | SRR1797814 |
| *Meleagris gallopavo* | SRR1797820 |
| *Meleagris gallopavo* | SRR1796085 |
| *Meleagris gallopavo* | SRR1796067 |
| *Meleagris gallopavo* | SRR1796084 |
| *Meleagris gallopavo* | SRR1335734 |
| *Meleagris gallopavo* | SRR1796077 |
| *Meleagris gallopavo* | SRR1796070 |
| *Meleagris gallopavo* | SRR1335730 |
| *Meleagris gallopavo* | SRR1796056 |
| *Meleagris gallopavo* | SRR1796058 |
| *Meleagris gallopavo* | SRR1797815 |
| *Meleagris gallopavo* | SRR1796090 |
| *Meleagris gallopavo* | SRR1796059 |
| *Meleagris gallopavo silvestris* | SRR3084692 |
| *Meleagris gallopavo silvestris* | SRR3084690 |
| *Meleagris gallopavo silvestris* | SRR3084689 |
| *Meleagris gallopavo silvestris* | SRR3084693 |
| *Meleagris gallopavo silvestris* | SRR3084672 |
| *Meleagris gallopavo silvestris* | SRR3084677 |
| *Meleagris gallopavo silvestris* | SRR3084675 |
| *Meleagris gallopavo silvestris* | SRR3084688 |
| *Meleagris gallopavo silvestris* | SRR3084682 |
| *Meleagris gallopavo silvestris* | SRR3084686 |
| *Meleagris gallopavo silvestris* | SRR3084684 |
| *Meleagris gallopavo silvestris* | SRR3084685 |
| *Meleagris gallopavo silvestris* | SRR3084674 |
| *Meleagris gallopavo silvestris* | SRR3084681 |
| *Meleagris gallopavo silvestris* | SRR3084678 |
| *Melopsittacus undulatus* | SRR029329 |
| *Melopsittacus undulatus* | SRR029330 |
| *Melospiza melodia* | SRR393822 |
| *Melospiza melodia* | SRR500996 |
| *Melospiza melodia* | SRR393823 |
| *Melospiza melodia* | SRR1198306 |
| *Melospiza melodia* | SRR1198308 |
| *Melospiza melodia* | SRR1198302 |
| *Melospiza melodia* | SRR1198305 |
| *Melospiza melodia* | SRR1198320 |
| *Melospiza melodia* | SRR1198303 |
| *Melospiza melodia* | SRR1198307 |
| *Nesoptilotis leucotis* | SRR3901721 |
| *Numida meleagris* | SRR828636 |
| *Numida meleagris* | SRR1213063 |
| *Numida meleagris* | SRR1210380 |
| *Numida meleagris* | SRR830283 |
| *Numida meleagris* | SRR1795827 |
| *Numida meleagris* | SRR1795854 |
| *Numida meleagris* | SRR1795855 |
| *Numida meleagris* | SRR1795852 |
| *Numida meleagris* | SRR1795837 |
| *Numida meleagris* | SRR1795845 |
| *Numida meleagris* | SRR1795831 |
| *Numida meleagris* | SRR1795997 |
| *Numida meleagris* | SRR1795998 |
| *Numida meleagris* | SRR1795843 |
| *Numida meleagris* | SRR1795841 |
| *Numida meleagris* | SRR1795996 |
| *Numida meleagris* | SRR1795847 |
| *Numida meleagris* | SRR1795993 |
| *Numida meleagris* | SRR1795839 |
| *Numida meleagris* | SRR1795849 |
| *Numida meleagris* | SRR1795995 |
| *Numida meleagris* | SRR1795836 |
| *Numida meleagris* | SRR1795834 |
| *Numida meleagris* | SRR1795832 |
| *Numida meleagris* | SRR5482427 |
| *Numida meleagris* | SRR5482420 |
| *Numida meleagris* | SRR5482415 |
| *Numida meleagris* | SRR5482419 |
| *Numida meleagris* | SRR5482428 |
| *Numida meleagris* | SRR5482406 |
| *Numida meleagris* | SRR5482412 |
| *Numida meleagris* | SRR5482414 |
| *Numida meleagris* | SRR5482407 |
| *Numida meleagris* | SRR5482411 |
| *Numida meleagris* | SRR5482416 |
| *Numida meleagris* | SRR5482413 |
| *Numida meleagris* | SRR5482421 |
| *Numida meleagris* | SRR5482410 |
| *Numida meleagris* | SRR5482404 |
| *Numida meleagris* | SRR5482418 |
| *Numida meleagris* | SRR5482400 |
| *Numida meleagris* | SRR5482429 |
| *Numida meleagris* | SRR5482424 |
| *Numida meleagris* | SRR5482423 |
| *Numida meleagris* | SRR5482403 |
| *Numida meleagris* | SRR5482408 |
| *Numida meleagris* | SRR5482426 |
| *Numida meleagris* | SRR5482417 |
| *Numida meleagris* | SRR5482405 |
| *Numida meleagris* | SRR5482402 |
| *Numida meleagris* | SRR5482401 |
| *Numida meleagris* | SRR5482425 |
| *Numida meleagris* | SRR5482409 |
| *Numida meleagris* | SRR5482422 |
| *Otus bakkamoena* | SRR3203243 |
| *Otus scops* | SRR3203230 |
| *Pandion haliaetus* | SRR3218049 |
| *Pandion haliaetus* | SRR3218043 |
| *Pandion haliaetus* | SRR3218046 |
| *Pandion haliaetus* | SRR3218048 |
| *Pandion haliaetus* | SRR3218044 |
| *Pandion haliaetus* | SRR3218045 |
| *Pandion haliaetus* | SRR3218050 |
| *Pandion haliaetus* | SRR3218047 |
| *Pandion haliaetus* | SRR3218042 |
| *Paradoxornis webbianus bulomachus* | SRR392516 |
| *Parus major* | SRR074275 |
| *Parus major* | SRR074277 |
| *Parus major* | SRR074279 |
| *Parus major* | SRR074278 |
| *Parus major* | SRR074280 |
| *Parus major* | SRR074276 |
| *Parus major* | SRR074281 |
| *Parus major* | SRR074274 |
| *Parus major* | SRR1693190 |
| *Parus major* | SRR2170833 |
| *Parus major* | SRR2170832 |
| *Parus major* | SRR2170826 |
| *Parus major* | SRR2170824 |
| *Parus major* | SRR3955377 |
| *Parus major* | SRR3955325 |
| *Parus major* | SRR3955376 |
| *Parus major* | SRR3955382 |
| *Parus major* | SRR3955381 |
| *Parus major* | SRR3955331 |
| *Parus major* | SRR3955435 |
| *Parus major* | SRR3955327 |
| *Parus major* | SRR3955326 |
| *Parus major* | SRR3955437 |
| *Parus major* | SRR3955383 |
| *Parus major* | SRR3955379 |
| *Parus major* | SRR3955333 |
| *Parus major* | SRR3955461 |
| *Parus major* | SRR3955460 |
| *Parus major* | SRR3955436 |
| *Parus major* | SRR2170825 |
| *Parus major* | SRR3955438 |
| *Parus major* | SRR3955335 |
| *Parus major* | SRR3955323 |
| *Parus major* | SRR3955373 |
| *Parus major* | SRR3955330 |
| *Parus major* | SRR3955380 |
| *Parus major* | SRR3955378 |
| *Parus major* | SRR3955329 |
| *Parus major* | SRR1847415 |
| *Parus major* | SRR1847226 |
| *Parus major* | SRR1847225 |
| *Parus major* | SRR1847315 |
| *Parus major* | SRR1847228 |
| *Parus major* | SRR1847227 |
| *Parus major* | SRR1847224 |
| *Parus major* | SRR1847223 |
| *Passer domesticus* | SRR486075 |
| *Passer domesticus* | SRR486072 |
| *Passer domesticus* | SRR486074 |
| *Patagioenas fasciata* | SRR3146199 |
| *Patagioenas fasciata* | SRR3146201 |
| *Patagioenas fasciata* | SRR3146195 |
| *Patagioenas fasciata* | SRR3146198 |
| *Patagioenas fasciata* | SRR3146194 |
| *Patagioenas fasciata* | SRR3146197 |
| *Patagioenas fasciata* | SRR3146196 |
| *Patagioenas fasciata* | SRR3146192 |
| *Patagioenas fasciata* | SRR3146193 |
| *Pavo cristatus* | SRR1797882 |
| *Pavo cristatus* | SRR1797868 |
| *Pavo cristatus* | SRR1797849 |
| *Pavo cristatus* | SRR1797860 |
| *Pavo cristatus* | SRR1797877 |
| *Pavo cristatus* | SRR1797864 |
| *Pavo cristatus* | SRR1797870 |
| *Pavo cristatus* | SRR1797873 |
| *Pavo cristatus* | SRR1797862 |
| *Pavo cristatus* | SRR1797850 |
| *Pavo cristatus* | SRR1797874 |
| *Pavo cristatus* | SRR1797880 |
| *Pavo cristatus* | SRR1797865 |
| *Pavo cristatus* | SRR1797863 |
| *Pavo cristatus* | SRR1797861 |
| *Pavo cristatus* | SRR1797876 |
| *Pavo cristatus* | SRR1797866 |
| *Pavo cristatus* | SRR1797848 |
| *Pavo cristatus* | SRR1797869 |
| *Pavo cristatus* | SRR1797859 |
| *Periparus ater* | SRR5253663 |
| *Phasianidae gen. sp.* | SRR088928 |
| *Phasianus colchicus* | SRR1797827 |
| *Phasianus colchicus* | SRR1797838 |
| *Phasianus colchicus* | SRR1797839 |
| *Phasianus colchicus* | SRR1797825 |
| *Phasianus colchicus* | SRR1797826 |
| *Phasianus colchicus* | SRR1797831 |
| *Phasianus colchicus* | SRR1797840 |
| *Phasianus colchicus* | SRR1797845 |
| *Phasianus colchicus* | SRR1797829 |
| *Phasianus colchicus* | SRR1797832 |
| *Phasianus colchicus* | SRR1797821 |
| *Phasianus colchicus* | SRR1797836 |
| *Phasianus colchicus* | SRR1797828 |
| *Phasianus colchicus* | SRR1797834 |
| *Phasianus colchicus* | SRR1797837 |
| *Phasianus colchicus* | SRR1797823 |
| *Phasianus colchicus* | SRR1797846 |
| *Phasianus colchicus* | SRR1797843 |
| *Phasianus colchicus* | SRR1797842 |
| *Phasianus colchicus* | SRR1797844 |
| *Phasianus colchicus* | SRR1797822 |
| *Phasianus colchicus* | SRR1797835 |
| *Phylloscopus trochilus* | SRR528728 |
| *Phylloscopus trochilus* | SRR529891 |
| *Phylloscopus trochilus* | SRR528727 |
| *Phylloscopus trochilus* | SRR529556 |
| *Picus canus* | SRR3203240 |
| *Platycercus eximius* | SRR3901724 |
| *Pseudopodoces humilis* | SRR768235 |
| *Pseudopodoces humilis* | SRR768237 |
| *Pseudopodoces humilis* | SRR768236 |
| *Pygoscelis adeliae* | SRR5253662 |
| *Pygoscelis papua* | SRR2980760 |
| *Sericornis frontalis* | SRR3901710 |
| *Serinus canaria* | SRR3654318 |
| *Serinus canaria* | SRR3654317 |
| *Serinus canaria* | SRR2915371 |
| *Serinus canaria* | ERR324847 |
| *Serinus canaria* | SRR2915352 |
| *Serinus canaria* | SRR2915364 |
| *Serinus canaria* | SRR2915372 |
| *Spinus spinus* | SRR3234014 |
| *Spinus spinus* | SRR3234017 |
| *Spinus spinus* | SRR3233936 |
| *Spinus spinus* | SRR3233937 |
| *Spinus spinus* | SRR3233938 |
| *Spinus spinus* | SRR3234018 |
| *Spinus spinus* | SRR3234019 |
| *Spinus spinus* | SRR3234020 |
| *Spinus spinus* | SRR3233935 |
| *Spinus spinus* | SRR1551793 |
| *Spinus spinus* | SRR3234015 |
| *Spinus spinus* | SRR3234016 |
| *Spinus spinus* | SRR1551791 |
| *Spinus spinus* | SRR3234021 |
| *Spinus spinus* | SRR1551792 |
| *Spinus spinus* | SRR1551790 |
| *Spinus spinus* | SRR1551789 |
| *Spinus spinus* | SRR1551786 |
| *Spinus spinus* | SRR1551788 |
| *Spinus spinus* | SRR1551783 |
| *Spinus spinus* | SRR1551784 |
| *Spinus spinus* | SRR1551785 |
| *Spinus spinus* | SRR1551794 |
| *Streptopelia risoria* | SRR029331 |
| *Struthio camelus* | SRR513755 |
| *Struthio camelus* | SRR1619459 |
| *Struthio camelus* | SRR493274 |
| *Struthio camelus* | SRR513126 |
| *Struthio camelus* | SRR2097530 |
| *Struthio camelus* | SRR2093956 |
| *Struthio camelus* | SRR2097529 |
| *Struthio camelus* | SRR496214 |
| *Struthio camelus* | SRR488687 |
| *Struthio camelus* | SRR496182 |
| *Struthio camelus* | SRR513096 |
| *Struthio camelus* | SRR488565 |
| *Struthio camelus* | SRR493935 |
| *Struthio camelus* | SRR504696 |
| *Struthio camelus* | SRR507781 |
| *Struthio camelus* | SRR513754 |
| *Struthio camelus* | SRR1619445 |
| *Sturnus vulgaris* | SRR3990508 |
| *Sturnus vulgaris* | SRR3990507 |
| *Taeniopygia guttata* | SRR029798 |
| *Taeniopygia guttata* | SRR029799 |
| *Taeniopygia guttata* | SRR029807 |
| *Taeniopygia guttata* | SRR029802 |
| *Taeniopygia guttata* | SRR029803 |
| *Taeniopygia guttata* | SRR029805 |
| *Taeniopygia guttata* | SRR029808 |
| *Taeniopygia guttata* | SRR029809 |
| *Taeniopygia guttata* | SRR029804 |
| *Taeniopygia guttata* | SRR029806 |
| *Taeniopygia guttata* | SRR029800 |
| *Taeniopygia guttata* | SRR029801 |
| *Taeniopygia guttata* | SRR342052 |
| *Taeniopygia guttata* | SRR064171 |
| *Taeniopygia guttata* | SRR342051 |
| *Taeniopygia guttata* | SRR342053 |
| *Taeniopygia guttata* | SRR342050 |
| *Taeniopygia guttata* | SRR064172 |
| *Taeniopygia guttata* | SRR1619467 |
| *Taeniopygia guttata* | SRR1198328 |
| *Taeniopygia guttata* | SRR1198324 |
| *Taeniopygia guttata* | SRR1198326 |
| *Taeniopygia guttata* | SRR3475928 |
| *Taeniopygia guttata* | SRR3475930 |
| *Taeniopygia guttata* | SRR3475926 |
| *Taeniopygia guttata* | SRR3475931 |
| *Taeniopygia guttata* | SRR3475927 |
| *Taeniopygia guttata* | SRR3475929 |
| *Taeniopygia guttata* | SRR2299402 |
| *Taeniopygia guttata* | SRR3207681 |
| *Taeniopygia guttata* | SRR2299403 |
| *Taeniopygia guttata* | SRR3208114 |
| *Taeniopygia guttata* | SRR3208112 |
| *Taeniopygia guttata* | SRR3208117 |
| *Taeniopygia guttata* | SRR3208115 |
| *Taeniopygia guttata* | SRR3208120 |
| *Taeniopygia guttata* | SRR3208122 |
| *Taeniopygia guttata* | SRR2299404 |
| *Taeniopygia guttata* | SRR3208121 |
| *Taeniopygia guttata* | SRR5001848 |
| *Taeniopygia guttata* | SRR3208113 |
| *Taeniopygia guttata* | SRR5001843 |
| *Taeniopygia guttata* | SRR2545946 |
| *Taeniopygia guttata* | SRR5001849 |
| *Taeniopygia guttata* | SRR5001847 |
| *Taeniopygia guttata* | SRR5001844 |
| *Taeniopygia guttata* | SRR2545952 |
| *Taeniopygia guttata* | SRR2545943 |
| *Taeniopygia guttata* | SRR2545950 |
| *Taeniopygia guttata* | SRR5001851 |
| *Taeniopygia guttata* | SRR2545951 |
| *Taeniopygia guttata* | SRR5001850 |
| *Taeniopygia guttata* | SRR2545947 |
| *Taeniopygia guttata* | SRR5001846 |
| *Taeniopygia guttata* | SRR1564480 |
| *Taeniopygia guttata* | SRR2545945 |
| *Taeniopygia guttata* | SRR2545941 |
| *Taeniopygia guttata* | SRR2545948 |
| *Taeniopygia guttata* | SRR1564481 |
| *Taeniopygia guttata* | SRR5001845 |
| *Taeniopygia guttata* | SRR2545942 |
| *Taeniopygia guttata* | SRR1564483 |
| *Taeniopygia guttata* | SRR2545944 |
| *Taeniopygia guttata* | SRR2545949 |
| *Taeniopygia guttata* | SRR1564482 |
| *Tyto longimembris* | SRR3203222 |
| *Upupa epops* | SRR3203224 |
| *Uraeginthus granatina* | SRR955502 |
| *Zonotrichia albicollis* | SRR1200897 |
| *Zonotrichia albicollis* | SRR1200836 |
| *Zonotrichia albicollis* | SRR1200896 |
| *Zonotrichia albicollis* | SRR1200734 |
| *Zonotrichia albicollis* | SRR960571 |
| *Zonotrichia albicollis* | SRR960570 |
| *Zonotrichia albicollis* | SRR960573 |
| *Zonotrichia albicollis* | SRR960572 |
| *Zonotrichia albicollis* | SRR960569 |
| *Zonotrichia albicollis* | SRR960568 |
| *Zonotrichia albicollis* | SRR1200893 |
| *Zonotrichia albicollis* | SRR1200894 |
| *Zonotrichia albicollis* | SRR1200895 |
| *Zonotrichia capensis* | SRR2891613 |
| *Zonotrichia leucophrys* | SRR1199463 |
| *Zonotrichia leucophrys gambelii* | SRR1238747 |
