# Supplementary material for: Novel hepaci- and pegi-like viruses in native Australian wildlife and non-human primates
Source: Virus Evol. 2020 Aug 20;6(2):veaa064. doi: 10.1093/ve/veaa064 (PMC7673076; doi:10.1093/ve/veaa064)
Supplement: veaa064_Supplementary_Data [file veaa064_supplementary_data.zip › Porter.Supplementary Table 4.Revised.docx]

**Supplementary Table 4.** Results of the SRA mining of primate transcriptomes (excluding *Homo sapiens*).

| **Accession** | **Organism** |
| --- | --- |
| SRR2176206 | *Pongo pygmaeus* |
| SRR2176207 | *Pongo pygmaeus* |
| SRR306792 | *Pongo pygmaeus* |
| SRR306794 | *Pongo pygmaeus* |
| SRR306791 | *Pongo pygmaeus* |
| SRR306799 | *Pongo pygmaeus* |
| SRR306795 | *Pongo pygmaeus* |
| SRR306796 | *Pongo pygmaeus* |
| SRR306797 | *Pongo pygmaeus* |
| SRR306798 | *Pongo pygmaeus* |
| SRR306793 | *Pongo pygmaeus* |
| ERR247256 | *Pongo abelii* |
| ERR247254 | *Pongo abelii* |
| ERR247255 | *Pongo abelii* |
| ERR247258 | *Pongo abelii* |
| ERR247257 | *Pongo abelii* |
| ERR050798 | *Pongo abelii* |
| ERR050796 | *Pongo abelii* |
| ERR050795 | *Pongo abelii* |
| ERR050797 | *Pongo abelii* |
| SRR3053573 | *Gorilla gorilla gorilla* |
| ERR050791 | *Gorilla gorilla gorilla* |
| ERR050793 | *Gorilla gorilla gorilla* |
| ERR050792 | *Gorilla gorilla gorilla* |
| ERR050794 | *Gorilla gorilla gorilla* |
| SRR649365 | *Gorilla gorilla* |
| SRR649367 | *Gorilla gorilla* |
| SRR649366 | *Gorilla gorilla* |
| SRR306801 | *Gorilla gorilla* |
| SRR306800 | *Gorilla gorilla* |
| SRR306809 | *Gorilla gorilla* |
| SRR306808 | *Gorilla gorilla* |
| SRR306805 | *Gorilla gorilla* |
| SRR306807 | *Gorilla gorilla* |
| SRR306802 | *Gorilla gorilla* |
| SRR306804 | *Gorilla gorilla* |
| SRR306810 | *Gorilla gorilla* |
| SRR306803 | *Gorilla gorilla* |
| SRR306806 | *Gorilla gorilla* |
| ERR218143 | *Gorilla gorilla* |
| ERR218142 | *Gorilla gorilla* |
| SRR976180 | *Gorilla gorilla* |
| SRR976178 | *Gorilla gorilla* |
| SRR976176 | *Gorilla gorilla* |
| SRR976182 | *Gorilla gorilla* |
| SRR976181 | *Gorilla gorilla* |
| SRR976179 | *Gorilla gorilla* |
| SRR976177 | *Gorilla gorilla* |
| SRR832925 | *Gorilla* |
| SRR832926 | *Gorilla* |
| SRR873628 | *Pan paniscus* |
| SRR873627 | *Pan paniscus* |
| SRR306826 | *Pan paniscus* |
| SRR873626 | *Pan paniscus* |
| SRR306828 | *Pan paniscus* |
| SRR873629 | *Pan paniscus* |
| SRR306830 | *Pan paniscus* |
| SRR306829 | *Pan paniscus* |
| SRR306833 | *Pan paniscus* |
| SRR306831 | *Pan paniscus* |
| SRR306835 | *Pan paniscus* |
| SRR306832 | *Pan paniscus* |
| SRR306834 | *Pan paniscus* |
| SRR306827 | *Pan paniscus* |
| SRR306836 | *Pan paniscus* |
| SRR306837 | *Pan paniscus* |
| SRR976175 | *Pan paniscus* |
| SRR976174 | *Pan paniscus* |
| SRR832915 | *Pan troglodytes* |
| ERR247261 | *Pan troglodytes* |
| SRR832916 | *Pan troglodytes* |
| SRR1758920 | *Pan troglodytes* |
| SRR2040589 | *Pan troglodytes* |
| SRR2040585 | *Pan troglodytes* |
| SRR2040591 | *Pan troglodytes* |
| SRR2040586 | *Pan troglodytes* |
| SRR2040588 | *Pan troglodytes* |
| SRR2040587 | *Pan troglodytes* |
| SRR1758916 | *Pan troglodytes* |
| ERR712795 | *Pan troglodytes* |
| SRR1758922 | *Pan troglodytes* |
| SRR1758933 | *Pan troglodytes* |
| SRR1758918 | *Pan troglodytes* |
| SRR2040584 | *Pan troglodytes* |
| SRR2040590 | *Pan troglodytes* |
| SRR1758917 | *Pan troglodytes* |
| SRR1602576 | *Pan troglodytes* |
| SRR1758921 | *Pan troglodytes* |
| SRR1758915 | *Pan troglodytes* |
| ERR247263 | *Pan troglodytes* |
| ERR247260 | *Pan troglodytes* |
| ERR712835 | *Pan troglodytes* |
| DRR031589 | *Pan troglodytes* |
| ERR247262 | *Pan troglodytes* |
| SRR1758919 | *Pan troglodytes* |
| DRR031590 | *Pan troglodytes* |
| SRR544868 | *Pan troglodytes* |
| SRR1758928 | *Pan troglodytes* |
| ERR712815 | *Pan troglodytes* |
| SRR2096455 | *Pan troglodytes* |
| SRR1758923 | *Pan troglodytes* |
| SRR1758927 | *Pan troglodytes* |
| SRR2096453 | *Pan troglodytes* |
| DRR031591 | *Pan troglodytes* |
| SRR544870 | *Pan troglodytes* |
| ERR247259 | *Pan troglodytes* |
| SRR2096454 | *Pan troglodytes* |
| DRR031592 | *Pan troglodytes* |
| DRR031594 | *Pan troglodytes* |
| SRR1758926 | *Pan troglodytes* |
| DRR003368 | *Pan troglodytes* |
| DRR031593 | *Pan troglodytes* |
| DRR003369 | *Pan troglodytes* |
| DRR003370 | *Pan troglodytes* |
| DRR003367 | *Pan troglodytes* |
| SRR1758929 | *Pan troglodytes* |
| SRR4012414 | *Pan troglodytes* |
| SRR4012413 | *Pan troglodytes* |
| SRR545055 | *Pan troglodytes* |
| SRR873622 | *Pan troglodytes* |
| SRR2096452 | *Pan troglodytes* |
| SRR3711187 | *Pan troglodytes* |
| SRR3711188 | *Pan troglodytes* |
| SRR1758932 | *Pan troglodytes* |
| SRR544874 | *Pan troglodytes* |
| SRR873623 | *Pan troglodytes* |
| SRR873624 | *Pan troglodytes* |
| SRR1758925 | *Pan troglodytes* |
| SRR1758931 | *Pan troglodytes* |
| SRR1758924 | *Pan troglodytes* |
| ERR712841 | *Pan troglodytes* |
| ERR042385 | *Pan troglodytes* |
| ERR712801 | *Pan troglodytes* |
| SRR306816 | *Pan troglodytes* |
| ERR712809 | *Pan troglodytes* |
| SRR306815 | *Pan troglodytes* |
| ERR712810 | *Pan troglodytes* |
| SRR306813 | *Pan troglodytes* |
| SRR1758930 | *Pan troglodytes* |
| SRR1564416 | *Pan troglodytes* |
| SRR1564417 | *Pan troglodytes* |
| ERR712821 | *Pan troglodytes* |
| ERR712829 | *Pan troglodytes* |
| ERR712838 | *Pan troglodytes* |
| ERR712814 | *Pan troglodytes* |
| ERR712836 | *Pan troglodytes* |
| ERR712830 | *Pan troglodytes* |
| SRR306814 | *Pan troglodytes* |
| ERR712796 | *Pan troglodytes* |
| ERR712808 | *Pan troglodytes* |
| ERR712798 | *Pan troglodytes* |
| SRR306820 | *Pan troglodytes* |
| ERR712812 | *Pan troglodytes* |
| ERR712811 | *Pan troglodytes* |
| ERR712816 | *Pan troglodytes* |
| ERR712802 | *Pan troglodytes* |
| ERR712834 | *Pan troglodytes* |
| ERR712818 | *Pan troglodytes* |
| ERR712807 | *Pan troglodytes* |
| ERR712828 | *Pan troglodytes* |
| SRR3711184 | *Pan troglodytes* |
| SRR786425 | *Pan troglodytes* |
| SRR306812 | *Pan troglodytes* |
| SRR3711181 | *Pan troglodytes* |
| ERR712832 | *Pan troglodytes* |
| SRR786426 | *Pan troglodytes* |
| ERR712842 | *Pan troglodytes* |
| SRR3711182 | *Pan troglodytes* |
| SRR786428 | *Pan troglodytes* |
| ERR712837 | *Pan troglodytes* |
| SRR306822 | *Pan troglodytes* |
| ERR712831 | *Pan troglodytes* |
| SRR873625 | *Pan troglodytes* |
| ERR712827 | *Pan troglodytes* |
| SRR3711183 | *Pan troglodytes* |
| SRR786427 | *Pan troglodytes* |
| SRR1952234 | *Pan troglodytes* |
| SRR306817 | *Pan troglodytes* |
| SRR306819 | *Pan troglodytes* |
| ERR712797 | *Pan troglodytes* |
| SRR306823 | *Pan troglodytes* |
| SRR364819 | *Pan troglodytes* |
| SRR357440 | *Pan troglodytes* |
| ERR712822 | *Pan troglodytes* |
| SRR357432 | *Pan troglodytes* |
| ERR712805 | *Pan troglodytes* |
| ERR712817 | *Pan troglodytes* |
| ERR712804 | *Pan troglodytes* |
| SRR4012409 | *Pan troglodytes* |
| SRR365030 | *Pan troglodytes* |
| SRR306825 | *Pan troglodytes* |
| SRR365026 | *Pan troglodytes* |
| SRR365029 | *Pan troglodytes* |
| SRR2079375 | *Pan troglodytes* |
| SRR2079382 | *Pan troglodytes* |
| SRR306821 | *Pan troglodytes* |
| SRR1952233 | *Pan troglodytes* |
| ERR712839 | *Pan troglodytes* |
| ERR712799 | *Pan troglodytes* |
| ERR712824 | *Pan troglodytes* |
| SRR357406 | *Pan troglodytes* |
| SRR2079376 | *Pan troglodytes* |
| SRR1952232 | *Pan troglodytes* |
| ERR712825 | *Pan troglodytes* |
| SRR357413 | *Pan troglodytes* |
| SRR2079372 | *Pan troglodytes* |
| ERR712813 | *Pan troglodytes* |
| SRR2079379 | *Pan troglodytes* |
| SRR2079380 | *Pan troglodytes* |
| SRR1952235 | *Pan troglodytes* |
| SRR2079381 | *Pan troglodytes* |
| ERR712803 | *Pan troglodytes* |
| SRR4012412 | *Pan troglodytes* |
| SRR2079373 | *Pan troglodytes* |
| SRR2079378 | *Pan troglodytes* |
| SRR2079377 | *Pan troglodytes* |
| SRR1952236 | *Pan troglodytes* |
| SRR2079374 | *Pan troglodytes* |
| SRR4012406 | *Pan troglodytes* |
| SRR4012407 | *Pan troglodytes* |
| ERR712819 | *Pan troglodytes* |
| SRR306811 | *Pan troglodytes* |
| ERR712833 | *Pan troglodytes* |
| SRR4012404 | *Pan troglodytes* |
| SRR4012405 | *Pan troglodytes* |
| SRR306818 | *Pan troglodytes* |
| ERR712823 | *Pan troglodytes* |
| SRR306824 | *Pan troglodytes* |
| SRR2079371 | *Pan troglodytes* |
| SRR1510174 | *Pan troglodytes* |
| SRR945301 | *Pan troglodytes* |
| SRR2079370 | *Pan troglodytes* |
| SRR945251 | *Pan troglodytes* |
| SRR1510187 | *Pan troglodytes* |
| SRR949571 | *Pan troglodytes* |
| SRR2815938 | *Pan troglodytes* |
| SRR949569 | *Pan troglodytes* |
| SRR945253 | *Pan troglodytes* |
| SRR945303 | *Pan troglodytes* |
| SRR1510182 | *Pan troglodytes* |
| SRR945250 | *Pan troglodytes* |
| SRR945295 | *Pan troglodytes* |
| SRR949572 | *Pan troglodytes* |
| SRR1510164 | *Pan troglodytes* |
| SRR949565 | *Pan troglodytes* |
| SRR4012408 | *Pan troglodytes* |
| SRR1510189 | *Pan troglodytes* |
| SRR1510192 | *Pan troglodytes* |
| SRR1510169 | *Pan troglodytes* |
| SRR945283 | *Pan troglodytes* |
| SRR945265 | *Pan troglodytes* |
| SRR2815940 | *Pan troglodytes* |
| SRR949570 | *Pan troglodytes* |
| SRR949566 | *Pan troglodytes* |
| SRR2815919 | *Pan troglodytes* |
| SRR945252 | *Pan troglodytes* |
| SRR2815921 | *Pan troglodytes* |
| SRR945271 | *Pan troglodytes* |
| SRR1510178 | *Pan troglodytes* |
| SRR1510179 | *Pan troglodytes* |
| SRR1510162 | *Pan troglodytes* |
| SRR949564 | *Pan troglodytes* |
| SRR2815929 | *Pan troglodytes* |
| SRR2815951 | *Pan troglodytes* |
| SRR1510159 | *Pan troglodytes* |
| SRR945289 | *Pan troglodytes* |
| SRR945299 | *Pan troglodytes* |
| SRR2815931 | *Pan troglodytes* |
| SRR945255 | *Pan troglodytes* |
| SRR1510180 | *Pan troglodytes* |
| SRR945263 | *Pan troglodytes* |
| SRR945307 | *Pan troglodytes* |
| SRR949563 | *Pan troglodytes* |
| SRR2815933 | *Pan troglodytes* |
| SRR2815949 | *Pan troglodytes* |
| SRR2815923 | *Pan troglodytes* |
| SRR1510181 | *Pan troglodytes* |
| SRR2815924 | *Pan troglodytes* |
| SRR2815928 | *Pan troglodytes* |
| SRR1510160 | *Pan troglodytes* |
| SRR945279 | *Pan troglodytes* |
| SRR2815948 | *Pan troglodytes* |
| SRR945261 | *Pan troglodytes* |
| SRR2815913 | *Pan troglodytes* |
| SRR1562786 | *Pan troglodytes* |
| SRR945293 | *Pan troglodytes* |
| SRR2815930 | *Pan troglodytes* |
| SRR1510177 | *Pan troglodytes* |
| SRR2815950 | *Pan troglodytes* |
| SRR2815920 | *Pan troglodytes* |
| SRR945254 | *Pan troglodytes* |
| SRR949567 | *Pan troglodytes* |
| SRR2815922 | *Pan troglodytes* |
| SRR1562787 | *Pan troglodytes* |
| SRR2815927 | *Pan troglodytes* |
| SRR945259 | *Pan troglodytes* |
| SRR945269 | *Pan troglodytes* |
| SRR1510176 | *Pan troglodytes* |
| SRR1510196 | *Pan troglodytes* |
| SRR945287 | *Pan troglodytes* |
| SRR1510195 | *Pan troglodytes* |
| SRR2815939 | *Pan troglodytes* |
| SRR2815903 | *Pan troglodytes* |
| SRR949568 | *Pan troglodytes* |
| SRR2815925 | *Pan troglodytes* |
| SRR2815908 | *Pan troglodytes* |
| SRR2815937 | *Pan troglodytes* |
| SRR2815947 | *Pan troglodytes* |
| SRR2815926 | *Pan troglodytes* |
| SRR945260 | *Pan troglodytes* |
| SRR2815885 | *Pan troglodytes* |
| SRR2815906 | *Pan troglodytes* |
| SRR945258 | *Pan troglodytes* |
| SRR945291 | *Pan troglodytes* |
| SRR4012410 | *Pan troglodytes* |
| SRR2815886 | *Pan troglodytes* |
| SRR2815941 | *Pan troglodytes* |
| SRR1510194 | *Pan troglodytes* |
| SRR2815910 | *Pan troglodytes* |
| SRR1510167 | *Pan troglodytes* |
| SRR2815887 | *Pan troglodytes* |
| SRR2815943 | *Pan troglodytes* |
| SRR1510171 | *Pan troglodytes* |
| SRR2815944 | *Pan troglodytes* |
| SRR1562785 | *Pan troglodytes* |
| SRR2815942 | *Pan troglodytes* |
| SRR2815932 | *Pan troglodytes* |
| SRR2815905 | *Pan troglodytes* |
| SRR2815912 | *Pan troglodytes* |
| SRR2815918 | *Pan troglodytes* |
| SRR2815904 | *Pan troglodytes* |
| SRR2815911 | *Pan troglodytes* |
| SRR2815936 | *Pan troglodytes* |
| SRR2815902 | *Pan troglodytes* |
| SRR945305 | *Pan troglodytes* |
| SRR945267 | *Pan troglodytes* |
| SRR1510161 | *Pan troglodytes* |
| SRR945275 | *Pan troglodytes* |
| SRR1510184 | *Pan troglodytes* |
| SRR1510183 | *Pan troglodytes* |
| SRR2815890 | *Pan troglodytes* |
| SRR2815946 | *Pan troglodytes* |
| SRR1510158 | *Pan troglodytes* |
| SRR1510188 | *Pan troglodytes* |
| SRR1510172 | *Pan troglodytes* |
| ERR712806 | *Pan troglodytes* |
| SRR2815945 | *Pan troglodytes* |
| SRR1510170 | *Pan troglodytes* |
| SRR1562784 | *Pan troglodytes* |
| SRR2815883 | *Pan troglodytes* |
| SRR945270 | *Pan troglodytes* |
| SRR2815917 | *Pan troglodytes* |
| SRR1510186 | *Pan troglodytes* |
| SRR2815909 | *Pan troglodytes* |
| SRR2815888 | *Pan troglodytes* |
| SRR945277 | *Pan troglodytes* |
| SRR1510168 | *Pan troglodytes* |
| SRR2815897 | *Pan troglodytes* |
| SRR2815901 | *Pan troglodytes* |
| SRR945264 | *Pan troglodytes* |
| SRR1510185 | *Pan troglodytes* |
| SRR1510175 | *Pan troglodytes* |
| SRR945300 | *Pan troglodytes* |
| SRR2815896 | *Pan troglodytes* |
| SRR2815900 | *Pan troglodytes* |
| SRR1510193 | *Pan troglodytes* |
| SRR1510165 | *Pan troglodytes* |
| SRR1660282 | *Pan troglodytes* |
| SRR2815884 | *Pan troglodytes* |
| SRR945302 | *Pan troglodytes* |
| SRR2815891 | *Pan troglodytes* |
| SRR2815881 | *Pan troglodytes* |
| SRR1510173 | *Pan troglodytes* |
| SRR945257 | *Pan troglodytes* |
| SRR2815898 | *Pan troglodytes* |
| SRR2815895 | *Pan troglodytes* |
| SRR945273 | *Pan troglodytes* |
| SRR1510191 | *Pan troglodytes* |
| SRR2815894 | *Pan troglodytes* |
| SRR1510166 | *Pan troglodytes* |
| SRR2815899 | *Pan troglodytes* |
| SRR2815915 | *Pan troglodytes* |
| SRR2815935 | *Pan troglodytes* |
| SRR1510190 | *Pan troglodytes* |
| SRR945281 | *Pan troglodytes* |
| SRR2815889 | *Pan troglodytes* |
| SRR2815892 | *Pan troglodytes* |
| ERR712826 | *Pan troglodytes* |
| SRR945309 | *Pan troglodytes* |
| SRR2815907 | *Pan troglodytes* |
| SRR2815914 | *Pan troglodytes* |
| SRR945262 | *Pan troglodytes* |
| SRR2815882 | *Pan troglodytes* |
| SRR945285 | *Pan troglodytes* |
| SRR945256 | *Pan troglodytes* |
| SRR945297 | *Pan troglodytes* |
| SRR2815934 | *Pan troglodytes* |
| SRR945268 | *Pan troglodytes* |
| SRR945294 | *Pan troglodytes* |
| SRR2815893 | *Pan troglodytes* |
| SRR2815880 | *Pan troglodytes* |
| SRR1510163 | *Pan troglodytes* |
| SRR945266 | *Pan troglodytes* |
| SRR945298 | *Pan troglodytes* |
| SRR945288 | *Pan troglodytes* |
| SRR945306 | *Pan troglodytes* |
| SRR504802 | *Pan troglodytes* |
| SRR945272 | *Pan troglodytes* |
| SRR945292 | *Pan troglodytes* |
| SRR945304 | *Pan troglodytes* |
| SRR945286 | *Pan troglodytes* |
| SRR786630 | *Pan troglodytes* |
| SRR945282 | *Pan troglodytes* |
| SRR945290 | *Pan troglodytes* |
| SRR2070747 | *Pan troglodytes* |
| ERR038439 | *Pan troglodytes* |
| SRR066749 | *Pan troglodytes* |
| SRR945308 | *Pan troglodytes* |
| SRR066750 | *Pan troglodytes* |
| SRR1562773 | *Pan troglodytes* |
| SRR1562772 | *Pan troglodytes* |
| SRR1168240 | *Pan troglodytes* |
| SRR1562766 | *Pan troglodytes* |
| SRR1562783 | *Pan troglodytes* |
| SRR1562767 | *Pan troglodytes* |
| SRR1562782 | *Pan troglodytes* |
| SRR2070746 | *Pan troglodytes* |
| SRR1168238 | *Pan troglodytes* |
| SRR786632 | *Pan troglodytes* |
| SRR945278 | *Pan troglodytes* |
| SRR066748 | *Pan troglodytes* |
| SRR2070745 | *Pan troglodytes* |
| SRR945296 | *Pan troglodytes* |
| SRR1562765 | *Pan troglodytes* |
| ERR712800 | *Pan troglodytes* |
| SRR1562764 | *Pan troglodytes* |
| SRR1562776 | *Pan troglodytes* |
| SRR363722 | *Pan troglodytes* |
| SRR1562777 | *Pan troglodytes* |
| SRR786631 | *Pan troglodytes* |
| SRR945274 | *Pan troglodytes* |
| SRR1562762 | *Pan troglodytes* |
| SRR786633 | *Pan troglodytes* |
| SRR945276 | *Pan troglodytes* |
| SRR1562763 | *Pan troglodytes* |
| SRR1562775 | *Pan troglodytes* |
| SRR1562790 | *Pan troglodytes* |
| SRR1562791 | *Pan troglodytes* |
| SRR363721 | *Pan troglodytes* |
| SRR4095768 | *Pan troglodytes* |
| SRR1562774 | *Pan troglodytes* |
| SRR786628 | *Pan troglodytes* |
| SRR945280 | *Pan troglodytes* |
| SRR976207 | *Pan troglodytes* |
| ERR712840 | *Pan troglodytes* |
| SRR4095761 | *Pan troglodytes* |
| SRR1562789 | *Pan troglodytes* |
| SRR4095734 | *Pan troglodytes* |
| SRR786629 | *Pan troglodytes* |
| SRR945284 | *Pan troglodytes* |
| SRR1562761 | *Pan troglodytes* |
| SRR1562707 | *Pan troglodytes* |
| SRR2070739 | *Pan troglodytes* |
| SRR1562760 | *Pan troglodytes* |
| SRR4095725 | *Pan troglodytes* |
| SRR1168242 | *Pan troglodytes* |
| SRR1562706 | *Pan troglodytes* |
| SRR1562788 | *Pan troglodytes* |
| SRR1562710 | *Pan troglodytes* |
| SRR1562711 | *Pan troglodytes* |
| SRR4095737 | *Pan troglodytes* |
| SRR4095767 | *Pan troglodytes* |
| SRR063985 | *Pan troglodytes* |
| SRR4095736 | *Pan troglodytes* |
| SRR976208 | *Pan troglodytes* |
| SRR1562709 | *Pan troglodytes* |
| SRR4095735 | *Pan troglodytes* |
| SRR976210 | *Pan troglodytes* |
| SRR1562708 | *Pan troglodytes* |
| SRR4095726 | *Pan troglodytes* |
| SRR4095759 | *Pan troglodytes* |
| SRR4095774 | *Pan troglodytes* |
| ERR712820 | *Pan troglodytes* |
| SRR4095762 | *Pan troglodytes* |
| SRR4095732 | *Pan troglodytes* |
| SRR1562683 | *Pan troglodytes* |
| SRR4095733 | *Pan troglodytes* |
| SRR4095760 | *Pan troglodytes* |
| SRR1562689 | *Pan troglodytes* |
| SRR4095750 | *Pan troglodytes* |
| SRR1168239 | *Pan troglodytes* |
| ERR038434 | *Pan troglodytes* |
| SRR4095727 | *Pan troglodytes* |
| SRR4096032 | *Pan troglodytes* |
| SRR1562682 | *Pan troglodytes* |
| SRR4095724 | *Pan troglodytes* |
| ERR038441 | *Pan troglodytes* |
| SRR4095743 | *Pan troglodytes* |
| SRR1562688 | *Pan troglodytes* |
| ERR038442 | *Pan troglodytes* |
| SRR1562695 | *Pan troglodytes* |
| SRR1562713 | *Pan troglodytes* |
| SRR976209 | *Pan troglodytes* |
| SRR4095744 | *Pan troglodytes* |
| SRR4095949 | *Pan troglodytes* |
| SRR504801 | *Pan troglodytes* |
| SRR1562686 | *Pan troglodytes* |
| ERR038433 | *Pan troglodytes* |
| SRR1562692 | *Pan troglodytes* |
| SRR1562677 | *Pan troglodytes* |
| SRR1562687 | *Pan troglodytes* |
| SRR1562693 | *Pan troglodytes* |
| SRR4095752 | *Pan troglodytes* |
| SRR504799 | *Pan troglodytes* |
| SRR1562712 | *Pan troglodytes* |
| ERR038444 | *Pan troglodytes* |
| SRR1562694 | *Pan troglodytes* |
| SRR4096027 | *Pan troglodytes* |
| SRR504798 | *Pan troglodytes* |
| SRR4095717 | *Pan troglodytes* |
| SRR4095729 | *Pan troglodytes* |
| SRR504800 | *Pan troglodytes* |
| SRR4095773 | *Pan troglodytes* |
| ERR038443 | *Pan troglodytes* |
| SRR1562676 | *Pan troglodytes* |
| SRR1562685 | *Pan troglodytes* |
| SRR1562716 | *Pan troglodytes* |
| SRR1562698 | *Pan troglodytes* |
| SRR1562717 | *Pan troglodytes* |
| SRR4096033 | *Pan troglodytes* |
| ERR038436 | *Pan troglodytes* |
| SRR1562691 | *Pan troglodytes* |
| SRR1562699 | *Pan troglodytes* |
| SRR1562684 | *Pan troglodytes* |
| SRR1562680 | *Pan troglodytes* |
| SRR032128 | *Pan troglodytes* |
| SRR1562681 | *Pan troglodytes* |
| SRR1562690 | *Pan troglodytes* |
| SRR1562701 | *Pan troglodytes* |
| SRR4095718 | *Pan troglodytes* |
| SRR4095751 | *Pan troglodytes* |
| ERR038446 | *Pan troglodytes* |
| SRR976223 | *Pan troglodytes* |
| SRR4095942 | *Pan troglodytes* |
| SRR4095951 | *Pan troglodytes* |
| SRR032129 | *Pan troglodytes* |
| SRR1562715 | *Pan troglodytes* |
| SRR1562697 | *Pan troglodytes* |
| SRR4095985 | *Pan troglodytes* |
| ERR038437 | *Pan troglodytes* |
| SRR1562714 | *Pan troglodytes* |
| SRR1562679 | *Pan troglodytes* |
| SRR1562700 | *Pan troglodytes* |
| SRR4095754 | *Pan troglodytes* |
| SRR1562696 | *Pan troglodytes* |
| SRR4095946 | *Pan troglodytes* |
| SRR4095758 | *Pan troglodytes* |
| ERR038447 | *Pan troglodytes* |
| SRR1562678 | *Pan troglodytes* |
| SRR4095944 | *Pan troglodytes* |
| ERR038431 | *Pan troglodytes* |
| SRR976222 | *Pan troglodytes* |
| ERR038448 | *Pan troglodytes* |
| SRR1562704 | *Pan troglodytes* |
| SRR1562705 | *Pan troglodytes* |
| SRR4096028 | *Pan troglodytes* |
| SRR4095728 | *Pan troglodytes* |
| SRR032135 | *Pan troglodytes* |
| SRR4095960 | *Pan troglodytes* |
| SRR976211 | *Pan troglodytes* |
| SRR4095742 | *Pan troglodytes* |
| SRR4095745 | *Pan troglodytes* |
| SRR4095962 | *Pan troglodytes* |
| SRR4095978 | *Pan troglodytes* |
| SRR4095979 | *Pan troglodytes* |
| SRR4095753 | *Pan troglodytes* |
| SRR4095977 | *Pan troglodytes* |
| SRR4095722 | *Pan troglodytes* |
| SRR4095945 | *Pan troglodytes* |
| SRR4095941 | *Pan troglodytes* |
| SRR4095995 | *Pan troglodytes* |
| SRR4095997 | *Pan troglodytes* |
| SRR4095952 | *Pan troglodytes* |
| ERR038445 | *Pan troglodytes* |
| SRR4095741 | *Pan troglodytes* |
| SRR4095984 | *Pan troglodytes* |
| SRR4095719 | *Pan troglodytes* |
| SRR4095959 | *Pan troglodytes* |
| SRR363715 | *Pan troglodytes* |
| SRR1562703 | *Pan troglodytes* |
| SRR4095731 | *Pan troglodytes* |
| SRR4095988 | *Pan troglodytes* |
| SRR4095996 | *Pan troglodytes* |
| SRR4095716 | *Pan troglodytes* |
| SRR4095955 | *Pan troglodytes* |
| ERR038450 | *Pan troglodytes* |
| SRR1168241 | *Pan troglodytes* |
| SRR1562702 | *Pan troglodytes* |
| SRR4095720 | *Pan troglodytes* |
| SRR4095765 | *Pan troglodytes* |
| SRR4095771 | *Pan troglodytes* |
| SRR4096013 | *Pan troglodytes* |
| ERR038452 | *Pan troglodytes* |
| SRR4096039 | *Pan troglodytes* |
| SRR032137 | *Pan troglodytes* |
| SRR976220 | *Pan troglodytes* |
| SRR4095715 | *Pan troglodytes* |
| SRR4095749 | *Pan troglodytes* |
| SRR363723 | *Pan troglodytes* |
| SRR4095976 | *Pan troglodytes* |
| SRR4096038 | *Pan troglodytes* |
| SRR976224 | *Pan troglodytes* |
| SRR4095738 | *Pan troglodytes* |
| SRR4095973 | *Pan troglodytes* |
| SRR4095986 | *Pan troglodytes* |
| ERR038432 | *Pan troglodytes* |
| SRR4095746 | *Pan troglodytes* |
| SRR4096015 | *Pan troglodytes* |
| SRR4095763 | *Pan troglodytes* |
| SRR4095983 | *Pan troglodytes* |
| SRR4096029 | *Pan troglodytes* |
| SRR4096050 | *Pan troglodytes* |
| ERR038453 | *Pan troglodytes* |
| SRR4095747 | *Pan troglodytes* |
| SRR4095748 | *Pan troglodytes* |
| SRR4095713 | *Pan troglodytes* |
| SRR4096035 | *Pan troglodytes* |
| SRR4096045 | *Pan troglodytes* |
| SRR4095740 | *Pan troglodytes* |
| SRR4095756 | *Pan troglodytes* |
| SRR4095730 | *Pan troglodytes* |
| SRR4096003 | *Pan troglodytes* |
| SRR4096002 | *Pan troglodytes* |
| SRR4095739 | *Pan troglodytes* |
| SRR4095964 | *Pan troglodytes* |
| ERR038440 | *Pan troglodytes* |
| SRR976214 | *Pan troglodytes* |
| SRR4095757 | *Pan troglodytes* |
| SRR4095965 | *Pan troglodytes* |
| SRR4095994 | *Pan troglodytes* |
| SRR4096055 | *Pan troglodytes* |
| SRR032134 | *Pan troglodytes* |
| SRR4095987 | *Pan troglodytes* |
| SRR4095991 | *Pan troglodytes* |
| SRR4096026 | *Pan troglodytes* |
| SRR1168237 | *Pan troglodytes* |
| SRR4095770 | *Pan troglodytes* |
| SRR4095969 | *Pan troglodytes* |
| SRR4095972 | *Pan troglodytes* |
| SRR4095980 | *Pan troglodytes* |
| SRR4095999 | *Pan troglodytes* |
| SRR4096001 | *Pan troglodytes* |
| SRR4095950 | *Pan troglodytes* |
| SRR4095956 | *Pan troglodytes* |
| SRR4095961 | *Pan troglodytes* |
| SRR032139 | *Pan troglodytes* |
| ERR038451 | *Pan troglodytes* |
| SRR4095835 | *Pan troglodytes* |
| SRR4095958 | *Pan troglodytes* |
| SRR4096022 | *Pan troglodytes* |
| ERR038430 | *Pan troglodytes* |
| SRR4095981 | *Pan troglodytes* |
| SRR4095721 | *Pan troglodytes* |
| SRR4095772 | *Pan troglodytes* |
| SRR4095844 | *Pan troglodytes* |
| SRR4095957 | *Pan troglodytes* |
| SRR4095982 | *Pan troglodytes* |
| SRR976212 | *Pan troglodytes* |
| SRR4095971 | *Pan troglodytes* |
| SRR4096030 | *Pan troglodytes* |
| SRR4095884 | *Pan troglodytes* |
| SRR4095947 | *Pan troglodytes* |
| SRR4096056 | *Pan troglodytes* |
| SRR032138 | *Pan troglodytes* |
| SRR4095790 | *Pan troglodytes* |
| SRR4095833 | *Pan troglodytes* |
| SRR4095954 | *Pan troglodytes* |
| SRR4096021 | *Pan troglodytes* |
| SRR032130 | *Pan troglodytes* |
| SRR4096016 | *Pan troglodytes* |
| ERR038435 | *Pan troglodytes* |
| SRR4095966 | *Pan troglodytes* |
| SRR4096044 | *Pan troglodytes* |
| SRR4096037 | *Pan troglodytes* |
| SRR4096046 | *Pan troglodytes* |
| SRR032131 | *Pan troglodytes* |
| SRR4095992 | *Pan troglodytes* |
| SRR4096000 | *Pan troglodytes* |
| SRR4096024 | *Pan troglodytes* |
| ERR038438 | *Pan troglodytes* |
| SRR4095828 | *Pan troglodytes* |
| SRR4095953 | *Pan troglodytes* |
| SRR4096031 | *Pan troglodytes* |
| SRR976213 | *Pan troglodytes* |
| SRR4095832 | *Pan troglodytes* |
| SRR4095921 | *Pan troglodytes* |
| SRR4095963 | *Pan troglodytes* |
| SRR032136 | *Pan troglodytes* |
| SRR363725 | *Pan troglodytes* |
| SRR4095948 | *Pan troglodytes* |
| SRR4096014 | *Pan troglodytes* |
| SRR4096049 | *Pan troglodytes* |
| SRR4095989 | *Pan troglodytes* |
| SRR4096047 | *Pan troglodytes* |
| SRR4095823 | *Pan troglodytes* |
| SRR4096042 | *Pan troglodytes* |
| SRR4096017 | *Pan troglodytes* |
| SRR4095967 | *Pan troglodytes* |
| SRR4095993 | *Pan troglodytes* |
| SRR4096006 | *Pan troglodytes* |
| ERR038449 | *Pan troglodytes* |
| SRR4096020 | *Pan troglodytes* |
| SRR4096048 | *Pan troglodytes* |
| SRR4095876 | *Pan troglodytes* |
| SRR4096040 | *Pan troglodytes* |
| SRR4096052 | *Pan troglodytes* |
| SRR4096034 | *Pan troglodytes* |
| SRR032133 | *Pan troglodytes* |
| SRR4095998 | *Pan troglodytes* |
| SRR032132 | *Pan troglodytes* |
| SRR976221 | *Pan troglodytes* |
| SRR4095837 | *Pan troglodytes* |
| SRR4095968 | *Pan troglodytes* |
| SRR4095990 | *Pan troglodytes* |
| SRR4096012 | *Pan troglodytes* |
| SRR4095834 | *Pan troglodytes* |
| SRR4095970 | *Pan troglodytes* |
| SRR4096011 | *Pan troglodytes* |
| SRR4095791 | *Pan troglodytes* |
| SRR4096018 | *Pan troglodytes* |
| SRR4095943 | *Pan troglodytes* |
| SRR4095974 | *Pan troglodytes* |
| SRR4096009 | *Pan troglodytes* |
| SRR4095839 | *Pan troglodytes* |
| SRR4095886 | *Pan troglodytes* |
| SRR4095893 | *Pan troglodytes* |
| SRR4095822 | *Pan troglodytes* |
| SRR4096025 | *Pan troglodytes* |
| SRR4095827 | *Pan troglodytes* |
| SRR4095878 | *Pan troglodytes* |
| SRR4096004 | *Pan troglodytes* |
| SRR4096023 | *Pan troglodytes* |
| SRR4096051 | *Pan troglodytes* |
| SRR4095785 | *Pan troglodytes* |
| SRR4095817 | *Pan troglodytes* |
| SRR4095879 | *Pan troglodytes* |
| SRR4095796 | *Pan troglodytes* |
| SRR4095870 | *Pan troglodytes* |
| SRR4095924 | *Pan troglodytes* |
| SRR4096043 | *Pan troglodytes* |
| SRR4095872 | *Pan troglodytes* |
| SRR363720 | *Pan troglodytes* |
| SRR1562780 | *Pan troglodytes* |
| SRR2815916 | *Pan troglodytes* |
| SRR4095824 | *Pan troglodytes* |
| SRR4095867 | *Pan troglodytes* |
| SRR1562781 | *Pan troglodytes* |
| SRR4095862 | *Pan troglodytes* |
| SRR4095869 | *Pan troglodytes* |
| SRR4095826 | *Pan troglodytes* |
| SRR4095764 | *Pan troglodytes* |
| SRR4095782 | *Pan troglodytes* |
| SRR4095885 | *Pan troglodytes* |
| SRR4095799 | *Pan troglodytes* |
| SRR4095838 | *Pan troglodytes* |
| SRR4095840 | *Pan troglodytes* |
| SRR4095864 | *Pan troglodytes* |
| SRR4095899 | *Pan troglodytes* |
| SRR4096053 | *Pan troglodytes* |
| SRR4095792 | *Pan troglodytes* |
| SRR4095919 | *Pan troglodytes* |
| SRR4096019 | *Pan troglodytes* |
| SRR4095769 | *Pan troglodytes* |
| SRR4095780 | *Pan troglodytes* |
| SRR4095871 | *Pan troglodytes* |
| SRR4095882 | *Pan troglodytes* |
| SRR4095766 | *Pan troglodytes* |
| SRR4095777 | *Pan troglodytes* |
| SRR4095801 | *Pan troglodytes* |
| SRR4095820 | *Pan troglodytes* |
| SRR4095849 | *Pan troglodytes* |
| SRR4095888 | *Pan troglodytes* |
| SRR4095975 | *Pan troglodytes* |
| SRR4096010 | *Pan troglodytes* |
| SRR4095877 | *Pan troglodytes* |
| SRR4095906 | *Pan troglodytes* |
| SRR4095830 | *Pan troglodytes* |
| SRR4095925 | *Pan troglodytes* |
| SRR4095856 | *Pan troglodytes* |
| SRR4095902 | *Pan troglodytes* |
| SRR4095916 | *Pan troglodytes* |
| SRR4095890 | *Pan troglodytes* |
| SRR4095927 | *Pan troglodytes* |
| SRR1562779 | *Pan troglodytes* |
| SRR4095818 | *Pan troglodytes* |
| SRR4095861 | *Pan troglodytes* |
| SRR4095819 | *Pan troglodytes* |
| SRR4095848 | *Pan troglodytes* |
| SRR4095783 | *Pan troglodytes* |
| SRR4095793 | *Pan troglodytes* |
| SRR4096008 | *Pan troglodytes* |
| SRR504797 | *Pan troglodytes* |
| SRR976215 | *Pan troglodytes* |
| SRR1562778 | *Pan troglodytes* |
| SRR4095809 | *Pan troglodytes* |
| SRR4095829 | *Pan troglodytes* |
| SRR4095892 | *Pan troglodytes* |
| SRR4095918 | *Pan troglodytes* |
| SRR4095797 | *Pan troglodytes* |
| SRR4095923 | *Pan troglodytes* |
| SRR4096054 | *Pan troglodytes* |
| SRR4095778 | *Pan troglodytes* |
| SRR4095779 | *Pan troglodytes* |
| SRR4095891 | *Pan troglodytes* |
| SRR4095908 | *Pan troglodytes* |
| SRR4095851 | *Pan troglodytes* |
| SRR4095865 | *Pan troglodytes* |
| SRR4095889 | *Pan troglodytes* |
| SRR4095787 | *Pan troglodytes* |
| SRR4095812 | *Pan troglodytes* |
| SRR4095845 | *Pan troglodytes* |
| SRR4095922 | *Pan troglodytes* |
| SRR4095825 | *Pan troglodytes* |
| SRR4095901 | *Pan troglodytes* |
| SRR4095723 | *Pan troglodytes* |
| SRR4095910 | *Pan troglodytes* |
| SRR4095810 | *Pan troglodytes* |
| SRR4095816 | *Pan troglodytes* |
| SRR4095853 | *Pan troglodytes* |
| SRR4095854 | *Pan troglodytes* |
| SRR4095875 | *Pan troglodytes* |
| SRR4095920 | *Pan troglodytes* |
| SRR4095821 | *Pan troglodytes* |
| SRR4095912 | *Pan troglodytes* |
| SRR4095940 | *Pan troglodytes* |
| SRR4096036 | *Pan troglodytes* |
| SRR4095863 | *Pan troglodytes* |
| SRR4095881 | *Pan troglodytes* |
| SRR4095911 | *Pan troglodytes* |
| SRR4095775 | *Pan troglodytes* |
| SRR4095855 | *Pan troglodytes* |
| SRR4095874 | *Pan troglodytes* |
| SRR4095909 | *Pan troglodytes* |
| SRR4095935 | *Pan troglodytes* |
| SRR4095846 | *Pan troglodytes* |
| SRR4095913 | *Pan troglodytes* |
| SRR4095928 | *Pan troglodytes* |
| SRR4095850 | *Pan troglodytes* |
| SRR4095931 | *Pan troglodytes* |
| SRR4095786 | *Pan troglodytes* |
| SRR4095788 | *Pan troglodytes* |
| SRR4095815 | *Pan troglodytes* |
| SRR4095858 | *Pan troglodytes* |
| SRR4095900 | *Pan troglodytes* |
| SRR4095776 | *Pan troglodytes* |
| SRR4095895 | *Pan troglodytes* |
| SRR4095915 | *Pan troglodytes* |
| SRR363718 | *Pan troglodytes* |
| SRR4095814 | *Pan troglodytes* |
| SRR4095866 | *Pan troglodytes* |
| SRR4095898 | *Pan troglodytes* |
| SRR4095932 | *Pan troglodytes* |
| SRR4095937 | *Pan troglodytes* |
| SRR4095938 | *Pan troglodytes* |
| SRR4095887 | *Pan troglodytes* |
| SRR4095897 | *Pan troglodytes* |
| SRR4095857 | *Pan troglodytes* |
| SRR4095933 | *Pan troglodytes* |
| SRR4095794 | *Pan troglodytes* |
| SRR4095883 | *Pan troglodytes* |
| SRR4095789 | *Pan troglodytes* |
| SRR4095836 | *Pan troglodytes* |
| SRR4095852 | *Pan troglodytes* |
| SRR4095868 | *Pan troglodytes* |
| SRR4095930 | *Pan troglodytes* |
| SRR4096041 | *Pan troglodytes* |
| SRR4095813 | *Pan troglodytes* |
| SRR4095806 | *Pan troglodytes* |
| SRR4095873 | *Pan troglodytes* |
| SRR4096005 | *Pan troglodytes* |
| SRR4095795 | *Pan troglodytes* |
| SRR4095798 | *Pan troglodytes* |
| SRR4095914 | *Pan troglodytes* |
| SRR4095917 | *Pan troglodytes* |
| SRR4095894 | *Pan troglodytes* |
| SRR4095903 | *Pan troglodytes* |
| SRR4095934 | *Pan troglodytes* |
| SRR363724 | *Pan troglodytes* |
| SRR4095860 | *Pan troglodytes* |
| SRR4095896 | *Pan troglodytes* |
| SRR4095905 | *Pan troglodytes* |
| SRR4095800 | *Pan troglodytes* |
| SRR4095907 | *Pan troglodytes* |
| SRR1562770 | *Pan troglodytes* |
| SRR4095929 | *Pan troglodytes* |
| SRR1562771 | *Pan troglodytes* |
| SRR976202 | *Pan troglodytes* |
| SRR4095804 | *Pan troglodytes* |
| SRR363727 | *Pan troglodytes* |
| SRR4095714 | *Pan troglodytes* |
| SRR4095939 | *Pan troglodytes* |
| SRR4095755 | *Pan troglodytes* |
| SRR1562769 | *Pan troglodytes* |
| SRR4095841 | *Pan troglodytes* |
| SRR4095784 | *Pan troglodytes* |
| SRR4095904 | *Pan troglodytes* |
| SRR4095880 | *Pan troglodytes* |
| SRR4095843 | *Pan troglodytes* |
| SRR976205 | *Pan troglodytes* |
| SRR363716 | *Pan troglodytes* |
| SRR1562768 | *Pan troglodytes* |
| SRR4095842 | *Pan troglodytes* |
| SRR4095936 | *Pan troglodytes* |
| SRR4096007 | *Pan troglodytes* |
| SRR357434 | *Varecia variegata variegata* |
| SRR361345 | *Varecia variegata variegata* |
| SRR357444 | *Varecia variegata variegata* |
| SRR357426 | *Varecia variegata variegata* |
| SRR3131108 | *Propithecus diadema* |
| SRR3131109 | *Propithecus diadema* |
| SRR3131110 | *Propithecus diadema* |
| SRR361350 | *Propithecus coquereli* |
| SRR357427 | *Propithecus coquereli* |
| SRR357415 | *Propithecus coquereli* |
| SRR361336 | *Propithecus coquereli* |
| SRR1634169 | *Cheirogaleus medius* |
| SRR1634266 | *Cheirogaleus medius* |
| SRR1634454 | *Cheirogaleus medius* |
| SRR1634152 | *Cheirogaleus medius* |
| SRR1634149 | *Cheirogaleus medius* |
| SRR1634146 | *Cheirogaleus medius* |
| SRR1634148 | *Cheirogaleus medius* |
| SRR1634270 | *Cheirogaleus medius* |
| SRR1634147 | *Cheirogaleus medius* |
| SRR1634269 | *Cheirogaleus medius* |
| SRR1634268 | *Cheirogaleus medius* |
| SRR1634272 | *Cheirogaleus medius* |
| SRR357445 | *Eulemur mongoz* |
| SRR357441 | *Eulemur mongoz* |
| SRR361343 | *Eulemur mongoz* |
| SRR361346 | *Eulemur mongoz* |
| SRR361352 | *Eulemur coronatus* |
| SRR357443 | *Eulemur coronatus* |
| SRR361344 | *Eulemur coronatus* |
| SRR357431 | *Eulemur coronatus* |
| SRR357428 | *Daubentonia madagascariensis* |
| SRR361335 | *Daubentonia madagascariensis* |
| SRR357416 | *Daubentonia madagascariensis* |
| SRR357435 | *Daubentonia madagascariensis* |
| SRR832954 | *Lemur catta* |
| SRR832955 | *Lemur catta* |
| SRR1602584 | *Lemur catta* |
| SRR3131106 | *Indri indri* |
| SRR3131107 | *Indri indri* |
| SRR3131105 | *Indri indri* |
| SRR832933 | *Microcebus murinus* |
| SRR832934 | *Microcebus murinus* |
| SRR1758993 | *Microcebus murinus* |
| SRR1758994 | *Microcebus murinus* |
| SRR1758998 | *Microcebus murinus* |
| SRR1758997 | *Microcebus murinus* |
| SRR1759000 | *Microcebus murinus* |
| SRR1758992 | *Microcebus murinus* |
| SRR1758989 | *Microcebus murinus* |
| SRR1758996 | *Microcebus murinus* |
| SRR1759003 | *Microcebus murinus* |
| SRR1758990 | *Microcebus murinus* |
| SRR1758991 | *Microcebus murinus* |
| SRR1758995 | *Microcebus murinus* |
| SRR1759002 | *Microcebus murinus* |
| SRR1758999 | *Microcebus murinus* |
| SRR1759001 | *Microcebus murinus* |
| SRR363079 | *Galago senegalensis* |
| SRR361358 | *Galago senegalensis* |
| SRR361353 | *Galago senegalensis* |
| SRR361348 | *Galago senegalensis* |
| SRR363083 | *Nycticebus coucang* |
| SRR361360 | *Nycticebus coucang* |
| SRR361349 | *Nycticebus coucang* |
| SRR361354 | *Nycticebus coucang* |
| SRR1602580 | *Aotus lemurinus* |
| SRR1981986 | *Aotus nancymaae* |
| SRR1981980 | *Aotus nancymaae* |
| SRR1981985 | *Aotus nancymaae* |
| SRR1981979 | *Aotus nancymaae* |
| SRR1981981 | *Aotus nancymaae* |
| SRR1981977 | *Aotus nancymaae* |
| SRR1981978 | *Aotus nancymaae* |
| SRR1981994 | *Aotus nancymaae* |
| SRR1981992 | *Aotus nancymaae* |
| SRR1981990 | *Aotus nancymaae* |
| SRR1981987 | *Aotus nancymaae* |
| SRR1981991 | *Aotus nancymaae* |
| SRR1981988 | *Aotus nancymaae* |
| SRR1981989 | *Aotus nancymaae* |
| SRR2048502 | *Sapajus apella* |
| SRR2048505 | *Sapajus apella* |
| SRR1759043 | *Saimiri sciureus* |
| SRR1759038 | *Saimiri sciureus* |
| SRR1759040 | *Saimiri sciureus* |
| SRR1602586 | *Saimiri sciureus* |
| SRR1759034 | *Saimiri sciureus* |
| SRR1759033 | *Saimiri sciureus* |
| SRR1759042 | *Saimiri sciureus* |
| SRR1759044 | *Saimiri sciureus* |
| SRR1759036 | *Saimiri sciureus* |
| SRR1759039 | *Saimiri sciureus* |
| SRR1759037 | *Saimiri sciureus* |
| SRR1759035 | *Saimiri sciureus* |
| SRR1759041 | *Saimiri sciureus* |
| SRR1759045 | *Saimiri sciureus* |
| SRR1759046 | *Saimiri sciureus* |
| SRR500936 | *Saimiri boliviensis boliviensis* |
| SRR500935 | *Saimiri boliviensis boliviensis* |
| SRR500940 | *Saimiri boliviensis boliviensis* |
| SRR500939 | *Saimiri boliviensis boliviensis* |
| SRR500949 | *Saimiri boliviensis boliviensis* |
| SRR500941 | *Saimiri boliviensis boliviensis* |
| SRR500946 | *Saimiri boliviensis boliviensis* |
| SRR500938 | *Saimiri boliviensis boliviensis* |
| SRR500942 | *Saimiri boliviensis boliviensis* |
| SRR500947 | *Saimiri boliviensis boliviensis* |
| SRR500945 | *Saimiri boliviensis boliviensis* |
| SRR500948 | *Saimiri boliviensis boliviensis* |
| SRR500943 | *Saimiri boliviensis boliviensis* |
| SRR500944 | *Saimiri boliviensis boliviensis* |
| SRR500934 | *Saimiri boliviensis boliviensis* |
| SRR500937 | *Saimiri boliviensis boliviensis* |
| SRR1057507 | *Rhinopithecus roxellana* |
| SRR1300771 | *Rhinopithecus bieti* |
| SRR1300767 | *Rhinopithecus bieti* |
| SRR1300763 | *Rhinopithecus bieti* |
| SRR1300762 | *Rhinopithecus bieti* |
| SRR1300768 | *Rhinopithecus bieti* |
| SRR1300764 | *Rhinopithecus bieti* |
| SRR1300761 | *Rhinopithecus bieti* |
| SRR1300770 | *Rhinopithecus bieti* |
| SRR1300769 | *Rhinopithecus bieti* |
| SRR1300766 | *Rhinopithecus bieti* |
| SRR1300765 | *Rhinopithecus bieti* |
| SRR1300759 | *Rhinopithecus bieti* |
| SRR504496 | *Papio hamadryas* |
| SRR504490 | *Papio hamadryas* |
| SRR504486 | *Papio hamadryas* |
| SRR504484 | *Papio hamadryas* |
| SRR504494 | *Papio hamadryas* |
| SRR504488 | *Papio hamadryas* |
| SRR504508 | *Papio hamadryas* |
| SRR504502 | *Papio hamadryas* |
| SRR504512 | *Papio hamadryas* |
| SRR504498 | *Papio hamadryas* |
| SRR504506 | *Papio hamadryas* |
| SRR504492 | *Papio hamadryas* |
| SRR486116 | *Papio hamadryas* |
| SRR486117 | *Papio hamadryas* |
| SRR486119 | *Papio hamadryas* |
| SRR486115 | *Papio hamadryas* |
| SRR504504 | *Papio hamadryas* |
| SRR486112 | *Papio hamadryas* |
| SRR486121 | *Papio hamadryas* |
| SRR486120 | *Papio hamadryas* |
| SRR486113 | *Papio hamadryas* |
| SRR504500 | *Papio hamadryas* |
| SRR486114 | *Papio hamadryas* |
| SRR486111 | *Papio hamadryas* |
| SRR486118 | *Papio hamadryas* |
| SRR486110 | *Papio hamadryas* |
| SRR1282072 | *Papio cynocephalus* |
| SRR1282067 | *Papio cynocephalus* |
| SRR1282073 | *Papio cynocephalus* |
| SRR1282069 | *Papio cynocephalus* |
| SRR1282076 | *Papio cynocephalus* |
| SRR1282070 | *Papio cynocephalus* |
| SRR1282075 | *Papio cynocephalus* |
| SRR1282074 | *Papio cynocephalus* |
| SRR1282071 | *Papio cynocephalus* |
| SRR1282077 | *Papio cynocephalus* |
| SRR1282066 | *Papio cynocephalus* |
| SRR1282068 | *Papio cynocephalus* |
| SRR1688511 | *Papio cynocephalus* |
| SRR1688503 | *Papio cynocephalus* |
| SRR1688522 | *Papio cynocephalus* |
| SRR1688520 | *Papio cynocephalus* |
| SRR1688518 | *Papio cynocephalus* |
| SRR1688507 | *Papio cynocephalus* |
| SRR1688527 | *Papio cynocephalus* |
| SRR1688521 | *Papio cynocephalus* |
| SRR1688530 | *Papio cynocephalus* |
| SRR1688505 | *Papio cynocephalus* |
| SRR1688506 | *Papio cynocephalus* |
| SRR1688515 | *Papio cynocephalus* |
| SRR1688529 | *Papio cynocephalus* |
| SRR1688501 | *Papio cynocephalus* |
| SRR1688519 | *Papio cynocephalus* |
| SRR1688526 | *Papio cynocephalus* |
| SRR1688504 | *Papio cynocephalus* |
| SRR1688514 | *Papio cynocephalus* |
| SRR1688525 | *Papio cynocephalus* |
| SRR1688517 | *Papio cynocephalus* |
| SRR1688510 | *Papio cynocephalus* |
| SRR1688523 | *Papio cynocephalus* |
| SRR1688509 | *Papio cynocephalus* |
| SRR1688532 | *Papio cynocephalus* |
| SRR1688531 | *Papio cynocephalus* |
| SRR1688508 | *Papio cynocephalus* |
| SRR1688534 | *Papio cynocephalus* |
| SRR1688512 | *Papio cynocephalus* |
| SRR1688524 | *Papio cynocephalus* |
| SRR1688502 | *Papio cynocephalus* |
| SRR1688513 | *Papio cynocephalus* |
| SRR1688528 | *Papio cynocephalus* |
| SRR1688516 | *Papio cynocephalus* |
| SRR1688495 | *Papio cynocephalus* |
| SRR1688487 | *Papio cynocephalus* |
| SRR1688482 | *Papio cynocephalus* |
| SRR1688491 | *Papio cynocephalus* |
| SRR1688490 | *Papio cynocephalus* |
| SRR1688498 | *Papio cynocephalus* |
| SRR1688486 | *Papio cynocephalus* |
| SRR1688477 | *Papio cynocephalus* |
| SRR1688488 | *Papio cynocephalus* |
| SRR1688500 | *Papio cynocephalus* |
| SRR1688481 | *Papio cynocephalus* |
| SRR1688484 | *Papio cynocephalus* |
| SRR1688489 | *Papio cynocephalus* |
| SRR1688483 | *Papio cynocephalus* |
| SRR1688478 | *Papio cynocephalus* |
| SRR1688497 | *Papio cynocephalus* |
| SRR1688485 | *Papio cynocephalus* |
| SRR1688480 | *Papio cynocephalus* |
| SRR1688479 | *Papio cynocephalus* |
| SRR1688492 | *Papio cynocephalus* |
| SRR1688539 | *Papio cynocephalus* |
| SRR1688499 | *Papio cynocephalus* |
| SRR1688496 | *Papio cynocephalus* |
| SRR1688494 | *Papio cynocephalus* |
| SRR1688493 | *Papio cynocephalus* |
| SRR1688536 | *Papio cynocephalus* |
| SRR1688535 | *Papio cynocephalus* |
| SRR1688537 | *Papio cynocephalus* |
| SRR1688538 | *Papio cynocephalus* |
| SRR1688533 | *Papio cynocephalus* |
| SRR832903 | *Papio anubis* |
| SRR832905 | *Papio anubis* |
| SRR1045089 | *Papio anubis* |
| SRR1758914 | *Papio anubis* |
| SRR4015316 | *Papio anubis* |
| SRR4015337 | *Papio anubis* |
| SRR4015320 | *Papio anubis* |
| SRR4015327 | *Papio anubis* |
| SRR4015330 | *Papio anubis* |
| SRR4015315 | *Papio anubis* |
| SRR4015324 | *Papio anubis* |
| SRR4015338 | *Papio anubis* |
| SRR4015339 | *Papio anubis* |
| SRR4015336 | *Papio anubis* |
| SRR4015323 | *Papio anubis* |
| SRR4015328 | *Papio anubis* |
| SRR4015326 | *Papio anubis* |
| SRR4015321 | *Papio anubis* |
| SRR4015317 | *Papio anubis* |
| SRR4015314 | *Papio anubis* |
| SRR4015325 | *Papio anubis* |
| SRR4015319 | *Papio anubis* |
| SRR4015318 | *Papio anubis* |
| SRR4015322 | *Papio anubis* |
| SRR4015329 | *Papio anubis* |
| SRR1045085 | *Papio anubis* |
| SRR1758904 | *Papio anubis* |
| SRR4015347 | *Papio anubis* |
| SRR1041110 | *Papio anubis* |
| SRR1758901 | *Papio anubis* |
| SRR1041111 | *Papio anubis* |
| SRR1758902 | *Papio anubis* |
| SRR1045086 | *Papio anubis* |
| SRR1758909 | *Papio anubis* |
| SRR4015332 | *Papio anubis* |
| SRR4015356 | *Papio anubis* |
| SRR1041117 | *Papio anubis* |
| SRR1758913 | *Papio anubis* |
| SRR4015340 | *Papio anubis* |
| SRR4015349 | *Papio anubis* |
| SRR4015333 | *Papio anubis* |
| SRR4015335 | *Papio anubis* |
| SRR832912 | *Papio anubis* |
| SRR4015341 | *Papio anubis* |
| SRR4015343 | *Papio anubis* |
| SRR4015345 | *Papio anubis* |
| SRR4015344 | *Papio anubis* |
| SRR4015348 | *Papio anubis* |
| SRR1041113 | *Papio anubis* |
| SRR1758906 | *Papio anubis* |
| SRR4015331 | *Papio anubis* |
| SRR4015342 | *Papio anubis* |
| SRR1045087 | *Papio anubis* |
| SRR1758911 | *Papio anubis* |
| SRR1041115 | *Papio anubis* |
| SRR1758908 | *Papio anubis* |
| SRR1041112 | *Papio anubis* |
| SRR1758905 | *Papio anubis* |
| SRR1045084 | *Papio anubis* |
| SRR1758903 | *Papio anubis* |
| SRR1041116 | *Papio anubis* |
| SRR1758910 | *Papio anubis* |
| SRR1041114 | *Papio anubis* |
| SRR1758907 | *Papio anubis* |
| SRR1410036 | *Papio anubis* |
| SRR4015334 | *Papio anubis* |
| SRR4015346 | *Papio anubis* |
| SRR1041109 | *Papio anubis* |
| SRR1758900 | *Papio anubis* |
| SRR1602575 | *Papio anubis* |
| SRR1045088 | *Papio anubis* |
| SRR1758912 | *Papio anubis* |
| SRR832906 | *Papio anubis* |
| SRR832907 | *Papio anubis* |
| SRR1410037 | *Papio anubis* |
| SRR2029579 | *Macaca thibetana* |
| SRR2029581 | *Macaca thibetana* |
| SRR2029582 | *Macaca thibetana* |
| SRR832956 | *Cercocebus atys* |
| SRR832957 | *Cercocebus atys* |
| SRR1759024 | *Cercocebus atys* |
| SRR1759025 | *Cercocebus atys* |
| SRR1759026 | *Cercocebus atys* |
| SRR1759032 | *Cercocebus atys* |
| SRR1759023 | *Cercocebus atys* |
| SRR1759027 | *Cercocebus atys* |
| SRR1759030 | *Cercocebus atys* |
| SRR1759017 | *Cercocebus atys* |
| SRR1759031 | *Cercocebus atys* |
| SRR1759022 | *Cercocebus atys* |
| SRR1602585 | *Cercocebus atys* |
| SRR1759029 | *Cercocebus atys* |
| SRR1759019 | *Cercocebus atys* |
| SRR1759021 | *Cercocebus atys* |
| SRR1759018 | *Cercocebus atys* |
| SRR1759028 | *Cercocebus atys* |
| SRR1759020 | *Cercocebus atys* |
| SRR3412937 | *Cebus capucinus imitator* |
| SRR850169 | *Callithrix jacchus* |
| SRR850168 | *Callithrix jacchus* |
| SRR850167 | *Callithrix jacchus* |
| ERR636251 | *Callithrix jacchus* |
| ERR636250 | *Callithrix jacchus* |
| ERR636255 | *Callithrix jacchus* |
| ERR636249 | *Callithrix jacchus* |
| ERR636253 | *Callithrix jacchus* |
| ERR636252 | *Callithrix jacchus* |
| ERR636256 | *Callithrix jacchus* |
| ERR636257 | *Callithrix jacchus* |
| ERR636254 | *Callithrix jacchus* |
| SRR1758976 | *Callithrix jacchus* |
| SRR1758985 | *Callithrix jacchus* |
| SRR1758982 | *Callithrix jacchus* |
| SRR1758979 | *Callithrix jacchus* |
| SRR1758980 | *Callithrix jacchus* |
| SRR1758978 | *Callithrix jacchus* |
| SRR1758981 | *Callithrix jacchus* |
| SRR1758984 | *Callithrix jacchus* |
| SRR1758983 | *Callithrix jacchus* |
| SRR1758988 | *Callithrix jacchus* |
| SRR1758987 | *Callithrix jacchus* |
| SRR2148000 | *Callithrix jacchus* |
| SRR1758986 | *Callithrix jacchus* |
| SRR952783 | *Callithrix jacchus* |
| SRR1758977 | *Callithrix jacchus* |
| SRR1041908 | *Callithrix jacchus* |
| SRR2148001 | *Callithrix jacchus* |
| SRR952790 | *Callithrix jacchus* |
| SRR2148002 | *Callithrix jacchus* |
| SRR952780 | *Callithrix jacchus* |
| SRR952785 | *Callithrix jacchus* |
| SRR952789 | *Callithrix jacchus* |
| SRR1041906 | *Callithrix jacchus* |
| SRR1041909 | *Callithrix jacchus* |
| SRR952611 | *Callithrix jacchus* |
| SRR866208 | *Callithrix jacchus* |
| SRR866213 | *Callithrix jacchus* |
| SRR867043 | *Callithrix jacchus* |
| SRR1041907 | *Callithrix jacchus* |
| SRR629517 | *Callithrix jacchus* |
| SRR866209 | *Callithrix jacchus* |
| SRR629519 | *Callithrix jacchus* |
| SRR867044 | *Callithrix jacchus* |
| SRR1172198 | *Callithrix jacchus* |
| SRR629515 | *Callithrix jacchus* |
| SRR629520 | *Callithrix jacchus* |
| SRR952610 | *Callithrix jacchus* |
| SRR2147999 | *Callithrix jacchus* |
| SRR975183 | *Callithrix jacchus* |
| SRR1172192 | *Callithrix jacchus* |
| SRR1172194 | *Callithrix jacchus* |
| SRR975176 | *Callithrix jacchus* |
| SRR1172193 | *Callithrix jacchus* |
| SRR1172195 | *Callithrix jacchus* |
| SRR1172197 | *Callithrix jacchus* |
| SRR1172191 | *Callithrix jacchus* |
| SRR975174 | *Callithrix jacchus* |
| SRR1172196 | *Callithrix jacchus* |
| SRR975182 | *Callithrix jacchus* |
| SRR1172190 | *Callithrix jacchus* |
| SRR975186 | *Callithrix jacchus* |
| SRR975187 | *Callithrix jacchus* |
| ERR731523 | *Callithrix jacchus* |
| ERR731511 | *Callithrix jacchus* |
| ERR731499 | *Callithrix jacchus* |
| SRR975185 | *Callithrix jacchus* |
| SRR975179 | *Callithrix jacchus* |
| ERR731487 | *Callithrix jacchus* |
| ERR731521 | *Callithrix jacchus* |
| SRR975177 | *Callithrix jacchus* |
| ERR731509 | *Callithrix jacchus* |
| ERR731497 | *Callithrix jacchus* |
| ERR731522 | *Callithrix jacchus* |
| ERR731485 | *Callithrix jacchus* |
| ERR731510 | *Callithrix jacchus* |
| ERR731498 | *Callithrix jacchus* |
| ERR731486 | *Callithrix jacchus* |
| SRR975175 | *Callithrix jacchus* |
| SRR606728 | *Callithrix jacchus* |
| SRR975178 | *Callithrix jacchus* |
| SRR975180 | *Callithrix jacchus* |
| SRR357436 | *Callithrix jacchus* |
| SRR975181 | *Callithrix jacchus* |
| SRR357446 | *Callithrix jacchus* |
| SRR357442 | *Callithrix jacchus* |
| SRR975184 | *Callithrix jacchus* |
| SRR357421 | *Callithrix jacchus* |
| SRR606715 | *Callithrix jacchus* |
| DRR056309 | *Callithrix jacchus* |
| SRR1758438 | *Callithrix jacchus* |
| SRR606744 | *Callithrix jacchus* |
| SRR1758447 | *Callithrix jacchus* |
| SRR1758443 | *Callithrix jacchus* |
| SRR1758440 | *Callithrix jacchus* |
| SRR1758446 | *Callithrix jacchus* |
| SRR1758444 | *Callithrix jacchus* |
| SRR1758445 | *Callithrix jacchus* |
| SRR1758434 | *Callithrix jacchus* |
| SRR1758439 | *Callithrix jacchus* |
| SRR1758435 | *Callithrix jacchus* |
| SRR1758441 | *Callithrix jacchus* |
| SRR2102232 | *Callithrix jacchus* |
| SRR1758442 | *Callithrix jacchus* |
| SRR1046711 | *Chlorocebus sabaeus* |
| SRR1046709 | *Chlorocebus sabaeus* |
| SRR5275331 | *Chlorocebus sabaeus* |
| SRR1032355 | *Chlorocebus sabaeus* |
| SRR5275323 | *Chlorocebus sabaeus* |
| SRR1032432 | *Chlorocebus sabaeus* |
| SRR1032445 | *Chlorocebus sabaeus* |
| SRR1032467 | *Chlorocebus sabaeus* |
| SRR5275352 | *Chlorocebus sabaeus* |
| SRR1032459 | *Chlorocebus sabaeus* |
| SRR1032434 | *Chlorocebus sabaeus* |
| SRR1032343 | *Chlorocebus sabaeus* |
| SRR1032463 | *Chlorocebus sabaeus* |
| SRR5275325 | *Chlorocebus sabaeus* |
| SRR5275363 | *Chlorocebus sabaeus* |
| SRR1032422 | *Chlorocebus sabaeus* |
| SRR5275322 | *Chlorocebus sabaeus* |
| SRR1032469 | *Chlorocebus sabaeus* |
| SRR5275350 | *Chlorocebus sabaeus* |
| SRR1032471 | *Chlorocebus sabaeus* |
| SRR1032431 | *Chlorocebus sabaeus* |
| SRR1032360 | *Chlorocebus sabaeus* |
| SRR1032460 | *Chlorocebus sabaeus* |
| SRR1032454 | *Chlorocebus sabaeus* |
| SRR5275359 | *Chlorocebus sabaeus* |
| SRR5275368 | *Chlorocebus sabaeus* |
| SRR5275329 | *Chlorocebus sabaeus* |
| SRR1032443 | *Chlorocebus sabaeus* |
| SRR1032359 | *Chlorocebus sabaeus* |
| SRR5275344 | *Chlorocebus sabaeus* |
| SRR5275349 | *Chlorocebus sabaeus* |
| SRR5275330 | *Chlorocebus sabaeus* |
| SRR5275381 | *Chlorocebus sabaeus* |
| SRR5275365 | *Chlorocebus sabaeus* |
| SRR1032381 | *Chlorocebus sabaeus* |
| SRR5275375 | *Chlorocebus sabaeus* |
| SRR5275373 | *Chlorocebus sabaeus* |
| SRR5275328 | *Chlorocebus sabaeus* |
| SRR5275367 | *Chlorocebus sabaeus* |
| SRR5275371 | *Chlorocebus sabaeus* |
| SRR1032450 | *Chlorocebus sabaeus* |
| SRR1032462 | *Chlorocebus sabaeus* |
| SRR1032440 | *Chlorocebus sabaeus* |
| SRR1032344 | *Chlorocebus sabaeus* |
| SRR1032468 | *Chlorocebus sabaeus* |
| SRR1032461 | *Chlorocebus sabaeus* |
| SRR1032464 | *Chlorocebus sabaeus* |
| SRR1032364 | *Chlorocebus sabaeus* |
| SRR1032472 | *Chlorocebus sabaeus* |
| SRR1032435 | *Chlorocebus sabaeus* |
| SRR5275335 | *Chlorocebus sabaeus* |
| SRR1032345 | *Chlorocebus sabaeus* |
| SRR1032385 | *Chlorocebus sabaeus* |
| SRR1046710 | *Chlorocebus sabaeus* |
| SRR1032350 | *Chlorocebus sabaeus* |
| SRR5275324 | *Chlorocebus sabaeus* |
| SRR1032389 | *Chlorocebus sabaeus* |
| SRR5275369 | *Chlorocebus sabaeus* |
| SRR1032453 | *Chlorocebus sabaeus* |
| SRR5275370 | *Chlorocebus sabaeus* |
| SRR5275379 | *Chlorocebus sabaeus* |
| SRR1032377 | *Chlorocebus sabaeus* |
| SRR1032366 | *Chlorocebus sabaeus* |
| SRR5275355 | *Chlorocebus sabaeus* |
| SRR1032418 | *Chlorocebus sabaeus* |
| SRR1032421 | *Chlorocebus sabaeus* |
| SRR1032370 | *Chlorocebus sabaeus* |
| SRR1032354 | *Chlorocebus sabaeus* |
| SRR5275353 | *Chlorocebus sabaeus* |
| SRR1032414 | *Chlorocebus sabaeus* |
| SRR1032369 | *Chlorocebus sabaeus* |
| SRR5275377 | *Chlorocebus sabaeus* |
| SRR1032402 | *Chlorocebus sabaeus* |
| SRR5275364 | *Chlorocebus sabaeus* |
| SRR1032374 | *Chlorocebus sabaeus* |
| SRR5275366 | *Chlorocebus sabaeus* |
| SRR5275339 | *Chlorocebus sabaeus* |
| SRR5275376 | *Chlorocebus sabaeus* |
| SRR5275342 | *Chlorocebus sabaeus* |
| SRR1032393 | *Chlorocebus sabaeus* |
| SRR1032430 | *Chlorocebus sabaeus* |
| SRR1032456 | *Chlorocebus sabaeus* |
| SRR5275326 | *Chlorocebus sabaeus* |
| SRR5275343 | *Chlorocebus sabaeus* |
| SRR5275374 | *Chlorocebus sabaeus* |
| SRR5275346 | *Chlorocebus sabaeus* |
| SRR1032361 | *Chlorocebus sabaeus* |
| SRR1032409 | *Chlorocebus sabaeus* |
| SRR5275337 | *Chlorocebus sabaeus* |
| SRR1032404 | *Chlorocebus sabaeus* |
| SRR5275341 | *Chlorocebus sabaeus* |
| SRR5275354 | *Chlorocebus sabaeus* |
| SRR5275362 | *Chlorocebus sabaeus* |
| SRR5275334 | *Chlorocebus sabaeus* |
| SRR5275338 | *Chlorocebus sabaeus* |
| SRR1032395 | *Chlorocebus sabaeus* |
| SRR5275348 | *Chlorocebus sabaeus* |
| SRR1032399 | *Chlorocebus sabaeus* |
| SRR1032373 | *Chlorocebus sabaeus* |
| SRR1032390 | *Chlorocebus sabaeus* |
| SRR1032427 | *Chlorocebus sabaeus* |
| SRR1032391 | *Chlorocebus sabaeus* |
| SRR1046712 | *Chlorocebus sabaeus* |
| SRR1032420 | *Chlorocebus sabaeus* |
| SRR5275351 | *Chlorocebus sabaeus* |
| SRR5275340 | *Chlorocebus sabaeus* |
| SRR5275358 | *Chlorocebus sabaeus* |
| SRR1032375 | *Chlorocebus sabaeus* |
| SRR5275327 | *Chlorocebus sabaeus* |
| SRR1032348 | *Chlorocebus sabaeus* |
| SRR5275356 | *Chlorocebus sabaeus* |
| SRR1032383 | *Chlorocebus sabaeus* |
| SRR5275333 | *Chlorocebus sabaeus* |
| SRR1032387 | *Chlorocebus sabaeus* |
| SRR1032451 | *Chlorocebus sabaeus* |
| SRR1032353 | *Chlorocebus sabaeus* |
| SRR5275357 | *Chlorocebus sabaeus* |
| SRR1032379 | *Chlorocebus sabaeus* |
| SRR1032448 | *Chlorocebus sabaeus* |
| SRR5275336 | *Chlorocebus sabaeus* |
| SRR1032425 | *Chlorocebus sabaeus* |
| SRR1032349 | *Chlorocebus sabaeus* |
| SRR5275372 | *Chlorocebus sabaeus* |
| SRR5275378 | *Chlorocebus sabaeus* |
| SRR1636446 | *Chlorocebus sabaeus* |
| SRR1032351 | *Chlorocebus sabaeus* |
| SRR5275345 | *Chlorocebus sabaeus* |
| SRR1636474 | *Chlorocebus sabaeus* |
| SRR1032365 | *Chlorocebus sabaeus* |
| SRR1636406 | *Chlorocebus sabaeus* |
| SRR1636422 | *Chlorocebus sabaeus* |
| SRR1636452 | *Chlorocebus sabaeus* |
| SRR1636421 | *Chlorocebus sabaeus* |
| SRR1636418 | *Chlorocebus sabaeus* |
| SRR1032352 | *Chlorocebus sabaeus* |
| SRR1636356 | *Chlorocebus sabaeus* |
| SRR1636419 | *Chlorocebus sabaeus* |
| SRR1636472 | *Chlorocebus sabaeus* |
| SRR5275380 | *Chlorocebus sabaeus* |
| SRR1636476 | *Chlorocebus sabaeus* |
| SRR1636420 | *Chlorocebus sabaeus* |
| SRR1636443 | *Chlorocebus sabaeus* |
| SRR5275332 | *Chlorocebus sabaeus* |
| SRR5275347 | *Chlorocebus sabaeus* |
| SRR1032392 | *Chlorocebus sabaeus* |
| SRR1032405 | *Chlorocebus sabaeus* |
| SRR1636481 | *Chlorocebus sabaeus* |
| SRR1032371 | *Chlorocebus sabaeus* |
| SRR1636408 | *Chlorocebus sabaeus* |
| SRR1636407 | *Chlorocebus sabaeus* |
| SRR1636358 | *Chlorocebus sabaeus* |
| SRR5275360 | *Chlorocebus sabaeus* |
| SRR1636417 | *Chlorocebus sabaeus* |
| SRR1178508 | *Chlorocebus sabaeus* |
| SRR1636470 | *Chlorocebus sabaeus* |
| SRR1636441 | *Chlorocebus sabaeus* |
| SRR1032358 | *Chlorocebus sabaeus* |
| SRR1032376 | *Chlorocebus sabaeus* |
| SRR1636437 | *Chlorocebus sabaeus* |
| SRR1636400 | *Chlorocebus sabaeus* |
| SRR1046708 | *Chlorocebus sabaeus* |
| SRR1032415 | *Chlorocebus sabaeus* |
| SRR1636363 | *Chlorocebus sabaeus* |
| SRR1636426 | *Chlorocebus sabaeus* |
| SRR1636473 | *Chlorocebus sabaeus* |
| SRR1636433 | *Chlorocebus sabaeus* |
| SRR1178501 | *Chlorocebus sabaeus* |
| SRR1636428 | *Chlorocebus sabaeus* |
| SRR1032342 | *Chlorocebus sabaeus* |
| SRR1178506 | *Chlorocebus sabaeus* |
| SRR1636380 | *Chlorocebus sabaeus* |
| SRR1636424 | *Chlorocebus sabaeus* |
| SRR1178504 | *Chlorocebus sabaeus* |
| SRR1636415 | *Chlorocebus sabaeus* |
| SRR1636479 | *Chlorocebus sabaeus* |
| SRR1636403 | *Chlorocebus sabaeus* |
| SRR1032382 | *Chlorocebus sabaeus* |
| SRR1636416 | *Chlorocebus sabaeus* |
| SRR1636477 | *Chlorocebus sabaeus* |
| SRR1636334 | *Chlorocebus sabaeus* |
| SRR1032367 | *Chlorocebus sabaeus* |
| SRR1032406 | *Chlorocebus sabaeus* |
| SRR1636445 | *Chlorocebus sabaeus* |
| SRR1636454 | *Chlorocebus sabaeus* |
| SRR1636332 | *Chlorocebus sabaeus* |
| SRR1636354 | *Chlorocebus sabaeus* |
| SRR1636442 | *Chlorocebus sabaeus* |
| SRR1636373 | *Chlorocebus sabaeus* |
| SRR1636494 | *Chlorocebus sabaeus* |
| SRR1178505 | *Chlorocebus sabaeus* |
| SRR1636430 | *Chlorocebus sabaeus* |
| SRR1032452 | *Chlorocebus sabaeus* |
| SRR1032388 | *Chlorocebus sabaeus* |
| SRR1636469 | *Chlorocebus sabaeus* |
| SRR1636374 | *Chlorocebus sabaeus* |
| SRR1636399 | *Chlorocebus sabaeus* |
| SRR5275361 | *Chlorocebus sabaeus* |
| SRR1032470 | *Chlorocebus sabaeus* |
| SRR1636370 | *Chlorocebus sabaeus* |
| SRR1032419 | *Chlorocebus sabaeus* |
| SRR1636425 | *Chlorocebus sabaeus* |
| SRR1178507 | *Chlorocebus sabaeus* |
| SRR1636402 | *Chlorocebus sabaeus* |
| SRR1636453 | *Chlorocebus sabaeus* |
| SRR1636353 | *Chlorocebus sabaeus* |
| SRR1032396 | *Chlorocebus sabaeus* |
| SRR1636439 | *Chlorocebus sabaeus* |
| SRR1032426 | *Chlorocebus sabaeus* |
| SRR1636372 | *Chlorocebus sabaeus* |
| SRR1636468 | *Chlorocebus sabaeus* |
| SRR1636365 | *Chlorocebus sabaeus* |
| SRR1178503 | *Chlorocebus sabaeus* |
| SRR1184473 | *Chlorocebus sabaeus* |
| SRR1636371 | *Chlorocebus sabaeus* |
| SRR1636361 | *Chlorocebus sabaeus* |
| SRR1636382 | *Chlorocebus sabaeus* |
| SRR1636450 | *Chlorocebus sabaeus* |
| SRR1636497 | *Chlorocebus sabaeus* |
| SRR1636492 | *Chlorocebus sabaeus* |
| SRR1636478 | *Chlorocebus sabaeus* |
| SRR1032449 | *Chlorocebus sabaeus* |
| SRR1178509 | *Chlorocebus sabaeus* |
| SRR1636330 | *Chlorocebus sabaeus* |
| SRR1032384 | *Chlorocebus sabaeus* |
| SRR1636352 | *Chlorocebus sabaeus* |
| SRR1032408 | *Chlorocebus sabaeus* |
| SRR1636401 | *Chlorocebus sabaeus* |
| SRR1636369 | *Chlorocebus sabaeus* |
| SRR1636436 | *Chlorocebus sabaeus* |
| SRR1636411 | *Chlorocebus sabaeus* |
| SRR1636378 | *Chlorocebus sabaeus* |
| SRR1636337 | *Chlorocebus sabaeus* |
| SRR1032423 | *Chlorocebus sabaeus* |
| SRR1636360 | *Chlorocebus sabaeus* |
| SRR1636435 | *Chlorocebus sabaeus* |
| SRR1636429 | *Chlorocebus sabaeus* |
| SRR1636389 | *Chlorocebus sabaeus* |
| SRR1636432 | *Chlorocebus sabaeus* |
| SRR1636379 | *Chlorocebus sabaeus* |
| SRR1636427 | *Chlorocebus sabaeus* |
| SRR1178500 | *Chlorocebus sabaeus* |
| SRR1032394 | *Chlorocebus sabaeus* |
| SRR1636395 | *Chlorocebus sabaeus* |
| SRR1636480 | *Chlorocebus sabaeus* |
| SRR1636331 | *Chlorocebus sabaeus* |
| SRR1636355 | *Chlorocebus sabaeus* |
| SRR1636457 | *Chlorocebus sabaeus* |
| SRR1636384 | *Chlorocebus sabaeus* |
| SRR1636381 | *Chlorocebus sabaeus* |
| SRR1636341 | *Chlorocebus sabaeus* |
| SRR1636483 | *Chlorocebus sabaeus* |
| SRR1636359 | *Chlorocebus sabaeus* |
| SRR1636385 | *Chlorocebus sabaeus* |
| SRR1636414 | *Chlorocebus sabaeus* |
| SRR1636364 | *Chlorocebus sabaeus* |
| SRR1636449 | *Chlorocebus sabaeus* |
| SRR1636490 | *Chlorocebus sabaeus* |
| SRR1636410 | *Chlorocebus sabaeus* |
| SRR1636387 | *Chlorocebus sabaeus* |
| SRR1032417 | *Chlorocebus sabaeus* |
| SRR1636397 | *Chlorocebus sabaeus* |
| SRR1636434 | *Chlorocebus sabaeus* |
| SRR1636413 | *Chlorocebus sabaeus* |
| SRR1636350 | *Chlorocebus sabaeus* |
| SRR1636339 | *Chlorocebus sabaeus* |
| SRR1636377 | *Chlorocebus sabaeus* |
| SRR1636376 | *Chlorocebus sabaeus* |
| SRR1636396 | *Chlorocebus sabaeus* |
| SRR1636398 | *Chlorocebus sabaeus* |
| SRR1636451 | *Chlorocebus sabaeus* |
| SRR1636329 | *Chlorocebus sabaeus* |
| SRR1032413 | *Chlorocebus sabaeus* |
| SRR1032438 | *Chlorocebus sabaeus* |
| SRR1178502 | *Chlorocebus sabaeus* |
| SRR1636368 | *Chlorocebus sabaeus* |
| SRR1636367 | *Chlorocebus sabaeus* |
| SRR1636357 | *Chlorocebus sabaeus* |
| SRR1636347 | *Chlorocebus sabaeus* |
| SRR1636485 | *Chlorocebus sabaeus* |
| SRR1032372 | *Chlorocebus sabaeus* |
| SRR1032444 | *Chlorocebus sabaeus* |
| SRR1636489 | *Chlorocebus sabaeus* |
| SRR1636440 | *Chlorocebus sabaeus* |
| SRR1636346 | *Chlorocebus sabaeus* |
| SRR1636409 | *Chlorocebus sabaeus* |
| SRR1046707 | *Chlorocebus sabaeus* |
| SRR1636362 | *Chlorocebus sabaeus* |
| SRR1636498 | *Chlorocebus sabaeus* |
| SRR1636493 | *Chlorocebus sabaeus* |
| SRR1636461 | *Chlorocebus sabaeus* |
| SRR1032397 | *Chlorocebus sabaeus* |
| SRR1636366 | *Chlorocebus sabaeus* |
| SRR1636444 | *Chlorocebus sabaeus* |
| SRR1636349 | *Chlorocebus sabaeus* |
| SRR1636393 | *Chlorocebus sabaeus* |
| SRR1636486 | *Chlorocebus sabaeus* |
| SRR1636431 | *Chlorocebus sabaeus* |
| SRR1636496 | *Chlorocebus sabaeus* |
| SRR1636383 | *Chlorocebus sabaeus* |
| SRR1636333 | *Chlorocebus sabaeus* |
| SRR1636501 | *Chlorocebus sabaeus* |
| SRR1636459 | *Chlorocebus sabaeus* |
| SRR1032398 | *Chlorocebus sabaeus* |
| SRR1636438 | *Chlorocebus sabaeus* |
| SRR1636394 | *Chlorocebus sabaeus* |
| SRR1636351 | *Chlorocebus sabaeus* |
| SRR1636390 | *Chlorocebus sabaeus* |
| SRR1636375 | *Chlorocebus sabaeus* |
| SRR1636386 | *Chlorocebus sabaeus* |
| SRR1636388 | *Chlorocebus sabaeus* |
| SRR1636327 | *Chlorocebus sabaeus* |
| SRR1636391 | *Chlorocebus sabaeus* |
| SRR1636463 | *Chlorocebus sabaeus* |
| SRR1636336 | *Chlorocebus sabaeus* |
| SRR1032386 | *Chlorocebus sabaeus* |
| SRR1636342 | *Chlorocebus sabaeus* |
| SRR1636344 | *Chlorocebus sabaeus* |
| SRR1636345 | *Chlorocebus sabaeus* |
| SRR1032439 | *Chlorocebus sabaeus* |
| SRR1636488 | *Chlorocebus sabaeus* |
| SRR1636499 | *Chlorocebus sabaeus* |
| SRR1636392 | *Chlorocebus sabaeus* |
| SRR1636348 | *Chlorocebus sabaeus* |
| SRR1636462 | *Chlorocebus sabaeus* |
| SRR1636423 | *Chlorocebus sabaeus* |
| SRR1032407 | *Chlorocebus sabaeus* |
| SRR1636505 | *Chlorocebus sabaeus* |
| SRR1636338 | *Chlorocebus sabaeus* |
| SRR1032403 | *Chlorocebus sabaeus* |
| SRR1636335 | *Chlorocebus sabaeus* |
| SRR3204337 | *Chlorocebus sabaeus* |
| SRR1636471 | *Chlorocebus sabaeus* |
| SRR1636328 | *Chlorocebus sabaeus* |
| SRR1032455 | *Chlorocebus sabaeus* |
| SRR1636448 | *Chlorocebus sabaeus* |
| SRR1636458 | *Chlorocebus sabaeus* |
| SRR3204321 | *Chlorocebus sabaeus* |
| SRR1636500 | *Chlorocebus sabaeus* |
| SRR1032490 | *Chlorocebus sabaeus* |
| SRR1032362 | *Chlorocebus sabaeus* |
| SRR1636340 | *Chlorocebus sabaeus* |
| SRR1636343 | *Chlorocebus sabaeus* |
| SRR1032346 | *Chlorocebus sabaeus* |
| SRR3204336 | *Chlorocebus sabaeus* |
| SRR3204335 | *Chlorocebus sabaeus* |
| SRR3204326 | *Chlorocebus sabaeus* |
| SRR1636456 | *Chlorocebus sabaeus* |
| SRR3204319 | *Chlorocebus sabaeus* |
| SRR1032412 | *Chlorocebus sabaeus* |
| SRR1636475 | *Chlorocebus sabaeus* |
| SRR1636466 | *Chlorocebus sabaeus* |
| SRR1636491 | *Chlorocebus sabaeus* |
| SRR1636412 | *Chlorocebus sabaeus* |
| SRR1636460 | *Chlorocebus sabaeus* |
| SRR1636465 | *Chlorocebus sabaeus* |
| SRR1032446 | *Chlorocebus sabaeus* |
| SRR3204322 | *Chlorocebus sabaeus* |
| SRR1032416 | *Chlorocebus sabaeus* |
| SRR1032433 | *Chlorocebus sabaeus* |
| SRR1032493 | *Chlorocebus sabaeus* |
| SRR1636455 | *Chlorocebus sabaeus* |
| SRR1636482 | *Chlorocebus sabaeus* |
| SRR1032356 | *Chlorocebus sabaeus* |
| SRR3204327 | *Chlorocebus sabaeus* |
| SRR1032400 | *Chlorocebus sabaeus* |
| SRR1032491 | *Chlorocebus sabaeus* |
| SRR1032492 | *Chlorocebus sabaeus* |
| SRR1032410 | *Chlorocebus sabaeus* |
| SRR1636495 | *Chlorocebus sabaeus* |
| SRR4279867 | *Chlorocebus sabaeus* |
| SRR3204342 | *Chlorocebus sabaeus* |
| SRR3204333 | *Chlorocebus sabaeus* |
| SRR1636484 | *Chlorocebus sabaeus* |
| SRR1636503 | *Chlorocebus sabaeus* |
| SRR1032378 | *Chlorocebus sabaeus* |
| SRR1636502 | *Chlorocebus sabaeus* |
| SRR1032489 | *Chlorocebus sabaeus* |
| SRR1032441 | *Chlorocebus sabaeus* |
| SRR3204331 | *Chlorocebus sabaeus* |
| SRR1636504 | *Chlorocebus sabaeus* |
| SRR1032483 | *Chlorocebus sabaeus* |
| SRR1032487 | *Chlorocebus sabaeus* |
| SRR1032457 | *Chlorocebus sabaeus* |
| SRR1032380 | *Chlorocebus sabaeus* |
| SRR1032436 | *Chlorocebus sabaeus* |
| SRR1032484 | *Chlorocebus sabaeus* |
| SRR1032488 | *Chlorocebus sabaeus* |
| SRR1032465 | *Chlorocebus sabaeus* |
| SRR1636467 | *Chlorocebus sabaeus* |
| SRR1032486 | *Chlorocebus sabaeus* |
| SRR1636487 | *Chlorocebus sabaeus* |
| SRR1032347 | *Chlorocebus sabaeus* |
| SRR1032502 | *Chlorocebus sabaeus* |
| SRR1032447 | *Chlorocebus sabaeus* |
| SRR3204343 | *Chlorocebus sabaeus* |
| SRR1636405 | *Chlorocebus sabaeus* |
| SRR1032428 | *Chlorocebus sabaeus* |
| SRR1032363 | *Chlorocebus sabaeus* |
| SRR3204334 | *Chlorocebus sabaeus* |
| SRR1032482 | *Chlorocebus sabaeus* |
| SRR1032357 | *Chlorocebus sabaeus* |
| SRR1032473 | *Chlorocebus sabaeus* |
| SRR1032505 | *Chlorocebus sabaeus* |
| SRR1636447 | *Chlorocebus sabaeus* |
| SRR1032504 | *Chlorocebus sabaeus* |
| SRR1636464 | *Chlorocebus sabaeus* |
| SRR1032503 | *Chlorocebus sabaeus* |
| SRR1636404 | *Chlorocebus sabaeus* |
| SRR3204344 | *Chlorocebus sabaeus* |
| SRR1032411 | *Chlorocebus sabaeus* |
| SRR3204340 | *Chlorocebus sabaeus* |
| SRR1032485 | *Chlorocebus sabaeus* |
| SRR1032442 | *Chlorocebus sabaeus* |
| SRR1032401 | *Chlorocebus sabaeus* |
| SRR1032458 | *Chlorocebus sabaeus* |
| SRR1032496 | *Chlorocebus sabaeus* |
| SRR3204323 | *Chlorocebus sabaeus* |
| SRR1032499 | *Chlorocebus sabaeus* |
| SRR1032501 | *Chlorocebus sabaeus* |
| SRR1032495 | *Chlorocebus sabaeus* |
| SRR1032437 | *Chlorocebus sabaeus* |
| SRR1032368 | *Chlorocebus sabaeus* |
| SRR3204320 | *Chlorocebus sabaeus* |
| SRR1032466 | *Chlorocebus sabaeus* |
| SRR3204341 | *Chlorocebus sabaeus* |
| SRR3204313 | *Chlorocebus sabaeus* |
| SRR1032500 | *Chlorocebus sabaeus* |
| SRR1032498 | *Chlorocebus sabaeus* |
| SRR3204317 | *Chlorocebus sabaeus* |
| SRR1032494 | *Chlorocebus sabaeus* |
| SRR1032474 | *Chlorocebus sabaeus* |
| SRR3204311 | *Chlorocebus sabaeus* |
| SRR1032429 | *Chlorocebus sabaeus* |
| SRR1032497 | *Chlorocebus sabaeus* |
| SRR3204310 | *Chlorocebus sabaeus* |
| SRR3204324 | *Chlorocebus sabaeus* |
| SRR1178296 | *Chlorocebus sabaeus* |
| SRR3204330 | *Chlorocebus sabaeus* |
| SRR3204328 | *Chlorocebus sabaeus* |
| SRR1032424 | *Chlorocebus sabaeus* |
| SRR3204309 | *Chlorocebus sabaeus* |
| SRR3204312 | *Chlorocebus sabaeus* |
| SRR3204338 | *Chlorocebus sabaeus* |
| SRR931093 | *Chlorocebus sabaeus* |
| SRR1178320 | *Chlorocebus sabaeus* |
| SRR931094 | *Chlorocebus sabaeus* |
| SRR1178316 | *Chlorocebus sabaeus* |
| SRR3204332 | *Chlorocebus sabaeus* |
| SRR3204315 | *Chlorocebus sabaeus* |
| SRR1178304 | *Chlorocebus sabaeus* |
| SRR1178299 | *Chlorocebus sabaeus* |
| SRR3204318 | *Chlorocebus sabaeus* |
| SRR1056199 | *Chlorocebus sabaeus* |
| SRR1178315 | *Chlorocebus sabaeus* |
| SRR1178276 | *Chlorocebus sabaeus* |
| SRR1056206 | *Chlorocebus sabaeus* |
| SRR3204339 | *Chlorocebus sabaeus* |
| SRR1178279 | *Chlorocebus sabaeus* |
| SRR1056205 | *Chlorocebus sabaeus* |
| SRR1056204 | *Chlorocebus sabaeus* |
| SRR3204325 | *Chlorocebus sabaeus* |
| SRR1056201 | *Chlorocebus sabaeus* |
| SRR1178308 | *Chlorocebus sabaeus* |
| SRR1056202 | *Chlorocebus sabaeus* |
| SRR1178284 | *Chlorocebus sabaeus* |
| SRR1178295 | *Chlorocebus sabaeus* |
| SRR1178312 | *Chlorocebus sabaeus* |
| SRR1178282 | *Chlorocebus sabaeus* |
| SRR1178300 | *Chlorocebus sabaeus* |
| SRR1178307 | *Chlorocebus sabaeus* |
| SRR1056203 | *Chlorocebus sabaeus* |
| SRR1178288 | *Chlorocebus sabaeus* |
| SRR1178319 | *Chlorocebus sabaeus* |
| SRR1178292 | *Chlorocebus sabaeus* |
| SRR1056195 | *Chlorocebus sabaeus* |
| SRR1178291 | *Chlorocebus sabaeus* |
| SRR1178275 | *Chlorocebus sabaeus* |
| SRR1056194 | *Chlorocebus sabaeus* |
| SRR1178280 | *Chlorocebus sabaeus* |
| SRR1056200 | *Chlorocebus sabaeus* |
| SRR1178283 | *Chlorocebus sabaeus* |
| SRR1178298 | *Chlorocebus sabaeus* |
| SRR1178318 | *Chlorocebus sabaeus* |
| DRR018833 | *Chlorocebus sabaeus* |
| SRR1178287 | *Chlorocebus sabaeus* |
| SRR1178303 | *Chlorocebus sabaeus* |
| SRR1178311 | *Chlorocebus sabaeus* |
| SRR3204314 | *Chlorocebus sabaeus* |
| SRR1178285 | *Chlorocebus sabaeus* |
| SRR1178286 | *Chlorocebus sabaeus* |
| SRR1178302 | *Chlorocebus sabaeus* |
| SRR1178278 | *Chlorocebus sabaeus* |
| SRR1178294 | *Chlorocebus sabaeus* |
| SRR1178317 | *Chlorocebus sabaeus* |
| DRR018835 | *Chlorocebus sabaeus* |
| SRR1178310 | *Chlorocebus sabaeus* |
| SRR1178254 | *Chlorocebus sabaeus* |
| SRR1178313 | *Chlorocebus sabaeus* |
| SRR1178306 | *Chlorocebus sabaeus* |
| SRR1919329 | *Chlorocebus sabaeus* |
| SRR1178314 | *Chlorocebus sabaeus* |
| SRR1178281 | *Chlorocebus sabaeus* |
| SRR1178277 | *Chlorocebus sabaeus* |
| SRR1919327 | *Chlorocebus sabaeus* |
| SRR1919328 | *Chlorocebus sabaeus* |
| SRR1919325 | *Chlorocebus sabaeus* |
| SRR1178309 | *Chlorocebus sabaeus* |
| SRR1178293 | *Chlorocebus sabaeus* |
| SRR1178289 | *Chlorocebus sabaeus* |
| SRR1919330 | *Chlorocebus sabaeus* |
| SRR1178297 | *Chlorocebus sabaeus* |
| SRR1056196 | *Chlorocebus sabaeus* |
| SRR1056197 | *Chlorocebus sabaeus* |
| SRR1919326 | *Chlorocebus sabaeus* |
| SRR1178305 | *Chlorocebus sabaeus* |
| SRR1178321 | *Chlorocebus sabaeus* |
| SRR1178301 | *Chlorocebus sabaeus* |
| SRR1178290 | *Chlorocebus sabaeus* |
| SRR3204316 | *Chlorocebus sabaeus* |
| SRR1056198 | *Chlorocebus sabaeus* |
| SRR3204329 | *Chlorocebus sabaeus* |
| DRR018834 | *Chlorocebus sabaeus* |
| DRR018832 | *Chlorocebus sabaeus* |
| SRR1046755 | *Chlorocebus pygerythrus* |
| SRR1046714 | *Chlorocebus pygerythrus* |
| SRR1046726 | *Chlorocebus pygerythrus* |
| SRR1046728 | *Chlorocebus pygerythrus* |
| SRR1046750 | *Chlorocebus pygerythrus* |
| SRR1046732 | *Chlorocebus pygerythrus* |
| SRR1046727 | *Chlorocebus pygerythrus* |
| SRR1046753 | *Chlorocebus pygerythrus* |
| SRR1046757 | *Chlorocebus pygerythrus* |
| SRR1046752 | *Chlorocebus pygerythrus* |
| SRR1046729 | *Chlorocebus pygerythrus* |
| SRR1046747 | *Chlorocebus pygerythrus* |
| SRR1046756 | *Chlorocebus pygerythrus* |
| SRR1046737 | *Chlorocebus pygerythrus* |
| SRR1046731 | *Chlorocebus pygerythrus* |
| SRR1046749 | *Chlorocebus pygerythrus* |
| SRR1046734 | *Chlorocebus pygerythrus* |
| SRR1046716 | *Chlorocebus pygerythrus* |
| SRR1046743 | *Chlorocebus pygerythrus* |
| SRR1046725 | *Chlorocebus pygerythrus* |
| SRR1046717 | *Chlorocebus pygerythrus* |
| SRR1046715 | *Chlorocebus pygerythrus* |
| SRR1046744 | *Chlorocebus pygerythrus* |
| SRR1046733 | *Chlorocebus pygerythrus* |
| SRR1046745 | *Chlorocebus pygerythrus* |
| SRR1046746 | *Chlorocebus pygerythrus* |
| SRR1046738 | *Chlorocebus pygerythrus* |
| SRR1046724 | *Chlorocebus pygerythrus* |
| SRR1046718 | *Chlorocebus pygerythrus* |
| SRR1046722 | *Chlorocebus pygerythrus* |
| SRR1046723 | *Chlorocebus pygerythrus* |
| SRR1046742 | *Chlorocebus pygerythrus* |
| SRR1046721 | *Chlorocebus pygerythrus* |
| SRR1046754 | *Chlorocebus pygerythrus* |
| SRR1046735 | *Chlorocebus pygerythrus* |
| SRR1046751 | *Chlorocebus pygerythrus* |
| SRR1046713 | *Chlorocebus pygerythrus* |
| SRR1046719 | *Chlorocebus pygerythrus* |
| SRR1046748 | *Chlorocebus pygerythrus* |
| SRR1046736 | *Chlorocebus pygerythrus* |
| SRR1046739 | *Chlorocebus pygerythrus* |
| SRR1046730 | *Chlorocebus pygerythrus* |
| SRR1046720 | *Chlorocebus pygerythrus* |
| SRR1046741 | *Chlorocebus pygerythrus* |
| SRR1046740 | *Chlorocebus pygerythrus* |
| SRR5171870 | *Chlorocebus aethiops* |
| SRR5171871 | *Chlorocebus aethiops* |
| SRR5171872 | *Chlorocebus aethiops* |
| SRR5171873 | *Chlorocebus aethiops* |
| SRR5171874 | *Chlorocebus aethiops* |
| SRR5171875 | *Chlorocebus aethiops* |
| SRR357433 | *Chlorocebus aethiops* |
| SRR361347 | *Chlorocebus aethiops* |
| SRR636841 | *Chlorocebus aethiops* |
| SRR636936 | *Chlorocebus aethiops* |
| SRR357430 | *Chlorocebus aethiops* |
| SRR357414 | *Chlorocebus aethiops* |
| SRR636891 | *Chlorocebus aethiops* |
| SRR5163655 | *Chlorocebus aethiops* |
| SRR5163659 | *Chlorocebus aethiops* |
| SRR5163658 | *Chlorocebus aethiops* |
| SRR5163657 | *Chlorocebus aethiops* |
| SRR933997 | *Chlorocebus aethiops* |
| SRR933996 | *Chlorocebus aethiops* |
| SRR5163654 | *Chlorocebus aethiops* |
| SRR5163656 | *Chlorocebus aethiops* |
| SRR832929 | *Macaca fuscata* |
| SRR832928 | *Macaca fuscata* |
| SRR1758963 | *Macaca fuscata* |
| SRR1758975 | *Macaca fuscata* |
| SRR832930 | *Macaca fuscata* |
| SRR1758974 | *Macaca fuscata* |
| SRR1758968 | *Macaca fuscata* |
| SRR1758973 | *Macaca fuscata* |
| SRR1758971 | *Macaca fuscata* |
| SRR1758964 | *Macaca fuscata* |
| SRR1758967 | *Macaca fuscata* |
| SRR1758969 | *Macaca fuscata* |
| SRR1758970 | *Macaca fuscata* |
| SRR1758966 | *Macaca fuscata* |
| SRR1758972 | *Macaca fuscata* |
| SRR1758965 | *Macaca fuscata* |
| SRR1602579 | *Macaca fuscata* |
| SRR832931 | *Macaca fuscata* |
| SRR832932 | *Macaca fuscata* |
| SRR1758962 | *Macaca fuscata* |
| SRR832917 | *Macaca fascicularis* |
| SRR832918 | *Macaca fascicularis* |
| SRR832924 | *Macaca fascicularis* |
| SRR832923 | *Macaca fascicularis* |
| SRR1758935 | *Macaca fascicularis* |
| SRR832970 | *Macaca fascicularis* |
| SRR1758957 | *Macaca fascicularis* |
| SRR1758936 | *Macaca fascicularis* |
| SRR1758952 | *Macaca fascicularis* |
| SRR832919 | *Macaca fascicularis* |
| SRR1758958 | *Macaca fascicularis* |
| SRR1758950 | *Macaca fascicularis* |
| SRR1758945 | *Macaca fascicularis* |
| SRR1758960 | *Macaca fascicularis* |
| SRR1758941 | *Macaca fascicularis* |
| SRR1758953 | *Macaca fascicularis* |
| SRR1758959 | *Macaca fascicularis* |
| SRR1758944 | *Macaca fascicularis* |
| SRR1758939 | *Macaca fascicularis* |
| SRR1758938 | *Macaca fascicularis* |
| SRR1758937 | *Macaca fascicularis* |
| SRR1758943 | *Macaca fascicularis* |
| SRR1758940 | *Macaca fascicularis* |
| SRR1758956 | *Macaca fascicularis* |
| SRR1758955 | *Macaca fascicularis* |
| SRR1758961 | *Macaca fascicularis* |
| SRR1758947 | *Macaca fascicularis* |
| SRR1758934 | *Macaca fascicularis* |
| SRR1758942 | *Macaca fascicularis* |
| SRR1758946 | *Macaca fascicularis* |
| SRR1758954 | *Macaca fascicularis* |
| SRR1602577 | *Macaca fascicularis* |
| SRR1758951 | *Macaca fascicularis* |
| SRR1602578 | *Macaca fascicularis* |
| SRR832922 | *Macaca fascicularis* |
| SRR832921 | *Macaca fascicularis* |
| SRR1758949 | *Macaca fascicularis* |
| SRR3957242 | *Macaca fascicularis* |
| SRR3957243 | *Macaca fascicularis* |
| SRR3957238 | *Macaca fascicularis* |
| SRR3957252 | *Macaca fascicularis* |
| SRR1758948 | *Macaca fascicularis* |
| SRR3957239 | *Macaca fascicularis* |
| SRR2057724 | *Macaca fascicularis* |
| SRR832920 | *Macaca fascicularis* |
| SRR2057725 | *Macaca fascicularis* |
| SRR3957241 | *Macaca fascicularis* |
| SRR3957240 | *Macaca fascicularis* |
| SRR2057721 | *Macaca fascicularis* |
| SRR2057722 | *Macaca fascicularis* |
| SRR2057726 | *Macaca fascicularis* |
| SRR3957247 | *Macaca fascicularis* |
| SRR3957248 | *Macaca fascicularis* |
| SRR2057720 | *Macaca fascicularis* |
| SRR2057723 | *Macaca fascicularis* |
| SRR2057719 | *Macaca fascicularis* |
| SRR3957245 | *Macaca fascicularis* |
| SRR3957244 | *Macaca fascicularis* |
| SRR223513 | *Macaca fascicularis* |
| SRR223514 | *Macaca fascicularis* |
| SRR223512 | *Macaca fascicularis* |
| SRR223516 | *Macaca fascicularis* |
| SRR223515 | *Macaca fascicularis* |
| SRR3957250 | *Macaca fascicularis* |
| SRR223517 | *Macaca fascicularis* |
| SRR3957249 | *Macaca fascicularis* |
| SRR1015772 | *Macaca fascicularis* |
| SRR1015770 | *Macaca fascicularis* |
| SRR1015774 | *Macaca fascicularis* |
| SRR1015780 | *Macaca fascicularis* |
| SRR1015777 | *Macaca fascicularis* |
| SRR1015768 | *Macaca fascicularis* |
| SRR1015773 | *Macaca fascicularis* |
| SRR1015781 | *Macaca fascicularis* |
| SRR3957251 | *Macaca fascicularis* |
| SRR1015759 | *Macaca fascicularis* |
| SRR1015763 | *Macaca fascicularis* |
| SRR1015776 | *Macaca fascicularis* |
| SRR1015779 | *Macaca fascicularis* |
| SRR1015761 | *Macaca fascicularis* |
| SRR1015782 | *Macaca fascicularis* |
| SRR1015775 | *Macaca fascicularis* |
| SRR1015760 | *Macaca fascicularis* |
| SRR1015762 | *Macaca fascicularis* |
| SRR1015778 | *Macaca fascicularis* |
| SRR3921583 | *Macaca fascicularis* |
| SRR1299387 | *Macaca fascicularis* |
| SRR1015767 | *Macaca fascicularis* |
| SRR1015769 | *Macaca fascicularis* |
| SRR3921573 | *Macaca fascicularis* |
| SRR2015597 | *Macaca fascicularis* |
| SRR1015771 | *Macaca fascicularis* |
| SRR3921578 | *Macaca fascicularis* |
| SRR2015585 | *Macaca fascicularis* |
| SRR1015764 | *Macaca fascicularis* |
| SRR1015766 | *Macaca fascicularis* |
| SRR3921584 | *Macaca fascicularis* |
| SRR1299386 | *Macaca fascicularis* |
| SRR1735736 | *Macaca fascicularis* |
| SRR2015584 | *Macaca fascicularis* |
| SRR2015582 | *Macaca fascicularis* |
| SRR1299384 | *Macaca fascicularis* |
| SRR3921585 | *Macaca fascicularis* |
| SRR1299385 | *Macaca fascicularis* |
| SRR2015603 | *Macaca fascicularis* |
| SRR1735746 | *Macaca fascicularis* |
| SRR1299383 | *Macaca fascicularis* |
| SRR1735726 | *Macaca fascicularis* |
| SRR2015600 | *Macaca fascicularis* |
| SRR1506083 | *Macaca fascicularis* |
| SRR3921575 | *Macaca fascicularis* |
| SRR3921574 | *Macaca fascicularis* |
| SRR2015583 | *Macaca fascicularis* |
| SRR1015765 | *Macaca fascicularis* |
| SRR1735728 | *Macaca fascicularis* |
| SRR1735735 | *Macaca fascicularis* |
| SRR1735732 | *Macaca fascicularis* |
| SRR1735727 | *Macaca fascicularis* |
| SRR2015578 | *Macaca fascicularis* |
| SRR1735752 | *Macaca fascicularis* |
| SRR2015601 | *Macaca fascicularis* |
| SRR1735738 | *Macaca fascicularis* |
| SRR2015586 | *Macaca fascicularis* |
| SRR3921572 | *Macaca fascicularis* |
| SRR3921579 | *Macaca fascicularis* |
| SRR1735745 | *Macaca fascicularis* |
| SRR1735743 | *Macaca fascicularis* |
| SRR3921576 | *Macaca fascicularis* |
| SRR3921589 | *Macaca fascicularis* |
| SRR3921581 | *Macaca fascicularis* |
| SRR3921580 | *Macaca fascicularis* |
| SRR1299381 | *Macaca fascicularis* |
| SRR1735733 | *Macaca fascicularis* |
| SRR2015587 | *Macaca fascicularis* |
| SRR2015589 | *Macaca fascicularis* |
| SRR2015598 | *Macaca fascicularis* |
| SRR3921587 | *Macaca fascicularis* |
| SRR1735731 | *Macaca fascicularis* |
| SRR3921588 | *Macaca fascicularis* |
| SRR3921592 | *Macaca fascicularis* |
| SRR1735730 | *Macaca fascicularis* |
| SRR1735748 | *Macaca fascicularis* |
| SRR3921577 | *Macaca fascicularis* |
| SRR1755244 | *Macaca fascicularis* |
| SRR2015602 | *Macaca fascicularis* |
| SRR1735744 | *Macaca fascicularis* |
| SRR1735734 | *Macaca fascicularis* |
| SRR1735740 | *Macaca fascicularis* |
| SRR3921590 | *Macaca fascicularis* |
| SRR1735729 | *Macaca fascicularis* |
| SRR3957246 | *Macaca fascicularis* |
| SRR1735725 | *Macaca fascicularis* |
| SRR2015580 | *Macaca fascicularis* |
| SRR1735737 | *Macaca fascicularis* |
| SRR3921586 | *Macaca fascicularis* |
| SRR3921591 | *Macaca fascicularis* |
| SRR1506085 | *Macaca fascicularis* |
| SRR3921582 | *Macaca fascicularis* |
| SRR1755240 | *Macaca fascicularis* |
| SRR1735747 | *Macaca fascicularis* |
| SRR2015593 | *Macaca fascicularis* |
| SRR1735741 | *Macaca fascicularis* |
| SRR1735750 | *Macaca fascicularis* |
| SRR1735753 | *Macaca fascicularis* |
| SRR1735751 | *Macaca fascicularis* |
| SRR2015591 | *Macaca fascicularis* |
| SRR1735742 | *Macaca fascicularis* |
| DRR001354 | *Macaca fascicularis* |
| SRR1755239 | *Macaca fascicularis* |
| SRR1735749 | *Macaca fascicularis* |
| SRR2015595 | *Macaca fascicularis* |
| SRR1755243 | *Macaca fascicularis* |
| SRR1735754 | *Macaca fascicularis* |
| SRR1735739 | *Macaca fascicularis* |
| SRR1506084 | *Macaca fascicularis* |
| SRR1035249 | *Macaca fascicularis* |
| SRR832936 | *Macaca nemestrina* |
| SRR832935 | *Macaca nemestrina* |
| SRR1759005 | *Macaca nemestrina* |
| SRR1759012 | *Macaca nemestrina* |
| SRR1759007 | *Macaca nemestrina* |
| SRR3932676 | *Macaca nemestrina* |
| SRR1759013 | *Macaca nemestrina* |
| SRR1759014 | *Macaca nemestrina* |
| SRR1759006 | *Macaca nemestrina* |
| SRR1759010 | *Macaca nemestrina* |
| SRR1759009 | *Macaca nemestrina* |
| SRR1759008 | *Macaca nemestrina* |
| SRR1759011 | *Macaca nemestrina* |
| SRR1759015 | *Macaca nemestrina* |
| SRR1759016 | *Macaca nemestrina* |
| SRR1759004 | *Macaca nemestrina* |
| SRR1602581 | *Macaca nemestrina* |
| SRR3932677 | *Macaca nemestrina* |
| SRR832942 | *Macaca nemestrina* |
| SRR832943 | *Macaca nemestrina* |
| SRR3932678 | *Macaca nemestrina* |
| SRR832941 | *Macaca nemestrina* |
| SRR832940 | *Macaca nemestrina* |
| SRR1739301 | *Macaca nemestrina* |
| SRR1739297 | *Macaca nemestrina* |
| SRR1739298 | *Macaca nemestrina* |
| SRR1739299 | *Macaca nemestrina* |
| SRR3480538 | *Macaca nemestrina* |
| SRR3480537 | *Macaca nemestrina* |
| SRR3480540 | *Macaca nemestrina* |
| SRR3480539 | *Macaca nemestrina* |
| SRR3480541 | *Macaca nemestrina* |
| SRR3480536 | *Macaca nemestrina* |
| SRR3480535 | *Macaca nemestrina* |
| SRR3480533 | *Macaca nemestrina* |
| SRR3480532 | *Macaca nemestrina* |
| SRR3480534 | *Macaca nemestrina* |
| SRR832948 | *Macaca mulatta* |
| SRR832949 | *Macaca mulatta* |
| SRR832945 | *Macaca mulatta* |
| SRR832944 | *Macaca mulatta* |
| SRR2337336 | *Macaca mulatta* |
| SRR2337337 | *Macaca mulatta* |
| ERR247247 | *Macaca mulatta* |
| SRR1369648 | *Macaca mulatta* |
| SRR1369618 | *Macaca mulatta* |
| SRR1369615 | *Macaca mulatta* |
| SRR1369644 | *Macaca mulatta* |
| SRR1979020 | *Macaca mulatta* |
| SRR1369631 | *Macaca mulatta* |
| SRR1369606 | *Macaca mulatta* |
| SRR1369622 | *Macaca mulatta* |
| SRR1369657 | *Macaca mulatta* |
| SRR1978687 | *Macaca mulatta* |
| SRR1369656 | *Macaca mulatta* |
| SRR3169277 | *Macaca mulatta* |
| SRR1369647 | *Macaca mulatta* |
| SRR594463 | *Macaca mulatta* |
| SRR594461 | *Macaca mulatta* |
| SRR1369645 | *Macaca mulatta* |
| SRR1369629 | *Macaca mulatta* |
| SRR594459 | *Macaca mulatta* |
| SRR1369610 | *Macaca mulatta* |
| SRR594460 | *Macaca mulatta* |
| SRR1369663 | *Macaca mulatta* |
| SRR1369640 | *Macaca mulatta* |
| SRR594457 | *Macaca mulatta* |
| SRR594458 | *Macaca mulatta* |
| SRR1369613 | *Macaca mulatta* |
| SRR594455 | *Macaca mulatta* |
| SRR1369623 | *Macaca mulatta* |
| ERR247246 | *Macaca mulatta* |
| SRR832950 | *Macaca mulatta* |
| SRR594456 | *Macaca mulatta* |
| SRR2040593 | *Macaca mulatta* |
| SRR1369672 | *Macaca mulatta* |
| SRR1369642 | *Macaca mulatta* |
| SRR1369664 | *Macaca mulatta* |
| SRR2040592 | *Macaca mulatta* |
| SRR2040595 | *Macaca mulatta* |
| SRR594462 | *Macaca mulatta* |
| SRR1369627 | *Macaca mulatta* |
| SRR1369671 | *Macaca mulatta* |
| SRR1369612 | *Macaca mulatta* |
| SRR1369669 | *Macaca mulatta* |
| SRR1369643 | *Macaca mulatta* |
| SRR1369655 | *Macaca mulatta* |
| SRR389101 | *Macaca mulatta* |
| SRR1369658 | *Macaca mulatta* |
| SRR1369668 | *Macaca mulatta* |
| SRR2040594 | *Macaca mulatta* |
| SRR1369649 | *Macaca mulatta* |
| SRR1369639 | *Macaca mulatta* |
| DRR003378 | *Macaca mulatta* |
| SRR1369661 | *Macaca mulatta* |
| SRR1369673 | *Macaca mulatta* |
| SRR1369628 | *Macaca mulatta* |
| SRR1369646 | *Macaca mulatta* |
| SRR1369608 | *Macaca mulatta* |
| SRR1646498 | *Macaca mulatta* |
| SRR389100 | *Macaca mulatta* |
| SRR1369633 | *Macaca mulatta* |
| SRR1369634 | *Macaca mulatta* |
| SRR1369635 | *Macaca mulatta* |
| SRR1538602 | *Macaca mulatta* |
| SRR1369636 | *Macaca mulatta* |
| SRR1369620 | *Macaca mulatta* |
| SRR1369626 | *Macaca mulatta* |
| SRR1369609 | *Macaca mulatta* |
| SRR832971 | *Macaca mulatta* |
| DRR003380 | *Macaca mulatta* |
| SRR1602583 | *Macaca mulatta* |
| DRR003379 | *Macaca mulatta* |
| SRR630492 | *Macaca mulatta* |
| ERR247249 | *Macaca mulatta* |
| SRR1514858 | *Macaca mulatta* |
| SRR1369667 | *Macaca mulatta* |
| SRR1514856 | *Macaca mulatta* |
| SRR3112568 | *Macaca mulatta* |
| SRR1261481 | *Macaca mulatta* |
| SRR1514854 | *Macaca mulatta* |
| SRR1602582 | *Macaca mulatta* |
| SRR1369659 | *Macaca mulatta* |
| SRR1635016 | *Macaca mulatta* |
| SRR389099 | *Macaca mulatta* |
| SRR389102 | *Macaca mulatta* |
| SRR1369662 | *Macaca mulatta* |
| SRR1369616 | *Macaca mulatta* |
| SRR1369674 | *Macaca mulatta* |
| SRR2184106 | *Macaca mulatta* |
| SRR1369641 | *Macaca mulatta* |
| SRR1369654 | *Macaca mulatta* |
| SRR1369653 | *Macaca mulatta* |
| SRR1514852 | *Macaca mulatta* |
| SRR630494 | *Macaca mulatta* |
| SRR1048280 | *Macaca mulatta* |
| SRR1514855 | *Macaca mulatta* |
| SRR1514853 | *Macaca mulatta* |
| SRR1369666 | *Macaca mulatta* |
| SRR1602572 | *Macaca mulatta* |
| SRR1602567 | *Macaca mulatta* |
| SRR5273767 | *Macaca mulatta* |
| SRR1369621 | *Macaca mulatta* |
| ERR247251 | *Macaca mulatta* |
| SRR1369619 | *Macaca mulatta* |
| SRR1514857 | *Macaca mulatta* |
| ERR247245 | *Macaca mulatta* |
| SRR1369624 | *Macaca mulatta* |
| SRR1514859 | *Macaca mulatta* |
| SRR1977654 | *Macaca mulatta* |
| SRR5273753 | *Macaca mulatta* |
| SRR4304880 | *Macaca mulatta* |
| SRR1977658 | *Macaca mulatta* |
| SRR389103 | *Macaca mulatta* |
| SRR1602569 | *Macaca mulatta* |
| ERR247253 | *Macaca mulatta* |
| SRR1778441 | *Macaca mulatta* |
| SRR1602568 | *Macaca mulatta* |
| SRR1051572 | *Macaca mulatta* |
| SRR5273741 | *Macaca mulatta* |
| SRR630493 | *Macaca mulatta* |
| SRR1369650 | *Macaca mulatta* |
| SRR832952 | *Macaca mulatta* |
| SRR5273755 | *Macaca mulatta* |
| SRR1369652 | *Macaca mulatta* |
| SRR5273756 | *Macaca mulatta* |
| SRR3112570 | *Macaca mulatta* |
| SRR1746861 | *Macaca mulatta* |
| SRR1602571 | *Macaca mulatta* |
| SRR1602566 | *Macaca mulatta* |
| SRR5273742 | *Macaca mulatta* |
| SRR1048278 | *Macaca mulatta* |
| SRR1048279 | *Macaca mulatta* |
| SRR1602570 | *Macaca mulatta* |
| SRR1602563 | *Macaca mulatta* |
| SRR3112574 | *Macaca mulatta* |
| SRR2337332 | *Macaca mulatta* |
| SRR832946 | *Macaca mulatta* |
| SRR5273754 | *Macaca mulatta* |
| DRR003377 | *Macaca mulatta* |
| SRR5273748 | *Macaca mulatta* |
| SRR832947 | *Macaca mulatta* |
| SRR5273747 | *Macaca mulatta* |
| SRR5273762 | *Macaca mulatta* |
| SRR1602561 | *Macaca mulatta* |
| SRR1369670 | *Macaca mulatta* |
| SRR1048277 | *Macaca mulatta* |
| SRR1051563 | *Macaca mulatta* |
| SRR3112573 | *Macaca mulatta* |
| SRR3112567 | *Macaca mulatta* |
| SRR2337322 | *Macaca mulatta* |
| SRR1602573 | *Macaca mulatta* |
| SRR2337329 | *Macaca mulatta* |
| SRR5273761 | *Macaca mulatta* |
| SRR1369607 | *Macaca mulatta* |
| SRR832953 | *Macaca mulatta* |
| SRR5273765 | *Macaca mulatta* |
| SRR5273752 | *Macaca mulatta* |
| SRR4304875 | *Macaca mulatta* |
| SRR2337326 | *Macaca mulatta* |
| SRR5273757 | *Macaca mulatta* |
| SRR5273763 | *Macaca mulatta* |
| SRR1746856 | *Macaca mulatta* |
| SRR1602564 | *Macaca mulatta* |
| SRR1369617 | *Macaca mulatta* |
| SRR1369630 | *Macaca mulatta* |
| SRR1602574 | *Macaca mulatta* |
| SRR5273739 | *Macaca mulatta* |
| SRR1240147 | *Macaca mulatta* |
| SRR1051562 | *Macaca mulatta* |
| SRR2337324 | *Macaca mulatta* |
| SRR2337318 | *Macaca mulatta* |
| SRR5273750 | *Macaca mulatta* |
| SRR2337330 | *Macaca mulatta* |
| SRR1240155 | *Macaca mulatta* |
| SRR1240148 | *Macaca mulatta* |
| SRR5273745 | *Macaca mulatta* |
| SRR5273766 | *Macaca mulatta* |
| SRR1369660 | *Macaca mulatta* |
| SRR2337328 | *Macaca mulatta* |
| SRR3112566 | *Macaca mulatta* |
| SRR1240142 | *Macaca mulatta* |
| SRR2337315 | *Macaca mulatta* |
| SRR2337331 | *Macaca mulatta* |
| SRR4304860 | *Macaca mulatta* |
| SRR2337321 | *Macaca mulatta* |
| SRR1240158 | *Macaca mulatta* |
| SRR1240145 | *Macaca mulatta* |
| SRR3112569 | *Macaca mulatta* |
| SRR1240141 | *Macaca mulatta* |
| SRR2337327 | *Macaca mulatta* |
| SRR2337319 | *Macaca mulatta* |
| SRR1240159 | *Macaca mulatta* |
| SRR2337313 | *Macaca mulatta* |
| SRR1300775 | *Macaca mulatta* |
| SRR1300773 | *Macaca mulatta* |
| SRR1369651 | *Macaca mulatta* |
| SRR2337335 | *Macaca mulatta* |
| SRR1369614 | *Macaca mulatta* |
| SRR3112572 | *Macaca mulatta* |
| SRR2337317 | *Macaca mulatta* |
| SRR2337314 | *Macaca mulatta* |
| SRR4242887 | *Macaca mulatta* |
| SRR1369638 | *Macaca mulatta* |
| ERR247248 | *Macaca mulatta* |
| SRR1240146 | *Macaca mulatta* |
| SRR3112571 | *Macaca mulatta* |
| SRR2337312 | *Macaca mulatta* |
| SRR1300774 | *Macaca mulatta* |
| SRR5273738 | *Macaca mulatta* |
| SRR223519 | *Macaca mulatta* |
| SRR4242894 | *Macaca mulatta* |
| SRR1369605 | *Macaca mulatta* |
| SRR5273760 | *Macaca mulatta* |
| SRR4242892 | *Macaca mulatta* |
| SRR4242890 | *Macaca mulatta* |
| SRR5273759 | *Macaca mulatta* |
| SRR4242893 | *Macaca mulatta* |
| SRR1602559 | *Macaca mulatta* |
| SRR2337325 | *Macaca mulatta* |
| SRR5273758 | *Macaca mulatta* |
| SRR2337316 | *Macaca mulatta* |
| SRR4242891 | *Macaca mulatta* |
| SRR3885090 | *Macaca mulatta* |
| SRR2337320 | *Macaca mulatta* |
| SRR2532180 | *Macaca mulatta* |
| SRR1240149 | *Macaca mulatta* |
| SRR3885111 | *Macaca mulatta* |
| SRR5273746 | *Macaca mulatta* |
| SRR1240160 | *Macaca mulatta* |
| SRR4304896 | *Macaca mulatta* |
| SRR223522 | *Macaca mulatta* |
| SRR2337333 | *Macaca mulatta* |
| SRR1240150 | *Macaca mulatta* |
| SRR3660683 | *Macaca mulatta* |
| SRR3661151 | *Macaca mulatta* |
| SRR5273744 | *Macaca mulatta* |
| SRR5273751 | *Macaca mulatta* |
| SRR3885089 | *Macaca mulatta* |
| SRR5273743 | *Macaca mulatta* |
| SRR4242888 | *Macaca mulatta* |
| SRR5273749 | *Macaca mulatta* |
| SRR1369611 | *Macaca mulatta* |
| ERR247250 | *Macaca mulatta* |
| SRR1240139 | *Macaca mulatta* |
| SRR3885100 | *Macaca mulatta* |
| SRR1640449 | *Macaca mulatta* |
| SRR2674772 | *Macaca mulatta* |
| SRR3885104 | *Macaca mulatta* |
| ERR247252 | *Macaca mulatta* |
| SRR1282448 | *Macaca mulatta* |
| SRR2337323 | *Macaca mulatta* |
| ERR247244 | *Macaca mulatta* |
| SRR3885101 | *Macaca mulatta* |
| SRR1635039 | *Macaca mulatta* |
| SRR223523 | *Macaca mulatta* |
| SRR3309968 | *Macaca mulatta* |
| SRR3885092 | *Macaca mulatta* |
| SRR1282447 | *Macaca mulatta* |
| SRR223521 | *Macaca mulatta* |
| SRR3309970 | *Macaca mulatta* |
| SRR1282438 | *Macaca mulatta* |
| SRR1282426 | *Macaca mulatta* |
| SRR1282437 | *Macaca mulatta* |
| SRR3885096 | *Macaca mulatta* |
| SRR2184040 | *Macaca mulatta* |
| SRR5051057 | *Macaca mulatta* |
| SRR1282471 | *Macaca mulatta* |
| SRR5197965 | *Macaca mulatta* |
| SRR306779 | *Macaca mulatta* |
| SRR1104954 | *Macaca mulatta* |
| SRR1282440 | *Macaca mulatta* |
| SRR1240138 | *Macaca mulatta* |
| SRR1369632 | *Macaca mulatta* |
| SRR1282462 | *Macaca mulatta* |
| SRR1240156 | *Macaca mulatta* |
| SRR1282430 | *Macaca mulatta* |
| SRR1640450 | *Macaca mulatta* |
| SRR5351436 | *Macaca mulatta* |
| SRR2029580 | *Macaca mulatta* |
| SRR1240143 | *Macaca mulatta* |
| SRR5351438 | *Macaca mulatta* |
| SRR5051060 | *Macaca mulatta* |
| SRR4304876 | *Macaca mulatta* |
| SRR3306984 | *Macaca mulatta* |
| SRR1282425 | *Macaca mulatta* |
| SRR1282450 | *Macaca mulatta* |
| SRR3885091 | *Macaca mulatta* |
| SRR1282464 | *Macaca mulatta* |
| SRR3309958 | *Macaca mulatta* |
| SRR3885095 | *Macaca mulatta* |
| SRR358985 | *Macaca mulatta* |
| SRR1240157 | *Macaca mulatta* |
| SRR3018847 | *Macaca mulatta* |
| SRR223518 | *Macaca mulatta* |
| SRR1051561 | *Macaca mulatta* |
| SRR4304890 | *Macaca mulatta* |
| SRR1282449 | *Macaca mulatta* |
| SRR5351441 | *Macaca mulatta* |
| SRR3885083 | *Macaca mulatta* |
| SRR1240151 | *Macaca mulatta* |
| SRR1282452 | *Macaca mulatta* |
| SRR3306981 | *Macaca mulatta* |
| SRR1282442 | *Macaca mulatta* |
| SRR5197963 | *Macaca mulatta* |
| SRR1369665 | *Macaca mulatta* |
| SRR1282480 | *Macaca mulatta* |
| SRR1635914 | *Macaca mulatta* |
| SRR5351437 | *Macaca mulatta* |
| SRR1282436 | *Macaca mulatta* |
| SRR1282473 | *Macaca mulatta* |
| SRR1282478 | *Macaca mulatta* |
| SRR3309969 | *Macaca mulatta* |
| SRR1240154 | *Macaca mulatta* |
| SRR3885088 | *Macaca mulatta* |
| SRR4304882 | *Macaca mulatta* |
| SRR3885093 | *Macaca mulatta* |
| SRR5197968 | *Macaca mulatta* |
| SRR1282477 | *Macaca mulatta* |
| SRR3885099 | *Macaca mulatta* |
| SRR3885109 | *Macaca mulatta* |
| SRR3885112 | *Macaca mulatta* |
| SRR5051061 | *Macaca mulatta* |
| SRR4304873 | *Macaca mulatta* |
| SRR3017209 | *Macaca mulatta* |
| SRR1282474 | *Macaca mulatta* |
| SRR5351435 | *Macaca mulatta* |
| SRR1538601 | *Macaca mulatta* |
| SRR5051055 | *Macaca mulatta* |
| SRR1746855 | *Macaca mulatta* |
| SRR3885087 | *Macaca mulatta* |
| SRR5351432 | *Macaca mulatta* |
| SRR1282470 | *Macaca mulatta* |
| SRR5051062 | *Macaca mulatta* |
| SRR3885103 | *Macaca mulatta* |
| SRR3661109 | *Macaca mulatta* |
| SRR1282475 | *Macaca mulatta* |
| SRR3885098 | *Macaca mulatta* |
| SRR5351431 | *Macaca mulatta* |
| SRR1282423 | *Macaca mulatta* |
| SRR1538600 | *Macaca mulatta* |
| SRR3661162 | *Macaca mulatta* |
| SRR1282432 | *Macaca mulatta* |
| SRR4304878 | *Macaca mulatta* |
| SRR1282456 | *Macaca mulatta* |
| SRR1282429 | *Macaca mulatta* |
| SRR3885110 | *Macaca mulatta* |
| SRR1282459 | *Macaca mulatta* |
| SRR1369637 | *Macaca mulatta* |
| SRR4304891 | *Macaca mulatta* |
| SRR3885106 | *Macaca mulatta* |
| SRR5197969 | *Macaca mulatta* |
| SRR1282472 | *Macaca mulatta* |
| SRR3885094 | *Macaca mulatta* |
| SRR5351440 | *Macaca mulatta* |
| SRR5051056 | *Macaca mulatta* |
| SRR5051058 | *Macaca mulatta* |
| SRR1282433 | *Macaca mulatta* |
| SRR5351433 | *Macaca mulatta* |
| SRR3885097 | *Macaca mulatta* |
| SRR223520 | *Macaca mulatta* |
| SRR358986 | *Macaca mulatta* |
| SRR3885105 | *Macaca mulatta* |
| SRR1575129 | *Macaca mulatta* |
| SRR1282461 | *Macaca mulatta* |
| SRR1282476 | *Macaca mulatta* |
| SRR1282424 | *Macaca mulatta* |
| SRR3661166 | *Macaca mulatta* |
| SRR1575128 | *Macaca mulatta* |
| SRR5351420 | *Macaca mulatta* |
| SRR4242919 | *Macaca mulatta* |
| SRR5197967 | *Macaca mulatta* |
| SRR4304869 | *Macaca mulatta* |
| SRR5273740 | *Macaca mulatta* |
| SRR594337 | *Macaca mulatta* |
| SRR3660928 | *Macaca mulatta* |
| SRR3885102 | *Macaca mulatta* |
| SRR4304881 | *Macaca mulatta* |
| SRR596308 | *Macaca mulatta* |
| SRR3661171 | *Macaca mulatta* |
| SRR3885084 | *Macaca mulatta* |
| SRR594026 | *Macaca mulatta* |
| SRR5197964 | *Macaca mulatta* |
| SRR5351413 | *Macaca mulatta* |
| SRR1282479 | *Macaca mulatta* |
| SRR1240144 | *Macaca mulatta* |
| SRR1282427 | *Macaca mulatta* |
| SRR596305 | *Macaca mulatta* |
| SRR4304886 | *Macaca mulatta* |
| SRR596307 | *Macaca mulatta* |
| SRR1240140 | *Macaca mulatta* |
| SRR3306980 | *Macaca mulatta* |
| SRR1282412 | *Macaca mulatta* |
| SRR3885108 | *Macaca mulatta* |
| SRR1282444 | *Macaca mulatta* |
| SRR1282435 | *Macaca mulatta* |
| SRR5351418 | *Macaca mulatta* |
| SRR596309 | *Macaca mulatta* |
| SRR5351416 | *Macaca mulatta* |
| SRR3885107 | *Macaca mulatta* |
| SRR1282428 | *Macaca mulatta* |
| SRR3661175 | *Macaca mulatta* |
| SRR1648451 | *Macaca mulatta* |
| SRR1240153 | *Macaca mulatta* |
| SRR5051059 | *Macaca mulatta* |
| SRR4304864 | *Macaca mulatta* |
| SRR3660628 | *Macaca mulatta* |
| SRR4242967 | *Macaca mulatta* |
| SRR4304862 | *Macaca mulatta* |
| SRR596306 | *Macaca mulatta* |
| SRR5351412 | *Macaca mulatta* |
| SRR4242953 | *Macaca mulatta* |
| SRR5351421 | *Macaca mulatta* |
| SRR5351439 | *Macaca mulatta* |
| SRR1051560 | *Macaca mulatta* |
| ERR731526 | *Macaca mulatta* |
| SRR1282466 | *Macaca mulatta* |
| SRR4242977 | *Macaca mulatta* |
| SRR1282481 | *Macaca mulatta* |
| ERR731514 | *Macaca mulatta* |
| SRR1282465 | *Macaca mulatta* |
| SRR5351411 | *Macaca mulatta* |
| SRR3661154 | *Macaca mulatta* |
| ERR731502 | *Macaca mulatta* |
| SRR4242952 | *Macaca mulatta* |
| SRR4304856 | *Macaca mulatta* |
| SRR4304857 | *Macaca mulatta* |
| SRR4304861 | *Macaca mulatta* |
| SRR1282431 | *Macaca mulatta* |
| SRR1282463 | *Macaca mulatta* |
| SRR1282460 | *Macaca mulatta* |
| SRR1051569 | *Macaca mulatta* |
| SRR3309967 | *Macaca mulatta* |
| SRR1282451 | *Macaca mulatta* |
| SRR1282441 | *Macaca mulatta* |
| SRR3309961 | *Macaca mulatta* |
| SRR3661165 | *Macaca mulatta* |
| SRR3661094 | *Macaca mulatta* |
| SRR5351424 | *Macaca mulatta* |
| SRR3660993 | *Macaca mulatta* |
| SRR3661108 | *Macaca mulatta* |
| SRR5351419 | *Macaca mulatta* |
| SRR594453 | *Macaca mulatta* |
| SRR4242906 | *Macaca mulatta* |
| SRR2467191 | *Macaca mulatta* |
| SRR1648453 | *Macaca mulatta* |
| ERR731490 | *Macaca mulatta* |
| SRR594467 | *Macaca mulatta* |
| SRR3660697 | *Macaca mulatta* |
| SRR594465 | *Macaca mulatta* |
| SRR3660933 | *Macaca mulatta* |
| SRR1051594 | *Macaca mulatta* |
| SRR596311 | *Macaca mulatta* |
| SRR4242920 | *Macaca mulatta* |
| SRR3661174 | *Macaca mulatta* |
| SRR596568 | *Macaca mulatta* |
| SRR1648454 | *Macaca mulatta* |
| SRR1509488 | *Macaca mulatta* |
| SRR1746859 | *Macaca mulatta* |
| SRR1104953 | *Macaca mulatta* |
| SRR1648452 | *Macaca mulatta* |
| SRR1051664 | *Macaca mulatta* |
| SRR1282445 | *Macaca mulatta* |
| SRR1746854 | *Macaca mulatta* |
| SRR2337334 | *Macaca mulatta* |
| SRR594471 | *Macaca mulatta* |
| SRR3661111 | *Macaca mulatta* |
| SRR3660661 | *Macaca mulatta* |
| SRR4242898 | *Macaca mulatta* |
| SRR3309964 | *Macaca mulatta* |
| SRR3661017 | *Macaca mulatta* |
| SRR1282439 | *Macaca mulatta* |
| SRR2467177 | *Macaca mulatta* |
| SRR3661093 | *Macaca mulatta* |
| SRR3660703 | *Macaca mulatta* |
| SRR1509496 | *Macaca mulatta* |
| SRR1282458 | *Macaca mulatta* |
| SRR3661153 | *Macaca mulatta* |
| SRR1051692 | *Macaca mulatta* |
| SRR1282443 | *Macaca mulatta* |
| SRR1282453 | *Macaca mulatta* |
| SRR596569 | *Macaca mulatta* |
| SRR4242976 | *Macaca mulatta* |
| SRR5351426 | *Macaca mulatta* |
| SRR3661164 | *Macaca mulatta* |
| SRR3661138 | *Macaca mulatta* |
| SRR1575130 | *Macaca mulatta* |
| SRR1282434 | *Macaca mulatta* |
| SRR2467193 | *Macaca mulatta* |
| SRR596312 | *Macaca mulatta* |
| SRR596310 | *Macaca mulatta* |
| SRR4242917 | *Macaca mulatta* |
| SRR1509492 | *Macaca mulatta* |
| SRR1240152 | *Macaca mulatta* |
| SRR4304898 | *Macaca mulatta* |
| SRR3001790 | *Macaca mulatta* |
| SRR1635913 | *Macaca mulatta* |
| SRR4304868 | *Macaca mulatta* |
| SRR1746857 | *Macaca mulatta* |
| SRR594451 | *Macaca mulatta* |
| SRR4304889 | *Macaca mulatta* |
| SRR1051595 | *Macaca mulatta* |
| SRR3309960 | *Macaca mulatta* |
| SRR1051568 | *Macaca mulatta* |
| SRR1648456 | *Macaca mulatta* |
| SRR3661147 | *Macaca mulatta* |
| SRR1635910 | *Macaca mulatta* |
| SRR2467196 | *Macaca mulatta* |
| SRR2467170 | *Macaca mulatta* |
| SRR1575127 | *Macaca mulatta* |
| SRR3306989 | *Macaca mulatta* |
| SRR3309962 | *Macaca mulatta* |
| SRR3661158 | *Macaca mulatta* |
| SRR2467155 | *Macaca mulatta* |
| SRR3660682 | *Macaca mulatta* |
| SRR3661036 | *Macaca mulatta* |
| SRR3661084 | *Macaca mulatta* |
| SRR3660888 | *Macaca mulatta* |
| SRR5351430 | *Macaca mulatta* |
| SRR3661033 | *Macaca mulatta* |
| SRR1648455 | *Macaca mulatta* |
| SRR3661144 | *Macaca mulatta* |
| SRR1509494 | *Macaca mulatta* |
| SRR950224 | *Macaca mulatta* |
| SRR1051601 | *Macaca mulatta* |
| SRR3660678 | *Macaca mulatta* |
| SRR2467166 | *Macaca mulatta* |
| SRR3661170 | *Macaca mulatta* |
| SRR1635911 | *Macaca mulatta* |
| SRR3660969 | *Macaca mulatta* |
| SRR3661088 | *Macaca mulatta* |
| SRR1282467 | *Macaca mulatta* |
| SRR2467154 | *Macaca mulatta* |
| SRR3661176 | *Macaca mulatta* |
| SRR5351415 | *Macaca mulatta* |
| SRR2467167 | *Macaca mulatta* |
| SRR3661103 | *Macaca mulatta* |
| SRR4304895 | *Macaca mulatta* |
| SRR1051559 | *Macaca mulatta* |
| SRR3661139 | *Macaca mulatta* |
| SRR3661058 | *Macaca mulatta* |
| SRR3661114 | *Macaca mulatta* |
| SRR3661163 | *Macaca mulatta* |
| SRR3660692 | *Macaca mulatta* |
| SRR3660694 | *Macaca mulatta* |
| SRR2467180 | *Macaca mulatta* |
| SRR5273764 | *Macaca mulatta* |
| SRR3661160 | *Macaca mulatta* |
| SRR2467190 | *Macaca mulatta* |
| SRR3661078 | *Macaca mulatta* |
| SRR596571 | *Macaca mulatta* |
| SRR2467158 | *Macaca mulatta* |
| SRR1282469 | *Macaca mulatta* |
| SRR1282468 | *Macaca mulatta* |
| SRR3661141 | *Macaca mulatta* |
| SRR2467173 | *Macaca mulatta* |
| SRR1282454 | *Macaca mulatta* |
| SRR3661023 | *Macaca mulatta* |
| SRR3661134 | *Macaca mulatta* |
| SRR3661169 | *Macaca mulatta* |
| SRR3660708 | *Macaca mulatta* |
| SRR3661155 | *Macaca mulatta* |
| SRR950258 | *Macaca mulatta* |
| SRR5351414 | *Macaca mulatta* |
| SRR3306978 | *Macaca mulatta* |
| SRR3661018 | *Macaca mulatta* |
| SRR596572 | *Macaca mulatta* |
| SRR5351417 | *Macaca mulatta* |
| SRR3661037 | *Macaca mulatta* |
| SRR1051600 | *Macaca mulatta* |
| SRR4242897 | *Macaca mulatta* |
| SRR596570 | *Macaca mulatta* |
| SRR3661038 | *Macaca mulatta* |
| SRR594466 | *Macaca mulatta* |
| SRR2467157 | *Macaca mulatta* |
| SRR950227 | *Macaca mulatta* |
| SRR2603330 | *Macaca mulatta* |
| SRR2096994 | *Macaca mulatta* |
| SRR3661054 | *Macaca mulatta* |
| SRR3660707 | *Macaca mulatta* |
| SRR3661115 | *Macaca mulatta* |
| SRR3661112 | *Macaca mulatta* |
| SRR1635909 | *Macaca mulatta* |
| SRR4242895 | *Macaca mulatta* |
| SRR3661135 | *Macaca mulatta* |
| SRR2467184 | *Macaca mulatta* |
| SRR1282446 | *Macaca mulatta* |
| SRR4242896 | *Macaca mulatta* |
| SRR1635912 | *Macaca mulatta* |
| SRR950292 | *Macaca mulatta* |
| SRR3661123 | *Macaca mulatta* |
| SRR4242903 | *Macaca mulatta* |
| SRR5351428 | *Macaca mulatta* |
| SRR594448 | *Macaca mulatta* |
| SRR2467194 | *Macaca mulatta* |
| SRR3661129 | *Macaca mulatta* |
| SRR3660679 | *Macaca mulatta* |
| SRR2467178 | *Macaca mulatta* |
| SRR594446 | *Macaca mulatta* |
| SRR3306982 | *Macaca mulatta* |
| SRR3661040 | *Macaca mulatta* |
| SRR3660662 | *Macaca mulatta* |
| SRR3660958 | *Macaca mulatta* |
| SRR3661119 | *Macaca mulatta* |
| SRR2467182 | *Macaca mulatta* |
| SRR3660965 | *Macaca mulatta* |
| SRR5351427 | *Macaca mulatta* |
| SRR3660660 | *Macaca mulatta* |
| SRR3660999 | *Macaca mulatta* |
| SRR1051663 | *Macaca mulatta* |
| SRR3660988 | *Macaca mulatta* |
| SRR3661179 | *Macaca mulatta* |
| SRR3661028 | *Macaca mulatta* |
| SRR2467165 | *Macaca mulatta* |
| SRR2467176 | *Macaca mulatta* |
| SRR3660650 | *Macaca mulatta* |
| SRR4242911 | *Macaca mulatta* |
| SRR3660665 | *Macaca mulatta* |
| SRR2467189 | *Macaca mulatta* |
| SRR3661106 | *Macaca mulatta* |
| SRR1952671 | *Macaca mulatta* |
| SRR1369625 | *Macaca mulatta* |
| SRR3309959 | *Macaca mulatta* |
| SRR3661045 | *Macaca mulatta* |
| SRR3660978 | *Macaca mulatta* |
| SRR3660996 | *Macaca mulatta* |
| SRR950261 | *Macaca mulatta* |
| SRR3660643 | *Macaca mulatta* |
| SRR3660898 | *Macaca mulatta* |
| SRR3660918 | *Macaca mulatta* |
| SRR594470 | *Macaca mulatta* |
| SRR2467198 | *Macaca mulatta* |
| SRR1952719 | *Macaca mulatta* |
| SRR5197966 | *Macaca mulatta* |
| SRR3661059 | *Macaca mulatta* |
| SRR3661120 | *Macaca mulatta* |
| SRR3660663 | *Macaca mulatta* |
| SRR2467168 | *Macaca mulatta* |
| SRR2674750 | *Macaca mulatta* |
| SRR3661046 | *Macaca mulatta* |
| SRR3306986 | *Macaca mulatta* |
| SRR3661063 | *Macaca mulatta* |
| SRR1746858 | *Macaca mulatta* |
| SRR4242902 | *Macaca mulatta* |
| SRR2467162 | *Macaca mulatta* |
| SRR3660974 | *Macaca mulatta* |
| SRR3661149 | *Macaca mulatta* |
| SRR3661014 | *Macaca mulatta* |
| SRR3661159 | *Macaca mulatta* |
| SRR3660982 | *Macaca mulatta* |
| SRR5351422 | *Macaca mulatta* |
| SRR832969 | *Macaca mulatta* |
| SRR5088727 | *Macaca mulatta* |
| SRR832964 | *Macaca mulatta* |
| SRR1509493 | *Macaca mulatta* |
| SRR3660669 | *Macaca mulatta* |
| SRR3661027 | *Macaca mulatta* |
| SRR3661150 | *Macaca mulatta* |
| SRR2467197 | *Macaca mulatta* |
| SRR832966 | *Macaca mulatta* |
| SRR4242954 | *Macaca mulatta* |
| SRR4304871 | *Macaca mulatta* |
| SRR3660671 | *Macaca mulatta* |
| SRR3660948 | *Macaca mulatta* |
| SRR3660666 | *Macaca mulatta* |
| SRR3660670 | *Macaca mulatta* |
| SRR1051662 | *Macaca mulatta* |
| SRR1952720 | *Macaca mulatta* |
| SRR3660994 | *Macaca mulatta* |
| SRR4242969 | *Macaca mulatta* |
| SRR3661148 | *Macaca mulatta* |
| SRR4304858 | *Macaca mulatta* |
| SRR950295 | *Macaca mulatta* |
| SRR3660989 | *Macaca mulatta* |
| SRR3661145 | *Macaca mulatta* |
| SRR3660645 | *Macaca mulatta* |
| SRR1952675 | *Macaca mulatta* |
| SRR5351429 | *Macaca mulatta* |
| SRR3661125 | *Macaca mulatta* |
| SRR3661128 | *Macaca mulatta* |
| SRR4242965 | *Macaca mulatta* |
| SRR4242968 | *Macaca mulatta* |
| SRR094948 | *Macaca mulatta* |
| SRR786430 | *Macaca mulatta* |
| SRR3661021 | *Macaca mulatta* |
| SRR4304859 | *Macaca mulatta* |
| SRR786431 | *Macaca mulatta* |
| SRR3660964 | *Macaca mulatta* |
| SRR3661055 | *Macaca mulatta* |
| SRR3661146 | *Macaca mulatta* |
| SRR832968 | *Macaca mulatta* |
| SRR3660677 | *Macaca mulatta* |
| SRR3660702 | *Macaca mulatta* |
| SRR5088729 | *Macaca mulatta* |
| SRR3660655 | *Macaca mulatta* |
| SRR2467192 | *Macaca mulatta* |
| SRR1003319 | *Macaca mulatta* |
| SRR2059410 | *Macaca mulatta* |
| SRR2467174 | *Macaca mulatta* |
| SRR2467179 | *Macaca mulatta* |
| SRR3661104 | *Macaca mulatta* |
| SRR3660794 | *Macaca mulatta* |
| SRR4242966 | *Macaca mulatta* |
| SRR4242973 | *Macaca mulatta* |
| SRR2467160 | *Macaca mulatta* |
| SRR4242955 | *Macaca mulatta* |
| SRR4242964 | *Macaca mulatta* |
| SRR3661083 | *Macaca mulatta* |
| SRR4242971 | *Macaca mulatta* |
| SRR594469 | *Macaca mulatta* |
| SRR1051599 | *Macaca mulatta* |
| SRR2059394 | *Macaca mulatta* |
| SRR4242963 | *Macaca mulatta* |
| SRR4242962 | *Macaca mulatta* |
| SRR1602560 | *Macaca mulatta* |
| SRR3660696 | *Macaca mulatta* |
| SRR4242915 | *Macaca mulatta* |
| SRR2467161 | *Macaca mulatta* |
| SRR4242972 | *Macaca mulatta* |
| SRR2467185 | *Macaca mulatta* |
| SRR1282457 | *Macaca mulatta* |
| SRR2467188 | *Macaca mulatta* |
| SRR3660627 | *Macaca mulatta* |
| SRR5088728 | *Macaca mulatta* |
| SRR1952673 | *Macaca mulatta* |
| SRR3660923 | *Macaca mulatta* |
| SRR832965 | *Macaca mulatta* |
| SRR3660814 | *Macaca mulatta* |
| SRR3661136 | *Macaca mulatta* |
| SRR3661172 | *Macaca mulatta* |
| SRR3660903 | *Macaca mulatta* |
| SRR594449 | *Macaca mulatta* |
| SRR3660970 | *Macaca mulatta* |
| SRR3661015 | *Macaca mulatta* |
| SRR3661022 | *Macaca mulatta* |
| SRR2467195 | *Macaca mulatta* |
| SRR3660654 | *Macaca mulatta* |
| SRR4242974 | *Macaca mulatta* |
| SRR3306977 | *Macaca mulatta* |
| SRR3661011 | *Macaca mulatta* |
| SRR1051590 | *Macaca mulatta* |
| SRR1282455 | *Macaca mulatta* |
| SRR2603328 | *Macaca mulatta* |
| SRR3661143 | *Macaca mulatta* |
| SRR4304893 | *Macaca mulatta* |
| SRR1003397 | *Macaca mulatta* |
| SRR3660788 | *Macaca mulatta* |
| SRR2467171 | *Macaca mulatta* |
| SRR3660688 | *Macaca mulatta* |
| SRR3309963 | *Macaca mulatta* |
| SRR1746860 | *Macaca mulatta* |
| SRR3661012 | *Macaca mulatta* |
| SRR3660699 | *Macaca mulatta* |
| SRR3660773 | *Macaca mulatta* |
| SRR3661124 | *Macaca mulatta* |
| SRR357438 | *Macaca mulatta* |
| SRR832967 | *Macaca mulatta* |
| SRR3660983 | *Macaca mulatta* |
| SRR3660844 | *Macaca mulatta* |
| SRR4242914 | *Macaca mulatta* |
| SRR594452 | *Macaca mulatta* |
| SRR3660783 | *Macaca mulatta* |
| SRR1952717 | *Macaca mulatta* |
| SRR2467169 | *Macaca mulatta* |
| SRR3661133 | *Macaca mulatta* |
| SRR5088730 | *Macaca mulatta* |
| SRR4242978 | *Macaca mulatta* |
| SRR2096990 | *Macaca mulatta* |
| SRR3660854 | *Macaca mulatta* |
| SRR3661001 | *Macaca mulatta* |
| SRR306790 | *Macaca mulatta* |
| SRR3661140 | *Macaca mulatta* |
| SRR3660629 | *Macaca mulatta* |
| SRR3660799 | *Macaca mulatta* |
| SRR3660647 | *Macaca mulatta* |
| SRR3660824 | *Macaca mulatta* |
| SRR3661013 | *Macaca mulatta* |
| SRR5351423 | *Macaca mulatta* |
| SRR2467163 | *Macaca mulatta* |
| SRR5351434 | *Macaca mulatta* |
| SRR3660829 | *Macaca mulatta* |
| SRR3661008 | *Macaca mulatta* |
| SRR4242979 | *Macaca mulatta* |
| SRR3660667 | *Macaca mulatta* |
| SRR3660869 | *Macaca mulatta* |
| SRR1952239 | *Macaca mulatta* |
| SRR3660979 | *Macaca mulatta* |
| SRR3661050 | *Macaca mulatta* |
| SRR3661004 | *Macaca mulatta* |
| SRR3660705 | *Macaca mulatta* |
| SRR3661177 | *Macaca mulatta* |
| SRR3660704 | *Macaca mulatta* |
| SRR2603331 | *Macaca mulatta* |
| SRR3660693 | *Macaca mulatta* |
| SRR3661069 | *Macaca mulatta* |
| SRR5088725 | *Macaca mulatta* |
| SRR3660630 | *Macaca mulatta* |
| SRR3660631 | *Macaca mulatta* |
| SRR4242909 | *Macaca mulatta* |
| SRR3660633 | *Macaca mulatta* |
| SRR2059391 | *Macaca mulatta* |
| SRR2096993 | *Macaca mulatta* |
| SRR3661056 | *Macaca mulatta* |
| SRR3661157 | *Macaca mulatta* |
| SRR786432 | *Macaca mulatta* |
| SRR3660652 | *Macaca mulatta* |
| SRR3660967 | *Macaca mulatta* |
| SRR1051598 | *Macaca mulatta* |
| SRR2467186 | *Macaca mulatta* |
| SRR3660675 | *Macaca mulatta* |
| SRR2059407 | *Macaca mulatta* |
| SRR2059415 | *Macaca mulatta* |
| SRR3661097 | *Macaca mulatta* |
| SRR3660992 | *Macaca mulatta* |
| SRR2467156 | *Macaca mulatta* |
| SRR2467164 | *Macaca mulatta* |
| SRR3661118 | *Macaca mulatta* |
| SRR3661161 | *Macaca mulatta* |
| SRR3661070 | *Macaca mulatta* |
| SRR1952670 | *Macaca mulatta* |
| SRR3660673 | *Macaca mulatta* |
| SRR3660709 | *Macaca mulatta* |
| SRR3660973 | *Macaca mulatta* |
| SRR3660985 | *Macaca mulatta* |
| SRR3660962 | *Macaca mulatta* |
| SRR649368 | *Macaca mulatta* |
| SRR2096989 | *Macaca mulatta* |
| ERR731525 | *Macaca mulatta* |
| SRR3661110 | *Macaca mulatta* |
| SRR3660834 | *Macaca mulatta* |
| SRR3661030 | *Macaca mulatta* |
| SRR3661026 | *Macaca mulatta* |
| SRR2467172 | *Macaca mulatta* |
| SRR3661095 | *Macaca mulatta* |
| SRR3660966 | *Macaca mulatta* |
| SRR331901 | *Macaca mulatta* |
| SRR649369 | *Macaca mulatta* |
| SRR4242910 | *Macaca mulatta* |
| SRR832959 | *Macaca mulatta* |
| ERR731501 | *Macaca mulatta* |
| SRR3661003 | *Macaca mulatta* |
| SRR3661019 | *Macaca mulatta* |
| SRR3660640 | *Macaca mulatta* |
| SRR4242900 | *Macaca mulatta* |
| ERR731513 | *Macaca mulatta* |
| SRR3660642 | *Macaca mulatta* |
| SRR3660664 | *Macaca mulatta* |
| SRR3660691 | *Macaca mulatta* |
| SRR3660809 | *Macaca mulatta* |
| SRR4242913 | *Macaca mulatta* |
| SRR2467183 | *Macaca mulatta* |
| SRR649370 | *Macaca mulatta* |
| SRR2059386 | *Macaca mulatta* |
| SRR3661152 | *Macaca mulatta* |
| SRR832961 | *Macaca mulatta* |
| SRR4242905 | *Macaca mulatta* |
| SRR3661067 | *Macaca mulatta* |
| SRR3660657 | *Macaca mulatta* |
| SRR3660685 | *Macaca mulatta* |
| SRR3660867 | *Macaca mulatta* |
| SRR2467159 | *Macaca mulatta* |
| SRR2059402 | *Macaca mulatta* |
| SRR832958 | *Macaca mulatta* |
| SRR2059390 | *Macaca mulatta* |
| SRR3660698 | *Macaca mulatta* |
| SRR3661044 | *Macaca mulatta* |
| SRR3660690 | *Macaca mulatta* |
| SRR3661031 | *Macaca mulatta* |
| SRR3660990 | *Macaca mulatta* |
| SRR2603332 | *Macaca mulatta* |
| SRR3660700 | *Macaca mulatta* |
| SRR3660998 | *Macaca mulatta* |
| SRR3660991 | *Macaca mulatta* |
| SRR3306979 | *Macaca mulatta* |
| SRR3660864 | *Macaca mulatta* |
| SRR832951 | *Macaca mulatta* |
| SRR3661068 | *Macaca mulatta* |
| SRR5088721 | *Macaca mulatta* |
| SRR3660995 | *Macaca mulatta* |
| SRR3660641 | *Macaca mulatta* |
| SRR3660649 | *Macaca mulatta* |
| ERR731489 | *Macaca mulatta* |
| SRR3661042 | *Macaca mulatta* |
| SRR3661107 | *Macaca mulatta* |
| SRR1952238 | *Macaca mulatta* |
| SRR2059421 | *Macaca mulatta* |
| SRR1051698 | *Macaca mulatta* |
| SRR1952672 | *Macaca mulatta* |
| SRR4242907 | *Macaca mulatta* |
| ERR731524 | *Macaca mulatta* |
| SRR3660972 | *Macaca mulatta* |
| SRR3661024 | *Macaca mulatta* |
| SRR1952667 | *Macaca mulatta* |
| SRR2059393 | *Macaca mulatta* |
| SRR594450 | *Macaca mulatta* |
| SRR3661061 | *Macaca mulatta* |
| SRR3660638 | *Macaca mulatta* |
| SRR3661009 | *Macaca mulatta* |
| SRR4012428 | *Macaca mulatta* |
| SRR594454 | *Macaca mulatta* |
| SRR4304866 | *Macaca mulatta* |
| ERR731512 | *Macaca mulatta* |
| SRR3660971 | *Macaca mulatta* |
| SRR3661080 | *Macaca mulatta* |
| SRR3661167 | *Macaca mulatta* |
| SRR3661043 | *Macaca mulatta* |
| SRR2059398 | *Macaca mulatta* |
| SRR3660658 | *Macaca mulatta* |
| SRR786429 | *Macaca mulatta* |
| SRR3660651 | *Macaca mulatta* |
| SRR2059406 | *Macaca mulatta* |
| SRR3660653 | *Macaca mulatta* |
| SRR3660648 | *Macaca mulatta* |
| ERR731500 | *Macaca mulatta* |
| SRR1047663 | *Macaca mulatta* |
| SRR1952718 | *Macaca mulatta* |
| SRR3661025 | *Macaca mulatta* |
| SRR5088736 | *Macaca mulatta* |
| SRR3660804 | *Macaca mulatta* |
| SRR2467187 | *Macaca mulatta* |
| SRR3660637 | *Macaca mulatta* |
| SRR1051733 | *Macaca mulatta* |
| SRR4242918 | *Macaca mulatta* |
| SRR5088724 | *Macaca mulatta* |
| SRR3661076 | *Macaca mulatta* |
| SRR2467200 | *Macaca mulatta* |
| SRR3661007 | *Macaca mulatta* |
| SRR2096992 | *Macaca mulatta* |
| SRR3661005 | *Macaca mulatta* |
| SRR3661098 | *Macaca mulatta* |
| SRR3660883 | *Macaca mulatta* |
| SRR2059420 | *Macaca mulatta* |
| SRR3661099 | *Macaca mulatta* |
| SRR3661113 | *Macaca mulatta* |
| SRR1051558 | *Macaca mulatta* |
| SRR594472 | *Macaca mulatta* |
| SRR3660968 | *Macaca mulatta* |
| SRR3661062 | *Macaca mulatta* |
| SRR5088723 | *Macaca mulatta* |
| SRR2059409 | *Macaca mulatta* |
| SRR3660656 | *Macaca mulatta* |
| SRR2674852 | *Macaca mulatta* |
| SRR3661092 | *Macaca mulatta* |
| SRR3660997 | *Macaca mulatta* |
| SRR3661101 | *Macaca mulatta* |
| SRR3661002 | *Macaca mulatta* |
| ERR731488 | *Macaca mulatta* |
| SRR2467181 | *Macaca mulatta* |
| SRR3661178 | *Macaca mulatta* |
| SRR3660701 | *Macaca mulatta* |
| SRR5088726 | *Macaca mulatta* |
| SRR3660943 | *Macaca mulatta* |
| SRR1602565 | *Macaca mulatta* |
| SRR2059395 | *Macaca mulatta* |
| SRR2059397 | *Macaca mulatta* |
| SRR3661156 | *Macaca mulatta* |
| SRR3661122 | *Macaca mulatta* |
| SRR208300 | *Macaca mulatta* |
| SRR2059412 | *Macaca mulatta* |
| SRR2059422 | *Macaca mulatta* |
| SRR4242901 | *Macaca mulatta* |
| SRR1051567 | *Macaca mulatta* |
| SRR3661057 | *Macaca mulatta* |
| SRR1051691 | *Macaca mulatta* |
| SRR5088722 | *Macaca mulatta* |
| SRR1952237 | *Macaca mulatta* |
| SRR3660689 | *Macaca mulatta* |
| SRR306782 | *Macaca mulatta* |
| SRR2059399 | *Macaca mulatta* |
| SRR1952669 | *Macaca mulatta* |
| SRR3661032 | *Macaca mulatta* |
| SRR3661082 | *Macaca mulatta* |
| SRR832962 | *Macaca mulatta* |
| SRR3660632 | *Macaca mulatta* |
| SRR2059385 | *Macaca mulatta* |
| SRR3660676 | *Macaca mulatta* |
| SRR3661035 | *Macaca mulatta* |
| SRR3660819 | *Macaca mulatta* |
| SRR3661081 | *Macaca mulatta* |
| SRR3661105 | *Macaca mulatta* |
| SRR1051589 | *Macaca mulatta* |
| SRR3661047 | *Macaca mulatta* |
| SRR3661096 | *Macaca mulatta* |
| SRR3661041 | *Macaca mulatta* |
| SRR3660768 | *Macaca mulatta* |
| SRR3661065 | *Macaca mulatta* |
| SRR3661075 | *Macaca mulatta* |
| SRR3661060 | *Macaca mulatta* |
| SRR3660986 | *Macaca mulatta* |
| SRR2096991 | *Macaca mulatta* |
| SRR594468 | *Macaca mulatta* |
| SRR3661049 | *Macaca mulatta* |
| SRR3661051 | *Macaca mulatta* |
| SRR3661066 | *Macaca mulatta* |
| SRR3660748 | *Macaca mulatta* |
| SRR3661077 | *Macaca mulatta* |
| SRR4304884 | *Macaca mulatta* |
| SRR3661121 | *Macaca mulatta* |
| SRR3661006 | *Macaca mulatta* |
| SRR594464 | *Macaca mulatta* |
| SRR3661029 | *Macaca mulatta* |
| SRR3660695 | *Macaca mulatta* |
| SRR3661102 | *Macaca mulatta* |
| SRR3661137 | *Macaca mulatta* |
| SRR2059383 | *Macaca mulatta* |
| SRR5088719 | *Macaca mulatta* |
| SRR3661090 | *Macaca mulatta* |
| SRR365028 | *Macaca mulatta* |
| SRR1952666 | *Macaca mulatta* |
| SRR3660753 | *Macaca mulatta* |
| SRR3660668 | *Macaca mulatta* |
| SRR3660646 | *Macaca mulatta* |
| SRR3660849 | *Macaca mulatta* |
| SRR3661072 | *Macaca mulatta* |
| SRR832960 | *Macaca mulatta* |
| SRR3661053 | *Macaca mulatta* |
| SRR2059389 | *Macaca mulatta* |
| SRR3660672 | *Macaca mulatta* |
| SRR3660752 | *Macaca mulatta* |
| SRR3661016 | *Macaca mulatta* |
| SRR3661089 | *Macaca mulatta* |
| SRR3660952 | *Macaca mulatta* |
| SRR3660873 | *Macaca mulatta* |
| SRR2059388 | *Macaca mulatta* |
| SRR3660975 | *Macaca mulatta* |
| SRR3661091 | *Macaca mulatta* |
| SRR3661100 | *Macaca mulatta* |
| SRR4304879 | *Macaca mulatta* |
| SRR3660977 | *Macaca mulatta* |
| SRR3661052 | *Macaca mulatta* |
| SRR2603329 | *Macaca mulatta* |
| SRR2674773 | *Macaca mulatta* |
| SRR4012425 | *Macaca mulatta* |
| SRR832963 | *Macaca mulatta* |
| SRR2467199 | *Macaca mulatta* |
| SRR3660639 | *Macaca mulatta* |
| SRR3661010 | *Macaca mulatta* |
| SRR3660706 | *Macaca mulatta* |
| SRR3661000 | *Macaca mulatta* |
| SRR950291 | *Macaca mulatta* |
| SRR3661116 | *Macaca mulatta* |
| SRR3661086 | *Macaca mulatta* |
| SRR2059418 | *Macaca mulatta* |
| SRR3660896 | *Macaca mulatta* |
| SRR3660680 | *Macaca mulatta* |
| SRR3661034 | *Macaca mulatta* |
| SRR3661071 | *Macaca mulatta* |
| SRR3661087 | *Macaca mulatta* |
| SRR3661020 | *Macaca mulatta* |
| SRR3661048 | *Macaca mulatta* |
| SRR3660976 | *Macaca mulatta* |
| SRR3660839 | *Macaca mulatta* |
| SRR3661117 | *Macaca mulatta* |
| SRR3660684 | *Macaca mulatta* |
| SRR2059419 | *Macaca mulatta* |
| SRR3660763 | *Macaca mulatta* |
| SRR3661130 | *Macaca mulatta* |
| SRR3660963 | *Macaca mulatta* |
| SRR950272 | *Macaca mulatta* |
| SRR3660916 | *Macaca mulatta* |
| SRR2059405 | *Macaca mulatta* |
| SRR950306 | *Macaca mulatta* |
| SRR3660913 | *Macaca mulatta* |
| SRR3660926 | *Macaca mulatta* |
| SRR3661074 | *Macaca mulatta* |
| SRR3660687 | *Macaca mulatta* |
| SRR950238 | *Macaca mulatta* |
| SRR1047646 | *Macaca mulatta* |
| SRR4242904 | *Macaca mulatta* |
| SRR950239 | *Macaca mulatta* |
| SRR3660938 | *Macaca mulatta* |
| SRR950307 | *Macaca mulatta* |
| SRR950325 | *Macaca mulatta* |
| SRR2059411 | *Macaca mulatta* |
| SRR3660817 | *Macaca mulatta* |
| SRR2059387 | *Macaca mulatta* |
| SRR3661073 | *Macaca mulatta* |
| SRR1048282 | *Macaca mulatta* |
| SRR3660921 | *Macaca mulatta* |
| SRR3660942 | *Macaca mulatta* |
| SRR950273 | *Macaca mulatta* |
| SRR3660853 | *Macaca mulatta* |
| SRR3661173 | *Macaca mulatta* |
| SRR4242899 | *Macaca mulatta* |
| SRR950257 | *Macaca mulatta* |
| SRR3660644 | *Macaca mulatta* |
| SRR2059400 | *Macaca mulatta* |
| SRR950225 | *Macaca mulatta* |
| SRR5088720 | *Macaca mulatta* |
| SRR3660782 | *Macaca mulatta* |
| SRR3660802 | *Macaca mulatta* |
| SRR1003035 | *Macaca mulatta* |
| SRR357411 | *Macaca mulatta* |
| SRR3309965 | *Macaca mulatta* |
| SRR365031 | *Macaca mulatta* |
| SRR3660634 | *Macaca mulatta* |
| SRR365032 | *Macaca mulatta* |
| SRR950260 | *Macaca mulatta* |
| SRR3660762 | *Macaca mulatta* |
| SRR3660635 | *Macaca mulatta* |
| SRR3660980 | *Macaca mulatta* |
| SRR3660778 | *Macaca mulatta* |
| SRR357439 | *Macaca mulatta* |
| SRR3661142 | *Macaca mulatta* |
| SRR306780 | *Macaca mulatta* |
| SRR5088735 | *Macaca mulatta* |
| SRR1051597 | *Macaca mulatta* |
| SRR3660937 | *Macaca mulatta* |
| SRR3660828 | *Macaca mulatta* |
| SRR3661085 | *Macaca mulatta* |
| SRR3660743 | *Macaca mulatta* |
| SRR3661079 | *Macaca mulatta* |
| SRR3660822 | *Macaca mulatta* |
| SRR4304867 | *Macaca mulatta* |
| SRR3661131 | *Macaca mulatta* |
| SRR3660827 | *Macaca mulatta* |
| SRR2059413 | *Macaca mulatta* |
| SRR331900 | *Macaca mulatta* |
| SRR3660936 | *Macaca mulatta* |
| SRR950228 | *Macaca mulatta* |
| SRR3660931 | *Macaca mulatta* |
| SRR5088734 | *Macaca mulatta* |
| SRR4242916 | *Macaca mulatta* |
| SRR950294 | *Macaca mulatta* |
| SRR1047643 | *Macaca mulatta* |
| SRR3661132 | *Macaca mulatta* |
| SRR594447 | *Macaca mulatta* |
| SRR4304874 | *Macaca mulatta* |
| SRR3660837 | *Macaca mulatta* |
| SRR2059392 | *Macaca mulatta* |
| SRR3660981 | *Macaca mulatta* |
| SRR3660636 | *Macaca mulatta* |
| SRR3309966 | *Macaca mulatta* |
| SRR3660876 | *Macaca mulatta* |
| SRR1047655 | *Macaca mulatta* |
| SRR950226 | *Macaca mulatta* |
| SRR1051588 | *Macaca mulatta* |
| SRR1047648 | *Macaca mulatta* |
| SRR3660891 | *Macaca mulatta* |
| SRR950232 | *Macaca mulatta* |
| SRR3660772 | *Macaca mulatta* |
| SRR4242889 | *Macaca mulatta* |
| SRR3661126 | *Macaca mulatta* |
| SRR4304897 | *Macaca mulatta* |
| SRR1047647 | *Macaca mulatta* |
| SRR3660901 | *Macaca mulatta* |
| SRR5088731 | *Macaca mulatta* |
| SRR1051593 | *Macaca mulatta* |
| SRR950283 | *Macaca mulatta* |
| SRR4242908 | *Macaca mulatta* |
| SRR3660791 | *Macaca mulatta* |
| SRR1051729 | *Macaca mulatta* |
| SRR2467175 | *Macaca mulatta* |
| SRR3660832 | *Macaca mulatta* |
| SRR3660911 | *Macaca mulatta* |
| SRR950234 | *Macaca mulatta* |
| SRR3660681 | *Macaca mulatta* |
| SRR3660757 | *Macaca mulatta* |
| SRR950259 | *Macaca mulatta* |
| SRR365027 | *Macaca mulatta* |
| SRR1047641 | *Macaca mulatta* |
| SRR1047652 | *Macaca mulatta* |
| SRR3660806 | *Macaca mulatta* |
| SRR2079383 | *Macaca mulatta* |
| SRR1048284 | *Macaca mulatta* |
| SRR3660842 | *Macaca mulatta* |
| SRR306785 | *Macaca mulatta* |
| SRR3660927 | *Macaca mulatta* |
| SRR1047644 | *Macaca mulatta* |
| SRR2079384 | *Macaca mulatta* |
| SRR3660847 | *Macaca mulatta* |
| SRR4242912 | *Macaca mulatta* |
| SRR1047653 | *Macaca mulatta* |
| SRR950317 | *Macaca mulatta* |
| SRR1004352 | *Macaca mulatta* |
| SRR3660957 | *Macaca mulatta* |
| SRR3660984 | *Macaca mulatta* |
| SRR2059414 | *Macaca mulatta* |
| SRR1047665 | *Macaca mulatta* |
| SRR950269 | *Macaca mulatta* |
| SRR1047651 | *Macaca mulatta* |
| SRR2059396 | *Macaca mulatta* |
| SRR3660932 | *Macaca mulatta* |
| SRR950293 | *Macaca mulatta* |
| SRR950249 | *Macaca mulatta* |
| SRR3660897 | *Macaca mulatta* |
| SRR1047660 | *Macaca mulatta* |
| SRR1047662 | *Macaca mulatta* |
| SRR306789 | *Macaca mulatta* |
| SRR3660738 | *Macaca mulatta* |
| SRR2079387 | *Macaca mulatta* |
| SRR2532182 | *Macaca mulatta* |
| SRR3660747 | *Macaca mulatta* |
| SRR4304900 | *Macaca mulatta* |
| SRR2059408 | *Macaca mulatta* |
| SRR1047654 | *Macaca mulatta* |
| SRR5088733 | *Macaca mulatta* |
| SRR950268 | *Macaca mulatta* |
| SRR3660767 | *Macaca mulatta* |
| SRR3660823 | *Macaca mulatta* |
| SRR3660807 | *Macaca mulatta* |
| SRR3660906 | *Macaca mulatta* |
| SRR1047658 | *Macaca mulatta* |
| SRR950285 | *Macaca mulatta* |
| SRR950262 | *Macaca mulatta* |
| SRR3661127 | *Macaca mulatta* |
| SRR1046130 | *Macaca mulatta* |
| SRR3660742 | *Macaca mulatta* |
| SRR950266 | *Macaca mulatta* |
| SRR1047649 | *Macaca mulatta* |
| SRR3660947 | *Macaca mulatta* |
| SRR2079396 | *Macaca mulatta* |
| SRR4012422 | *Macaca mulatta* |
| SRR3660922 | *Macaca mulatta* |
| SRR950303 | *Macaca mulatta* |
| SRR3660877 | *Macaca mulatta* |
| SRR3660801 | *Macaca mulatta* |
| SRR3660934 | *Macaca mulatta* |
| SRR3660902 | *Macaca mulatta* |
| SRR3660737 | *Macaca mulatta* |
| SRR3660946 | *Macaca mulatta* |
| SRR1051586 | *Macaca mulatta* |
| SRR3660816 | *Macaca mulatta* |
| SRR306788 | *Macaca mulatta* |
| SRR950240 | *Macaca mulatta* |
| SRR3660659 | *Macaca mulatta* |
| SRR3660941 | *Macaca mulatta* |
| SRR950288 | *Macaca mulatta* |
| SRR950308 | *Macaca mulatta* |
| SRR3660833 | *Macaca mulatta* |
| SRR950274 | *Macaca mulatta* |
| SRR950302 | *Macaca mulatta* |
| SRR2079388 | *Macaca mulatta* |
| SRR950265 | *Macaca mulatta* |
| SRR3660776 | *Macaca mulatta* |
| SRR950290 | *Macaca mulatta* |
| SRR1051596 | *Macaca mulatta* |
| SRR3660846 | *Macaca mulatta* |
| SRR4242975 | *Macaca mulatta* |
| SRR950230 | *Macaca mulatta* |
| SRR1051566 | *Macaca mulatta* |
| SRR306778 | *Macaca mulatta* |
| SRR2079392 | *Macaca mulatta* |
| SRR1051690 | *Macaca mulatta* |
| SRR1051587 | *Macaca mulatta* |
| SRR950296 | *Macaca mulatta* |
| SRR3660750 | *Macaca mulatta* |
| SRR4012427 | *Macaca mulatta* |
| SRR950319 | *Macaca mulatta* |
| SRR3660786 | *Macaca mulatta* |
| SRR3660917 | *Macaca mulatta* |
| SRR950300 | *Macaca mulatta* |
| SRR3660780 | *Macaca mulatta* |
| SRR3660858 | *Macaca mulatta* |
| SRR1051689 | *Macaca mulatta* |
| SRR1047656 | *Macaca mulatta* |
| SRR3660886 | *Macaca mulatta* |
| SRR950271 | *Macaca mulatta* |
| SRR950305 | *Macaca mulatta* |
| SRR950251 | *Macaca mulatta* |
| SRR950277 | *Macaca mulatta* |
| SRR950237 | *Macaca mulatta* |
| SRR3306983 | *Macaca mulatta* |
| SRR950311 | *Macaca mulatta* |
| SRR1047645 | *Macaca mulatta* |
| SRR950270 | *Macaca mulatta* |
| SRR950304 | *Macaca mulatta* |
| SRR950322 | *Macaca mulatta* |
| SRR950243 | *Macaca mulatta* |
| SRR3660686 | *Macaca mulatta* |
| SRR950299 | *Macaca mulatta* |
| SRR950236 | *Macaca mulatta* |
| SRR950287 | *Macaca mulatta* |
| SRR950324 | *Macaca mulatta* |
| SRR950235 | *Macaca mulatta* |
| SRR3660758 | *Macaca mulatta* |
| SRR950231 | *Macaca mulatta* |
| SRR950275 | *Macaca mulatta* |
| SRR950254 | *Macaca mulatta* |
| SRR1048283 | *Macaca mulatta* |
| SRR950309 | *Macaca mulatta* |
| SRR950276 | *Macaca mulatta* |
| SRR350011 | *Macaca mulatta* |
| SRR3660813 | *Macaca mulatta* |
| SRR950310 | *Macaca mulatta* |
| SRR950242 | *Macaca mulatta* |
| SRR3660859 | *Macaca mulatta* |
| SRR1048281 | *Macaca mulatta* |
| SRR3660674 | *Macaca mulatta* |
| SRR950241 | *Macaca mulatta* |
| SRR950256 | *Macaca mulatta* |
| SRR4304863 | *Macaca mulatta* |
| SRR306786 | *Macaca mulatta* |
| SRR3660890 | *Macaca mulatta* |
| SRR3660818 | *Macaca mulatta* |
| SRR4304853 | *Macaca mulatta* |
| SRR1051702 | *Macaca mulatta* |
| SRR950267 | *Macaca mulatta* |
| SRR3660848 | *Macaca mulatta* |
| SRR1051557 | *Macaca mulatta* |
| SRR3660766 | *Macaca mulatta* |
| SRR3660953 | *Macaca mulatta* |
| SRR2079385 | *Macaca mulatta* |
| SRR2079386 | *Macaca mulatta* |
| SRR2079390 | *Macaca mulatta* |
| SRR2079394 | *Macaca mulatta* |
| SRR2079402 | *Macaca mulatta* |
| SRR3660787 | *Macaca mulatta* |
| SRR3660826 | *Macaca mulatta* |
| SRR1047661 | *Macaca mulatta* |
| SRR4304883 | *Macaca mulatta* |
| SRR950284 | *Macaca mulatta* |
| SRR950321 | *Macaca mulatta* |
| SRR357410 | *Macaca mulatta* |
| SRR353359 | *Macaca mulatta* |
| SRR2059417 | *Macaca mulatta* |
| SRR3660812 | *Macaca mulatta* |
| SRR3660792 | *Macaca mulatta* |
| SRR3660862 | *Macaca mulatta* |
| SRR3660863 | *Macaca mulatta* |
| SRR4012424 | *Macaca mulatta* |
| SRR3660803 | *Macaca mulatta* |
| SRR3661064 | *Macaca mulatta* |
| SRR306781 | *Macaca mulatta* |
| SRR950253 | *Macaca mulatta* |
| SRR3660956 | *Macaca mulatta* |
| SRR3660951 | *Macaca mulatta* |
| SRR1051592 | *Macaca mulatta* |
| SRR3660875 | *Macaca mulatta* |
| SRR3660950 | *Macaca mulatta* |
| SRR1047659 | *Macaca mulatta* |
| SRR2079393 | *Macaca mulatta* |
| SRR3660808 | *Macaca mulatta* |
| SRR1047650 | *Macaca mulatta* |
| SRR950264 | *Macaca mulatta* |
| SRR950301 | *Macaca mulatta* |
| SRR1047657 | *Macaca mulatta* |
| SRR950286 | *Macaca mulatta* |
| SRR3660912 | *Macaca mulatta* |
| SRR3660765 | *Macaca mulatta* |
| SRR306783 | *Macaca mulatta* |
| SRR2059416 | *Macaca mulatta* |
| SRR3660871 | *Macaca mulatta* |
| SRR950263 | *Macaca mulatta* |
| SRR950278 | *Macaca mulatta* |
| SRR950229 | *Macaca mulatta* |
| SRR950312 | *Macaca mulatta* |
| SRR3660857 | *Macaca mulatta* |
| SRR950318 | *Macaca mulatta* |
| SRR950244 | *Macaca mulatta* |
| SRR3660907 | *Macaca mulatta* |
| SRR950289 | *Macaca mulatta* |
| SRR950250 | *Macaca mulatta* |
| SRR2079395 | *Macaca mulatta* |
| SRR3660798 | *Macaca mulatta* |
| SRR3660745 | *Macaca mulatta* |
| SRR4304892 | *Macaca mulatta* |
| SRR950298 | *Macaca mulatta* |
| SRR3660771 | *Macaca mulatta* |
| SRR3660843 | *Macaca mulatta* |
| SRR2059384 | *Macaca mulatta* |
| SRR4304865 | *Macaca mulatta* |
| SRR950320 | *Macaca mulatta* |
| SRR950252 | *Macaca mulatta* |
| SRR1051556 | *Macaca mulatta* |
| SRR950297 | *Macaca mulatta* |
| SRR3660777 | *Macaca mulatta* |
| SRR5088732 | *Macaca mulatta* |
| SRR1047664 | *Macaca mulatta* |
| SRR950323 | *Macaca mulatta* |
| SRR3660746 | *Macaca mulatta* |
| SRR3660760 | *Macaca mulatta* |
| SRR950255 | *Macaca mulatta* |
| SRR3660811 | *Macaca mulatta* |
| SRR950233 | *Macaca mulatta* |
| SRR950280 | *Macaca mulatta* |
| SRR950314 | *Macaca mulatta* |
| SRR950246 | *Macaca mulatta* |
| SRR3660756 | *Macaca mulatta* |
| SRR3660892 | *Macaca mulatta* |
| SRR3660924 | *Macaca mulatta* |
| SRR945323 | *Macaca mulatta* |
| SRR950279 | *Macaca mulatta* |
| SRR950313 | *Macaca mulatta* |
| SRR3660868 | *Macaca mulatta* |
| SRR1051728 | *Macaca mulatta* |
| SRR1051640 | *Macaca mulatta* |
| SRR4304899 | *Macaca mulatta* |
| SRR3660915 | *Macaca mulatta* |
| SRR950245 | *Macaca mulatta* |
| SRR4012416 | *Macaca mulatta* |
| SRR3660775 | *Macaca mulatta* |
| SRR3660781 | *Macaca mulatta* |
| SRR1051625 | *Macaca mulatta* |
| SRR3660872 | *Macaca mulatta* |
| SRR331898 | *Macaca mulatta* |
| SRR950282 | *Macaca mulatta* |
| SRR306777 | *Macaca mulatta* |
| SRR1003031 | *Macaca mulatta* |
| SRR1051634 | *Macaca mulatta* |
| SRR4012417 | *Macaca mulatta* |
| SRR553579 | *Macaca mulatta* |
| SRR4012418 | *Macaca mulatta* |
| SRR4304888 | *Macaca mulatta* |
| SRR2532193 | *Macaca mulatta* |
| SRR2674775 | *Macaca mulatta* |
| SRR3660838 | *Macaca mulatta* |
| SRR3660954 | *Macaca mulatta* |
| SRR4304887 | *Macaca mulatta* |
| SRR1051697 | *Macaca mulatta* |
| SRR1051613 | *Macaca mulatta* |
| SRR4242956 | *Macaca mulatta* |
| SRR4242980 | *Macaca mulatta* |
| ERR127386 | *Macaca mulatta* |
| SRR3660959 | *Macaca mulatta* |
| SRR3660884 | *Macaca mulatta* |
| SRR4304894 | *Macaca mulatta* |
| ERR1759744 | *Macaca mulatta* |
| SRR3660761 | *Macaca mulatta* |
| SRR950316 | *Macaca mulatta* |
| SRR3660840 | *Macaca mulatta* |
| SRR3660885 | *Macaca mulatta* |
| SRR3660751 | *Macaca mulatta* |
| SRR2079389 | *Macaca mulatta* |
| SRR3660770 | *Macaca mulatta* |
| SRR950281 | *Macaca mulatta* |
| ERR127390 | *Macaca mulatta* |
| SRR950248 | *Macaca mulatta* |
| SRR3660945 | *Macaca mulatta* |
| SRR950315 | *Macaca mulatta* |
| ERR127393 | *Macaca mulatta* |
| SRR950247 | *Macaca mulatta* |
| SRR353284 | *Macaca mulatta* |
| SRR2059403 | *Macaca mulatta* |
| SRR4242970 | *Macaca mulatta* |
| SRR3660882 | *Macaca mulatta* |
| SRR2532179 | *Macaca mulatta* |
| SRR3660815 | *Macaca mulatta* |
| ERR127387 | *Macaca mulatta* |
| ERR127391 | *Macaca mulatta* |
| SRR4304885 | *Macaca mulatta* |
| SRR2532176 | *Macaca mulatta* |
| SRR3660944 | *Macaca mulatta* |
| SRR1051624 | *Macaca mulatta* |
| SRR3660821 | *Macaca mulatta* |
| ERR127405 | *Macaca mulatta* |
| SRR3660949 | *Macaca mulatta* |
| SRR2059401 | *Macaca mulatta* |
| SRR4304872 | *Macaca mulatta* |
| SRR2674801 | *Macaca mulatta* |
| SRR1004359 | *Macaca mulatta* |
| SRR1051688 | *Macaca mulatta* |
| SRR306784 | *Macaca mulatta* |
| ERR127388 | *Macaca mulatta* |
| SRR1004346 | *Macaca mulatta* |
| SRR3660914 | *Macaca mulatta* |
| SRR945315 | *Macaca mulatta* |
| SRR3660852 | *Macaca mulatta* |
| SRR1051612 | *Macaca mulatta* |
| ERR127402 | *Macaca mulatta* |
| SRR1051696 | *Macaca mulatta* |
| SRR3660961 | *Macaca mulatta* |
| SRR1004337 | *Macaca mulatta* |
| ERR127400 | *Macaca mulatta* |
| SRR1051548 | *Macaca mulatta* |
| SRR3660797 | *Macaca mulatta* |
| SRR553577 | *Macaca mulatta* |
| ERR127403 | *Macaca mulatta* |
| SRR2079391 | *Macaca mulatta* |
| SRR3660887 | *Macaca mulatta* |
| SRR1051655 | *Macaca mulatta* |
| SRR945314 | *Macaca mulatta* |
| SRR3660939 | *Macaca mulatta* |
| SRR2674774 | *Macaca mulatta* |
| SRR3660866 | *Macaca mulatta* |
| SRR1051649 | *Macaca mulatta* |
| SRR553578 | *Macaca mulatta* |
| SRR353357 | *Macaca mulatta* |
| SRR2059404 | *Macaca mulatta* |
| SRR1051623 | *Macaca mulatta* |
| ERR127407 | *Macaca mulatta* |
| SRR1051555 | *Macaca mulatta* |
| ERR127404 | *Macaca mulatta* |
| SRR3660893 | *Macaca mulatta* |
| ERR127394 | *Macaca mulatta* |
| SRR1004336 | *Macaca mulatta* |
| SRR3660920 | *Macaca mulatta* |
| SRR2816053 | *Macaca mulatta* |
| SRR1004341 | *Macaca mulatta* |
| SRR4304854 | *Macaca mulatta* |
| SRR1051611 | *Macaca mulatta* |
| SRR2079397 | *Macaca mulatta* |
| SRR4012419 | *Macaca mulatta* |
| ERR127401 | *Macaca mulatta* |
| SRR2816026 | *Macaca mulatta* |
| SRR3660925 | *Macaca mulatta* |
| SRR3660935 | *Macaca mulatta* |
| SRR3660805 | *Macaca mulatta* |
| SRR1051654 | *Macaca mulatta* |
| SRR1051684 | *Macaca mulatta* |
| SRR2567998 | *Macaca mulatta* |
| ERR127395 | *Macaca mulatta* |
| SRR1004344 | *Macaca mulatta* |
| SRR1004349 | *Macaca mulatta* |
| SRR2296417 | *Macaca mulatta* |
| SRR3660960 | *Macaca mulatta* |
| SRR1051610 | *Macaca mulatta* |
| SRR1602562 | *Macaca mulatta* |
| SRR3660755 | *Macaca mulatta* |
| ERR127397 | *Macaca mulatta* |
| ERR127392 | *Macaca mulatta* |
| SRR3660889 | *Macaca mulatta* |
| SRR1004327 | *Macaca mulatta* |
| SRR1051619 | *Macaca mulatta* |
| SRR1051682 | *Macaca mulatta* |
| SRR2079398 | *Macaca mulatta* |
| ERR1759739 | *Macaca mulatta* |
| SRR1051622 | *Macaca mulatta* |
| SRR1004331 | *Macaca mulatta* |
| SRR299128 | *Macaca mulatta* |
| SRR1051683 | *Macaca mulatta* |
| SRR1004325 | *Macaca mulatta* |
| SRR1004322 | *Macaca mulatta* |
| SRR1004361 | *Macaca mulatta* |
| SRR3500047 | *Macaca mulatta* |
| SRR1051680 | *Macaca mulatta* |
| SRR945359 | *Macaca mulatta* |
| SRR1051621 | *Macaca mulatta* |
| SRR1051681 | *Macaca mulatta* |
| SRR1051732 | *Macaca mulatta* |
| SRR3500048 | *Macaca mulatta* |
| ERR127406 | *Macaca mulatta* |
| SRR3660784 | *Macaca mulatta* |
| SRR1051701 | *Macaca mulatta* |
| SRR1051687 | *Macaca mulatta* |
| SRR2532178 | *Macaca mulatta* |
| SRR1004328 | *Macaca mulatta* |
| SRR4304855 | *Macaca mulatta* |
| SRR1004330 | *Macaca mulatta* |
| SRR1051679 | *Macaca mulatta* |
| SRR1051620 | *Macaca mulatta* |
| SRR2079401 | *Macaca mulatta* |
| ERR127389 | *Macaca mulatta* |
| ERR127399 | *Macaca mulatta* |
| SRR3660851 | *Macaca mulatta* |
| SRR353358 | *Macaca mulatta* |
| SRR2079403 | *Macaca mulatta* |
| SRR299127 | *Macaca mulatta* |
| SRR2816148 | *Macaca mulatta* |
| SRR3660919 | *Macaca mulatta* |
| SRR2816051 | *Macaca mulatta* |
| SRR4304870 | *Macaca mulatta* |
| SRR1051661 | *Macaca mulatta* |
| SRR2816069 | *Macaca mulatta* |
| SRR3660779 | *Macaca mulatta* |
| SRR945313 | *Macaca mulatta* |
| ERR1759738 | *Macaca mulatta* |
| SRR2296421 | *Macaca mulatta* |
| SRR1004340 | *Macaca mulatta* |
| SRR299126 | *Macaca mulatta* |
| SRR1051653 | *Macaca mulatta* |
| SRR2816063 | *Macaca mulatta* |
| SRR353360 | *Macaca mulatta* |
| SRR1004326 | *Macaca mulatta* |
| SRR1051673 | *Macaca mulatta* |
| SRR3660739 | *Macaca mulatta* |
| SRR3660841 | *Macaca mulatta* |
| SRR2816068 | *Macaca mulatta* |
| SRR2079399 | *Macaca mulatta* |
| SRR3500039 | *Macaca mulatta* |
| SRR1051648 | *Macaca mulatta* |
| SRR945322 | *Macaca mulatta* |
| SRR945327 | *Macaca mulatta* |
| SRR3500040 | *Macaca mulatta* |
| SRR2079400 | *Macaca mulatta* |
| SRR1051584 | *Macaca mulatta* |
| SRR3660764 | *Macaca mulatta* |
| SRR3660904 | *Macaca mulatta* |
| SRR299124 | *Macaca mulatta* |
| SRR945351 | *Macaca mulatta* |
| SRR3660741 | *Macaca mulatta* |
| SRR1051671 | *Macaca mulatta* |
| SRR1004323 | *Macaca mulatta* |
| SRR2816092 | *Macaca mulatta* |
| SRR1051608 | *Macaca mulatta* |
| SRR949575 | *Macaca mulatta* |
| SRR3500043 | *Macaca mulatta* |
| SRR945329 | *Macaca mulatta* |
| SRR1004351 | *Macaca mulatta* |
| SRR2296423 | *Macaca mulatta* |
| SRR3309956 | *Macaca mulatta* |
| SRR1051633 | *Macaca mulatta* |
| SRR3500044 | *Macaca mulatta* |
| SRR945357 | *Macaca mulatta* |
| SRR2816061 | *Macaca mulatta* |
| SRR2816072 | *Macaca mulatta* |
| SRR4044033 | *Macaca mulatta* |
| SRR553581 | *Macaca mulatta* |
| SRR3660789 | *Macaca mulatta* |
| SRR945347 | *Macaca mulatta* |
| SRR2816088 | *Macaca mulatta* |
| SRR2816070 | *Macaca mulatta* |
| ERR127398 | *Macaca mulatta* |
| SRR945312 | *Macaca mulatta* |
| SRR1003033 | *Macaca mulatta* |
| SRR2296422 | *Macaca mulatta* |
| SRR1051672 | *Macaca mulatta* |
| SRR1051678 | *Macaca mulatta* |
| SRR1004343 | *Macaca mulatta* |
| SRR4044034 | *Macaca mulatta* |
| SRR1051585 | *Macaca mulatta* |
| SRR2816080 | *Macaca mulatta* |
| ERR127396 | *Macaca mulatta* |
| SRR1004333 | *Macaca mulatta* |
| SRR1004342 | *Macaca mulatta* |
| SRR1051609 | *Macaca mulatta* |
| SRR2816073 | *Macaca mulatta* |
| SRR3660749 | *Macaca mulatta* |
| SRR2296420 | *Macaca mulatta* |
| SRR3660899 | *Macaca mulatta* |
| SRR2816062 | *Macaca mulatta* |
| SRR3660855 | *Macaca mulatta* |
| SRR3500046 | *Macaca mulatta* |
| SRR3500045 | *Macaca mulatta* |
| SRR553605 | *Macaca mulatta* |
| SRR949576 | *Macaca mulatta* |
| SRR1051618 | *Macaca mulatta* |
| SRR3660955 | *Macaca mulatta* |
| SRR945333 | *Macaca mulatta* |
| SRR1004339 | *Macaca mulatta* |
| SRR4044029 | *Macaca mulatta* |
| SRR1004353 | *Macaca mulatta* |
| SRR299125 | *Macaca mulatta* |
| SRR4012420 | *Macaca mulatta* |
| SRR3660930 | *Macaca mulatta* |
| SRR1051700 | *Macaca mulatta* |
| SRR1004338 | *Macaca mulatta* |
| SRR1051615 | *Macaca mulatta* |
| SRR2296419 | *Macaca mulatta* |
| SRR1051652 | *Macaca mulatta* |
| SRR1051669 | *Macaca mulatta* |
| SRR4012421 | *Macaca mulatta* |
| SRR3660820 | *Macaca mulatta* |
| SRR3660831 | *Macaca mulatta* |
| SRR1051677 | *Macaca mulatta* |
| SRR3660785 | *Macaca mulatta* |
| SRR3660900 | *Macaca mulatta* |
| SRR949580 | *Macaca mulatta* |
| SRR2816087 | *Macaca mulatta* |
| SRR3500041 | *Macaca mulatta* |
| SRR3660790 | *Macaca mulatta* |
| SRR945325 | *Macaca mulatta* |
| SRR4044030 | *Macaca mulatta* |
| SRR945339 | *Macaca mulatta* |
| SRR1004354 | *Macaca mulatta* |
| SRR116844 | *Macaca mulatta* |
| SRR3500042 | *Macaca mulatta* |
| SRR1051565 | *Macaca mulatta* |
| SRR1051676 | *Macaca mulatta* |
| SRR2816083 | *Macaca mulatta* |
| SRR2816085 | *Macaca mulatta* |
| SRR4012423 | *Macaca mulatta* |
| SRR4044032 | *Macaca mulatta* |
| SRR1004348 | *Macaca mulatta* |
| SRR3660908 | *Macaca mulatta* |
| SRR1004358 | *Macaca mulatta* |
| SRR1051617 | *Macaca mulatta* |
| SRR2816081 | *Macaca mulatta* |
| DRR077165 | *Macaca mulatta* |
| SRR553606 | *Macaca mulatta* |
| SRR1051616 | *Macaca mulatta* |
| SRR1051695 | *Macaca mulatta* |
| SRR1051670 | *Macaca mulatta* |
| SRR3660836 | *Macaca mulatta* |
| SRR1004329 | *Macaca mulatta* |
| SRR1004332 | *Macaca mulatta* |
| SRR3660905 | *Macaca mulatta* |
| SRR2816033 | *Macaca mulatta* |
| SRR2816049 | *Macaca mulatta* |
| SRR949579 | *Macaca mulatta* |
| SRR2816066 | *Macaca mulatta* |
| SRR2816134 | *Macaca mulatta* |
| SRR3660856 | *Macaca mulatta* |
| SRR945343 | *Macaca mulatta* |
| SRR2816043 | *Macaca mulatta* |
| SRR2816089 | *Macaca mulatta* |
| SRR2296418 | *Macaca mulatta* |
| SRR2816045 | *Macaca mulatta* |
| SRR3660825 | *Macaca mulatta* |
| SRR2816025 | *Macaca mulatta* |
| SRR2816078 | *Macaca mulatta* |
| SRR1051710 | *Macaca mulatta* |
| SRR945335 | *Macaca mulatta* |
| SRR1051686 | *Macaca mulatta* |
| SRR4012426 | *Macaca mulatta* |
| SRR1003028 | *Macaca mulatta* |
| SRR1004356 | *Macaca mulatta* |
| SRR945369 | *Macaca mulatta* |
| SRR2816044 | *Macaca mulatta* |
| SRR116845 | *Macaca mulatta* |
| SRR1051583 | *Macaca mulatta* |
| SRR1051554 | *Macaca mulatta* |
| SRR3660861 | *Macaca mulatta* |
| ERR1759737 | *Macaca mulatta* |
| SRR2816030 | *Macaca mulatta* |
| SRR553580 | *Macaca mulatta* |
| SRR2816060 | *Macaca mulatta* |
| SRR3660830 | *Macaca mulatta* |
| SRR945319 | *Macaca mulatta* |
| SRR1051668 | *Macaca mulatta* |
| SRR3660774 | *Macaca mulatta* |
| SRR1051667 | *Macaca mulatta* |
| SRR4044031 | *Macaca mulatta* |
| SRR1051632 | *Macaca mulatta* |
| SRR1051665 | *Macaca mulatta* |
| SRR3660881 | *Macaca mulatta* |
| SRR2816034 | *Macaca mulatta* |
| SRR1004345 | *Macaca mulatta* |
| SRR3660845 | *Macaca mulatta* |
| SRR1004357 | *Macaca mulatta* |
| SRR2816077 | *Macaca mulatta* |
| SRR3660860 | *Macaca mulatta* |
| SRR3660870 | *Macaca mulatta* |
| SRR1004335 | *Macaca mulatta* |
| SRR1051582 | *Macaca mulatta* |
| SRR945337 | *Macaca mulatta* |
| SRR1004324 | *Macaca mulatta* |
| SRR1051542 | *Macaca mulatta* |
| SRR1004347 | *Macaca mulatta* |
| SRR1051666 | *Macaca mulatta* |
| SRR949582 | *Macaca mulatta* |
| SRR2816065 | *Macaca mulatta* |
