# Supplementary material for: Novel hepaci- and pegi-like viruses in native Australian wildlife and non-human primates
Source: Virus Evol. 2020 Aug 20;6(2):veaa064. doi: 10.1093/ve/veaa064 (PMC7673076; doi:10.1093/ve/veaa064)
Supplement: veaa064_Supplementary_Data [file veaa064_supplementary_data.zip › Porter.Supplementary Table 5.Revised.docx]

**Supplementary Table 5.** Accession numbers and description of the hepaci- and pegi-like virus amino acid sequences used in the phylogenetic analysis.

| **Accession** | **Description** |
| --- | --- |
| KC411796 | Rodent hepacivirus |
| YP_009506360.1 | Hepacivirus J |
| AZA36446.1 | Sifaka hepacivirus |
| AYP67560 | Collins beach virus |
| YP_009506358.1 | Hepacivirus F |
| ATP66832.1 | Rodent hepacvirus |
| YP_007905733.1 | Rodent hepacivirus |
| AVW79985.1 | Rodent hepacivirus |
| YP_009109557.1 | Norway rat hepacivirus 1 |
| ASM47312.1 | Norway rat hepacivirus 1 |
| YP_009553586.1 | Hepacivirus P |
| YP_009109558.1 | Norway rat hepacivirus 2 |
| YP_009506356.1 | Non-primate hepacivirus NZP1 |
| AFV91338 | Canine hepacivirus |
| AFJ20705.1 | Hepacivirus AK-2012 |
| AXB50120.1 | Equine hepacivirus |
| AXB50121.1 | Equine hepacivirus |
| AFJ20709.1 | Hepacivirus AK-2012 |
| AXB50128.1 | Equine hepacivirus |
| AFJ20704.1 | Hepacivirus AK-2012 |
| YP_009058898.1 | Equine hepacivirus JPN3 |
| AFJ20706.1 | Hepacivirus AK-2012 |
| NP_671491 | Hepatitis C virus genotype 1 |
| YP_001469632.1 | Hepatitis C virus genotype 4 |
| YP_001469634.1 | Hepatitis C virus genotype 6 |
| YP_001469633.1 | Hepatitis C virus genotype 5 |
| YP_001469631.1 | Hepatitis C virus genotype 3 |
| YP_001469630.1 | Hepatitis C virus genotype 2 |
| YP_009272536.1 | Hepatitis C virus genotype 7 |
| YP_009325330.1 | Bat hepacivirus |
| YP_009506361.1 | Hepacivirus K |
| AGQ22075.1 | Guereza hepacivirus |
| YP_009325369.1 | Guereza hepacivirus |
| YP_009322132.1 | Hepacivirus L |
| YP_009506363.1 | Hepacivirus N |
| AKH10572.1 | Bovine hepacivirus |
| YP_009130616.1 | Bovine hepacivirus |
| AKA20771.1 | Bovine hepacivirus |
| YP_009506359.1 | Hepacivirus I |
| AGC52837.1 | Rodent hepacivirus |
| AWU67496.1 | Sigmodontinae hepacivirus |
| NP_056931.1 | Hepatitis GB virus B |
| BAK24073.1 | Hepatitis GB virus B |
| BAK24074.1 | Hepatitis GB virus B |
| BAK24075.1 | Hepatitis GB virus B |
| NP_042692.1 | Hepacivirus B |
| MG599999 | softshell turtle hepacivirus |
| AVM87260.1 | Chinese broad-headed pond turtle hepacivirus |
| AVM87253.1 | Xiamen guitarfish hepacivirus |
| AVM87258.1 | Nanhai ghost shark hepacivirus 2 |
| ALL52891.1 | Wenling shark virus |
| AVM87256.1 | Guangxi houndshark hepacivirus |
| AVM87254.1 | Xiamen sepia Stingray hepacivirus |
| MN133813 | jogolong hepacivirus |
| QFR04963.1 | Bald eagle hepacivirus |
| QDF44088.1 | Duck hepacivirus |
| QDF44087.1 | Duck hepacivirus |
| QDF44086.1 | Duck hepacivirus |
| AVM87255.1 | Western African lungfish hepacivirus |
| AVM87257.1 | Nanhai dogfish shark hepacivirus |
| AWM96387.1 | Hepacivirus sp. |
| NP_045010.1 | Pegivirus A |
| T08841 | Douroucouli hepatitis GB virus A |
| AAC99765.1 | GB virus C |
| BAA13321.1 | GB virus C |
| AZU96895.1 | GB virus C |
| AJS14317.1 | Human pegivirus genotype 2 |
| AAG26007.1 | Hepatitis G virus isolate PEI |
| QDK56780.1 | Human pegivirus genotype 1 |
| NP_043570.1 | GB virus C |
| QGN67998.1 | GB virus C |
| AEQ91881.1 | GB virus C |
| AIZ47339.1 | GB virus C |
| CVH74177.1 | Human pegivirus |
| AKP06520.1 | GB virus C |
| AHA61261.1 | GB virus C |
| AXN77727.1 | Human pegivirus |
| AYD60143.1 | Human pegivirus |
| AHH32931.1 | Simian pegivirus |
| YP_009044207.1 | Simian pegivirus |
| ALO60267.1 | Simian pegivirus |
| AJP36585.1 | Simian pegivirus |
| AGK41003.1 | Bat pegivirus |
| YP_009256194.1 | Pegivirus B |
| AGK41018.1 | Pegivirus I |
| ANO81672.1 | Porcine pegivirus |
| YP_009361867.1 | Pegivirus K |
| QAX58264.1 | Porcine pegivirus |
| AZY88695.1 | Dolphin pegivirus |
| AGH70217.1 | Theiler's disease-associated virus |
| AWV91977.1 | Theiler's disease-associated virus |
| ASS36970.1 | Equine pegivirus |
| YP_007697649.1 | Equine pegivirus 1 |
| AVR58712.1 | Equine pegivirus |
| ARF06726.1 | Human hepegivirus |
| AUW64508.1 | Human hepegivirus |
| AWD84264.1 | Human hepegivirus |
| AVR48551.1 | Human hepegivirus 1 |
| YP_009506367.1 | Human hepegivirus |
| YP_009227295.1 | Human pegivirus 2 |
| AYN72347.1 | Human pegivirus 2 |
| AVW79981.1 | Rodent pegivirus |
| YP_009109602.1 | Norway rat pegivirus |
| YP_007905734.1 | Rodent pegivirus |
| YP_009506365.1 | Pegivirus F |
| YP_009506366.1 | Pegivirus G |
| AVM87252.1 | Guangxi chinese leopard gecko hepacivirus |
| AVM87251.1 | Yili teratoscincus roborowskii hepacivirus |
| AVM87555.1 | Wenling moray eel hepacivirus |
