# Supplementary figures and images for: Novel hepaci- and pegi-like viruses in native Australian wildlife and non-human primates
Source: Virus Evol. 2020 Aug 20;6(2):veaa064. doi: 10.1093/ve/veaa064 (PMC7673076; doi:10.1093/ve/veaa064)

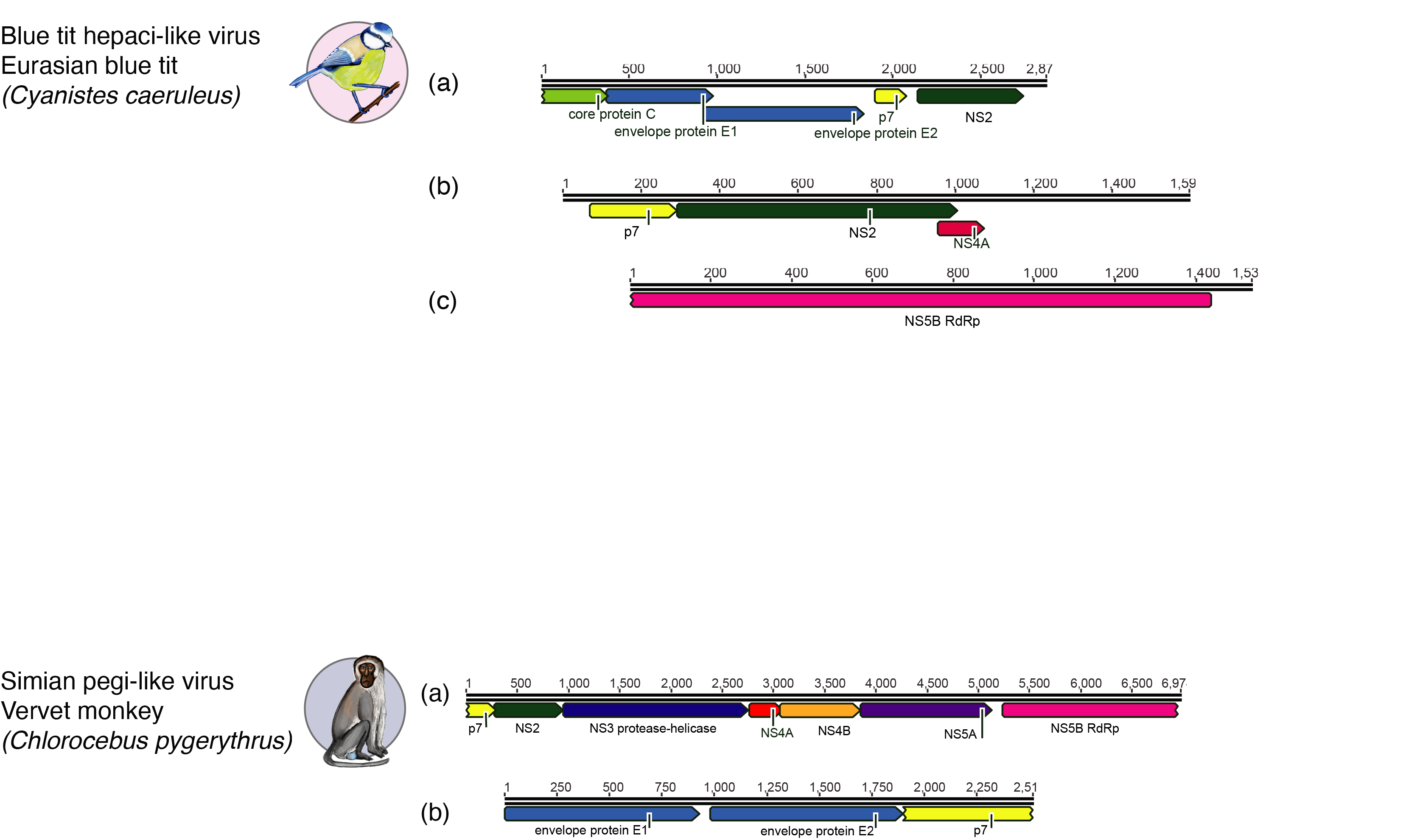

Supplement: veaa064_Supplementary_Data [file veaa064_supplementary_data.zip › Porter.Figure S1.png]
